# Optimized Conditions for Passerini-Smiles Reactions and Applications to Benzoxazinone Syntheses

**DOI:** 10.3390/molecules21091257

**Published:** 2016-09-21

**Authors:** Elodie Martinand-Lurin, Aurélie Dos Santos, Emmanuelle Robineau, Pascal Retailleau, Philippe Dauban, Laurence Grimaud, Laurent El Kaïm

**Affiliations:** 1 Institut de Chimie des Substances Naturelles, CNRS UPR 2301, Univ. Paris-Sud, Université Paris-Saclay, Avenue de la Terrasse, 91198 Gif-sur-Yvette CEDEX, France; Elodie.MARTINAND-LURIN@cnrs.fr (E.M.-L.); pascal.retailleau@icsn.cnrs-gif.fr (P.R.); Philippe.DAUBAN@cnrs.fr (P.D.); 2 1 Ecole Normale Supérieure, PSL Research University, UPMC Univ. Paris 06, CNRS, Département de Chimie, PASTEUR, 24, rue Lhomond, 75005 Paris, France; 2 Sorbonne Universités, UPMC Univ. Paris 06, ENS, CNRS, PASTEUR, 75005 Paris, France; Emmanuelle.robineau@ens.fr; 3 Laboratoire de Synthèse Organique, CNRS, Ecole Polytechnique, ENSTA ParisTech–UMR 7652, Université Paris-Saclay, 91128 Palaiseau, France; aurelie.dossantos@ensta-paristech.fr

**Keywords:** isocyanide, Passerini-Smiles, multicomponent reaction, heterocycles, benzoxazinones

## Abstract

Initial conditions disclosed for the Passerini-Smiles reaction are associated with a lack of efficiency that has prevented chemists from using it since its discovery. We wish to report herein our thorough study in the development of new experimental conditions for this coupling between electron-poor phenols, isocyanides, and carbonyl derivatives. These new conditions have been applied to several synthetic strategies towards benzoxazinones.

## 1. Introduction

In the last decade, we developed a variant of Ugi couplings in which the carboxylic acid is replaced with an electron-deficient phenol. Due to the nature of the final rearrangement involved in the process, we named this extension Ugi-Smiles coupling [1,2,3,4]. The potential of this new reaction was thoroughly studied by exploring the scope of the various phenols and related hydroxy heterocycles prone to react in this coupling and by developing efficient and rapid heterocyclic syntheses through the development of post-condensation transformations of the adducts. Soon after our first report on Ugi-Smiles coupling, we reported its three-component version, which involves a carbonyl derivative, an isocyanide and an electron-deficient phenol. In analogy with the previous four-component reaction, the term Passerini-Smiles reaction was used to coin these couplings [5]. However, the latter turned out to be of rather limited scope and its potential was, thus, neglected in favor of the more efficient Ugi-Smiles transformation. Herein, we wish to report all our results on improved conditions for the Passerini-Smiles coupling and their use in new heterocyclic syntheses.

## 2. Results

**Scheme 1:**
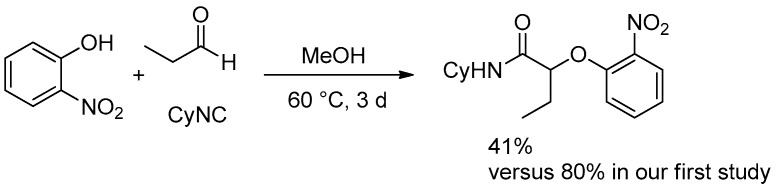
Passerini-Smiles couplings of *ortho*-nitrophenol according to previous conditions.

We recently reinvestigated the Passerini-Smiles reaction in order to study possible transformations of the three-component adducts [6]. Actually, when a stoichiometric amount of propanal, cyclohexylisocyanide, and 2-nitrophenol was stirred in methanol at 60 °C for three days, as previously settled, we were surprised to be unable to reproduce our previous results (80% of isolated yields), whatever the conditions of time and temperature (Scheme 1). We surmised that these divergent results could be due to an uncontrolled evaporation of the solvent during the reaction time and decided to investigate this reaction under neat conditions.

**Scheme 2:**
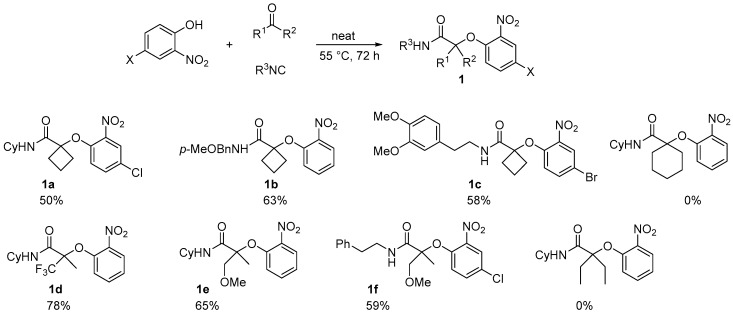
Neat Passerini-Smiles for ketones.

When performed with an activated ketone, the neat Passerini-Smiles proceeded smoothly giving the desired product in good yields. Indeed, cyclobutanone, trifluoromethylacetone, and methoxy acetone gave the desired product within 72 h in good yields whereas no adduct could be formed when using pentan-3-one and cyclohexanone under the same conditions (Scheme 2).

**Scheme 3:**
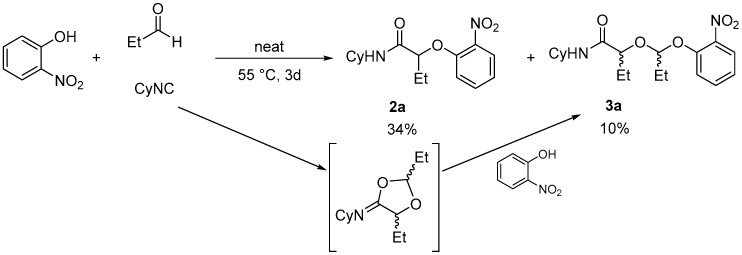
Neat Passerini-Smiles for aldehydes and byproduct formation.

However when an aldehyde, such as propanal, was chosen as the electrophilic partner, no improvement was observed for the reaction and the isolation of **2a** turned out to be difficult as an impurity is formed along with the product. This byproduct turned out to be the ketal **3a** formed by the incorporation of a second aldehyde and from the nitrophenol which triggered ring-opening of a cyclic imidate (Scheme 3). Probably due to higher steric hindrance, the ketal byproduct was never observed for ketones, even when used in excess.

**Scheme 4:**
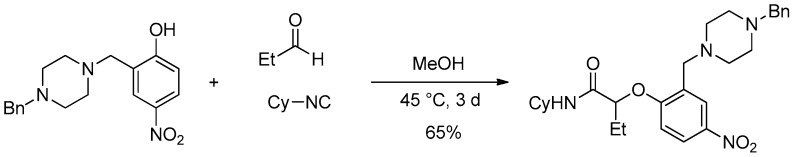
Mannich-modified *para*-nitrophenol in Passerini-Smiles couplings.

These first results encouraged us to further optimize the Passerini-Smiles reaction with aldehydes. During our former studies, we have observed that low yields of products were isolated in the case of 4-nitrophenol, probably due to the impossibility to activate the hydroxy group through a hydrogen bond occuring during the Smiles rearrangement (as demonstrated for Ugi-Smiles coupling) [7]. Nevertheless, a modified 4-nitrophenol was prepared via a Mannich reaction and showed a particular efficiency in the three-component (Scheme 4). Indeed, the piperazinyl-modified 4-nitrophenol afforded the desired adduct in 65% isolated yield after three days in MeOH.

**Scheme 5:**
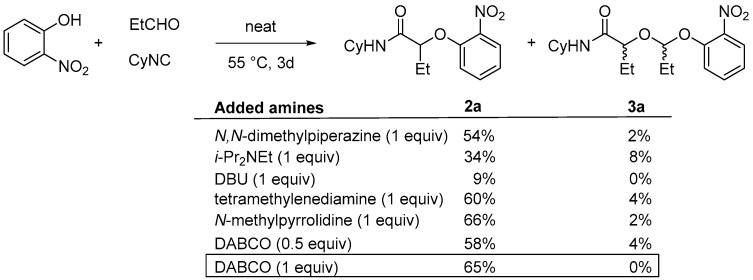
Additives in Passerini-Smiles reaction.

We, thus, decided to test the Passerini-Smiles in the presence of additives such as tertiary amines [8]. Various amines were tested for this purpose in the reaction of a stoichiometric amount of propanal, cyclohexylisocyanide and 2-nitrophenol (Scheme 5). The formation of the Passerini-Smiles adduct **2a** was not improved when adding tertiary amines such as the Hunig′s base. However, the use of more nucleophilic tertiary amines dramatically impacted the reaction rate and yields. The best results were obtained with DABCO (1,4-diazabicyclo[2.2.2]octane). Indeed, the addition of 1 equivalent of DABCO afforded **2a** in 65% isolated yield with no traces of byproduct.

## 3. Discussion

**Figure 1:**
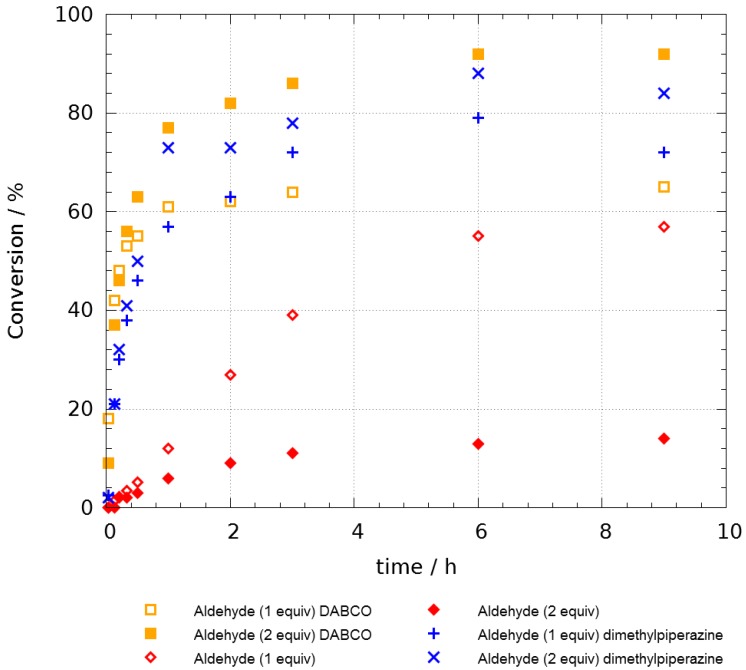
Formation of **2a** with time under various conditions.

In order to prove the effect of additives, we then performed kinetic studies (See Supplementary Materials) with 1 and 2 equivalents of propanal (Figure 1). The above reaction was done at 55 °C and the conversion was determined and plotted against time. This study confirmed that piperazine and DABCO both accelerated the reaction as the half-reaction time is under one hour in their presence. However, whatever the additives are, the conversion seemed to be limited to 70% after one day. We decided, thus, to use an excess of aldehyde and, under these optimized conditions, we were pleased to observe a very high level of conversion within a few hours with no trace of ketal **3a** as a byproduct. The role of the additive is still unexplained at the moment but in order to rationalize the inhibition of the by-path towards **3a**, we proposed that the aldehyde could be momentaneously stored as a hemiaminal intermediate. This would lower the concentration of the aldehyde in the medium and avoid the addition of a second aldehyde before the Smiles process.

**Scheme 6:**
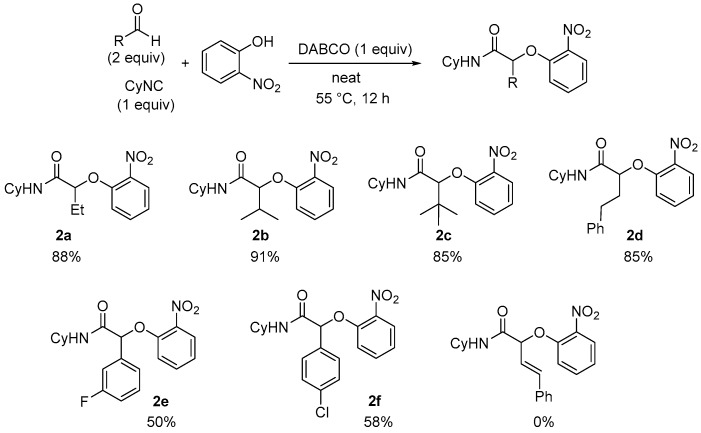
Passerini-Smiles couplings of aldehyde in the presence of DABCO.

Under these new optimized conditions, the scope of the Passerini-Smiles coupling was further investigated by varying the three reagents. The reaction was first tested using 2-nitrophenol and cyclohexylisocyanide, and varying the aldehyde partner. The reaction is highly efficient with aliphatic aldehydes, but aromatic aldehydes give only moderate yields (Scheme 6). As previously observed for Ugi-Smiles couplings, no adduct could be isolated from α,β-unsaturated aldehydes.

**Scheme 7:**
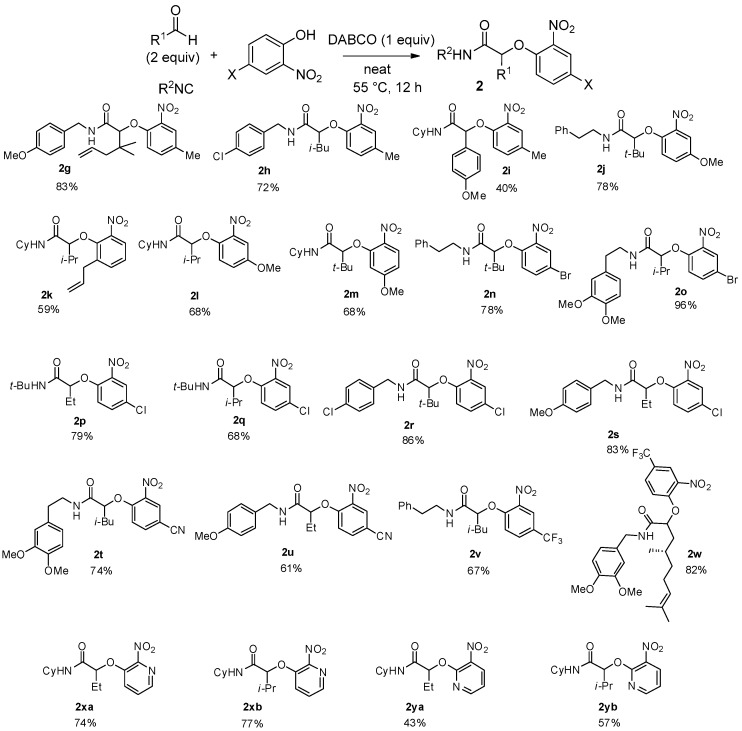
Passerini-Smiles couplings of substituted 2-nitro phenol and hydroxyl heterocycles.

The scope was next tested using substituted 2-nitrophenols. The nature of the substituent did not affect the efficiency of the reaction as electron-donating groups such as methyl, methoxy, or allyl and electron-withdrawing groups—halogen atom, cyano or trifluoromethyl—gave the desired adducts **2** in good yields (Scheme 7). It is worth noting that different isocyanides—cyclohexyl, *tert*-butyl, benzylic, etc.—can be used in this reaction with constant efficiency. These optimized conditions were also successfully applied to heterocyclic compounds such as hydroxyl nitropyridines and the corresponding Passerini-Smiles adducts **2x** and **2y** were isolated in good yields (Scheme 7).

**Scheme 8:**
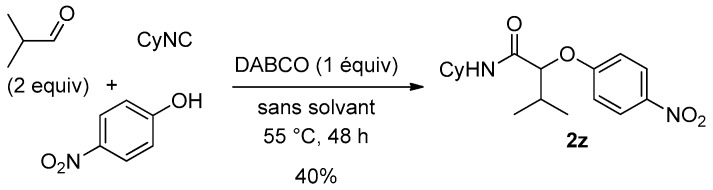
Passerini-Smiles couplings of 4-nitrophenol.

Surprisingly, using this new set of experimental conditions, the 4-nitrophenol was successfully coupled with isobutyraldehyde and cyclohexylisocyanide to give the desired adduct **2z** in 40% yield after two days along with about 15% of the corresponding acetal (Scheme 8).

**Scheme 9:**
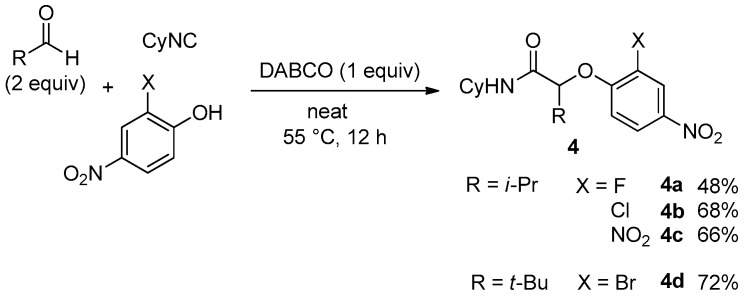
Passerini-Smiles couplings of substituted 4-nitrophenols.

Due to our theoretical studies concerning the mechanism of the Ugi-Smiles coupling [9], we assumed that 2-substituted 4-nitrophenol could behave more efficiently due to the possibility for the substituent to interact with the hydroxy group through an intramolecular hydrogen bond. This interaction should stabilize the spiro intermediate along the Smiles rearrangement. Therefore, we started to investigate 2-heterosubstituted-4-nitrophenols in this coupling. 2,4-dinitrophenol and 2-chloro or 2-bromo substituted 4-nitrophenols gave the desired **4** with good yields, but 2-fluoro-4-nitrophenol gave lower conversions after 12 h as the isolated yield of **4a** did not exceed 50% (Scheme 9).

**Scheme 10:**
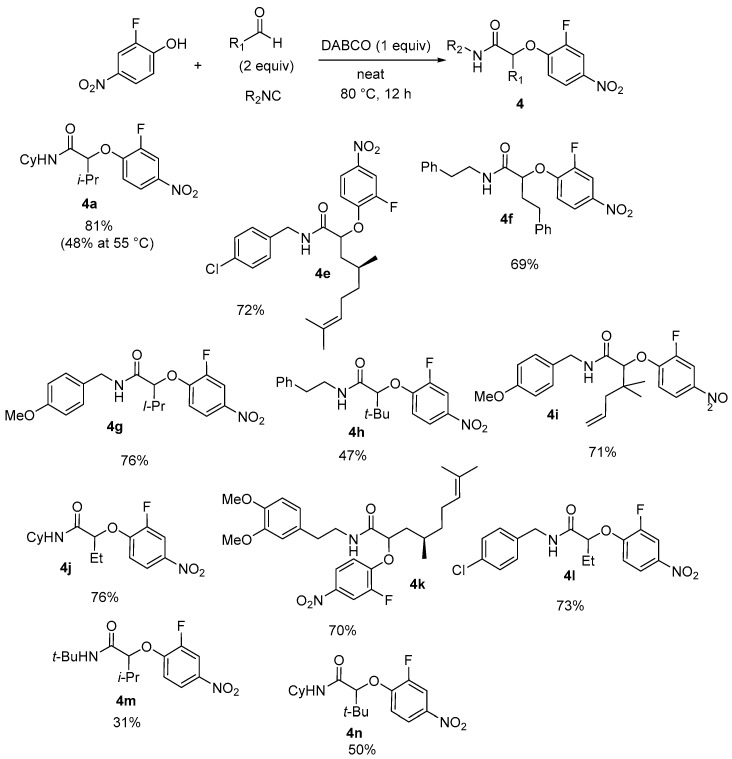
Passerini-Smiles couplings of substituted 2-fluoro-4-nitrophenol.

After 12 h of heating at 80 °C—instead of 60 °C—various isocyanides and aliphatic aldehydes were coupled with 2-fluoro-4-nitrophenol to form the three-component adducts in yields ranging from 31% to 81% (Scheme 10).

**Scheme 11:**
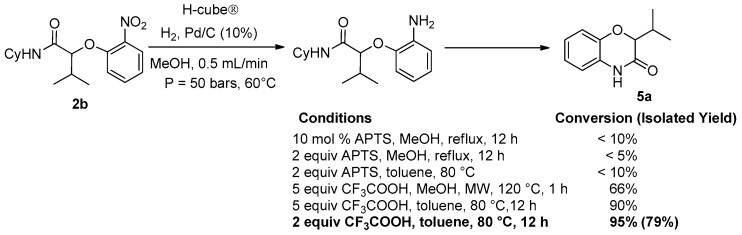
Reduction and acidic cyclization towards benzoxazinones.

Considering the high potential of Passerini and Ugi adducts in heterocyclic synthesis, we decided to investigate further post-condensation transformations of Passerini-Smiles adducts. The presence of the nitro group affords easy access to the corresponding anilines by direct reduction. Indeed, we recently reported a two step sequence involving a hydrogenolysis and an acidic cyclization towards the synthesis of NH-benzoxazinones as already done with Ugi-Smiles adducts a few years ago [10]. However, in contrast to our previous results, in this case, the whole sequence turned out to be difficult and required harsh conditions to proceed. Indeed, the reduction towards aniline was achieved using Pd/C (10%) at 60 °C in a H-cube apparatus under high pressure conditions (50 bars) and then the crude aniline was treated with two equivalents of trifluoroacetic acid in toluene at 80 °C overnight (Scheme 11).

**Scheme 12:**
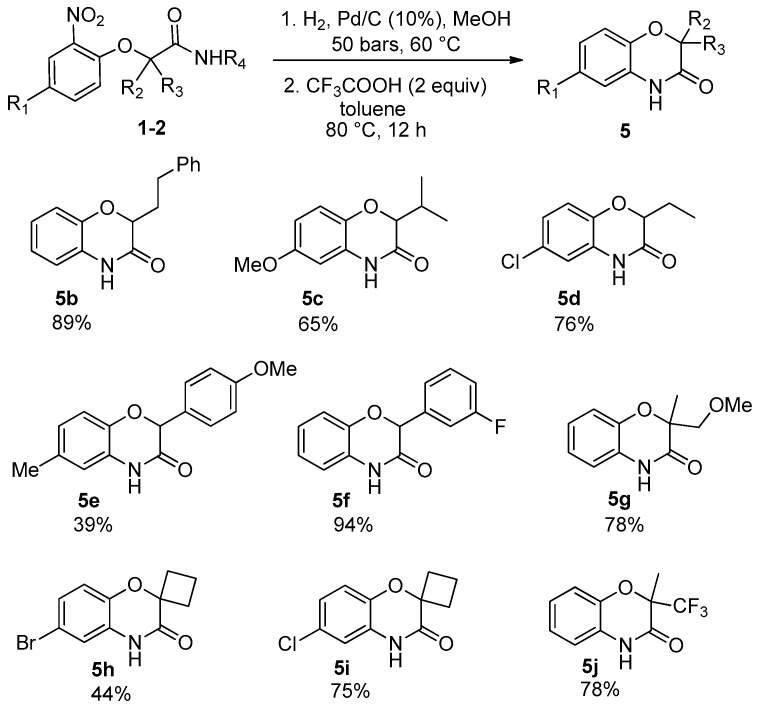
Benzoxazinone synthesis through a two-step cascade from Passerini-Smiles adducts.

Under these experimental conditions, the desired benzoxazinones **5** were synthesized in moderate to excellent yields (Scheme 12). However, in this synthesis, the loss of the former isocyanide moiety reduces the molecular diversity traditionally associated with Passerini couplings.

**Scheme 13:**
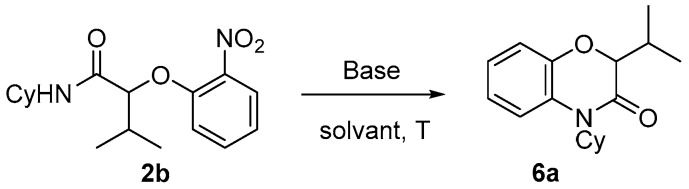
Optimization for the second Smiles rearrangement.

When considering the literature upon Smiles rearrangements, an impressive number of reactions involves the transfer of the aromatic moiety from an oxygen atom to the nitrogen atom of an amide under basic conditions [11,12,13]. Due to the structure of Passerini-Smiles adducts, we surmised that it could be possible to perform a second Smiles rearrangement in order to obtain *N*-aryl α-hydroxy carboxamides. The Passerini-Smiles product **2b** was, thus, treated with sodium hydride in DMF. At room temperature, no evolution occurred. However, after 1 h at 100 °C, a new cyclized product was formed. The latter turned out to a *N*-alkyl benzoxazinone **6a**. The reaction was further optimized by varying both the solvent and the base. Potassium *tert*-butoxide in DMF (at a concentration of 0.2 M) at 100 °C turned out to be the best set of experimental conditions (Scheme 13).

**Scheme 14:**
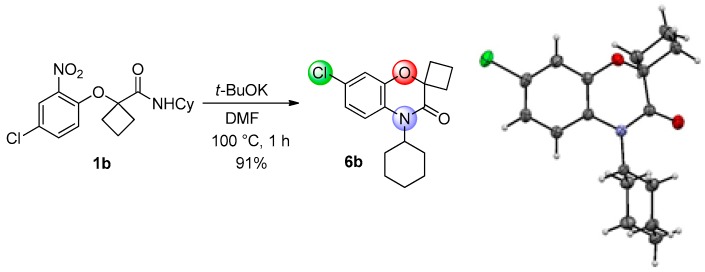
Smiles rearrangement for substrate **1b**.

This method constitutes an alternative to the former NH-benzoxazinone formation as in the present case, the benzoxazinone presents all three points of diversity of the Passerini-Smiles reaction. In order to precisely determine the mechanism of this cyclization, compound **1b** was then submitted to the cyclization conditions. The structure of the resulting product **6b** was undoubtfully assigned through X-ray analyses (Scheme 14).

**Scheme 15:**
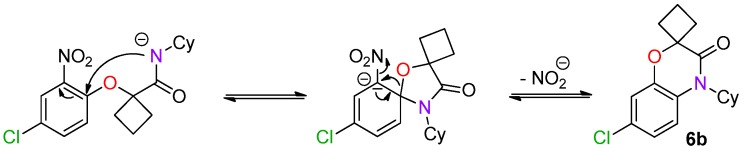
Plausible Mechanism for the Smiles rearrangement.

As the chlorine atom was located at the para-position of the amide, a second Smiles rearrangement was involved and the spiro ring-opening probably occurred with concomittant substitution of the nitro by the released alkoxide (Scheme 15) [11,12,13,14]. Similar concerted processes have been clearly evidenced by theoretical calculations [15].

**Scheme 16:**
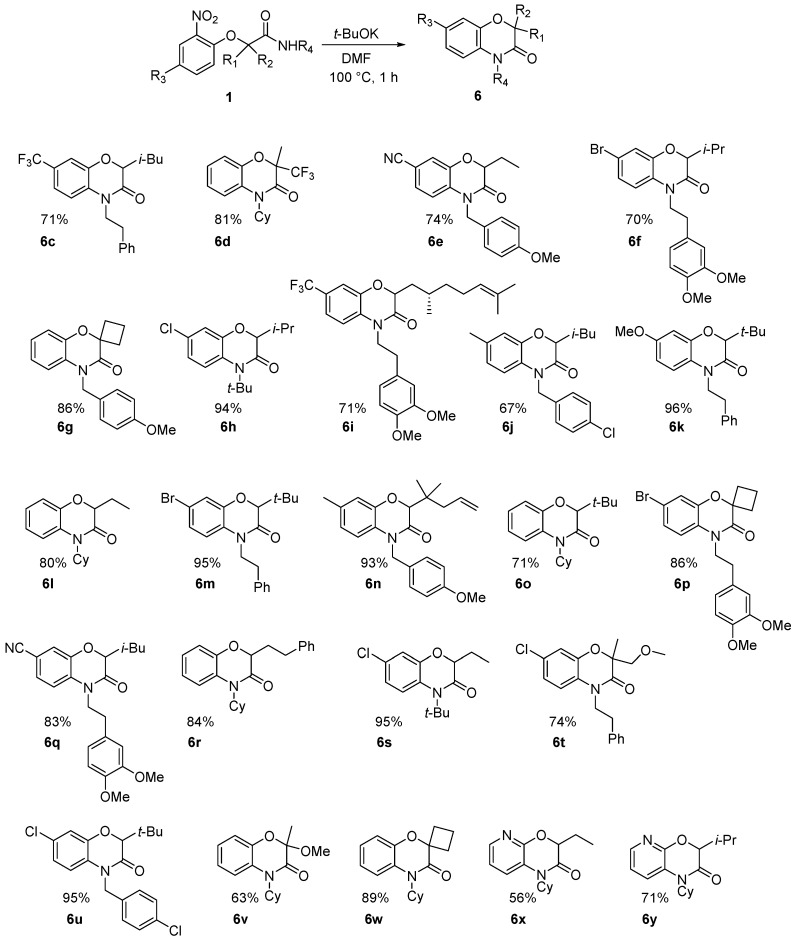
Scope for the second Smiles rearrangement and benzoxazinone formation.

**Scheme 17:**
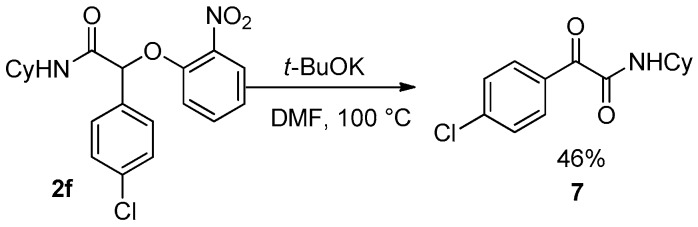
Oxidative cleavage for Passerini-Smiles adducts with aromatic substituent under anionic conditions.

Various benzoxazinones **6** were formed under these conditions as most of the Passerini-Smiles substrates **1** and **2** derived from 2-nitrophenols were successfully transformed under basic conditions with good to excellent yields (Scheme 16). Similarly, Passerini-adducts derived from hydroxy heterocycles were cyclized to form the corresponding benzofused systems **6x** and **6y** in good yields (Scheme 16). Noteworthy is the special behavior of the dinitro compound **4c** that decomposed under these conditions. Unfortunately, the reaction failed for Passerini-adducts derived from an aromatic aldehyde. Indeed, compound **2f** treated under basic conditions gave the pyruvamide **7** through an oxidative process of the benzylic anion (Scheme 17).

**Scheme 18:**
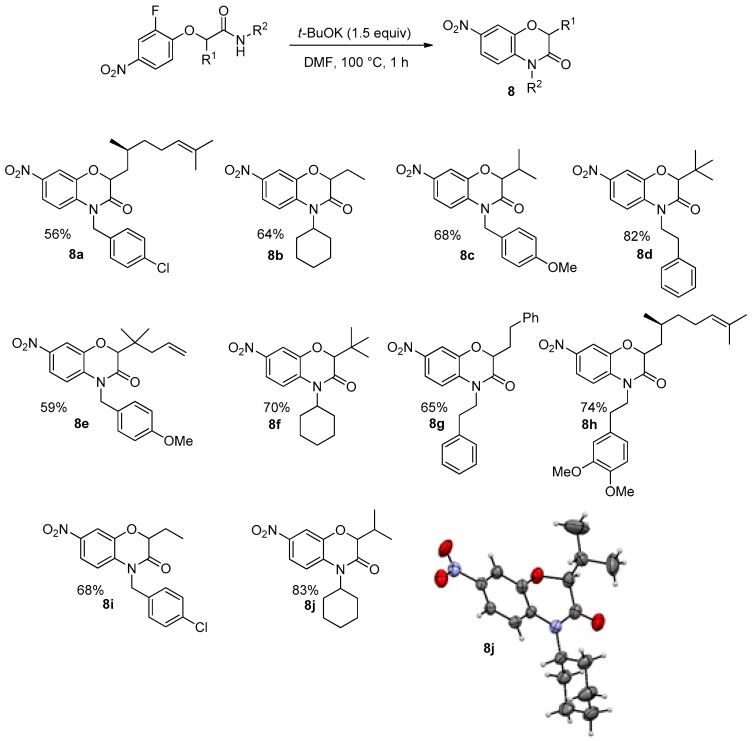
Smiles rearrangement of Passerini-Smiles adducts from 2-fluoro-4-nitrophenols.

A similar cascade towards substituted benzoxazinones could be done starting with 2-fluoro-4-nitrophenol as a partner of the Passerini-Smiles reaction. Under similar experimental conditions, the reaction proceeded smoothly with the release of a fluoride ion in place of the previous nitro group elimination (Scheme 18). Indeed, X-ray analyses of **8j** confirmed that a Smiles rearrangement occurred prior to cyclization.

Due to the simple experimental procedure settled for the three-component coupling, the whole sequence Passerini-double Smiles could be performed in the same pot. Indeed after 12 h of stirring at 55 °C, DMF was added to dilute the reaction mixture and the desired amount of potassium *tert*-butoxide was added at once. This one-pot sequential procedure affords a very convenient and straightforward access to benzoxazinones in moderate to high yields with three points of molecular diversity. In all cases, the products are isolated in similar or even better yields (Table 1).

## 4. Materials and Methods

### 4.1. General Procedure A for Passerini-Smiles Reaction with 2-Nitrophenol Derivatives

#### 4.1.1. Aldehydes

To 1.0 equiv. of phenol were added successively 1.0 equiv. of DABCO, 2.0 equiv. of aldehyde and 1.0 equiv. of isocyanide. The resulting mixture was stirred neat at 55 °C for 12 h. The crude product was purified by flash chromatography on silica gel.

#### 4.1.2. Ketones

To 1.0 equiv. of phenol were added successively 1.0 equiv. of ketone and 1.0 equiv. of isocyanide under an inert atmosphere. The resulting mixture was stirred neat at 55 °C for three days. The crude product was purified by flash chromatography on silica gel.

### 4.2. General Procedure B for Passerini-Smiles Reaction with 2-Fluoro-4-nitrophenol Derivatives

To 1.0 equiv. of phenol were added successively 1.0 equiv. of DABCO, 2.0 equiv. of aldehyde, and 1.0 equiv. of isocyanide. The resulting mixture was stirred neat at 80 °C for 12 h. The crude product was dissolved in dichloromethane. The organic layer was washed with 1 M NaOH, water, dried over MgSO_4_ and then concentrated. The crude product was purified by flash chromatography on silica gel.

### 4.3. General Procedure C for Reduction-Cyclization Sequence

The reduction of nitro compounds was carried out using a H-cube^®^ system (Thalesnano, Budapest, Hungary) with a 10% Pd/C as a catalyst. The Passerini-Smiles adduct (0.5 mmol) was dissolved in methanol (0.06 M) and was then passed through the H-cube^®^ system at 60 °C, 50 bars pressure with a H_2_ constant flow rate of 0.5 mL/min. The methanol was then removed under reduced pressure and the ^1^H-NMR analysis showed total conversion.

The crude product was dissolved in toluene (0.1 M) and TFA (2.5 mmol, 5 equiv.) was added. After 12 h of stirring at 85 °C, the reaction mixture was diluted with dichloromethane. The content was transferred to a separatory funnel and the organic phase was washed with a saturated aqueous solution of NaHCO_3_. The aqueous layer was extracted with CH_2_Cl_2_. The combined organic layers were washed with water, dried over MgSO_4_ and concentrated under reduced pressure. The crude product was purified by flash chromatography on silica gel.

### 4.4. General Procedure D for Smiles Cyclization

To a 0.2 M solution of Passerini-Smiles adduct in DMF were added 1.5 equiv. (aldehyde) or 2.0 equiv. (ketone) of potassium *tert*-butoxide. The resulting mixture was stirred for 1 h at 100 °C. The resulting mixture was diluted with 1 M HCl. The aqueous layer was extracted three times with CH_2_Cl_2_. Organic layers were combined, washed with water, dried over MgSO_4_, and concentrated. The crude product was purified by flash chromatography on silica gel.

### 4.5. General Procedure E for Passerini-Double-Smiles Reaction (One-Pot)

To 1.0 equiv. of phenol were added successively 1.0 equiv. of DABCO (only for aldehydes), 2.0 equiv. of aldehyde (1.0 equiv. of ketone), and 1.0 equiv. of isocyanide under an inert atmosphere. The resulting mixture was stirred neat at 55 °C (or 80 °C for 2-fluoro-4-nitrophenol derivatives) for 12 h for aldehyde (three days for ketone). Then, DMF (0.2 M) and 1.5 equiv. (aldehyde) or 2.0 equiv. (ketone) of potassium *tert*-butoxide were added. The resulting mixture was stirred for 1 h at 100 °C. The resulting mixture was diluted with CH_2_Cl_2_ then washed with H_2_O and 1 M HCl. The aqueous layer was extracted three times with CH_2_Cl_2_. Organic layers were combined, washed with water, dried over MgSO_4_, and concentrated. The crude product was purified by flash chromatography on silica gel.

## 5. Conclusions

In conclusion, we have settled new conditions for efficient Passerini-Smiles reactions. The dramatic improvement of this reaction is strongly associated to the use of a tertiary nucleophilic amine as additive together with solvent free conditions. This new procedure has allowed us to propose various syntheses of functionalized 1,4-benzoxazin-3-ones through post-condensation transformations of the resulting adducts. The most interesting transformations are based on a Smiles cyclization cascade taking full advantage of the phenoxy group introduced during the first Passerini-Smiles process. The whole sequence could be performed according to a very convenient one-pot procedure affording one of the most straightforward access to 1,4-benzoxazin-3-ones, heterocycles of interest for both agrochemical [16] and pharmaceutical [17,18,19] applications.

## Figures and Tables

**Table:** 

Conditions ^a^	Yields (%)
Cs_2_CO_3_ (1 equiv.), DMF, 100 °C, 3 h	0
DBU (1.5 equiv.), DMF, 100 °C, 3 h	0
NaH (2 equiv.), DMF, 100 °C, 3 h	54
KH (1.5 equiv.), DMF, 100 °C, 3 h	69
t-BuOK (1.5 equiv.), DMF, 100 °C, 2 h	82 (89) ^b^
t-BuOK (1.5 equiv.), MeCN, 75 °C, 3 h	28
t-BuOK (1.5 equiv.), THF, 65 °C, 2 h	36

^a^ All reactions were done at [**2b**] = 0.4 M. ^b^ at [**2b**] = 0.2 M, for 1 h.

**Table 1:**
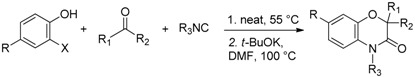
Comparison of sequential and one-pot benzoxazinone synthesis.

Entry	Phenol	Product	Yield over 2 Steps (%)	Yield One-Pot (%) ^1^
**1**	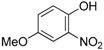	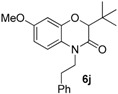	75	63 ^2^
**2**	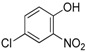	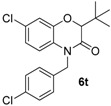	82	89 ^2^
**3**	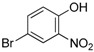	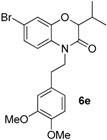	67	65 ^2^
**4**	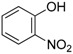	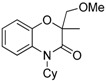	49	48 ^2^
**5**	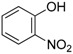	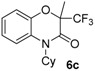	63	71 ^3^
**6**	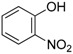	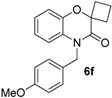	54	61 ^3^
**7**	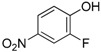	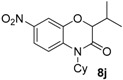	66	68 ^2^
**8**	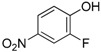	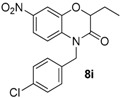	50	66 ^2^
**9**	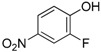	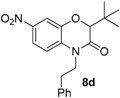	39	53 ^2^

^1^ Isolated Yields. ^2^ The first step was performed using 2 equiv. of aldehyde and adding 1 equiv. of DABCO. ^3^ The first step was performed using a stoichiometric ratio of the three starting materials.

## Supplementary Materials

Supplementary materials can be accessed at: http://www.mdpi.com/1420-3049/21/9/1257/s1.
