# Supplementary material for: Optimized Conditions for Passerini-Smiles Reactions and Applications to Benzoxazinone Syntheses
Source: Molecules. 2016 Sep 21;21(9):1257. doi: 10.3390/molecules21091257 (PMC6273966; doi:10.3390/molecules21091257)
Supplement: Supplementary file 1 [file molecules-21-01257-s001.pdf]

Supplementary Materials: Optimized Conditions for Passerini-Smiles Reactions and Applications to Benzoxazinone Syntheses

Elodie Martinand-Lurin, Aurélie Dos Santos, Emmanuelle Robineau, Pascal Retailleau, Philippe Dauban, Laurence Grimaud and Laurent El Kaïm

Table of Contents

1. General Notes S2
2. Products S2
3. ^{1}H- and ^{13}C-NMR spectra S25
4. X-ray Crystallography S134
5. Kinetic studies S138

1. General Notes

Melting points, measured in capillary tubes on a Büchi B-540 apparatus, are uncorrected. IR spectra were recorded on a Perkin Elmer Spectrum 100 FT-IR spectrometer (Villebon-sur-Yvette, France).

Proton (^{1}H) and carbon (^{13}C) NMR spectra were recorded on Bruker spectrometers (Wissembourg, France): Avance 300 MHz (QNP-^{13}C, ^{31}P, ^{19}F-probe or Dual ^{13}C probe) and Avance 500 MHz (BB0-ATM probe or BBI-ATM probe). Chemical shifts (δ) are reported in parts per million (ppm) with reference to $CDCl_3$ (^{1}H: 7.26; ^{13}C: 77.13) or CD_2Cl_2 (^{1}H: 5.32; ^{13}C: 53.80). The following abbreviations are used for the proton spectra multiplicities: s: singlet, d: doublet, t: triplet, q: quartet, quint.: quintuplet, sept.: septuplet, m: multiplet, br: broad. Coupling constants (*J*) are reported in Hertz (Hz). The multiplicity of carbons was given using 2D spectra (HMQC and HMBC). Some quaternary carbons were determined using HMBC couplings.

UPLC-MS analyses were run using a Acquity Waters UPLC equipped with a Waters LCT Premier XE (ESI ionization) (Guyancourt, France) and a Waters Acquity PDA detector, using a column BEH C_{18} 1.7 µm, 2.1 mm × 50 mm. Gradients were run using water and acetonitrile (1:1) with 0.1% of acetic acid. Temperature: 40 °C. UV detection from 210 to 410 nm. ESI$^+$ detection in the 80–1500 *m*/*z* range.

Thin-layer chromatography was performed on silica gel 60 F_{254} on aluminum plates (Merck, Fontenay-sous-Bois, France) and visualized under a UVP Mineralight UVLS-28 lamp (254 nm). Flash chromatography was performed on silica gel 60 (230–400 mesh).

All reagents were obtained from commercial suppliers (Sigma-Aldrich (Saint Quentin Fallavier, France) and Acros Organics (Geel, Belgium)) and were used as received.

2. Products

2.1. General Procedure E for Passerini-Double-Smiles Reaction (One-pot)

To 1.0 equiv of phenol were added successively 1.0 equiv of DABCO (only for aldehydes), 2.0 equiv of aldehyde (1.0 equiv of ketone) and 1.0 equiv of isocyanide under inert atmosphere. The resulting mixture was stirred neat at 55 °C (or 80 °C for 2-fluoro-4-nitrophenol derivatives) during 12 h for aldehyde (3 days for ketone). Then, DMF (0.2 M) and 1.5 equiv (aldehyde) or 2.0 equiv (ketone) of potassium *tert*-butoxide were added. The resulting mixture was stirred for 1 h at 100 °C. The resulting mixture was diluted CH_2Cl_2 then washed with H_2O and HCl 1 M. The aqueous layer was extracted three times with CH_2Cl_2. Organic layers were combined, washed with water, dried over $MgSO_4$ and concentrated. The crude product was purified by flash chromatography on silica gel.

1-(4-Chloro-2-nitrophenoxy)-N-cyclohexylcyclobutanecarboxamide (**1b**). Compound **1b** was prepared according to the general procedure A. Purification on a column of silica gel with a gradient of ethyl acetate in petroleum ether (from 100/0 to 90/10) as eluent gave the desired product (350 mg, 50%) as a yellow solid. R_f = 0.8 (petroleum ether/ethyl acetate 70/30); mp 127–128 °C; ^{1}H-NMR (300 MHz, $CDCl_3$, 25 °C): δ = 7.78 (d, 1H, *J* = 2.7 Hz), 7.37 (dd, 1H, *J* = 9.0, 2.7 Hz), 6.67 (d, 1H, *J* = 9.0 Hz), 6.16 (br d, 1H, *J* = 8.4 Hz), 3.81–3.62 (m, 1H), 2.83–2.71 (m, 2H), 2.48–2.33 (m, 2H), 2.08–1.86 (m, 2H), 1.76–1.64 (m, 2H), 1.64–1.47 (m, 3H), 1.38–1.19 (m, 2H), 1.19–0.90 (m, 3H); ^{13}C-NMR (75 MHz, $CDCl_3$, 25 °C): δ = 170.2, 147.3, 140.8, 133.7, 126.6, 125.5, 117.8, 83.3, 48.2, 32.7, 32.0, 25.4, 24.6, 13.8; IR (Neat): ν = 3264, 2938, 2852, 1640, 1606, 1533, 1481, 1359, 1281, 1255, 1226, 1160, 1129, 1077 cm^{-1}; HRMS (ESI$^+$; MeCN/CH_2Cl_2): *m*/*z* calculated for $C_{17}H_{21}ClN_2O_4$ 353.1246, found 353.1244.

N-(4-Methoxybenzyl)-1-(2-nitrophenoxy)cyclobutanecarboxamide (**1c**). Compound **1c** was prepared according to the general procedure A. Purification on a column of silica gel with a gradient of ethyl acetate in petroleum ether (from 90/10 to 80/20) as eluent gave the desired product (223 mg, 63%) as a yellow oil. R_f = 0.4 (petroleum ether/ethyl acetate 70/30); ^{1}H-NMR (500 MHz, $CDCl_3$, 25 °C): δ = 7.75 (dd, 1H, *J* = 7.9, 1.7 Hz), 7.39 (td, 1H, *J* = 7.9, 1.7 Hz), 7.05 (td, 1H, *J* = 7.9, 1.3 Hz), 6.92 (d, 2H, *J* = 8.6 Hz), 6.75-6.68 (m, 3H), 6.64 (br t, 1H, *J* = 5.9 Hz), 4.33 (d, 1H, *J* = 5.9 Hz), 3.76 (s, 3H), 2.86–2.77 (m, 2H),

2.51–2.40 (m, 2H), 2.10–1.91 (m, 2H); ^{13}C-NMR (75 MHz, $CDCl_3$, 25 °C): δ = 171.5, 158.9, 148.5, 140.6, 133.9, 130.0, 128.7, 125.6, 121.5, 116.7, 114.0, 82.9, 55.3, 42.8, 32.1, 13.8; IR (Neat): ν = 3328, 2947, 2836, 1664, 1605, 1584, 1510, 1479, 1350, 1303, 1275, 1243, 1174, 1154, 1128, 1078, 1032 cm^{-1}; HRMS (ESI$^+$; MeCN/CH_2Cl_2): *m*/*z* calculated for $C_{19}H_{20}N_2O_5$ 357.1450, found 357.1447.

1-(4-Bromo-2-nitrophenoxy)-N-(3,4-dimethoxyphenethyl)cyclobutanecarboxamide (**1d**). Compound **1d** was prepared according to the general procedure A. Purification on a column of silica gel with a gradient of ethyl acetate in petroleum ether (from 90/10 to 70/30) as eluent gave the desired product (277 mg, 58%) as a yellow oil. R_f = 0.3 (petroleum ether/ethyl acetate 70/30); ^{1}H-NMR (500 MHz, $CDCl_3$, 25 °C): δ = 7.89 (d, 1H, *J* = 2.4 Hz), 7.44 (dd, 1H, *J* = 8.9, 2.4 Hz), 6.66 (d, 1H, *J* = 8.9 Hz), 6.59 (d, 1H, *J* = 1.8 Hz), 6.50–6.42 (d, 2H, *J* = 8.9 Hz), 6.23 (br t, 1H, *J* = 5.6 Hz), 3.85 (s, 3H), 3.82 (s, 3H), 3.50 (q, 2H, *J* = 6.5 Hz), 2.77–2.69 (m, 2H), 2.66 (t, 2H, *J* = 6.9 Hz), 2.41–2.32 (m, 2H), 2.05–1.88 (m, 2H); ^{13}C-NMR (75 MHz, $CDCl_3$, 25 °C): δ = 171.3, 149.0, 147.7, 147.5, 140.7, 136.5, 130.4, 128.4, 120.4, 117.9, 113.1, 111.4, 111.1, 83.1, 55.9, 55.8, 40.3, 34.8, 31.9, 13.8; IR (Neat): ν = 3358, 2940, 1664, 1602, 1513, 1473, 1417, 1346, 1259, 1235, 1155, 1127, 1103, 1077, 1026. HRMS (ESI$^+$; MeCN/CH_2Cl_2): *m*/*z* calculated for $C_{21}H_{23}BrN_2O_6$ 479.0818, found 479.0822.

N-Cyclohexyl-3,3,3-trifluoro-2-methyl-2-(2-nitrophenoxy)propanamide (**1e**). Compound **1e** was prepared according to the general procedure A (add 2 equiv. of ketone instead of one). Purification on a column of silica gel with a gradient of ethyl acetate in petroleum ether (from 95/5 to 80/20) as eluent gave the desired product (565 mg, 78%) as an off-white solid. R_f = 0.7 (petroleum ether/ethyl acetate 70/30); mp 91–92 °C; ^{1}H-NMR (300 MHz, $CDCl_3$, 25 °C): δ = 7.81 (dd, 1H, *J* = 8.1, 1.7 Hz), 7.54 (ddd, 1H, *J* = 8.3, 7.6, 1.7 Hz), 7.28 (ddd, 1H, *J* = 7.6, 8.1, 1.1 Hz), 7.22 (dd, 1H, *J* = 8.3, 1.1 Hz), 6.76 (br d, 1H, *J* = 7.7 Hz), 3.91–3.75 (m, 1H), 1.97–1.83 (m, 2H), 1.77–1.55 (m, 3H), 1.66 (s, 3H), 1.45–1.28 (m, 2H), 1.28–1.09 (m, 3H); ^{13}C-NMR (75 MHz, $CDCl_3$, 25 °C): δ = 164.0, 146.1, 144.0, 133.6, 125.5, 125.0, 123.3 (q, $J_{C\text{-}F}$ = 287.6 Hz), 122.9, 84.2 (q, $J_{C\text{-}F}$ = 28.8 Hz), 49.1, 32.6, 32.5, 25.5, 24.8, 15.4; ^{19}F-NMR (282 MHz, $CDCl_3$, 25 °C): δ = –78.0; IR (Neat): ν = 3307, 2940, 2859, 1664, 1605, 1536, 1481, 1449, 1379, 1360, 1295, 1267, 1240, 1198, 1178, 1134, 1106 cm^{-1}; HRMS (ESI$^+$; MeCN/CH_2Cl_2): *m*/*z* calculated for $C_{16}H_{19}F_3N_2O_4$ 361.1375, found 361.1365.

N-Cyclohexyl-3-methoxy-2-methyl-2-(2-nitrophenoxy)propanamide (**1f**). Compound **1f** was prepared according to the general procedure A. Purification on a column of silica gel with a gradient of ethyl acetate in heptane (from 90/10 to 70/30) as eluent gave the desired product (438 mg, 65%) as a yellow oil. R_f = 0.2 (petroleum ether/ethyl acetate 70/30); ^{1}H-NMR (500 MHz, $CDCl_3$, 25 °C): δ = 7.84 (dd, 1H, *J* = 8.1, 1.8 Hz), 7.51 (ddd, 1H, *J* = 8.5, 7.4, 1.8 Hz), 7.31 (dd, 1H, *J* = 8.3, 1.3 Hz), 7.32–7.24 (m, 1H), 7.18 (ddd, 1H, *J* = 8.1, 7.4, 1.3 Hz), 3.83–3.71 (m, 1H), 3,71 (d, 1H, *J* = 10.8 Hz), 3.50 (d, 1H, *J* = 10.8 Hz), 3.22 (s, 3H), 1.98–1.87 (m, 2H), 1.78–1.66 (m, 2H), 1.64–1.55 (m, 1H), 1.47 (s, 3H), 1.42-1.10 (m, 5H); ^{13}C-NMR (125 MHz, $CDCl_3$, 25 °C): δ = 170.1, 148.0, 144.0, 133.6, 125.7, 123.6, 123.5, 86.8, 75.0, 59.2, 48.4, 32.7, 25.5, 24.8, 19.6; IR (Neat): ν = 3399, 2930, 2854, 1671, 1602, 1582, 1521, 1478, 1450, 1353, 1239, 1199, 1107 cm^{-1}; HRMS (ESI$^+$; MeCN/CH_2Cl_2): *m*/*z* calculated for $C_{17}H_{24}N_2O_5$ 337.1763, found 337.1752.

2-(4-Chloro-2-nitrophenoxy)-3-methoxy-2-methyl-N-phenethylpropanamide (**1g**). Compound **1g** was prepared according to the general procedure A. Purification on a column of silica gel with a gradient of ethyl acetate in petroleum ether (from 90/10 to 80/20) as eluent gave the desired product (466 mg, 59%) as an orange oil. R_f = 0.2 (petroleum ether/ethyl acetate 80/20); ^{1}H-NMR (500 MHz, $CDCl_3$, 25 °C): δ = 7.83 (d, 1H, *J* = 2.7 Hz), 7.43 (dd, 1H, *J* = 8.9, 2.7 Hz), 7.33–7.20 (m, 7H), 3.69 (d, 1H, *J* = 10.8 Hz), 3.67–3.54 (m, 2H), 3.49 (d, 1H, *J* = 10.8 Hz), 3.24 (s, 3H), 2.91 (dt, 1H, *J* = 15.5, 7.2 Hz), 2.88 (dt, 1H, *J* = 15.5, 7.2 Hz), 1.45 (s, 3H); ^{13}C-NMR (75 MHz, $CDCl_3$, 25 °C): δ = 171.0, 146.6, 144.2, 138.8, 133.6, 128.9, 128.6, 126.5, 125.6, 124.8, 87.2, 75.1, 59.3, 41.0, 35.6, 19.7; IR (Neat): ν = 3412, 2930, 1670, 1603, 1527, 1497, 1475, 1454, 1353, 1240, 1198, 1150, 1104 cm^{-1}; HRMS (ESI$^+$; MeCN/CH_2Cl_2): *m*/*z* calculated for $C_{19}H_{21}ClN_2O_5$ 393.1217, found 393.1206.

N-Cyclohexyl-2-(2-nitrophenoxy)butanamide (**2a**). Compound **2a** was prepared according to the general procedure A. Purification on a column of silica gel with a gradient of ethyl acetate in petroleum ether (from 90/10 to 60/40) as eluent gave the desired product (269 mg, 88%) as an off-white solid. R_f = 0.2 (petroleum ether/ethyl acetate 70/30); mp 107–108 °C; ^{1}H-NMR (500 MHz, $CDCl_3$, 25 °C): δ = 7.91 (dd, 1H, J = 8.3, 1.7 Hz), 7.54 (ddd, 1H, J = 7.5, 8.4, 1.7 Hz), 7.15-7.02 (m, 3H), 4.84 (t, 1H, J = 5.0 Hz), 3.77 (m, 1H), 2.11-1.99 (m, 2H), 1.98-1.87 (m, 1H), 1.79-1.50 (m, 4H), 1.44-1.04 (m, 5H), 0.98 (t, 3H, J = 7.5 Hz); ^{13}C-NMR (75 MHz, $CDCl_3$, 25 °C): δ = 168.8, 150.8, 139.9, 134.9, 126.5, 121.5, 115.1, 80.3, 48.1, 33.1, 32.8, 25.6, 25.4, 24.8, 8.7; IR (Neat): ν = 3259, 2934, 2854, 1652, 1608, 1583, 1557, 1520, 1484, 1445, 1349, 1276, 1245, 1233, 1165, 1153, 1089, 1047, 1026 cm^{-1}; HRMS (ESI$^+$; MeCN/CH_2Cl_2): m/z calculated for $C_{16}H_{22}N_2O_4$ 307.1658, found 307.1652.

N-Cyclohexyl-3-methyl-2-(2-nitrophenoxy)butanamide (**2b**). Compound **2b** was prepared according to the general procedure A. Purification on a column of silica gel with a gradient of ethyl acetate in petroleum ether (from 90/10 to 80/20) as eluent gave the desired product (291 mg, 91%) as a yellow solid. R_f = 0.4 (petroleum ether/ethyl acetate 70/30); mp 121–122 °C; ^{1}H-NMR (300 MHz, $CDCl_3$, 25 °C): δ = 7.86 (dd, 1H, J = 8.2, 1.8 Hz), 7.52 (ddd, 1H, J = 8.6, 7.5, 1.8 Hz), 7.11–7.04 (m, 2H), 6.93 (br d, 1H, J = 8.2 Hz), 4.68 (d, 1H, J = 3.8 Hz), 3.83–3.68 (m, 1H), 2.36 (sept d, 1H, J = 6.9, 3.8 Hz), 1.98–1.87 (m, 1H), 1.76–1.50 (m, 5H), 1.43–0.90 (m, 4H), 1.06 (d, 6H, J = 6.9 Hz); ^{13}C-NMR (75 MHz, $CDCl_3$, 25 °C): δ = 168.4, 151.0, 140.0, 134.6, 126.0, 121.4, 115.0, 84.0, 47.9, 33.0, 32.6, 31.5, 25.4, 24.7, 24.6, 18.8, 17.0; IR (Neat): ν = 3261, 3080, 2937, 2856, 1649, 1607, 1584, 1557, 1521, 1484, 1449, 1388, 1351, 1309, 1276, 1259, 1248, 1233, 1165, 1154, 1091, 1048, 1036 cm^{-1}; HRMS (ESI$^+$; MeCN/CH_2Cl_2): m/z calculated for $C_{17}H_{24}N_2O_4$ 321.1814, found 321.1812.

N-Cyclohexyl-3,3-dimethyl-2-(2-nitrophenoxy)butanamide (**2c**). Compound **2c** was prepared according to the general procedure A. Purification on a column of silica gel with a gradient of ethyl acetate in petroleum ether (from 90/10 to 80/20) as eluent gave the desired product (283 mg, 85%) as a white solid. R_f = 0.2 (petroleum ether/ethyl acetate 80/20); mp 126–127 °C; ^{1}H-NMR (500 MHz, $CDCl_3$, 25 °C): δ = 7.83 (dd, 1H, J = 8.1, 1.6 Hz), 7.50 (ddd, 1H, J = 8.5, 7.5, 1.6 Hz), 7.10 (d, 1H, J = 8.5 Hz), 7.07 (ddd, 1H, J = 8.1, 7.5, 1.0 Hz), 6.64 (br d, 1H, J = 8.5 Hz), 4.46 (s, 1H), 3.73-3.67 (m, 1H), 1.93-1.86 (m, 1H), 1.72-1.63 (m, 1H), 1.54–1.45 (m, 3H), 1.39–1.07 (m, 4H), 1.11 (s, 9H), 0.94–0.84 (m, 1H); ^{13}C-NMR (125 MHz, $CDCl_3$, 25 °C): δ = 167.4, 150.8, 140.1, 134.5, 125.8, 121.4, 114.9, 87.0, 47.8, 34.7, 33.1, 32.5, 26.3, 25.4, 24.7, 24.6; IR (Neat): ν = 3259, 2926, 2853, 1646, 1607, 1584, 1568, 1522, 1479, 1449, 1365, 1344, 1309, 1279, 1248, 1197, 1167, 1151, 1099, 1060, 1019 cm^{-1}; HRMS (ESI$^+$; MeCN/CH_2Cl_2): m/z calculated for $C_{18}H_{26}N_2O_4$ 335.1971, found 335.197.

N-Cyclohexyl-2-(2-nitrophenoxy)-4-phenylbutanamide (**2d**). Compound **2d** was prepared according to the general procedure A. Purification on a column of silica gel with a gradient of ethyl acetate in petroleum ether (from 95/5 to 90/10) as eluent gave the desired product (647 mg, 85%) as a yellow solid. R_f = 0.4 (petroleum ether/ethyl acetate 70/30); mp 97–98 °C; ^{1}H-NMR (500 MHz, $CDCl_3$, 25 °C): δ = 7.91 (dd, 1H, J = 8.1, 1.7 Hz), 7.50 (ddd, 1H, J = 8.6, 7.5, 1.7 Hz), 7.28–7.22 (m, 2H), 7.20–7.14 (m, 3H), 7.13–7.05 (m, 2H), 6.94 (d, 1H, J = 8.6 Hz), 4.84 (t, 1H, J = 5.4 Hz), 3.83–3.72 (m, 1H), 2.86–2.71 (m, 2H), 2.32 (dt, 2H, J = 5.4, 7.9 Hz), 1.97–1.90 (m, 1H), 1.78–1.68 (m, 2H), 1.67–1.58 (m, 2H), 1.43–1.05 (m, 5H); ^{13}C-NMR (125 MHz, $CDCl_3$, 25 °C): δ = 168.7, 150.4, 140.5, 139.8, 134.7, 128.6, 128.5, 126.2, 121.5, 114.9, 78.6, 48.0, 33.9, 32.9, 32.7, 30.6, 25.4, 24.7, 24.6; IR (Neat): ν = 3263, 3028, 2930, 2853, 1651, 1607, 1582, 1518, 1496, 1482, 1449, 1345, 1307, 1271, 1246, 1165, 1152, 1084, 1047, 1028 cm^{-1}; HRMS (ESI$^+$; MeCN/CH_2Cl_2): m/z calculated for $C_{22}H_{26}N_2O_4$ 383.1971, found 383.1972.

N-Cyclohexyl-2-(3-fluorophenyl)-2-(2-nitrophenoxy)acetamide (**2e**). Compound **2e** was prepared according to the general procedure A. Purification on a column of silica gel with a gradient of ethyl acetate in petroleum ether (from 95/5 to 80/20) as eluent gave the desired product (186 mg, 50%) as an off-white solid. R_f = 0.4 (petroleum ether/ethyl acetate 70/30); mp 145–146 °C; ^{1}H-NMR (500 MHz, $CDCl_3$, 25 °C): δ = 8.04 (dd, 1H, J = 8.4, 1.5 Hz), 7.47 (ddd, 1H, J = 8.4, 7.3, 1.5 Hz), 7.42–7.30 (m, 3H), 7.23 (dt, 1H, J = 9.2, 2.2 Hz), 7.10 (br t, 1H, J = 7.8 Hz), 7.07–7.00 (m, 1H), 6.91 (d, 1H, J = 8.4 Hz), 5.63

(s, 1H), 3.81–3.71 (m, 1H), 1.93–1.83 (m, 2H), 1.77–1.69 (m, 2H), 1.64–1.58 (m, 1H), 1.41–1.19 (m, 5H); ^{13}C-NMR (125 MHz, $CDCl_3$, 25 °C): δ = 166.7, 163.1 (d, J_{C-F} = 246.1 Hz), 150.3, 139.4, 137.9 (d, J_{C-F} = 7.6 Hz), 135.2, 130.7 (d, J_{C-F} = 8.3 Hz), 126.8, 122.3 (d, J_{C-F} = 3.0 Hz), 122.0, 116.1 (d, J_{C-F} = 21.1 Hz), 115.9, 113.7 (d, J_{C-F} = 23.0 Hz), 80.3, 48.3, 32.79, 32.77, 25.5, 24.71, 24.69; ; ^{13}C-NMR (125 MHz, $CDCl_3$, 25 °C): δ = –111.6; IR (Neat): ν = 3259, 3077, 2921, 2853, 1655, 1610, 1585, 1559, 1525, 1487, 1445, 1372, 1342, 1281, 1266, 1246, 1227, 1171, 1151, 1139, 1103, 1054 cm^{-1}; HRMS (ESI$^+$; MeCN/CH_2Cl_2): *m*/*z* calculated for $C_{20}H_{22}FN_2O_4$ 373.1564, found 373.1561.

2-(4-Chlorophenyl)-N-cyclohexyl-2-(2-nitrophenoxy)acetamide (**2f**). Compound **2f** was prepared according to the general procedure A. Purification on a column of silica gel with a gradient of ethyl acetate in petroleum ether (from 95/5 to 70/30) as eluent gave the desired product (452 mg, 58%) as a yellow solid. R_f = 0.5 (petroleum ether/ethyl acetate 70/30); mp 147–148 °C; ^{1}H-NMR (300 MHz, $CDCl_3$, 25 °C): δ = 8.03 (dd, 1H, *J* = 8.2, 1.8 Hz), 7.50–7.31 (m, 6H), 7.09 (ddd, 1H, *J* = 8.2, 7.4, 1.1 Hz), 6.89 (dd, 1H, *J* = 8.5, 1.1 Hz), 5.62 (s, 1H), 3.75 (m, 1H), 1.95-1.79 (m, 2H), 1.77–1.50 (m, 3H), 1.44–1.17 (m, 5H); ^{13}C-NMR (75 MHz, $CDCl_3$, 25 °C): δ = 166.8, 150.2, 139.3, 135.2, 135.1, 134.0, 129.3, 127.9, 126.8, 121.9, 115.9, 80.2, 48.3, 32.8, 25.5, 24.7; IR (Neat): ν = 3265, 3081, 2918, 2852, 1653, 1609, 1560, 1524, 1490, 1357, 1289, 1244, 1090 cm^{-1}; HRMS (ESI$^+$; MeCN/CH_2Cl_2): *m*/*z* calculated for $C_{20}H_{22}ClN_2O_4$ 389.1268, found 389.1285.

N-(4-Methoxybenzyl)-3,3-dimethyl-2-(4-methyl-2-nitrophenoxy)hex-5-enamide (**2g**). Compound **2g** was prepared according to the general procedure A. Purification on a column of silica gel with a gradient of ethyl acetate in petroleum ether (from 90/10 to 80/20) as eluent gave the desired product (344 mg, 83%) as a yellow solid. R_f = 0.3 (petroleum ether/ethyl acetate 80/20); mp 64–65 °C; ^{1}H-NMR (500 MHz, $CDCl_3$, 25 °C): δ = 7.56 (d, 1H, *J* = 2.3 Hz), 7.26 (dd, 1H, *J* = 8.4, 2.3 Hz), 7.07 (br t, 1H, *J* = 5.8 Hz), 6.95 (d, 2H, *J* = 8.5 Hz), 6.92 (d, 1H, *J* = 8.4 Hz), 6.70 (d, 2H, *J* = 8.5 Hz), 5.87–5.76 (m, 1H), 5.04 (dd, 1H, *J* = 10.1, 1.4 Hz), 4.96 (dd, 1H, *J* = 17.0, 1.4 Hz), 4.55 (s, 1H), 4.35 (dd, 1H, *J* = 14.5, 6.0 Hz), 4.28 (dd, 1H, *J* = 14.5, 6.0 Hz), 3.75 (s, 3H), 2.33 (s, 3H), 2.26 (dd, 1H, *J* = 13.7, 7.8 Hz), 2.11 (dd, 1H, *J* = 13.7, 6.8 Hz), 1.08 (s, 3H), 1.06 (s, 3H); ^{13}C-NMR (125 MHz, $CDCl_3$, 25 °C): δ = 168.7, 158.9, 148.5, 139.5, 135.1, 134.1, 131.6, 130.1, 128.9, 126.0, 118.7, 114.7, 114.0, 85.1, 55.3, 43.7, 42.6, 37.8, 23.5, 23.4, 20.3; IR (Neat): ν = 3381, 2967, 2932, 2837, 1666, 1613, 1575, 1527, 1512, 1465, 1440, 1390, 1349, 1320, 1301, 1277, 1245, 1174, 1158, 1111, 1086, 1034, 1001 cm^{-1}; HRMS (ESI$^+$; MeCN/CH_2Cl_2): *m*/*z* calculated for $C_{23}H_{28}N_2O_5$ 413.2076, found 413.2081.

N-(4-Chlorobenzyl)-4-methyl-2-(4-methyl-2-nitrophenoxy)pentanamide (**2h**). Compound **2h** was prepared according to the general procedure A. Purification on a column of silica gel with a gradient of ethyl acetate in petroleum ether (from 99/1 to 95/5) as eluent gave the desired product (291 mg, 72%) as a yellow solid. R_f = 0.2 (petroleum ether/ethyl acetate 80/20); mp 102–103 °C; ^{1}H-NMR (500 MHz, $CDCl_3$, 25 °C): δ = 7.60 (d, 1H, *J* = 2.2 Hz), 7.45 (br t, 1H, *J* = 6.7 Hz), 7.31 (dd, 1H, *J* = 8.6, 2.2 Hz), 7.19 (d, 2H, *J* = 8.4 Hz), 7.05 (d, 2H, *J* = 8.4 Hz), 6.96 (d, 1H, *J* = 8.6 Hz), 4.87 (dd, 1H, *J* = 8.1, 4.0 Hz), 4.43 (dd, 1H, *J* = 14.9, 6.4 Hz), 4.34 (dd, 1H, *J* = 14.9, 6.4 Hz), 2.35 (s, 3H), 1.94–1.77 (m, 3H), 0.96 (d, 3H, *J* = 6.2 Hz), 0.93 (d, 3H, *J* = 6.2 Hz); ^{13}C-NMR (125 MHz, $CDCl_3$, 25 °C): δ = 171.0, 148.3, 139.7, 136.5, 135.2, 133.3, 131.9, 129.0, 128.8, 126.2, 114.7, 78.7, 42.5, 41.7, 24.7, 23.2, 22.1, 20.3; IR (Neat): ν = 3262, 3062, 2956, 2870, 1651, 1566, 1528, 1490, 1467, 1350, 1282, 1257, 1163, 1088, 1014 cm^{-1}; HRMS (ESI$^+$; MeCN/CH_2Cl_2): *m*/*z* calculated for $C_{20}H_{23}ClN_2O_4$ 391.1425, found 391.1410.

N-Cyclohexyl-2-(4-methoxyphenyl)-2-(4-methyl-2-nitrophenoxy)acetamide (**2i**). Compound **2i** was prepared according to the general procedure A. Purification on a column of silica gel with a gradient of ethyl acetate in petroleum ether (from 90/10 to 80/20) as eluent gave the desired product (317 mg, 40%) as an off-white solid. R_f = 0.3 (petroleum ether/ethyl acetate 60/40); mp 133–134 °C; ^{1}H-NMR (500 MHz, $CDCl_3$, 25 °C): δ = 7.80 (d, 1H, *J* = 2.1 Hz), 7.44–7.35 (m, 1H), 7.40 (d, 2H, *J* = 8.8 Hz), 7.23 (dd, 1H, *J* = 8.5, 2.1 Hz), 6.90–6.86 (m, 2H), 6.84 (d, 1H, *J* = 8.5 Hz), 5.56 (s, 1H), 3.81–3.73 (m, 1H), 3.78 (s, 3H), 2.32 (s, 3H), 1.93–1.84 (m, 2H), 1.76–1.69 (m, 2H), 1.63–1.58 (m, 1H), 1.40–1.22 (m, 5H); ^{13}C-NMR (125 MHz, $CDCl_3$, 25 °C): δ = 167.7, 160.1, 148.4, 139.0, 135.7, 131.6, 128.0, 127.7, 126.6, 116.1,

114.4, 80.6, 55.4, 48.1, 32.8, 25.6, 24.7, 24.6, 20.3; IR (Neat): ν = 3258, 3080, 2921, 2853, 1651, 1625, 1614, 1531, 1512, 1463, 1445, 1346, 1293, 1266, 1244, 1196, 1175, 1152, 1094, 1054, 1030 cm^{-1}; HRMS (ESI^+; $MeCN/CH_2Cl_2$): *m*/*z* calculated for $C_{22}H_{27}N_2O_5$ 399.1920, found 399.1913.

2-(4-Methoxy-2-nitrophenoxy)-3,3-dimethyl-N-phenethylbutanamide (**2j**). Compound **2j** was prepared according to the general procedure A. Purification on a column of silica gel with a gradient of ethyl acetate in petroleum ether (from 90/10 to 80/20) as eluent gave the desired product (301 mg, 78%) as a yellow solid. R_f = 0.4 (petroleum ether/ethyl acetate 70/30); mp 88–89 °C; ^{1}H-NMR (500 MHz, $CDCl_3$, 25 °C): δ = 7.32 (d, 1H, *J* = 3.1 Hz), 7.20–7.10 (m, 3H), 7.05–6.99 (m, 3H), 6.93 (d, 1H, *J* = 9.3 Hz), 6.75 (br t, 1H, *J* = 5.7 Hz), 4.34 (s, 1H), 3.82 (s, 3H), 3.57 (dq, 1H, *J* = 13.5, 6.6 Hz), 3.47 (ddt, 1H, *J* = 13.6, 5.4, 7.2 Hz), 2.73 (dt, 1H, *J* = 16.6, 7.1 Hz), 2.70 (dt, 1H, *J* = 16.6, 7.1 Hz), 1.04 (s, 9H); ^{13}C-NMR (75 MHz, $CDCl_3$, 25 °C): δ = 168.9, 153.4, 145.0, 139.5, 138.3, 128.5, 126.5, 121.0, 115.6, 110.5, 87.2, 56.2, 40.0, 35.4, 34.9, 26.3; IR (Neat): ν = 3026, 2959, 1660, 1520, 1492, 1442, 1346, 1289, 1277, 1256, 1219, 1164, 1092, 1082, 1051, 1035, 1004 cm^{-1}; HRMS (ESI^+; $MeCN/CH_2Cl_2$): *m*/*z* calculated for $C_{21}H_{26}N_2O_5$ 387.1920, found 387.1929.

2-(2-Allyl-6-nitrophenoxy)-N-cyclohexyl-3-methylbutanamide (**2k**). Compound **2k** was prepared according to the general procedure A. Purification on a column of silica gel with a gradient of ethyl acetate in petroleum ether (from 100/0 to 98/2) as eluent gave the desired product (213 mg, 59%) as a white solid. R_f = 0.5 (petroleum ether/ethyl acetate 90/10); mp 90–91 °C; ^{1}H-NMR (300 MHz, $CDCl_3$, 25 °C): δ = 7.63 (dd, 1H, *J* = 7.8, 1.8 Hz), 7.49 (dd, 1H, *J* = 7.8, 1.8 Hz), 7.14 (t, 1H, *J* = 7.8 Hz), 6.51 (br d, 1H, *J* = 8.2 Hz), 5.93 (ddt, 1H, *J* = 17.1, 10.2, 6.3 Hz), 5.18 (dq, 1H, *J* = 10.2, 1.4 Hz), 5.10 (dq, 1H, *J* = 17.1, 1.4 Hz), 4.24 (d, 1H, *J* = 3.9 Hz), 3.88–3.73 (m, 1H), 3.59–3.41 (m, 2H), 2.11 (sept d, 1H, *J* = 6.9, 3.9 Hz), 1.95–1.80 (m, 2H), 1.77–1.53 (m, 3H), 1.45–1.26 (m, 2H), 1.26–1.08 (m, 3H), 1.06 (d, 3H, *J* = 6.9 Hz), 0.91 (d, 3H, *J* = 6.9 Hz); ^{13}C-NMR (75 MHz, $CDCl_3$, 25 °C): δ = 167.9, 149.0, 144.0, 135.5, 135.2, 135.1, 123.7, 117.7, 89.2, 48.0, 34.6, 33.2, 32.8, 31.9, 25.6, 24.9, 18.2, 17.4; IR (Neat): ν = 3285, 3078, 2960, 2927, 2855, 1650, 1600, 1553, 1526, 1449, 1346, 1284, 1249, 1233, 1219, 1180, 1152, 1106, 1087, 1010 cm^{-1}; HRMS (ESI^+; $MeCN/CH_2Cl_2$): *m*/*z* calculated for $C_{20}H_{29}N_2O_4$ 361.2127, found 361.2123.

N-Cyclohexyl-2-(4-methoxy-2-nitrophenoxy)-3-methylbutanamide (**2l**). Compound **2l** was prepared according to the general procedure A. Purification on a column of silica gel with a gradient of ethyl acetate in petroleum ether (from 90/10 to 70/30) as eluent gave the desired product (238 mg, 68%) as a yellow solid. R_f = 0.3 (petroleum ether/ethyl acetate 70/30); mp 120–121 °C; ^{1}H-NMR (300 MHz, $CDCl_3$, 25 °C): δ = 7.37 (d, 1H, *J* = 3.0 Hz), 7.07 (dd, 1H, *J* = 9.3, 3.0 Hz), 6.99 (d, 1H, *J* = 9.3 Hz), 6.94 (br d, 1H, *J* = 8.5 Hz), 4.58 (d, 1H, *J* = 3.9 Hz), 3.81 (s, 3H), 3.80–3.67 (m, 1H), 2.39–2.26 (m, 1H), 1.97–1.85 (m, 1H), 1.73–1.50 (m, 4H), 1.43–0.94 (m, 5H), 1.05 (d, 3H, *J* = 6.9 Hz), 1.04 (d, 3H, *J* = 6.9 Hz); ^{13}C-NMR (75 MHz, $CDCl_3$, 25 °C): δ = 168.7, 153.5, 145.2, 139.8, 121.2, 116.3, 110.2, 84.5, 56.2, 48.0, 33.1, 32.7, 31.5, 25.5, 24.8, 24.7, 18.9, 17.0; IR (Neat): ν = 3262, 2924, 2852, 1651, 1526, 1495, 1469, 1440, 1354, 1308, 1265, 1245, 1224, 1186, 1154, 1094, 1040, 1027 cm^{-1}; HRMS (ESI^+; $MeCN/CH_2Cl_2$): *m*/*z* calculated for $C_{18}H_{27}N_2O_5$ 351.1920, found 351.1903.

N-Cyclohexyl-2-(4-methoxy-2-nitrophenoxy)-3,3-dimethylbutanamide (**2m**). Compound **2m** was prepared according to the general procedure A. Purification on a column of silica gel with a gradient of ethyl acetate in petroleum ether (from 95/5 to 80/20) as eluent gave the desired product (247 mg, 68%) as a yellow solid. R_f = 0.7 (petroleum ether/ethyl acetate 60/40); mp 140–141 °C; ^{1}H-NMR (500 MHz, $CDCl_3$, 25 °C): δ = 7.97–7.92 (d, 1H, *J* = 9.8 Hz), 6.80 (br d, 1H, *J* = 8.1 Hz), 6.57–6.52 (m, 2H), 4.44 (s, 1H), 3.85 (s, 3H), 3.77–3.66 (m, 1H), 1.96–1.85 (m, 1H), 1.72–1.64 (m, 1H), 1.57–1.49 (m, 3H), 1.40–1.08 (m, 4H), 1.12 (s, 9H), 0.98–0.88 (m, 1H); ^{13}C-NMR (125 MHz, $CDCl_3$, 25 °C): δ = 167.4, 165.0, 153.5, 133.2, 128.3, 106.8, 100.5, 87.2, 56.1, 47.9, 34.8, 33.1, 32.6, 26.3, 25.4, 24.7, 24.6; IR (Neat): ν = 3315, 2930, 2855, 1646, 1591, 1540, 1512, 1480, 1466, 1446, 1397, 1367, 1345, 1312, 1287, 1264, 1249, 1228, 1207, 1172, 1090, 1058, 1030, 1018 cm^{-1}; HRMS (ESI^+; $MeCN/CH_2Cl_2$): *m*/*z* calculated for $C_{19}H_{29}N_2O_5$ 365.2076, found 365.2079.

2-(4-Bromo-2-nitrophenoxy)-3,3-dimethyl-N-phenethylbutanamide (**2n**). Compound **2n** was prepared according to the general procedure A. Purification on a column of silica gel with a gradient of ethyl acetate in petroleum ether (from 95/5 to 90/10) as eluent gave the desired product (338 mg, 78%) as a yellow solid. R_f = 0.3 (petroleum ether/ethyl acetate 80/20); mp 62–63 °C; ^{1}H-NMR (500 MHz, $CDCl_3$, 25 °C): δ = 7.89 (d, 1H, J = 2.5 Hz), 7.54 (dd, 1H, J = 9.1, 2.5 Hz), 7.20–7.12 (m, 3H), 7.01–6.97 (m, 2H), 6.86 (d, 1H, J = 9.1 Hz), 6.58–6.50 (m, 1H), 4.35 (s, 1H), 3.62 (dq, 1H, J = 13.6, 6.7 Hz), 3.47 (dtd, 1H, J = 13.6, 6.9, 5.2 Hz), 2.73 (t, 2H, J = 6.9 Hz), 1.03 (s, 9H); ^{13}C-NMR (75 MHz, $CDCl_3$, 25 °C): δ = 168.2, 149.9, 140.0, 138.0, 137.3, 128.8, 128.6, 128.4, 126.5, 116.1, 113.0, 87.3, 39.8, 35.2, 34.8, 26.2; IR (Neat): ν = 2959, 1663, 1603, 1522, 1477, 1396, 1338, 1262, 1246, 1163, 1095, 1048 cm^{-1}; HRMS (ESI$^+$; MeCN/CH_2Cl_2): *m*/*z* calculated for $C_{20}H_{23}BrN_2O_4$ 435.0919, found 435.0904.

2-(4-Bromo-2-nitrophenoxy)-N-(3,4-dimethoxyphenethyl)-3-methylbutanamide (**2o**). Compound **2o** was prepared according to the general procedure A. Purification on a column of silica gel with ethyl acetate in petroleum ether (70/30) as eluent gave the desired product (462 mg, 96%) as a yellow oil. R_f = 0.4 (petroleum ether/ethyl acetate 50/50); ^{1}H-NMR (500 MHz, $CDCl_3$, 25 °C): δ = 7.95 (d, 1H, J = 2.4 Hz), 7.57 (dd, 1H, J = 9.0, 2.4 Hz), 6.86 (d, 1H, J = 9.0 Hz), 6.79 (br t, 1H, J = 5.4 Hz), 6.68 (d, 1H, J = 8.1 Hz), 6.63 (d, 1H, J = 1.6 Hz), 6.56 (dd, 1H, J = 8.1, 1.6 Hz), 4.56 (d, 1H, J = 4.0 Hz), 3.85 (s, 3H), 3.82 (s, 3H), 3.60–3.48 (m, 2H), 2.74 (dt, 1H, J = 14.0, 7.0 Hz), 2.71 (dt, 1H, J = 14.0, 7.0 Hz), 2.34–2.25 (m, 1H), 1.01 (d, 3H, J = 7.5 Hz), 0.99 (d, 3H, J = 7.5 Hz); ^{13}C-NMR (125 MHz, $CDCl_3$, 25 °C): δ = 169.1, 150.1, 149.1, 147.8, 140.2, 137.3, 130.7, 128.8, 120.6, 116.4, 113.2, 111.7, 111.3, 84.6, 56.0, 55.9, 40.1, 35.0, 31.6, 18.8, 17.0; IR (Neat): ν = 3387, 2966, 1666, 1602, 1514, 1465, 1418, 1345, 1261, 1235, 1156, 1140, 1100, 1026, 1000 cm^{-1}; HRMS (ESI$^+$; MeCN/CH_2Cl_2): *m*/*z* calculated for $C_{21}H_{25}BrN_2O_6$ 481.0974, found 481.0969.

N-(Tert-butyl)-2-(4-chloro-2-nitrophenoxy)butanamide (**2p**). Compound **2p** was prepared according to the general procedure A. Purification on a column of silica gel with a gradient of ethyl acetate in petroleum ether (from 90/10 to 80/20) as eluent gave the desired product (500 mg, 79%) as an off-white solid. R_f = 0.5 (petroleum ether/ethyl acetate 80/20); mp 107–108 °C; ^{1}H-NMR (500 MHz, $CDCl_3$, 25 °C): δ = 7.92 (d, 1H, J = 2.5 Hz), 7.51 (dd, 1H, J = 9.1, 2.5 Hz), 7.02 (d, 1H, J = 9.1 Hz), 6.88 (br s, 1H), 4.70 (t, 1H, J = 4.8 Hz), 2.12–1.93 (m, 2H), 1.36 (s, 9H), 0.97 (t, 3H, J = 7.5 Hz); ^{13}C-NMR (125 MHz, $CDCl_3$, 25 °C): δ = 168.4, 149.3, 139.8, 134.6, 126.4, 126.2, 116.3, 80.8, 51.5, 28.7, 25.1, 8.4; IR (Neat): ν = 3308, 2973, 1655, 1606, 1555, 1527, 1481, 1461, 1393, 1349, 1270, 1249, 1221, 1163, 1120, 1107, 1057 cm^{-1}; HRMS (ESI$^+$; MeCN/CH_2Cl_2): *m*/*z* calculated for $C_{14}H_{19}ClN_2O_4$ 315.1112, found 315.1106.

N-(Tert-butyl)-2-(4-chloro-2-nitrophenoxy)-3-methylbutanamide (**2q**). Compound **2q** was prepared according to the general procedure A. Purification on a column of silica gel with a gradient of ethyl acetate in petroleum ether (from 95/5 to 90/10) as eluent gave the desired product (224 mg, 68%) as an off-white solid. R_f = 0.6 (petroleum ether/ethyl acetate 80/20); mp 111–112 °C; ^{1}H-NMR (300 MHz, $CDCl_3$, 25 °C): δ = 7.87 (d, 1H, J = 2.7 Hz), 7.50 (dd, 1H, J = 9.0, 2.7 Hz), 7.03 (d, 1H, J = 9.0 Hz), 6.68 (br s, 1H), 4.51 (d, 1H, J = 4.1 Hz), 2.32 (sept d, 1H, J = 6.9, 4.1 Hz), 1.31 (s, 9H), 1.06 (d, 3H, J = 6.9 Hz), 1.04 (d, 3H, J = 6.9 Hz); ^{13}C-NMR (75 MHz, $CDCl_3$, 25 °C): δ = 168.2, 149.7, 140.0, 134.4, 126.5, 125.9, 116.2, 84.7, 51.5, 31.5, 28.6, 18.7, 17.2; IR (Neat): ν = 3275, 3082, 2972, 1649, 1605, 1531, 1483, 1470, 1391, 1361, 1277, 1250, 1223, 1162, 1119, 1031, 1010 cm^{-1}; HRMS (ESI$^+$; MeCN/CH_2Cl_2): *m*/*z* calculated for $C_{15}H_{21}ClN_2O_4$ 329.1268, found 329.1271.

2-(4-Chloro-2-nitrophenoxy)-N-(4-chlorobenzyl)-3,3-dimethylbutanamide (**2r**). Compound **2r** was prepared according to the general procedure A. Purification on a column of silica gel with a gradient of ethyl acetate in petroleum ether (from 95/5 to 90/10) as eluent gave the desired product (352 mg, 86%) as a yellow solid. R_f = 0.3 (petroleum ether/ethyl acetate 80/20); mp 130–131 °C; ^{1}H-NMR (300 MHz, $CDCl_3$, 25 °C): δ = 7.77 (d, 1H, J = 2.6 Hz), 7.44 (dd, 1H, J = 9.0, 2.6 Hz), 7.18 (m, 2H), 7.03–6.97 (m, 4H), 4.48 (s, 1H), 4.40 (dd, 1H, J = 14.6, 6.6 Hz), 4.30 (dd, 1H, J = 14.6, 5.6 Hz), 1.09 (s, 9H); ^{13}C-NMR (125 MHz, $CDCl_3$, 25 °C): δ = 168.3, 149.4, 140.0, 136.4, 134.4, 133.5, 129.1, 128.8, 126.9, 125.8, 116.2, 87.7, 42.6, 35.0, 26.3; IR (Neat): ν = 2959, 2873, 1669, 1610, 1517, 1494, 1480, 1434, 1399, 1348, 1313,

1271, 1248, 1163, 1118, 1090, 1077, 1052, 1001 cm^{-1}; HRMS (ESI$^+$; MeCN/CH_2Cl_2): *m*/*z* calculated for $C_{19}H_{20}Cl_2N_2O_4$ 411.0878, found 411.0865.

2-(4-Chloro-2-nitrophenoxy)-N-(4-methoxybenzyl)butanamide (**2s**). Compound **2s** was prepared according to the general procedure A. Purification on a column of silica gel with a gradient of ethyl acetate in petroleum ether (from 90/10 to 80/20) as eluent gave the desired product (631 mg, 83%) as a yellow solid. R_f = 0.3 (petroleum ether/ethyl acetate 70/30); mp 103–104 °C; ^{1}H-NMR (300 MHz, $CDCl_3$, 25 °C): δ = 7.85 (d, 1H, *J* = 2.7 Hz), 7.48 (dd, 1H, *J* = 9.1, 2.7 Hz), 7.27 (br t, 1H, *J* = 5.7 Hz), 7.11 (m, 2H), 7.01 (d, 1H, *J* = 9.1 Hz), 6.80 (m, 2H), 4.82 (t, 1H, *J* = 5.2 Hz), 4.42 (dd, 1H, *J* = 14.6, 6.1 Hz), 4.37 (dd, 1H, *J* = 14.6, 6.1 Hz), 3.78 (s, 3H), 2.11–1.98 (m, 2H), 0.99 (t, 3H, *J* = 7.4 Hz); ^{13}C-NMR (75 MHz, $CDCl_3$, 25 °C): δ = 169.4, 159.1, 149.3, 139.9, 134.6, 129.9, 129.0, 126.7, 126.1, 116.4, 114.1, 81.0, 55.4, 42.8, 25.5, 8.8; IR (Neat): ν = 3274, 3066, 2932, 1643, 1611, 1526, 1513, 1483, 1459, 1437, 1353, 1301, 1272, 1248, 1229, 1181, 1162, 1138, 1121, 1105, 1060, 1029, 1000 cm^{-1}; HRMS (ESI$^+$; MeCN/CH_2Cl_2): *m*/*z* calculated for $C_{18}H_{20}ClN_2O_5$ 379.1061, found 379.1063.

2-(4-Cyano-2-nitrophenoxy)-N-(3,4-dimethoxyphenethyl)-4-methylpentanamide (**2t**). Compound **2t** was prepared according to the general procedure A. Purification on a column of silica gel with a gradient of ethyl acetate in petroleum ether (from 90/10 to 50/50) as eluent gave the desired product (327 mg, 74%) as a yellow oil. R_f = 0.4 (petroleum ether/ethyl acetate 50/50); ^{1}H-NMR (500 MHz, $CDCl_3$, 25 °C): δ = 8.11 (d, 1H, *J* = 2.0 Hz), 7.74 (dd, 1H, *J* = 8.9, 2.0 Hz), 7.06 (d, 1H, *J* = 8.9 Hz), 6.67 (d, 1H, *J* = 8.2 Hz), 6.62 (br t, 1H, *J* = 5.3 Hz), 6.60 (d, 1H, *J* = 1.9 Hz), 6.55 (dd, 1H, *J* = 8.2, 1.9 Hz), 4.81 (dd, 1H, *J* = 8.4, 3.9 Hz), 3.86 (s, 3H), 3.82 (s, 3H), 3.58 (dq, 1H, *J* = 13.5, 6.8 Hz), 3.51 (dtd, 1H, *J* = 13.5, 6.9, 5.3 Hz), 2.75 (dd, 1H, *J* = 14.1, 6.8 Hz), 2.71 (dd, 1H, *J* = 14.1, 6.8 Hz), 1.90–1.81 (m, 1H), 1.81–1.72 (m, 2H), 0.94 (d, 3H, *J* = 6.3 Hz), 0.89 (d, 3H, *J* = 6.3 Hz); ^{13}C-NMR (75 MHz, $CDCl_3$, 25 °C): δ = 169.5, 153.5, 149.0, 147.7, 139.6, 137.9, 130.3, 130.0, 120.5, 116.4, 115.6, 111.6, 111.2, 105.6, 79.5, 56.0, 55.9, 41.4, 40.0, 34.7, 24.6, 23.0, 21.9; IR (Neat): ν = 3396, 2959, 2235, 1671, 1615, 1536, 1515, 1493, 1465, 1418, 1355, 1260, 1235, 1157, 1141, 1085, 1027 cm^{-1}; HRMS (ESI$^+$; MeCN/CH_2Cl_2): *m*/*z* calculated for $C_{23}H_{28}N_3O_6$ 442.1978, found 442.1982.

2-(4-Cyano-2-nitrophenoxy)-N-(4-methoxybenzyl)butanamide (**2u**). Compound **2u** was prepared according to the general procedure A. Purification on a column of silica gel with a gradient of ethyl acetate in petroleum ether (from 90/10 to 60/40) as eluent gave the desired product (226 mg, 61%) as a yellow oil. R_f = 0.2 (petroleum ether/ethyl acetate 70/30); ^{1}H-NMR (300 MHz, $CDCl_3$, 25 °C): δ = 8.17 (d, 1H, *J* = 2.1 Hz), 7.80 (dd, 1H, *J* = 8.7, 2.1 Hz), 7.17 (d, 1H, *J* = 8.7 Hz), 7.19–7.00 (m, 3H), 6.81 (m, 2H), 4.92 (t, 1H, *J* = 5.2 Hz), 4.40 (d, 2H, *J* = 5.9 Hz), 3.78 (s, 3H), 2.18–2.00 (m, 2H), 1.00 (t, 3H, *J* = 7.4 Hz); ^{13}C-NMR (75 MHz, $CDCl_3$, 25 °C): δ = 168.6, 159.2, 153.6, 139.8, 138.1, 130.3, 129.6, 129.1, 116.4, 116.1, 114.2, 105.7, 81.4, 55.4, 42.9, 25.4, 8.8; IR (Neat): ν = 3262, 3073, 2973, 2938, 2234, 1647, 1615, 1533, 1514, 1496, 1460, 1441, 1357, 1283, 1250, 1175, 1136, 1103, 1087, 1061, 1028 cm^{-1}; HRMS (ESI$^+$; MeCN/CH_2Cl_2): *m*/*z* calculated for $C_{19}H_{19}N_3O_5$ 370.1403, found 370.1404.

4-Methyl-2-(2-nitro-4-(trifluoromethyl)phenoxy)-N-phenethylpentanamide (**2v**). Compound **2v** was prepared according to the general procedure A. Purification on a column of silica gel with a gradient of ethyl acetate in petroleum ether (from 95/5 to 80/20) as eluent gave the desired product (286 mg, 67%) as a yellow solid. R_f = 0.6 (petroleum ether/ethyl acetate 70/30); mp 79–80 °C; ^{1}H-NMR (500 MHz, $CDCl_3$, 25 °C): δ = 8.07 (d, 1H, *J* = 2.0 Hz), 7.73 (dd, 1H, *J* = 9.0, 2.0 Hz), 7.17-7.12 (m, 3H), 7.08 (d, 1H, *J* = 9.0 Hz), 7.01 (dd, 2H, *J* = 7.7, 1.9 Hz), 6.71 (br t, 1H, *J* = 6.8 Hz), 4.82 (dd, 1H, *J* = 8.4, 3.9 Hz), 3.64 (dq, 1H, *J* = 13.5, 6.8 Hz), 3.50 (dq, 1H, *J* = 13.5, 6.8 Hz), 2.78 (t, 2H, *J* = 6.8 Hz), 1.89–1.72 (m, 3H), 0.94 (d, 3H, *J* = 6.2 Hz), 0.89 (d, 3H, *J* = 6.2 Hz); ^{13}C-NMR (125 MHz, $CDCl_3$, 25 °C): δ = 170.0, 152.8, 139.5, 138.1, 131.4 (q, $J_{C\text{-}F}$ = 3.4 Hz), 128.6, 128.5, 126.6, 124.1 (q, $J_{C\text{-}F}$ = 34.4 Hz), 123.8 (q, $J_{C\text{-}F}$ = 3.7 Hz), 122.9 (q, $J_{C\text{-}F}$ = 273.1 Hz), 115.1, 79.3, 41.6, 40.0, 35.3, 24.7, 23.1, 21.9; ^{19}F-NMR (282 MHz, $CDCl_3$, 25 °C): δ = –62.2; IR (Neat): ν = 3250, 3086, 2957, 2873, 1654, 1627, 1540, 1506, 1468, 1455, 1368, 1326, 1297, 1275, 1251, 1234, 1189, 1163, 1129, 1100, 1073 cm^{-1}; HRMS (ESI$^+$; MeCN/CH_2Cl_2): *m*/*z* calculated for $C_{21}H_{24}F_3N_2O_4$ 425.1688, found 425.1690.

N-(3,4-Dimethoxyphenethyl)-4,8-dimethyl-2-(2-nitro-4-(trifluoromethyl)phenoxy)non-7-enamide (**2w**). Compound **2w** was prepared according to the general procedure A. Purification on a column of silica gel with a gradient of ethyl acetate in petroleum ether (from 95/5 to 90/10) as eluent gave the desired product (908 mg, 82%) as a 1:1 mixture of unseparable diastereomers (yellow oil). R_f = 0.3 (petroleum ether/ethyl acetate 70/30); ^{1}H-NMR (500 MHz, $CDCl_3$, 25 °C): δ = 8.12 (d, 0.5H, *J* = 2.0 Hz), 8.11 (d, 0.5H, *J* = 2.0 Hz), 7.75 (dd, 0.5H, *J* = 8.4, 2.0 Hz), 7.74 (dd, 0.5H, *J* = 8.4, 2.0 Hz), 7.11 (d, 0.5H, *J* = 8.4 Hz), 7.10 (d, 0.5H, *J* = 8.4 Hz), 6.78 (br t, 0.5H, *J* = 5.7 Hz), 6.78 (br t, 0.5H, *J* = 5.7 Hz), 6.67 (d, 0.5H, *J* = 8.0 Hz), 6.66 (d, 0.5H, *J* = 8.0 Hz), 6.64 (d, 0.5H, *J* = 1.9 Hz), 6.63 (d, 0.5H, *J* = 1.9 Hz), 6.56 (dd, 0.5H, *J* = 8.0, 2.0 Hz), 6.54 (dd, 0.5H, *J* = 8.0, 2.0 Hz), 5.05 (t, 0.5H, *J* = 7.1 Hz), 4.99 (t, 0.5H, *J* = 7.1 Hz), 4.88–4.82 (m, 1H), 3.83 (s, 3H), 3.81 (s, 3H), 3.62–3.45 (m, 2H), 2.73 (br t, 2H, *J* = 6.6 Hz), 2.05–1.57 (m, 5H), 1.66 (s, 1.5H), 1.61 (s, 1.5H), 1.58 (s, 1.5H), 1.54 (s, 1.5H), 1.39–1.11 (m, 2H), 0.94 (d, 1.5H, *J* = 6.5 Hz), 0.89 (d, 1.5H, *J* = 6.5 Hz); ^{13}C-NMR (125 MHz, $CDCl_3$, 25 °C): δ = 170.0, 169.9, 152.9, 152.8, 149.1, 147.8, 139.5, 139.4, 131.8, 131.7, 131.4 (q, $J_{C\text{-}F}$ = 3.3 Hz), 130.6, 130.5, 124.5, 124.4, 124.2, 124.1, 123.7 (q, $J_{C\text{-}F}$ = 3.9 Hz), 122.9 (q, $J_{C\text{-}F}$ = 272.2 Hz), 120.6, 115.2, 115.1, 111.8, 111.7, 111.3, 111.2, 79.3, 79.2, 55.9, 55.8, 40.3, 40.2, 40.0, 39.9, 37.4, 36.4, 35.0, 34.9, 29.0, 28.8, 25.7, 25.6, 25.3, 25.1, 19.9, 19.1, 17.7, 17.6; ^{19}F-NMR (282 MHz, $CDCl_3$, 25 °C): δ = –62.2; IR (Neat): ν = 3393, 2932, 1671, 1627, 1590, 1539, 1515, 1453, 1419, 1357, 1324, 1261, 1236, 1157, 1128, 1093, 1027 cm^{-1}; HRMS (ESI$^+$; MeCN/CH_2Cl_2): *m*/*z* calculated for $C_{28}H_{35}F_3N_2O_6$ 553.2525, found 553.2526.

N-Cyclohexyl-2-((2-nitropyridin-3-yl)oxy)butanamide (**2xa**). Compound **2xa** was prepared according to the general procedure A. Purification on a column of silica gel with a gradient of ethyl acetate in petroleum ether (from 90/10 to 60/40) as eluent gave the desired product (226 mg, 74%) as an off-white solid. R_f = 0.2 (petroleum ether/ethyl acetate 60/40); mp 122–123 °C; ^{1}H-NMR (500 MHz, $CDCl_3$, 25 °C): δ = 8.17 (dd, 1H, *J* = 4.3, 1.3 Hz), 7.56 (dd, 1H, *J* = 8.4, 4.3 Hz), 7.52 (dd, 1H, *J* = 8.4, 1,3 Hz), 6.76 (br d, 1H, *J* = 7.5 Hz), 4.79 (t, 1H, *J* = 5.3 Hz), 3.81–3.71 (m, 1H), 2.13–1.97 (m, 2H), 1.95–1.87 (m, 1H), 1.76–1.67 (m, 2H), 1.67–1.53 (m, 2H), 1.44–1.04 (m, 5H), 1.00 (t, 3H, *J* = 7.4 Hz); ^{13}C-NMR (125 MHz, $CDCl_3$, 25 °C): δ = 168.1, 148.8, 145.9, 140.5, 129.2, 124.3, 80.9, 48.2, 32.9, 32.7, 25.6, 25.4, 24.7, 24.6, 8.8; IR (Neat): ν = 3260, 3088, 2925, 2853, 1650, 1602, 1561, 1535, 1460, 1427, 1370, 1277, 1245, 1233, 1141, 1113, 1086, 1052, 1025 cm^{-1}; HRMS (ESI$^+$; MeCN/CH_2Cl_2): *m*/*z* calculated for $C_{15}H_{22}N_3O_4$ 308.1610, found 308.1607.

N-Cyclohexyl-3-methyl-2-((2-nitropyridin-3-yl)oxy)butanamide (**2xb**). Compound **2xb** was prepared according to the general procedure A. Purification on a column of silica gel with a gradient of ethyl acetate in petroleum ether (from 90/10 to 70/30) as eluent gave the desired product (246 mg, 77%) as an off-white solid. R_f = 0.2 (petroleum ether/ethyl acetate 70/30); mp 132–133 °C; ^{1}H-NMR (500 MHz, $CDCl_3$, 25 °C): δ = 8.15 (dd, 1H, *J* = 3.7, 2.1 Hz), 7.57–7.50 (m, 2H), 6.63 (br d, 1H, *J* = 7.9 Hz), 4.62 (d, 1H, *J* = 4.3 Hz), 3.80–3.68 (m, 1H), 2.34 (sept d, 1H, *J* = 6.7, 4.3 Hz), 1.95–1.87 (m, 1H), 1.77–1.52 (m, 4H), 1.42–1.00 (m, 5H), 1.06 (d, 3H, *J* = 6.7 Hz), 1.05 (d, 3H, *J* = 6.7 Hz); ^{13}C-NMR (125 MHz, $CDCl_3$, 25 °C): δ = 167.7, 148.9, 146.1, 140.4, 129.1, 124.3, 84.6, 48.1, 33.0, 32.6, 31.5, 25.4, 24.7, 24.6, 18.7, 17.0; IR (Neat): ν = 3283, 3071, 2969, 2930, 2853, 1651, 1600, 1569, 1537, 1519, 1458, 1435, 1376, 1349, 1299, 1269, 1244, 1232, 1152, 1130, 1114, 1089, 1034 cm^{-1}; HRMS (ESI$^+$; MeCN/CH_2Cl_2): *m*/*z* calculated for $C_{16}H_{24}N_3O_4$ 322.1767, found 322.1769.

N-Cyclohexyl-2-((3-nitropyridin-2-yl)oxy)butanamide (**2ya**). Compound **2ya** was prepared according to the general procedure A. Purification on a column of silica gel with a gradient of ethyl acetate in petroleum ether (from 90/10 to 80/20) as eluent gave the desired product (132 mg, 43%) as an off-white solid. R_f = 0.4 (petroleum ether/ethyl acetate 70/30); mp 142–143 °C; ^{1}H-NMR (500 MHz, $CDCl_3$, 25 °C): δ = 8.40 (dd, 1H, *J* = 4.8, 1.9 Hz), 8.34 (dd, 1H, *J* = 8.0, 1.9 Hz), 7.11 (dd, 1H, *J* = 8.0, 4.8 Hz), 7.04 (br d, 1H, *J* = 7.8 Hz), 5.77 (t, 1H, *J* = 4.9 Hz), 3.87–3.77 (m, 1H), 2.14–2.06 (m, 2H), 2.00–1.92 (m, 1H), 1.86–1.78 (m, 1H), 1.78–1.54 (m, 3H), 1.45–1.13 (m, 5H,), 0.95 (t, 3H, *J* = 7.4 Hz); ^{13}C-NMR (125 MHz, $CDCl_3$, 25 °C): δ = 169.0, 154.9, 152.3, 135.7, 133.9, 117.7, 77.9, 47.9, 33.1, 32.8, 25.6, 25.0, 24.7, 8.4; IR (Neat): ν = 3283, 3070, 2969, 2930, 2854, 1652, 1600, 1570, 1552, 1517, 1456, 1439, 1376, 1348, 1301, 1268,

1242, 1233, 1149, 1130, 1091 cm^{-1}; HRMS (ESI^+; $MeCN/CH_2Cl_2$): *m*/*z* calculated for $C_{15}H_{22}N_3O_4$ 308.1610, found 308.1615.

N-Cyclohexyl-3-methyl-2-((3-nitropyridin-2-yl)oxy)butanamide (**2yb**). Compound **2yb** was prepared according to the general procedure A. Purification on a column of silica gel with a gradient of ethyl acetate in petroleum ether (from 95/5 to 80/20) as eluent gave the desired product (184 mg, 57%) as an off-white solid. R_f = 0.5 (petroleum ether/ethyl acetate 70/30); mp 131–132 °C; ^{1}H-NMR (300 MHz, $CDCl_3$, 25 °C): δ = 8.38 (dd, 1H, *J* = 4.8, 1.8 Hz), 8.30 (dd, 1H, *J* = 8.0, 1.8 Hz), 7.10 (dd, 1H, *J* = 8.0, 4.8 Hz), 6.91 (br d, 1H, *J* = 8.2 Hz), 5.78 (d, 1H, *J* = 3.4 Hz), 3.85–3.71 (m, 1H), 2.44 (sept d, 1H, *J* = 6.9, 3.4 Hz), 2.00–1.88 (m, 1H), 1.78–1.49 (m, 4H), 1.45–1.06 (m, 5H), 1.03 (s, 3H, *J* = 6.9 Hz), 1.00 (d, 3H, *J* = 6.9 Hz); ^{13}C-NMR (75 MHz, $CDCl_3$, 25 °C): δ = 168.6, 155.1, 152.0, 135.4, 133.9, 117.6, 80.7, 47.7, 33.0, 32.7, 31.0, 25.4, 24.6, 18.7, 16.7; IR (Neat): ν = 3275, 3096, 2933, 2854, 1651, 1604, 1570, 1520, 1441, 1386, 1345, 1318, 1300, 1266, 1243, 1152, 1093, 1020 cm^{-1}; HRMS (ESI^+; $MeCN/CH_2Cl_2$): *m*/*z* calculated for $C_{16}H_{24}N_3O_4$ 322.1767, found 322.1757.

N-Cyclohexyl-3-methyl-2-(4-nitrophenoxy)butanamide (**2z**). Compound **2z** was prepared according to the general procedure A. Purification on a column of silica gel with a gradient of ethyl acetate in petroleum ether (from 90/10 to 80/20) as eluent gave the desired product (256 mg, 40%) as a yellow solid. R_f = 0.6 (petroleum ether/ethyl acetate 70/30); mp 155–156 °C; ^{1}H-NMR (300 MHz, $CDCl_3$, 25 °C): δ = 8.20 (d, 1H, *J* = 9.3 Hz), 6.99 (d, 1H, *J* = 9.3 Hz), 5.91 (br d, 1H, *J* = 8.7 Hz), 4.39 (d, 1H, *J* = 4.5 Hz), 3.88–3.69 (m, 1H), 2.39–2.23 (m, 1H), 1.97–1.82 (m, 1H), 1.75–1.62 (m, 3H), 1.62–1.50 (m, 1H), 1.45–1.20 (m, 2H), 1.20–0.79 (m, 3H), 1.06 (d, 3H, *J* = 6.8 Hz), 1.02 (d, 3H, *J* = 6.8 Hz); ^{13}C-NMR (75 MHz, $CDCl_3$, 25 °C): δ = 168.6, 162.9, 142.5, 126.2, 115.5, 84.7, 48.0, 33.2, 32.9, 31.8, 25.4, 24.8, 24.7, 18.9, 17.2; IR (Neat): ν = 3265, 3091, 2964, 2926, 2876, 2853, 1647, 1608, 1592, 1558, 1506, 1496, 1469, 1448, 1422, 1390, 1372, 1345, 1330, 1314, 1300, 1254, 1244, 1232, 1173, 1154, 1113, 1091, 1042 cm^{-1}; HRMS (ESI^+; $MeCN/CH_2Cl_2$): *m*/*z* calculated for $C_{17}H_{25}N_2O_4$ 321.1814, found 321.1808.

N-Cyclohexyl-2-(1-(2-nitrophenoxy)propoxy)butanamide (**3a**). The product **3a** was isolated during the first step of the reaction as a mixture of two diastereomers, and one of them cannot be separated from the Passerini-Smiles adduct.

Diastereomer 1 (contaminated by Passerini-Smiles adduct)

R_f = 0.4 (petroleum ether/ethyl acetate 70/30); ^{1}H-NMR (500 MHz, CD_2Cl_2, 25 °C): δ = 7.75 (dd, , 1H *J* = 8.0, 2.0 Hz), 7.51 (td, 1H, *J* = 8.0, 2.0 Hz), 7.18 (d, 1H, *J* = 8.0 Hz), 7.09 (t, 1H, *J* = 8.0 Hz), 6.12 (br d, 1H, *J* = 7.7 Hz), 5.30 (t, 1H, *J* = 5.3 Hz), 4.06 (t, 1H, *J* = 5.5 Hz), 3.72–3.61 (m, 1H), 1.97–1.62 (m, 8H), 1.61–1.54 (m, 1H), 1.38–1.24 (m, 2H), 1.21–1.01 (m, 3H), 1.03 (t, 3H, *J* = 7.5 Hz), 0.86 (t, 3H, *J* = 7.5 Hz); ^{13}C-NMR (125 MHz, CD_2Cl_2, 25 °C): δ = 170.5, 149.9, 141.9, 134.0, 125.7, 122.1, 117.9, 105.1, 79.1, 48.1, 33.4, 33.3, 27.6, 26.8, 25.9, 25.22, 25.17, 8.9, 8.8.

Diastereomer 2

R_f = 0.2 (petroleum ether/ethyl acetate 70/30); ^{1}H-NMR (500 MHz, CD_2Cl_2, 25 °C): δ = 7.76 (dd, 1H, *J* = 8.0, 1.7 Hz), 7.52 (td, 1H, *J* = 8.0, 1.7 Hz), 7.19 (d, 1H, *J* = 8.0 Hz), 7.10 (t, 1H, *J* = 8.0 Hz), 6.42 (br d, 1H, *J* = 7.1 Hz), 5.39 (t, 1H, *J* = 5.3 Hz), 4.07 (t, 1H, *J* = 5.1 Hz), 3.72–3.62 (m, 1H), 1.98–1.64 (m, 8H), 1.64–1.58 (m, 1H), 1.39–1.29 (m, 2H), 1.23–1.08 (m, 3H), 1.03 (t, 3H, *J* = 7.5 Hz), 0.86 (t, 3H, *J* = 7.5 Hz); ^{13}C-NMR (125 MHz, CD_2Cl_2, 25 °C): δ = 170.4, 149.9, 141.7, 134.1, 125.7, 122.2, 118.0, 105.5, 79.4, 48.2, 33.4, 33.0, 27.6, 26.1, 26.0, 25.3, 8.7, 8.5; IR (Neat): ν = 3301, 2931, 2855, 1654, 1605, 1583, 1524, 1484, 1451, 1350, 1313, 1276, 1249, 1150, 1110, 1087, 1024 cm^{-1}; HRMS (ESI^-; $MeCN/CH_2Cl_2$): *m*/*z* calculated for $C_{19}H_{28}N_2O_5$ 409.1975, found 409.1990.

N-Cyclohexyl-2-(2-fluoro-4-nitrophenoxy)-3-methylbutanamide (**4a**). Compound **4a** was prepared according to the general procedure B. Purification on a column of silica gel with a gradient of ethyl

acetate in petroleum ether (from 95/5 to 90/10) as eluent gave the desired product (275 mg, 81%) as a white solid. R_f = 0.5 (petroleum ether/ethyl acetate 80/20); mp 157–158 °C; ^{1}H-NMR (300 MHz, $CDCl_3$, 25 °C): δ = 8.06–7.98 (m, 2H), 7.03 (t, 1H, J = 8.6 Hz), 6.09 (br d, 1H, J = 8.5 Hz), 4.44 (d, 1H, J = 4.5 Hz), 3.87–3.72 (m, 1H), 2.35 (sept d, 1H, J = 6.9, 4.5 Hz), 1.97–1.86 (m, 1H), 1.77–1.54 (m, 4H), 1.44–0.90 (m, 5H), 1.08 (d, 6H, J = 6.9 Hz); ^{13}C-NMR (125 MHz, $CDCl_3$, 25 °C): δ = 168.2, 151.8 (d, $J_{C\text{-}F}$ = 251.6 Hz), 151.7 (d, $J_{C\text{-}F}$ = 10.9 Hz), 142.0 (d, $J_{C\text{-}F}$ = 7.3 Hz), 121.0 (d, $J_{C\text{-}F}$ = 3.7 Hz), 115.3, 112.9 (d, $J_{C\text{-}F}$ = 23.1 Hz), 86.2, 48.1, 33.2, 32.9, 31.9, 25.5, 24.8, 24.7, 18.8, 17.2; ^{19}F-NMR (282 MHz, $CDCl_3$, 25 °C): δ = –129.3; IR (Neat): ν = 3268, 3085, 2930, 2854, 1648, 1603, 1562, 1519, 1504, 1471, 1446, 1389, 1349, 1334, 1296, 1275, 1247, 1215, 1154, 1138, 1088, 1077, 1037 cm^{-1}; HRMS (ESI$^+$; MeCN/CH_2Cl_2): m/z calculated for $C_{17}H_{24}FN_2O_4$ 339.1720, found 339.1719.

2-(2-Chloro-4-nitrophenoxy)-N-cyclohexyl-3-methylbutanamide (**4b**). Compound **4b** was prepared according to the general procedure A. Purification on a column of silica gel with a gradient of ethyl acetate in petroleum ether (from 90/10 to 70/30) as eluent gave the desired product (486 mg, 68%) as a yellow solid. R_f = 0.7 (petroleum ether/ethyl acetate 70/30); mp 174–175 °C; ^{1}H-NMR (300 MHz, $CDCl_3$, 25 °C): δ = 8.31 (d, 1H, J = 2.7 Hz), 8.12 (dd, 1H, J = 9.1, 2.7 Hz), 6.97 (d, 1H, J = 9.1 Hz), 6.12 (br d, 1H, J = 8.4 Hz), 4.51 (d, 1H, J = 4.2 Hz), 3.86–3.71 (m, 1H), 2.43–2.29 (m, 1H), 1.97–1.85 (m, 1H), 1.78–1.51 (m, 4H), 1.44–0.81 (m, 5H), 1.10 (d, 3H, J = 6.3 Hz), 1.08 (d, 3H, J = 6.3 Hz); ^{13}C-NMR (75 MHz, $CDCl_3$, 25 °C): δ = 168.0, 158.4, 142.1, 126.3, 124.2, 124.0, 113.7, 85.6, 48.1, 33.1, 32.8, 31.8, 25.4, 24.7, 24.6, 18.9, 17.1; IR (Neat): ν = 3261, 3094, 2930, 2855, 1648, 1585, 1563, 1510, 1486, 1470, 1446, 1346, 1319, 1293, 1270, 1245, 1233, 1155, 1123, 1091, 1053, 1027 cm^{-1}; HRMS (ESI$^+$; MeCN/CH_2Cl_2): m/z calculated for $C_{17}H_{24}ClN_2O_4$ 355.1425, found 355.1428.

N-Cyclohexyl-2-(2,4-dinitrophenoxy)-3-methylbutanamide (**4c**). Compound **4c** was prepared according to the general procedure A. Purification on a column of silica gel with a gradient of ethyl acetate in petroleum ether (from 90/10 to 50/50) as eluent gave the desired product (481 mg, 66%) as an orange solid. R_f = 0.7 (petroleum ether/ethyl acetate 50/50); mp 178–179 °C; ^{1}H-NMR (300 MHz, $CDCl_3$, 25 °C): δ = 8.78 (d, 1H, J = 2.8 Hz), 8.42 (dd, 1H, J = 9.4, 2.8 Hz), 7.23 (d, 1H, J = 9.4 Hz), 6.60 (br d, 1H, J = 8.0 Hz), 4.75 (d, 1H, J = 4.1 Hz), 3.83–3.67 (m, 1H), 2.38 (sept d, 1H, J = 6.9, 4.1 Hz), 1.96–1.86 (m, 1H), 1.76–1.50 (m, 4H), 1.44–0.88 (m, 5H), 1.08 (d, 3H, J = 6.9 Hz), 1.06 (d, 3H, J = 6.9 Hz); ^{13}C-NMR (75 MHz, $CDCl_3$, 25 °C): δ = 167.1, 155.4, 140.9, 139.2, 129.5, 122.3, 115.3, 85.4, 48.2, 33.0, 32.7, 31.6, 25.4, 24.7, 24.6, 18.6, 17.1; IR (Neat): ν = 3267, 2926, 2855, 1644, 1602, 1561, 1534, 1516, 1486, 1448, 1342, 1319, 1296, 1274, 1244, 1231, 1158, 1084, 1066, 1018 cm^{-1}; HRMS (ESI$^+$; MeCN/CH_2Cl_2): m/z calculated for $C_{17}H_{24}N_3O_6$ 366.1665, found 366.1682.

2-(2-Bromo-4-nitrophenoxy)-N-cyclohexyl-3,3-dimethylbutanamide (**4d**). Compound **4d** was prepared according to the general procedure A. Purification on a column of silica gel with a gradient of ethyl acetate in petroleum ether (from 95/5 to 90/10) as eluent gave the desired product (298 mg, 72%) as a white solid. R_f = 0.6 (petroleum ether/ethyl acetate 80/20); mp 168–169 °C; ^{1}H-NMR (500 MHz, $CDCl_3$, 25 °C): δ = 8.48 (d, 1H, J = 2.7 Hz), 8.15 (dd, 1H, J = 9.2, 2.7 Hz), 6.93 (d, 1H, J = 9.2 Hz), 5.86 (br d, 1H, J = 8.4 Hz), 4.33 (s, 1H), 3.81–3.71 (m, 1H), 1.94–1.85 (m, 1H), 1.71–1.50 (m, 4H), 1.42–1.06 (m, 4H), 0.97–0.88 (m, 1H), 1.16 (s, 9H); ^{13}C-NMR (125 MHz, $CDCl_3$, 25 °C): δ = 167.0, 159.1, 142.4, 129.3, 124.9, 113.7, 112.5, 88.5, 48.0, 35.1, 33.2, 32.7, 26.4, 25.4, 24.7, 24.6; IR (Neat): ν = 3275, 3085, 2930, 2856, 1650, 1583, 1553, 1523, 1478, 1448, 1397, 1362, 1339, 1314, 1278, 1250, 1238, 1193, 1144, 1119, 1094, 1062, 1041, 1016 cm^{-1}; HRMS (ESI$^+$; MeCN/CH_2Cl_2): m/z calculated for $C_{18}H_{26}BrN_2O_4$ 413.1076, found 413.1068.

N-(4-Chlorobenzyl)-2-(2-fluoro-4-nitrophenoxy)-4,8-dimethylnon-7-enamide (**4e**). Compound **4e** was prepared according to the general procedure B. Purification on a column of silica gel with a gradient of ethyl acetate in petroleum ether (from 95/5 to 90/10) as eluent gave the desired product (334 mg, 72%) as a 1:1 mixture of unseparable diastereomers (yellow oil). R_f = 0.7 (petroleum ether/ethyl acetate 80/20); ^{1}H-NMR (500 MHz, $CDCl_3$, 25 °C): δ = 8.05–7.99 (m, 2H), 7.29–7.23 (m, 2H), 7.10 (dd, 2H, J = 8.3, 2.0 Hz), 7.02 (br t, 1H, J = 8.6 Hz), 6.54 (br t, 1H, J = 6.3 Hz), 5.08 (t, 0.5H, J = 7.1 Hz), 5.00 (t, 0.5H, J = 7.1 Hz), 4.78–4.74 (m, 1H), 4.45 (dd, 1H, J = 14.9, 5.8 Hz), 4.38 (dd, 0.5H, J = 14.9, 5.8 Hz), 4.38 (dd,

0.5H, *J* = 14.9, 5.8 Hz), 2.07–1.84 (m, 3.5H), 1.82–1.71 (m, 1.5H), 1.68 (s, 1.5H), 1.63 (s, 1.5H), 1.60 (s, 1.5H), 1.55 (s, 1.5H), 1.41–1.33 (m, 1H), 1.31–1.15 (m, 1H), 1.00 (d, 1.5H, *J* = 6.7 Hz), 0.92 (d, 1.5H, *J* = 6.2 Hz); ^{13}C-NMR (75 MHz, $CDCl_3$, 25 °C): δ = 170.5, 170.4, 151.5 (d, J_{C-F} = 249.4 Hz), 151.1 (d, J_{C-F} = 11.0 Hz), 151.0 (d, J_{C-F} = 11.0 Hz), 142.0 (d, J_{C-F} = 7.6 Hz), 136.1, 133.7, 131.84, 131.77, 129.0, 124.2, 124.1, 121.0 (d, J_{C-F} = 4.0 Hz), 114.8, 112.93 (d, J_{C-F} = 23.1 Hz), 112.83 (d, J_{C-F} = 23.1 Hz), 80.1, 79.9, 42.6, 40.4, 40.3, 37.4, 36.4, 29.2, 28.9, 25.8, 25.7, 25.4, 25.2, 20.0, 19.0, 17.8, 17.7; ^{19}F-NMR (282 MHz, $CDCl_3$, 25 °C): δ = –128.9, –128.8; IR (Neat): ν = 3289, 3088, 2961, 2915, 2854, 1655, 1603, 1520, 1501, 1454, 1431, 1409, 1378, 1345, 1277, 1248, 1215, 1177, 1138, 1090, 1073, 1014 cm^{-1}; HRMS (ESI$^+$; MeCN/CH_2Cl_2): *m*/*z* calculated for $C_{24}H_{29}ClFN_2O_4$ 463.1800, found 463.1823.

2-(2-Fluoro-4-nitrophenoxy)-N-phenethyl-4-phenylbutanamide (**4f**). Compound **4f** was prepared according to the general procedure B. Purification on a column of silica gel with with a gradient of ethyl acetate in petroleum ether (90/10 to 80/20) as eluent gave the desired product (579 mg, 69%) as a yellow solid. R_f = 0.3 (petroleum ether/ethyl acetate 80/20); mp 113–114 °C; ^{1}H-NMR (500 MHz, $CDCl_3$, 25 °C): δ = 8.00 (dd, 1H, *J* = 10.4, 2.7 Hz), 7.93 (m, 1H), 7.32–7.24 (m, 2H), 7.24–7.16 (m, 4H), 7.13 (d, 2H, *J* = 7.2 Hz), 7.06 (d, 2H, *J* = 7.2 Hz), 6.77 (dd, 1H, *J* = 9.1, 7.9 Hz), 6.28 (br t, 1H, *J* = 5.7 Hz), 4.60 (dd, 1H, *J* = 7.3, 4.8 Hz), 3.62 (dq, 1H, *J* = 19.7, 6.7 Hz), 3.52 (dq, 1H, *J* = 19.7, 6.7 Hz), 2.86–2.70 (m, 4H), 2.33–2.20 (m, 2H); ^{13}C-NMR (75 MHz, $CDCl_3$, 25 °C): δ = 169.6, 151.4 (d, J_{C-F} = 251.5 Hz), 150.7 (d, J_{C-F} = 10.9 Hz), 141.9 (d, J_{C-F} = 7.0 Hz), 140.1, 138.1, 128.8, 128.7, 128.62, 128.57, 126.8, 126.5, 121.0 (d, J_{C-F} = 3.8 Hz), 114.6, 112.8 (d, J_{C-F} = 22.7 Hz), 79.8, 40.1, 35.4, 34.3, 31.0; ^{19}F-NMR (282 MHz, $CDCl_3$, 25 °C): δ = –129.1; IR (Neat): ν = 3284, 3088, 3064, 3028, 2931, 2859, 1646, 1601, 1556, 1519, 1497, 1454, 1345, 1333, 1286, 1247, 1219, 1182, 1156, 1141, 1073, 1029, 1011 cm^{-1}; HRMS (ESI$^+$; MeCN/CH_2Cl_2): *m*/*z* calculated for $C_{24}H_{24}FN_2O_4$ 423.1720, found 423.1715.

2-(2-Fluoro-4-nitrophenoxy)-N-(4-methoxybenzyl)-3-methylbutanamide (**4g**). Compound **4g** was prepared according to the general procedure B. Purification on a column of silica gel with ethyl acetate in petroleum ether (80/20) as eluent gave the desired product (573 mg, 76%) as an off-white solid. R_f = 0.3 (petroleum ether/ethyl acetate 70/30); mp 113–114 °C; ^{1}H-NMR (500 MHz, $CDCl_3$, 25 °C): δ = 8.02–7.96 (m, 2H), 7.09 (d, 2H, *J* = 8.5 Hz), 7.01 (t, 1H, *J* = 8.5 Hz), 6.80 (d, 2H, *J* = 8.5 Hz), 6.44 (br t, 1H, *J* = 5.4 Hz), 4.51 (d, 1H, *J* = 4.7 Hz), 4.43 (dd, 1H, *J* = 14.5, 5.9 Hz), 4.35 (dd, 1H, *J* = 14.5, 5.9 Hz), 3.78 (s, 3H), 2.41–2.32 (m, 1H), 1.091 (d, 3H, *J* = 6.8 Hz), 1.086 (d, 3H, *J* = 6.8 Hz); ^{13}C-NMR (75 MHz, $CDCl_3$, 25 °C): δ = 169.1, 159.2, 151.58 (d, J_{C-F} = 252.1 Hz), 151.56 (d, J_{C-F} = 10.6 Hz), 141.9 (d, J_{C-F} = 7.2 Hz), 129.7, 129.1, 121.0 (d, J_{C-F} = 3.7 Hz), 115.1, 114.2, 112.8 (d, J_{C-F} = 23.2 Hz), 86.0, 55.3, 42.8, 31.9, 18.8, 17.3; ^{19}F-NMR (282 MHz, $CDCl_3$, 25 °C): δ = –129.1; IR (Neat): ν = 3270, 3089, 3042, 2964, 2930, 2878, 2833, 1639, 1613, 1604, 1587, 1537, 1514, 1501, 1461, 1441, 1389, 1337, 1293, 1277, 1249, 1213, 1183, 1176, 1150, 1139, 1111, 1076, 1057, 1032 cm^{-1}; HRMS (ESI$^+$; MeCN/CH_2Cl_2): *m*/*z* calculated for $C_{19}H_{22}FN_2O_5$ 377.1513, found 377.1531.

2-(2-Fluoro-4-nitrophenoxy)-3,3-dimethyl-N-phenethylbutanamide (**4h**). Compound **4h** was prepared according to the general procedure B. Purification on a column of silica gel with a gradient of ethyl acetate in petroleum ether (from 95/5 to 90/10) as eluent gave the desired product (354 mg, 47%) as a yellow oil. R_f = 0.5 (petroleum ether/ethyl acetate 80/20); ^{1}H-NMR (500 MHz, $CDCl_3$, 25 °C): δ = 8.00–7.93 (m, 2H), 7.21–7.13 (m, 3H), 7.02 (dd, 2H, *J* = 7.3, 2.0 Hz), 6.89 (t, 1H, *J* = 8.5 Hz), 6.04 (br t, 1H, *J* = 5.2 Hz), 4.25 (s, 1H), 3.61 (dq, 1H, *J* = 13.6, 6.8 Hz), 3.51 (dq, 1H, *J* = 13.6, 6.8 Hz), 2.76 (t, 2H, *J* = 6.8 Hz), 1.07 (s, 9H); ^{13}C-NMR (75 MHz, $CDCl_3$, 25 °C): δ = 168.3, 151.5 (d, J_{C-F} = 10.6 Hz), 151.4 (d, J_{C-F} = 250.7 Hz), 141.7 (d, J_{C-F} = 7.1 Hz), 138.1, 128.7, 128.5, 126.7, 121.0 (d, J_{C-F} = 4.0 Hz), 114.4, 112.7 (d, J_{C-F} = 23.3 Hz), 88.4, 39.9, 35.4, 35.1, 26.2; ^{19}F-NMR (282 MHz, $CDCl_3$, 25 °C): δ = –129.7; IR (Neat): ν = 3299, 3089, 3029, 2960, 1655, 1603, 1520, 1504, 1480, 1454, 1398, 1367, 1348, 1332, 1281, 1249, 1213, 1139, 1074, 1053, 1011 cm^{-1}; HRMS (ESI$^+$; MeCN/CH_2Cl_2): *m*/*z* calculated for $C_{20}H_{24}FN_2O_4$ 375.1720, found 375.1721.

2-(2-Fluoro-4-nitrophenoxy)-N-(4-methoxybenzyl)-3,3-dimethylhex-5-enamide (**4i**). Compound **4i** was prepared according to the general procedure B. Purification on a column of silica gel with ethyl

acetate in petroleum ether (80/20) as eluent gave the desired product (590 mg, 71%) as a yellow solid. R_f = 0.5 (petroleum ether/ethyl acetate 70/30); mp 96–97 °C; ^{1}H-NMR (500 MHz, $CDCl_3$, 25 °C): δ = 8.00–7.94 (m, 2H), 7.04 (d, 2H, J = 8.6 Hz), 6.95 (br t, 1H, J = 8.7 Hz), 6.77 (d, 2H, J = 8.5 Hz), 6.29 (br t, 1H, J = 5.9 Hz), 5.82 (m, 1H), 5.04 (dd, 1H, J = 10.2, 2.1 Hz), 4.96 (br d, 1H, J = 17.0 Hz), 4.40 (s, 1H), 4.37 (dd, 1H, J = 14.5, 6.0 Hz), 4.34 (dd, 1H, J = 14.5, 6.0 Hz), 3.77 (s, 3H), 2.30 (dd, 1H, J = 13.7, 8.3 Hz), 2.16 (dd, 1H, J = 13.7, 6.7 Hz), 1.12 (s, 3H), 1.10 (s, 3H); ^{13}C-NMR (75 MHz, $CDCl_3$, 25 °C): δ = 168.2, 159.3, 151.40 (d, J_{C-F} = 251.0 Hz), 151.38 (d, J_{C-F} = 10.2 Hz), 141.9 (d, J_{C-F} = 7.3 Hz), 133.8, 129.7, 129.2, 121.0 (d, J_{C-F} = 3.8 Hz), 118.9, 114.7 (d, J_{C-F} = 1.6 Hz), 114.2, 112.7 (d, J_{C-F} = 22.5 Hz), 86.7, 55.4, 43.8, 42.9, 38.2, 23.51, 23.46; ^{19}F-NMR (282 MHz, $CDCl_3$, 25 °C): δ = –129.4; IR (Neat): ν = 3285, 3083, 2975, 2955, 2834, 1649, 1615, 1601, 1561, 1514, 1464, 1390, 1370, 1343, 1317, 1286, 1274, 1244, 1214, 1174, 1140, 1112, 1095, 1074, 1057, 1039, 1002 cm^{-1}; HRMS (ESI$^+$; MeCN/CH_2Cl_2): m/z calculated for $C_{22}H_{26}FN_2O_5$ 417.1826, found 417.1822.

N-Cyclohexyl-2-(2-fluoro-4-nitrophenoxy)butanamide (**4j**). Compound **4j** was prepared according to the general procedure B. Purification on a column of silica gel with ethyl acetate in petroleum ether (80/20) as eluent gave the desired product (493 mg, 76%) as a yellow solid. R_f = 0.3 (petroleum ether/ethyl acetate 80/20); mp 132–133 °C; ^{1}H-NMR (500 MHz, $CDCl_3$, 25 °C): δ = 8.07–8.00 (m, 2H), 7.04 (t, 1H, J = 8.3 Hz), 6.19 (br d, 1H, J = 8.4 Hz), 4.64 (t, 1H, J = 5.5 Hz), 3.85–3.76 (m, 1H), 2.05 (qd, 2H, J = 7.4, 5.5 Hz), 1.96–1.89 (m, 1H), 1.86–1.74 (m, 1H), 1.74–1.67 (m, 1H), 1.67–1.58 (m, 2H), 1.43–1.31 (m, 2H), 1.22–1.13 (m, 2H), 1.10–1.01 (m, 1H), 1.05 (t, 3H, J = 7.4 Hz); ^{13}C-NMR (75 MHz, $CDCl_3$, 25 °C): δ = 168.6, 151.7 (d, J_{C-F} = 252.1 Hz), 151.1 (d, J_{C-F} = 10.4 Hz), 142.0 (d, J_{C-F} = 7.3 Hz), 121.0 (d, J_{C-F} = 3.7 Hz), 115.2, 112.9 (d, J_{C-F} = 23.7 Hz), 82.1, 48.1, 33.0, 32.9, 26.0, 25.4, 24.8, 24.7, 9.02; ^{19}F-NMR (282 MHz, $CDCl_3$, 25 °C): δ = –129.3; IR (Neat): ν = 3262, 3087, 2928, 2853, 1649, 1614, 1604, 1557, 1519, 1502, 1465, 1447, 1346, 1336, 1283, 1245, 1234, 1218, 1144, 1107, 1075, 1049 cm^{-1}; HRMS (ESI$^+$; MeCN/CH_2Cl_2): m/z calculated for $C_{16}H_{22}FN_2O_4$ 325.1564, found 325.1563.

N-(3,4-Dimethoxyphenethyl)-2-(2-fluoro-4-nitrophenoxy)-4,8-dimethylnon-7-enamide (**4k**). Compound **4k** was prepared according to the general procedure B. Purification on a column of silica gel with a gradient of ethyl acetate in petroleum ether (from 80/20 to 70/30) as eluent gave the desired product (701 mg, 70%) as a 1:1 mixture of unseparable diastereomers (yellow oil). R_f = 0.3 (petroleum ether/ethyl acetate 70/30); ^{1}H-NMR (500 MHz, $CDCl_3$, 25 °C): δ = 8.01–7.94 (m, 2H), 6.91–6.84 (m, 1H), 6.67 (dd, 1H, J = 8.1, 2.3 Hz), 6.61 (d, 1H, J = 2.3 Hz), 6.53 (dt, 1H, J = 8.1, 2.3 Hz), 6.18 (br t, 0.5H, J = 5.0 Hz), 6.17 (br t, 0.5H, J = 5.0 Hz), 5.07 (t, 0.5H, J = 7.0 Hz), 4.99 (t, 0.5H, J = 7.0 Hz), 4.64 (m, 1H), 3.83 (s, 3H), 3.82 (s, 3H), 3.62–3.53 (m, 1H), 3.52–3.45 (m, 1H), 2.72 (t, 2H, J = 6.9 Hz), 2.04–1.87 (m, 3H), 1.83–1.77 (m, 0.5H), 1.76–1.69 (m, 1.5H), 1.67 (s, 1.5H), 1.62 (s, 1.5H), 1.59 (s, 1.5H), 1.55 (s, 1.5H), 1.40–1.31 (m, 1H), 1.28–1.12 (m, 1H), 0.97 (d, 1.5H, J = 6.6 Hz), 0.89 (d, 1.5H, J = 6.1 Hz); ^{13}C-NMR (75 MHz, $CDCl_3$, 25 °C): δ = 169.7, 169.6, 150.8 (d, J_{C-F} = 249.9 Hz), 150.5 (d, J_{C-F} = 10.2 Hz), 150.4 (d, J_{C-F} = 10.2 Hz), 148.6, 147.4, 141.3 (d, J_{C-F} = 7.4 Hz), 131.4, 131.3, 130.1, 123.9, 123.8, 120.6 (d, J_{C-F} = 3.3 Hz), 120.3, 114.0, 112.5 (d, J_{C-F} = 22.6 Hz), 112.4 (d, J_{C-F} = 22.6 Hz), 111.3, 111.0, 79.9, 79.7, 56.04, 56.00, 40.6, 40.5, 40.4, 37.7, 36.6, 35.21, 35.19, 29.6, 29.2, 26.12, 26.09, 25.7, 25.5, 20.4, 19.3, 18.2, 18.1; ^{19}F-NMR (282 MHz, $CDCl_3$, 25 °C): δ = –129.17, –129.22; IR (Neat): ν = 3347, 2929, 1667, 1604, 1515, 1453, 1419, 1345, 1277, 1261, 1235, 1214, 1156, 1139, 1074, 1027 cm^{-1}; HRMS (ESI$^+$; MeCN/CH_2Cl_2): m/z calculated for $C_{27}H_{36}FN_2O_6$ 503.2557, found 503.2536.

N-(4-Chlorobenzyl)-2-(2-fluoro-4-nitrophenoxy)butanamide (**4l**). Compound **4l** was prepared according to the general procedure B. Purification on a column of silica gel with a gradient of ethyl acetate in petroleum ether (from 90/10 to 70/30) as eluent gave the desired product (533 mg, 73%) as a yellow solid. R_f = 0.3 (petroleum ether/ethyl acetate 70/30); mp 116–117 °C; ^{1}H-NMR (500 MHz, $CDCl_3$, 25 °C): δ = 8.05–8.00 (m, 2H), 7.28 (d, 2H, J = 8.4 Hz), 7.15 (d, 2H, J = 8.4 Hz), 7.03 (dd, 1H, J = 9.1, 8.2 Hz), 6.68 (br t, 1H, J = 5.8 Hz), 4.74 (t, 1H, J = 5.4 Hz), 4.50 (dd, 1H, J = 15.0, 6.1 Hz), 4.41 (dd, 1H, J = 15.0, 6.1 Hz), 2.08 (qd, 2H, J = 7.4, 5.4 Hz), 1.05 (t, 3H, J = 7.4 Hz); ^{13}C-NMR (75 MHz, $CDCl_3$, 25 °C): δ = 169.7, 151.7 (d, J_{C-F} = 251.1 Hz), 150.8 (d, J_{C-F} = 10.5 Hz), 142.1 (d, J_{C-F} = 7.4 Hz), 136.2, 133.7, 129.1, 129.0, 121.0 (d, J_{C-F} = 3.5 Hz), 115.2, 113.0 (d, J_{C-F} = 23.0 Hz), 81.9, 42.6, 25.9, 9.1; ^{19}F-NMR (282 MHz, $CDCl_3$,

25 °C): δ = –129.0; IR (Neat): ν = 3248, 3093, 2988, 2925, 1653, 1616, 1603, 1553, 1508, 1490, 1461, 1410, 1383, 1349, 1332, 1310, 1282, 1261, 1242, 1209, 1145, 1105, 1092, 1077, 1047, 1013 cm^{-1}; HRMS (ESI$^+$; MeCN/CH_2Cl_2): *m/z* calculated for $C_{17}H_{17}ClFN_2O_4$ 367.0861, found 367.0875.

N-(Tert-butyl)-2-(2-fluoro-4-nitrophenoxy)-3-methylbutanamide (**4m**). Compound **4m** was prepared according to the general procedure B. Purification on a column of silica gel with a gradient of ethyl acetate in petroleum ether (from 95/5 to 90/10) as eluent gave the desired product (191 mg, 31%) as an off-white solid. R_f = 0.7 (petroleum ether/ethyl acetate 70/30); mp 108–109 °C; ^{1}H-NMR (500 MHz, $CDCl_3$, 25 °C): δ = 8.04–8.00 (m, 2H), 7.04 (t, 1H, *J* = 8.3 Hz), 6.01 (br s, 1H), 4.33 (d, 1H, *J* = 4.7 Hz), 2.39–2.27 (m, 1H), 1.32 (s, 9H), 1.10 (d, 3H, *J* = 6.8 Hz), 1.08 (d, 3H, *J* = 6.8 Hz); ^{13}C-NMR (75 MHz, $CDCl_3$, 25 °C): δ = 168.4, 151.76 (d, $J_{C\text{-}F}$ = 254.3 Hz), 151.75 (d, $J_{C\text{-}F}$ = 10.6 Hz), 142.0 (d, $J_{C\text{-}F}$ = 7.3 Hz), 121.0 (d, $J_{C\text{-}F}$ = 3.3 Hz), 115.4, 112.9 (d, $J_{C\text{-}F}$ = 23.5 Hz), 86.6, 51.6, 31.9, 28.7, 18.8, 17.3; ^{19}F-NMR (282 MHz, $CDCl_3$, 25 °C): δ = –129.4; IR (Neat): ν = 3275, 3074, 2971, 2919, 1646, 1614, 1602, 1551, 1525, 1506, 1467, 1391, 1363, 1343, 1333, 1299, 1276, 1247, 1216, 1148, 1138, 1074, 1022 cm^{-1}; HRMS (ESI$^+$; MeCN/CH_2Cl_2): *m/z* calculated for $C_{15}H_{22}FN_2O_4$ 313.1564, found 313.1555.

N-Cyclohexyl-2-(2-fluoro-4-nitrophenoxy)-3,3-dimethylbutanamide (**4n**). Compound **4n** was prepared according to the general procedure B. Purification on a column of silica gel with ethyl acetate in petroleum ether (90/10) as eluent gave the desired product (351 mg, 50%) as a yellow solid. R_f = 0.6 (petroleum ether/ethyl acetate 80/20); mp 148–149 °C; ^{1}H-NMR (500 MHz, $CDCl_3$, 25 °C): δ = 8.06–7.98 (m, 2H), 7.02 (m, 1H), 5.90 (br d, 1H, *J* = 8.5 Hz), 4.26 (s, 1H), 3.84–3.75 (m, 1H), 1.95–1.88 (m, 1H), 1.72–1.58 (m, 3H), 1.41–1.25 (m, 3H), 1.17–1.10 (m, 2H), 1.13 (s, 9H), 1.00–0.91 (m, 1H); ^{13}C-NMR (75 MHz, $CDCl_3$, 25 °C): δ = 167.2, 151.7 (d, $J_{C\text{-}F}$ = 10.4 Hz), 151.6 (d, $J_{C\text{-}F}$ = 251.4 Hz), 141.8 (d, $J_{C\text{-}F}$ = 7.0 Hz), 121.0 (d, $J_{C\text{-}F}$ = 3.5 Hz), 114.9, 112.8 (d, $J_{C\text{-}F}$ = 22.8 Hz), 88.8, 48.1, 35.1, 33.3, 32.9, 26.3, 25.4, 24.8, 24.7; ^{19}F-NMR (282 MHz, $CDCl_3$, 25 °C): δ = –129.6; IR (Neat): ν = 3281, 3093, 2930, 2856, 1732, 1647, 1618, 1603, 1556, 1526, 1508, 1481, 1450, 1396, 1363, 1347, 1283, 1252, 1240, 1214, 1197, 1152, 1138, 1094, 1076, 1059, 1017 cm^{-1}; HRMS (ESI$^+$; MeCN/CH_2Cl_2): *m/z* calculated for $C_{18}H_{26}FN_2O_4$ 353.1877, found 353.1872.

2-Isopropyl-2H-benzo[b][1,4]oxazin-3(4H)-one (**5a**). Compound **5a** was prepared according to the general procedure C. Purification on a column of silica gel with a gradient of ethyl acetate in petroleum ether (from 90/10 to 80/20) as eluent gave the desired product (76 mg, 79%) as a white solid. R_f = 0.5 (petroleum ether/ethyl acetate 80/20); mp 113–114 °C; ^{1}H-NMR (300 MHz, $CDCl_3$, 25 °C): δ = 8.56 (br s, 1H), 7.00–6.87 (m, 3H), 6.81–6.75 (m, 1H), 4.35 (d, 1H, *J* = 5.9 Hz), 2.30 (sept d, 1H, *J* = 6.8, 5.9 Hz), 1.11 (d, 3H, *J* = 6.8 Hz), 1.04 (d, 3H, *J* = 6.8 Hz); ^{13}C-NMR (75 MHz, $CDCl_3$, 25 °C): δ = 167.5, 143.4, 126.2, 124.2, 122.3, 116.9, 115.6, 81.8, 29.8, 18.7, 17.5; IR (Neat): ν = 3187, 3134, 3072, 2965, 2924, 2869, 1673, 1604, 1500, 1466, 1436, 1387, 1366, 1307, 1275, 1262, 1205, 1161, 1134, 1112, 1039, 1026; HRMS (ESI$^+$; MeCN/CH_2Cl_2): *m/z* calculated for $C_{11}H_{14}NO_2$ 192.1025, found 192.1025.

2-Phenethyl-2H-benzo[b][1,4]oxazin-3(4H)-one (**5b**). Compound **5b** was prepared according to the general procedure C. Purification on a column of silica gel with a gradient of ethyl acetate in petroleum ether (from 95/5 to 80/20) as eluent gave the desired product (113 mg, 89%) as an off-white solid. R_f = 0.3 (petroleum ether/ethyl acetate 80/20); mp 135–136 °C; ^{1}H-NMR (300 MHz, $CDCl_3$, 25 °C): δ = 9.20 (br s, 1H), 7.34–7.15 (m, 5H), 7.04–6.91 (m, 3H), 6.88–6.79 (m, 1H), 4.55 (dd, 1H, *J* = 8.7, 4.5 Hz), 3.01–2.76 (m, 2H), 2.36–2.11 (m, 2H); ^{13}C-NMR (75 MHz, $CDCl_3$, 25 °C): δ = 168.2, 143.0, 140.8, 128.7, 128.6, 126.5, 126.2, 124.3, 122.7, 117.2, 115.8, 76.0, 32.0, 31.1; IR (Neat): ν = 3200, 3135, 3060, 3024, 2991, 2925, 2855, 1676, 1612, 1553, 1519, 1500, 1453, 1435, 1402, 1361, 1316, 1267, 1229, 1179, 1115, 1091, 1081, 1042, 1031; HRMS (ESI$^+$; MeCN/CH_2Cl_2): *m/z* calculated for $C_{16}H_{16}NO_2$ 254.1181, found 254.1191.

2-Isopropyl-6-methoxy-2H-benzo[b][1,4]oxazin-3(4H)-one (**5c**). Compound **5c** was prepared according to the general procedure C. Purification on a column of silica gel with petroleum ether/ethyl acetate 80/20 as eluent gave the desired product (72 mg, 65%) as an off-white solid. R_f = 0.3 (petroleum ether/ethyl acetate 80/20); mp 127–128 °C; ^{1}H-NMR (300 MHz, $CDCl_3$, 25 °C): δ = 8.68 (br s, 1H), 6.89 (d, 1H, *J* = 8.8 Hz), 6.50 (dd, 1H, *J* = 8.8, 2.8 Hz), 6.37 (d, 1H, *J* = 2.8 Hz), 4.26 (d, 1H, *J* = 6.3 Hz), 3.76 (s,

3H), 2.26 (oct, 1H, *J* = 6.8 Hz), 1.09 (d, 3H, *J* = 6.8 Hz), 1.04 (d, 3H, *J* = 6.8 Hz); ^{13}C-NMR (75 MHz, $CDCl_3$, 25 °C): *δ* = 168.4, 155.1, 137.2, 126.9, 117.2, 108.7, 102.0, 81.7, 55.9, 29.4, 18.7, 17.6; IR (Neat): *ν* = 3064, 2965, 2906, 1686, 1625, 1610, 1523, 1501, 1463, 1394, 1366, 1329, 1298, 1261, 1218, 1197, 1169, 1140, 1129, 1039, 1016; HRMS (ESI$^+$; MeCN/CH_2Cl_2): *m/z* calculated for $C_{12}H_{16}NO_3$ 222.1130, found 222.1137.

6-Chloro-2-ethyl-2H-benzo[b][1,4]oxazin-3(4H)-one (**5d**). Compound **5d** was prepared according to the general procedure C. Purification on a column of silica gel with a gradient of ethyl acetate in petroleum ether (from 95/5 to 90/10) as eluent gave the desired product (80 mg, 76%) as a white solid. R_f = 0.4 (petroleum ether/ethyl acetate 80/20); mp 155–156 °C; ^{1}H-NMR (300 MHz, $CDCl_3$, 25 °C): *δ* = 9.10 (br s, 1H), 6.94 (dd, 1H, *J* = 8.5, 2.0 Hz), 6.90 (d, 1H, *J* = 8.5 Hz), 6.83 (d, 1H, *J* = 2.0 Hz), 4.50 (dd, 1H, *J* = 8.1, 4.6 Hz), 2.05–1.81 (m, 2H), 1.09 (t, 3H, *J* = 7.4 Hz); ^{13}C-NMR (75 MHz, $CDCl_3$, 25 °C): *δ* = 168.2, 141.7, 127.4 (2C), 124.0, 118.2, 115.7, 78.2, 24.0, 9.5; IR (Neat): *ν* = 3181, 3086, 2965, 2877, 1675, 1602, 1493, 1463, 1454, 1400, 1378, 1354, 1330, 1303, 1292, 1273, 1259, 1241, 1227, 1144, 1112, 1084, 1054, 1014; HRMS (ESI$^+$; MeCN/CH_2Cl_2): *m/z* calculated for $C_{12}H_{14}ClN_2O_2$ 253.0744, found 253.0756.

2-(4-Methoxyphenyl)-6-methyl-2H-benzo[b][1,4]oxazin-3(4H)-one (**5e**). Compound **5e** was prepared according to the general procedure C. Purification on a column of silica gel with petroleum ether/ethyl acetate 80/20 as eluent gave the desired product (53 mg, 39%) as an off-white solid. R_f = 0.2 (petroleum ether/ethyl acetate 80/20); mp 189–190 °C; ^{1}H-NMR (300 MHz, $CDCl_3$, 25 °C): *δ* = 8.27 (br s, 1H), 7.38–7.32 (m, 2H), 6.91–6.84 (m, 3H), 6.76 (dd, 1H, *J* = 8.2, 1.8 Hz), 6.60 (d, 1H, *J* = 1.8 Hz), 5.60 (s, 1H), 3.78 (s, 3H), 2.26 (s, 3H); ^{13}C-NMR (75 MHz, $CDCl_3$, 25 °C): *δ* = 166.5, 160.1, 140.8, 132.5, 128.6, 127.3, 125.8, 124.9, 117.2, 116.3, 114.2, 78.5, 55.4, 20.8; IR (Neat): *ν* = 3000, 1683, 1607, 1515, 1497, 1450, 1392, 1350, 1302, 1246, 1221, 1205, 1175, 1130, 1111, 1031; HRMS (ESI$^+$; MeCN/CH_2Cl_2): *m/z* calculated for $C_{16}H_{16}NO_3$ 270.1130, found 270.1133.

2-(3-Fluorophenyl)-2H-benzo[b][1,4]oxazin-3(4H)-one (**5f**). Compound **5f** was prepared according to the general procedure C. Purification on a column of silica gel with petroleum ether/ethyl acetate 80/20 as eluent gave the desired product (114 mg, 94%) as a white solid. R_f = 0.3 (petroleum ether/ethyl acetate 80/20); mp 153–154 °C; ^{1}H-NMR (300 MHz, $CDCl_3$, 25 °C): *δ* = 8.67 (br s, 1H), 7.33 (td, 1H, *J* = 7.8, 5.6 Hz), 7.28–7.23 (m, 1H), 7.22–7.16 (m, 1H), 7.09–6.93 (m, 4H), 6.84–6.77 (m, 1H), 5.69 (s, 1H); ^{13}C-NMR (125 MHz, $CDCl_3$, 25 °C): *δ* = 165.5, 163.0 (d, $J_{C\text{-}F}$ = 246.5 Hz), 142.8, 137.5 (d, $J_{C\text{-}F}$ = 7.4 Hz), 130.4 (d, $J_{C\text{-}F}$ = 8.2 Hz), 125.8, 124.7, 123.1, 122.7 (d, $J_{C\text{-}F}$ = 2.8 Hz), 117.5, 116.02, 116.00 (d, $J_{C\text{-}F}$ = 21.1 Hz), 114.2 (d, $J_{C\text{-}F}$ = 21.1 Hz), 78.0; ^{19}F-NMR (282 MHz, $CDCl_3$, 25 °C): *δ* = –112.0; IR (Neat): *ν* = 3188, 3132, 3065, 2982, 2956, 2915, 1680, 1607, 1498, 1451, 1435, 1393, 1352, 1307, 1268, 1257, 1215, 1143, 1109, 1052, 1035, 1025; HRMS (ESI$^+$; MeCN/CH_2Cl_2): *m/z* calculated for $C_{14}H_{11}FNO_2$ 244.0774, found 244.0773.

2-(Methoxymethyl)-2-methyl-2H-benzo[b][1,4]oxazin-3(4H)-one (**5g**). Compound **5g** was prepared according to the general procedure C. Purification on a column of silica gel with petroleum ether/ethyl acetate 80/20 as eluent gave the desired product (81 mg, 78%) as an off-white solid. R_f = 0.2 (petroleum ether/ethyl acetate 80/20); mp 81–82 °C; ^{1}H-NMR (300 MHz, $CDCl_3$, 25 °C): *δ* = 9.26 (br s, 1H), 6.99–6.88 (m, 3H), 6.83–6.79 (m, 1H), 3.90 (d, 1H, *J* = 10.4 Hz), 3.64 (d, 1H, *J* = 10.4 Hz), 3.42 (s, 3H), 1.47 (s, 3H); ^{13}C-NMR (75 MHz, $CDCl_3$, 25 °C): *δ* = 168.8, 142.5, 126.1, 124.2, 122.3, 117.3, 115.5, 80.9, 75.7, 60.1, 19.5; IR (Neat): *ν* = 3204, 3140, 3077, 2996, 2902, 1675, 1608, 1502, 1472, 1451, 1432, 1391, 1375, 1308, 1285, 1273, 1257, 1214, 1199, 1160, 1143, 1115, 1033; HRMS (ESI$^+$; MeCN/CH_2Cl_2): *m/z* calculated for $C_{11}H_{14}NO_3$ 208.0974, found 208.0977.

6-Bromospiro[benzo[b][1,4]oxazine-2,1′-cyclobutan]-3(4H)-one (**5h**). Compound **5h** was prepared according to the general procedure C. Purification on a column of silica gel with a gradient of ethyl acetate in petroleum ether (from 95/5 to 80/20) as eluent gave the desired product (59 mg, 44%) as a white solid. R_f = 0.6 (petroleum ether/ethyl acetate 80/20); mp 226–227 °C; ^{1}H-NMR (300 MHz, $CDCl_3$, 25 °C): *δ* = 8.25 (br s, 1H), 7.07 (dd, 1H, *J* = 8.5, 2.3 Hz), 6.94 (d, 1H, *J* = 2.3 Hz), 6.88 (d, 1H, *J* = 8.5 Hz), 2.71–2.57 (m, 2H), 2.40–2.27 (m, 2H), 2.07–1.90 (m, 2H); ^{13}C-NMR (125 MHz, $CDCl_3$, 25 °C): *δ* = 168.8, 141.9, 128.3, 126.7, 119.2, 118.1, 114.8, 80.0, 31.6, 13.3; IR (Neat): *ν* = 3121, 3078, 3037, 3000, 2948, 2870,

1697, 1602, 1493, 1445, 1411, 1381, 1306, 1279, 1261, 1238, 1213, 1153, 1139, 1112, 1066, 1051; HRMS (ESI+; MeCN/CH_2Cl_2): *m/z* calculated for $C_{13}H_{14}BrN_2O_2$ 309.0239, found 309.0233.

6-Chlorospiro[benzo[b][1,4]oxazine-2,1′-cyclobutan]-3(4H)-one (**5i**). Compound **5i** was prepared according to the general procedure C. Purification on a column of silica gel with a gradient of ethyl acetate in petroleum ether (from 95/5 to 80/20) as eluent gave the desired product (84 mg, 75%) as a white solid. R_f = 0.6 (petroleum ether/ethyl acetate 80/20); mp 225–226 °C; ^{1}H-NMR (500 MHz, $CDCl_3$, 25 °C): δ = 7.88 (br s, 1H), 6.95 (d, 1H, *J* = 8.4 Hz), 6.92 (dd, 1H, *J* = 8.4, 2.0 Hz), 6.78 (d, 1H, *J* = 2.0 Hz), 2.71–2.58 (m, 2H), 2.40–2.26 (m, 2H), 2.06–1.91 (m, 2H); ^{13}C-NMR (125 MHz, $CDCl_3$, 25 °C): δ = 168.9, 141.3, 127.9, 127.7, 123.7, 118.7, 115.4, 80.0, 31.6, 13.3; IR (Neat): ν = 3082, 3042, 3003, 2962, 2874, 1693, 1607, 1495, 1442, 1412, 1379, 1307, 1282, 1261, 1240, 1213, 1155, 1138, 1110, 1077, 1052; HRMS (ESI+; MeCN/CH_2Cl_2): *m/z* calculated for $C_{13}H_{14}ClN_2O_2$ 265.0744, found 265.0757.

2-Methyl-2-(trifluoromethyl)-2H-benzo[b][1,4]oxazin-3(4H)-one (**5j**). Compound **5j** was prepared according to the general procedure C. Purification on a column of silica gel with petroleum ether/ethyl acetate 90/10 as eluent gave the desired product (90 mg, 78%) as a white solid. R_f = 0.5 (petroleum ether/ethyl acetate 80/20); mp 143–144 °C; ^{1}H-NMR (500 MHz, $CDCl_3$, 25 °C): δ = 8.45 (br s, 1H), 7.06–6.97 (m, 3H), 6.81 (d, 1H, *J* = 7.8 Hz), 1.80 (s, 3H); ^{13}C-NMR (75 MHz, $CDCl_3$, 25 °C): δ = 163.0, 141.5, 125.0, 124.9, 123.5 (q, J_{C-F} = 287.3 Hz), 123.3, 116.8, 115.9, 79.2 (q, J_{C-F} = 28.9 Hz), 18.3; ^{19}F-NMR (282 MHz, $CDCl_3$, 25 °C): δ = –77.9; IR (Neat): ν = 3064, 2965, 2906, 1687, 1625, 1610, 1523, 1501, 1463, 1450, 1393, 1366, 1329, 1298, 1276, 1261, 1218, 1197, 1169, 1141, 1129, 1108, 1039, 1016; HRMS (ESI+; MeCN/CH_2Cl_2): *m/z* calculated for $C_{12}H_{12}F_3N_2O_2$ 273.0851, found 273.0850.

4-Cyclohexyl-2-isopropyl-2H-benzo[b][1,4]oxazin-3(4H)-one (**6a**). Compound **6a** was prepared according to the general procedure D. Purification on a column of silica gel with ethyl acetate in petroleum ether (98/2) as eluent gave the desired product (73 mg, 89%) as a yellow oil. R_f = 0.6 (petroleum ether/ethyl acetate 95/5); ^{1}H-NMR (500 MHz, $CDCl_3$, 25 °C): δ = 7.17–7.10 (m, 1H), 7.02–6.94 (m, 3H), 4.20 (tt, 1H, *J* = 12.2, 3.8 Hz), 4.15 (d, 1H, *J* = 6.7 Hz), 2.45–2.32 (m, 2H), 2.21–2.10 (m, 1H), 1.93–1.66 (m, 5H), 1.44–1.22 (m, 3H), 1.04 (d, 3H, *J* = 6.8 Hz), 0.99 (d, 3H, *J* = 6.8 Hz); ^{13}C-NMR (75 MHz, $CDCl_3$, 25 °C): δ = 167.1, 145.6, 129.7, 123.8, 122.2, 117.7, 115.9, 82.8, 56.8, 29.8, 29.3, 28.9, 26.6, 26.5, 25.5, 18.7, 17.8. IR (Neat): (cm^{-1}) 2931, 2854, 1675, 1605, 1496, 1464, 1411, 1361, 1320, 1298, 1262, 1245, 1125, 1042, 1015; HRMS (ESI+; MeCN/CH_2Cl_2): *m/z* calculated for $C_{17}H_{23}NO_2$ 274.1807, found 274.1797.

7-Chloro-4-cyclohexylspiro[benzo[b][1,4]oxazine-2,1′-cyclobutan]-3(4H)-one (**6b**). Compound **6b** was prepared according to the general procedure D. Purification on a column of silica gel with ethyl acetate in petroleum ether (98/2) as eluent gave the desired product (45 mg, 91%) as a yellow solid. R_f = 0.3 (petroleum ether/ethyl acetate 99/1); mp 77–78 °C; ^{1}H-NMR (500 MHz, $CDCl_3$, 25 °C): δ = 7.04 (d, 1H, *J* = 8.6 Hz), 7.02 (d, 1H, *J* = 2.4 Hz), 6.96 (dd, 1H, *J* = 8.6, 2.4 Hz), 4.17 (tt, 1H, *J* = 12.5, 3.7 Hz), 2.60–2.46 (m, 2H), 2.38–2.15 (m, 4H), 2.02–1.82 (m, 4H), 1.80–1.65 (m, 3H), 1.42–1.12 (m, 3H); ^{13}C-NMR (75 MHz, $CDCl_3$, 25 °C): δ = 168.6, 145.4, 128.9, 128.3, 122.5, 118.7, 116.7, 80.4, 56.8, 30.9, 29.5, 26.5, 25.5, 13.2; IR (Neat): ν = 2932, 2855, 1678, 1581, 1491, 1452, 1424, 1405, 1360, 1336, 1270, 1245, 1147, 1118, 1081, 1045; HRMS (ESI+; MeCN/CH_2Cl_2): *m/z* calculated for $C_{17}H_{20}ClNO_2$ 306.1261, found 306.1259.

2-Isobutyl-4-phenethyl-7-(trifluoromethyl)-2H-benzo[b][1,4]oxazin-3(4H)-one (**6c**). Compound **6c** was prepared according to the general procedure D. Purification on a column of silica gel with ethyl acetate in petroleum ether (95/5) as eluent gave the desired product (80 mg, 71%) as a yellow oil. R_f = 0.4 (petroleum ether/ethyl acetate 95/5); ^{1}H-NMR (500 MHz, $CDCl_3$, 25 °C): δ = 7.34–7.29 (m, 2H), 7.29–7.26 (m, 1H), 7.26–7.21 (m, 4H), 6.99 (d, 1H, *J* = 8.4 Hz), 4.63 (dd, 1H, *J* = 9.8, 4.3 Hz), 4.19 (dt, 1H, *J* = 14.0, 7.9 Hz), 4.11 (dt, 1H, *J* = 14.0, 7.9 Hz), 2.95 (t, 2H, *J* = 7.9 Hz), 1.96–1.87 (m, 1H), 1.70 (ddd, 1H, *J* = 14.7, 9.8, 5.4 Hz), 1.70 (ddd, 1H, *J* = 14.7, 8.7, 4.3 Hz), 0.99 (d, 3H, *J* = 6.6 Hz), 0.98 (d, 3H, *J* = 6.6 Hz); ^{13}C-NMR (125 MHz, $CDCl_3$, 25 °C): δ = 166.2, 144.1, 137.9, 131.7, 128.9, 128.8, 127.0, 126.1 (q, J_{C-F} = 33.7 Hz), 123.8 (q, J_{C-F} = 271.1 Hz), 119.8 (q, J_{C-F} = 3.6 Hz), 115.0 (q, J_{C-F} = 3.6 Hz), 114.5, 75.9, 43.2, 38.9, 33.5, 24.5, 23.2, 21.7; ^{19}F-NMR (282 MHz, $CDCl_3$, 25 °C): δ = –62.1; IR (Neat): ν = 2959, 2872, 1689, 1622, 1519,

1498, 1444, 1394, 1369, 1325, 1303, 1260, 1235, 1169, 1146, 1118, 1076 cm^{-1}; HRMS (ESI$^+$; MeCN/CH_2Cl_2): *m/z* calculated for $C_{21}H_{23}F_3NO_2$ 378.1681, found 378.1677.

4-Cyclohexyl-2-methyl-2-(trifluoromethyl)-2H-benzo[b][1,4]oxazin-3(4H)-one (**6d**). Compound **6d** was prepared according to the general procedure D. Purification on a column of silica gel with a gradient of ethyl acetate in petroleum ether (from 99/1 to 98/2) as eluent gave the desired product (76 mg, 81%) as a yellow oil. R_f = 0.5 (petroleum ether/ethyl acetate 95/5); ^{1}H-NMR (500 MHz, $CDCl_3$, 25 °C): δ = 7.17–7.12 (m, 1H), 7.08–6.99 (m, 3H), 4.18 (tt, 1H, *J* = 12.3, 3.7 Hz), 2.49–2.32 (m, 2H), 1.94–1.87 (m, 2H), 1.84–1.76 (m, 2H), 1.75–1.68 (m, 1H), 1.70 (s, 3H), 1.44–1.32 (m, 2H), 1.32–1.03 (m, 1H); ^{13}C-NMR (125 MHz, $CDCl_3$, 25 °C): δ = 162.8, 143.1, 128.6, 124.4, 123.7 (q, $J_{C\text{-}F}$ = 288.4 Hz), 123.2, 117.7, 115.6, 79.5 (q, $J_{C\text{-}F}$ = 28.1 Hz), 57.9, 29.2, 28.9, 26.5, 26.4, 25.4, 18.3; ^{19}F-NMR (282 MHz, $CDCl_3$, 25 °C): δ = –77.2; IR (Neat): ν = 2934, 2857, 1685, 1611, 1592, 1498, 1453, 1413, 1379, 1361, 1324, 1299, 1274, 1248, 1191, 1148, 1102, 1044; HRMS (ESI$^+$; MeCN/CH_2Cl_2): *m/z* calculated for $C_{16}H_{18}F_3NO_2$ 314.1368, found 314.1360.

2-Ethyl-4-(4-methoxybenzyl)-3-oxo-3,4-dihydro-2H-benzo[b][1,4]oxazine-7-carbonitrile (**6e**). Compound **6e** was prepared according to the general procedure D. Purification on a column of silica gel with a gradient of ethyl acetate in petroleum ether (from 90/10 to 80/20) as eluent gave the desired product (72 mg, 74%) as a white solid. R_f = 0.6 (petroleum ether/ethyl acetate 70/30); mp 138–139 °C; ^{1}H-NMR (500 MHz, $CDCl_3$, 25 °C): δ = 7.26–7.24 (m, 1H), 7.20 (dd, 1H, *J* = 8.4, 1.8 Hz), 7.14 (d, 2H, *J* = 8.6 Hz), 6.94 (d, 1H, *J* = 8.4 Hz), 6.86 (d, 2H, *J* = 8.6 Hz), 5.17 (d, 1H, *J* = 15.9 Hz), 5.02 (d, 1H, *J* = 15.9 Hz), 4.64 (dd, 1H, *J* = 8.5, 4.5 Hz), 3.78 (s, 3H), 2.08–1.97 (m, 1H), 1.92 (dquint, 1H, *J* = 14.9, 7.3 Hz), 1.12 (t, 3H, *J* = 7.4 Hz); ^{13}C-NMR (125 MHz, $CDCl_3$, 25 °C): δ = 166.2, 159.3, 144.3, 133.1, 128.0, 127.2, 127.0, 120.7, 118.3, 115.9, 114.6, 107.1, 78.5, 55.4, 44.8, 24.2, 9.5; IR (Neat): ν = 3063, 2964, 2934, 2836, 2226, 1687, 1610, 1587, 1509, 1463, 1446, 1395, 1333, 1292, 1280, 1246, 1190, 1177, 1142, 1118, 1097, 1073, 1047, 1028; HRMS (ESI$^+$; MeCN/CH_2Cl_2): *m/z* calculated for $C_{19}H_{18}N_2O_3$ 323.1396, found 323.1380.

7-Bromo-4-(3,4-dimethoxyphenethyl)-2-isopropyl-2H-benzo[b][1,4]oxazin-3(4H)-one (**6f**). Compound **6f** was prepared according to the general procedure D. Purification on a column of silica gel with ethyl acetate in petroleum ether (80/20) as eluent gave the desired product (91 mg, 70%) as a yellow oil. R_f = 0.4 (petroleum ether/ethyl acetate 80/20); ^{1}H-NMR (500 MHz, $CDCl_3$, 25 °C): δ = 7.15 (d, 1H, *J* = 2.1 Hz), 7.11 (dd, 1H, *J* = 8.7, 2.1 Hz), 6.80 (d, 1H, *J* = 8.0 Hz), 6.78 (d, 1H, *J* = 8.7 Hz), 6.75 (dd, 1H, *J* = 8.0, 2.0 Hz), 6.71 (d, 1H, *J* = 2.0 Hz), 4.26 (d, 1H, *J* = 6.4 Hz), 4.18 (dt, 1H, *J* = 14.1, 7.8 Hz), 4.03 (dt, 1H, *J* = 14.1, 7.8 Hz), 3.86 (s, 3H), 3.85 (s, 3H), 2.87 (t, 2H, *J* = 7.0 Hz), 2.20 (oct, 1H, *J* = 6.8 Hz), 1.04 (d, 3H, *J* = 6.8 Hz), 0.96 (d, 3H, *J* = 6.8 Hz); ^{13}C-NMR (125 MHz, $CDCl_3$, 25 °C): δ = 164.9, 149.2, 148.0, 145.5, 130.5, 127.9, 125.3, 120.9, 120.6, 115.8, 115.7, 112.2, 111.6, 82.0, 56.1, 56.0, 43.0, 33.0, 29.6, 18.7, 17.5; IR (Neat): ν = 2963, 2934, 2835, 1678, 1590, 1515, 1493, 1463, 1420, 1388, 1323, 1260, 1235, 1182, 1156, 1140, 1075, 1027; HRMS (ESI$^+$; MeCN/CH_2Cl_2): *m/z* calculated for $C_{21}H_{24}BrNO_4$ 434.0967, found 434.0975.

4-(4-Methoxybenzyl)spiro[benzo[b][1,4]oxazine-2,1′-cyclobutan]-3(4H)-one (**6g**). Compound **6g** was prepared according to the general procedure D. Purification on a column of silica gel with ethyl acetate in petroleum ether (95/5) as eluent gave the desired product (79 mg, 86%) as a yellow oil. R_f = 0.3 (petroleum ether/ethyl acetate 95/5); ^{1}H-NMR (500 MHz, $CDCl_3$, 25 °C): δ = 7.18 (d, 2H, *J* = 8.6 Hz), 7.01 (dd, 1H, *J* = 7.7, 1.1 Hz), 6.95 (td, 1H, *J* = 7.9, 1.5 Hz), 6.92–6.86 (m, 2H), 6.85 (d, 2H, *J* = 8.7 Hz), 5.08 (s, 2H), 3.77 (s, 3H), 2.72–2.64 (m, 2H), 2.39–2.31 (m, 2H), 2.07–1.94 (m, 2H); ^{13}C-NMR (125 MHz, $CDCl_3$, 25 °C): δ = 167.8, 159.0, 143.7, 129.5, 128.6, 128.0, 123.7, 122.7, 117.9, 115.3, 114.4, 79.7, 55.3, 44.8, 31.5, 13.3; IR (Neat): ν = 2954, 2835, 1675, 1609, 1587, 1512, 1498, 1464, 1387, 1331, 1303, 1243, 1175, 1148, 1105, 1032, 1011; HRMS (ESI$^+$; MeCN/CH_2Cl_2): *m/z* calculated for $C_{19}H_{19}NO_3$ 310.1443, found 310.1436.

4-(Tert-butyl)-7-chloro-2-isopropyl-2*H*-benzo[*b*][1,4]oxazin-3(4*H*)-one (**6h**). Compound **6h** was prepared according to the general procedure D. Purification on a column of silica gel with ethyl acetate in petroleum ether (95/5) as eluent gave the desired product (79 mg, 94%) as a yellow oil. R_f = 0.5 (petroleum ether/ethyl acetate 95/5); ^{1}H-NMR (500 MHz, $CDCl_3$, 25 °C): δ = 7.05 (d, 1H, *J* = 8.7 Hz),

7.03 (d, 1H, *J* = 2.5 Hz), 6.95 (dd, 1H, *J* = 8.7, 2.5 Hz), 4.03 (d, 1H, *J* = 4.5 Hz), 2.36–2.25 (m, 1H), 1.62 (s, 9H), 1.09 (d, 3H, *J* = 6.9 Hz), 0.97 (d, 3H, *J* = 6.9 Hz); ^{13}C-NMR (125 MHz, $CDCl_3$, 25 °C): δ = 172.6, 149.7, 129.4, 128.9, 121.7, 118.1, 85.5, 59.2, 29.9, 29.7, 19.2, 16.9; IR (Neat): ν = 2969, 2936, 1683, 1579, 1489, 1466, 1419, 1398, 1367, 1347, 1324, 1292, 1269, 1247, 1191, 1136, 1079, 1020; HRMS (ESI$^+$; MeCN/CH_2Cl_2): *m*/*z* calculated for $C_{15}H_{20}ClNO_2$ 282.1261, found 282.1263.

4-(3,4-Dimethoxyphenethyl)-2-(2,6-dimethylhept-5-en-1-yl)-7-(trifluoromethyl)-2H-benzo[b][1,4]oxazin-3(4H)-one (**6i**). Compound **6i** was prepared according to the general procedure D. Purification on a column of silica gel with ethyl acetate in petroleum ether (90/10) as eluent gave the desired product (108 mg, 71%) as a 1:1 mixture of unseparable diastereomers (yellow oil). R_f = 0.2 (petroleum ether/ethyl acetate 90/10); ^{1}H-NMR (500 MHz, $CDCl_3$, 25 °C): δ = 7.31–7.27 (m, 1H), 7.23 (d, 0.5H, *J* = 2.1 Hz), 7.22 (d, 0.5H, *J* = 2.1 Hz), 7.02 (d, 0.5H, *J* = 8.5 Hz), 7.01 (d, 0.5H, *J* = 8.5 Hz), 6.80 (d, 1H, *J* = 8.0 Hz), 6.75 (br d, 1H, *J* = 8.0 Hz), 6.73–6.70 (s, 1H), 5.09 (t, 0.5H, *J* = 7.0 Hz), 5.05 (t, 0.5H, *J* = 7.0 Hz), 4.67–4.61 (m, 1H), 4.21–4.05 (m, 2H), 3.86 (s, 3H), 3.85 (s, 3H), 2.88 (br t, 2H, *J* = 7.3 Hz), 2.09–1.87 (m, 2H), 1.84–1.73 (m, 2H), 1.71–1.51 (m, 1H), 1.68 (s, 1.5H), 1.66 (s, 1.5H), 1.60 (s, 1.5H), 1.55 (s, 1.5H), 1.51–1.13 (m, 2H), 0.99 (d, 1.5H, *J* = 6.5 Hz), 0.98 (d, 1.5H, *J* = 6.5 Hz); ^{13}C-NMR (75 MHz, $CDCl_3$, 25 °C): δ = 166.4, 166.2, 149.1, 148.0, 144.2, 144.0, 131.7, 131.6, 130.3, 126.5 (q, $J_{C\text{-}F}$ = 33.0 Hz), 124.5, 124.3, 123.8 (q, $J_{C\text{-}F}$ = 268.9 Hz), 120.9, 119.8 (q, $J_{C\text{-}F}$ = 3.8 Hz), 115.0 (q, $J_{C\text{-}F}$ = 3.4 Hz), 114.6, 112.1, 111.4, 76.0, 75.4, 56.0, 43.2, 43.1, 37.5, 37.3, 36.9, 36.1, 33.0, 29.2, 28.4, 25.8, 25.7, 25.4, 25.3, 20.0, 18.8, 17.8, 17.7; ^{19}F-NMR (282 MHz, $CDCl_3$, 25 °C): δ = –62.1; IR (Neat): ν = 2926, 1689, 1622, 1591, 1516, 1443, 1395, 1325, 1305, 1261, 1237, 1142, 1119, 1074, 1028; HRMS (ESI$^+$; MeCN/CH_2Cl_2): *m*/*z* calculated for $C_{28}H_{34}F_3NO_4$ 506.2518, found 506.2533.

4-(4-Chlorobenzyl)-2-isobutyl-7-methyl-2H-benzo[b][1,4]oxazin-3(4H)-one (**6j**). Compound **6j** was prepared according to the general procedure D. Purification on a column of silica gel with a gradient of ethyl acetate in petroleum ether (from 98/2 to 95/5) as eluent gave the desired product (69 mg, 67%) as a yellow solid. R_f = 0.4 (petroleum ether/ethyl acetate 95/5); mp 77–78 °C; ^{1}H-NMR (500 MHz, $CDCl_3$, 25 °C): δ = 7.28 (d, 2H, *J* = 8.3 Hz), 7.16 (d, 2H, *J* = 8.3 Hz), 6.81 (br s, 1H), 6.70 (br d, 1H, *J* = 8.4 Hz), 6.67 (d, 1H, *J* = 8.4 Hz), 5.16 (d, 1H, *J* = 16.2 Hz), 4.99 (d, 1H, *J* = 16.2 Hz), 4.70 (dd, 1H, *J* = 10.2, 3.9 Hz), 2.26 (s, 3H), 2.03–1.90 (m, 1H), 1.81 (ddd, 1H, *J* = 14.4, 10.2, 5.1 Hz), 1.70 (ddd, 1H, *J* = 14.2, 8.9, 3.9 Hz), 1.01 (d, 3H, *J* = 6.9 Hz), 0.99 (d, 3H, *J* = 6.9 Hz); ^{13}C-NMR (125 MHz, $CDCl_3$, 25 °C): δ = 167.0, 143.8, 135.0, 134.3, 133.3, 129.1, 128.1, 126.3, 123.1, 118.3, 115.0, 75.9, 44.6, 38.8, 24.6, 23.3, 21.7, 20.8; IR (Neat): ν = 2957, 2870, 1675, 1511, 1490, 1469, 1430, 1397, 1368, 1317, 1294, 1260, 1207, 1156, 1089, 1059, 1030, 1013; HRMS (ESI$^+$; MeCN/CH_2Cl_2): *m*/*z* calculated for $C_{20}H_{22}ClNO_2$ 344.1417, found 344.1417.

2-(Tert-butyl)-7-methoxy-4-phenethyl-2H-benzo[b][1,4]oxazin-3(4H)-one (**6k**). Compound **6k** was prepared according to the general procedure D. Purification on a column of silica gel with ethyl acetate in petroleum ether (95/5) as eluent gave the desired product (98 mg, 96%) as a yellow solid. R_f = 0.2 (petroleum ether/ethyl acetate 95/5); mp 82–83 °C; ^{1}H-NMR (500 MHz, $CDCl_3$, 25 °C): δ = 7.32 (t, 2H, *J* = 7.4 Hz), 7.29–7.26 (m, 2H), 7.25–7.21 (t, 1H, *J* = 7.4 Hz), 6.83 (d, 1H, *J* = 9.0 Hz), 6.58 (d, 1H, *J* = 2.8 Hz), 6.53 (dd, 1H, *J* = 9.0, 2.8 Hz), 4.28 (s, 1H), 4.19 (ddd, 1H, *J* = 13.9, 10.0, 6.0 Hz), 4.04 (ddd, 1H, *J* = 13.9, 10.0, 6.0 Hz), 3.79 (s, 3H), 2.98 (ddd, 1H, *J* = 13.4, 10.0, 6.0 Hz), 2.92 (ddd, 1H, *J* = 13.4, 10.0, 6.0 Hz), 1.02 (s, 9H); ^{13}C-NMR (125 MHz, $CDCl_3$, 25 °C): δ = 163.6, 156.4, 146.4, 138.4, 128.9, 128.7, 126.7, 122.0, 114.7, 107.1, 103.0, 84.4, 55.7, 43.1, 37.0, 33.6, 26.6; IR (Neat): ν = 3029, 2969, 1671, 1624, 1592, 1511, 1476, 1454, 1431, 1397, 1367, 1329, 1306, 1274, 1238, 1194, 1159, 1126, 1072, 1057, 1039, 1022; HRMS (ESI$^+$; MeCN/CH_2Cl_2): *m*/*z* calculated for $C_{21}H_{25}NO_3$ 340.1913, found 340.1915.

4-Cyclohexyl-2-ethyl-2H-benzo[b][1,4]oxazin-3(4H)-one (**6l**). Compound **6l** was prepared according to the general procedure D. Purification on a column of silica gel with petroleum ether/ethyl acetate (98/2) as eluent gave the desired product (62 mg, 80%) as an orange oil. R_f = 0.5 (petroleum ether/ethyl acetate 95/5); ^{1}H-NMR (500 MHz, $CDCl_3$, 25 °C): δ = 7.10–7.04 (m, 1H), 6.97–6.88 (m, 3H), 4.25 (dd, 1H, *J* = 8.6, 4.5 Hz), 4.11 (tt, 1H, *J* = 12.3, 3.7 Hz), 2.38–2.20 (m, 2H), 1.90–1.58 (m, 7H), 1.39–1.10 (m, 3H), 0.98 (t, 3H, *J* = 7.4 Hz); ^{13}C-NMR (125 MHz, $CDCl_3$, 25 °C): δ = 167.9, 145.5, 130.0, 123.8, 122.4, 117.8,

116.0, 79.2, 57.0, 29.6, 29.5, 26.6, 26.5, 25.5 23.4, 9.6; IR (Neat): ν = 2931, 2854, 1676, 1604, 1589, 1496, 1454, 1411, 1363, 1322, 1298, 1269, 1245, 1211, 1191, 1150, 1118, 1049; HRMS (ESI$^+$; MeCN/CH_2Cl_2): *m/z* calculated for $C_{16}H_{21}NO_2$ 260.1651, found 260.1654.

7-Bromo-2-(tert-butyl)-4-phenethyl-2H-benzo[b][1,4]oxazin-3(4H)-one (**6m**). Compound **6m** was prepared according to the general procedure D. Purification on a column of silica gel with ethyl acetate in petroleum ether (95/5) as eluent gave the desired product (110 mg, 95%) as an off-white solid. R_f = 0.3 (petroleum ether/ethyl acetate 95/5); mp 117–118 °C; ^{1}H-NMR (500 MHz, $CDCl_3$, 25 °C): δ = 7.35–7.29 (m, 2H), 7.28–7.21 (m, 3H), 7.14 (d, 1H, *J* = 2.1 Hz), 7.09 (dd, 1H, *J* = 8.6, 2.1 Hz), 6.77 (d, 1H, *J* = 8.6 Hz), 4.29 (s, 1H), 4.20 (ddd, 1H, *J* = 14.0, 9.9, 6.0 Hz), 4.04 (ddd, 1H, *J* = 14.0, 9.9, 6.0 Hz), 2.97 (ddd, 1H, *J* = 13.3, 9.9, 6.0 Hz), 2.91 (ddd, 1H, *J* = 13.3, 9.9, 6.0 Hz), 1.02 (s, 9H); ^{13}C-NMR (125 MHz, $CDCl_3$, 25 °C): δ = 163.7, 146.3, 138.0, 128.9, 128.8, 127.7, 126.9, 124.9, 119.9, 115.9, 115.4, 84.4, 43.1, 37.1, 33.5, 26.6; IR (Neat): ν = 2965, 1667, 1581, 1494, 1463, 1420, 1395, 1370, 1330, 1276, 1229, 1186, 1141, 1056, 1018; HRMS (ESI$^+$; MeCN/CH_2Cl_2): *m/z* calculated for $C_{20}H_{22}BrNO_2$ 388.0912, found 388.0898.

4-(4-Methoxybenzyl)-7-methyl-2-(2-methylpent-4-en-2-yl)-2H-benzo[b][1,4]oxazin-3(4H)-one (**6n**). Compound **6n** was prepared according to the general procedure D. Purification on a column of silica gel with ethyl acetate in petroleum ether (95/5) as eluent gave the desired product (102 mg, 93%) as a yellow oil. R_f = 0.3 (petroleum ether/ethyl acetate 95/5); ^{1}H-NMR (500 MHz, $CDCl_3$, 25 °C): δ = 7.22 (d, 2H, *J* = 8.6 Hz), 6.84 (d, 2H, *J* = 8.6 Hz), 6.78 (d, 1H, *J* = 1.8 Hz), 6.77 (d, 1H, *J* = 8.4 Hz), 6.66 (dd, 1H, *J* = 8.4, 1.8 Hz), 5.93–5.81 (m, 1H), 5.14–5.07 (m, 3H), 5.00 (d, 1H, *J* = 15.7 Hz), 4.45 (s, 1H), 3.77 (s, 3H), 2.32 (dd, 1H, *J* = 14.0, 7.7 Hz), 2.25 (s, 3H), 2.20 (dd, 1H, *J* = 14.0, 7.7 Hz), 1.06 (s, 3H), 1.00 (s, 3H); ^{13}C-NMR (75 MHz, $CDCl_3$, 25 °C): δ = 164.7, 158.9, 145.3, 134.4, 134.0, 128.8, 128.4, 126.2, 122.5, 118.4, 117.0, 115.0, 114.2, 82.5, 55.4, 44.8, 43.9, 39.8, 24.1, 23.4, 20.8; IR (Neat): ν = 2963, 2932, 2836, 1670, 1639, 1613, 1586, 1511, 1440, 1396, 1325, 1289, 1245, 1175, 1143, 1112, 1035; HRMS (ESI$^+$; MeCN/CH_2Cl_2): *m/z* calculated for $C_{23}H_{27}NO_3$ 366.2069, found 366.2069.

2-(Tert-butyl)-4-cyclohexyl-2H-benzo[b][1,4]oxazin-3(4H)-one (**6o**). Compound **6o** was prepared according to the general procedure D. Purification on a column of silica gel with ethyl acetate in petroleum ether (98/2) as eluent gave the desired product (61 mg, 71%) as an orange oil. R_f = 0.6 (petroleum ether/ethyl acetate 95/5); ^{1}H-NMR (500 MHz, $CDCl_3$, 25 °C): δ = 7.13–7.07 (m, 1H), 6.98–6.92 (m, 3H), 4.24 (tt, 1H, *J* = 12.6, 3.9 Hz), 4.20 (s, 1H), 2.45 (qd, 1H, *J* = 12.4, 3.7 Hz), 2.36 (qd, 1H, *J* = 12.4, 3.7 Hz), 1.94–1.85 (m, 2H), 1.85–1.74 (m, 2H), 1.74–1.67 (m, 1H), 1.46–1.32 (m, 2H), 1.32–1.22 (m, 1H), 0.99 (s, 9H); ^{13}C-NMR (125 MHz, $CDCl_3$, 25 °C): δ = 166.0, 146.3, 129.6, 123.8, 121.9, 117.1, 115.7, 84.8, 56.8, 36.6, 29.8, 29.1, 26.8, 26.7, 26.5, 25.6; IR (Neat): ν = 2931, 2870, 1672, 1605, 1589, 1498, 1476, 1463, 1453, 1410, 1361, 1325, 1296, 1271, 1245, 1216, 1184, 1149, 1123, 1062, 1016; HRMS (ESI$^+$; MeCN/CH_2Cl_2): *m/z* calculated for $C_{18}H_{25}NO_2$ 288.1964, found 288.1959.

7-Bromo-4-(3,4-dimethoxyphenethyl)spiro[benzo[b][1,4]oxazine-2,1′-cyclobutan]-3(4H)-one (**6p**). Compound **6p** was prepared according to the general procedure D. Purification on a column of silica gel with ethyl acetate in petroleum ether (80/20) as eluent gave the desired product (111 mg, 86%) as a yellow oil. R_f = 0.5 (petroleum ether/ethyl acetate 70/30); ^{1}H-NMR (500 MHz, $CDCl_3$, 25 °C): δ = 7.18 (d, 1H, *J* = 2.0 Hz), 7.12 (dd, 1H, *J* = 8.4, 2.0 Hz), 6.80 (d, 1H, *J* = 8.1 Hz), 6.76 (d, 1H, *J* = 8.4 Hz), 6.75 (dd, 1H, *J* = 8.1, 1.9 Hz), 6.73 (d, 1H, *J* = 1.9 Hz), 4.08 (dd, 2H, *J* = 8.8, 6.8 Hz), 3.86 (s, 3H), 3.85 (s, 3H), 2.87 (dd, 2H, *J* = 8.8, 6.8 Hz), 2.60–2.51 (m, 2H), 2.32–2.22 (m, 2H), 2.04–1.85 (m, 2H); ^{13}C-NMR (125 MHz, $CDCl_3$, 25 °C): δ = 166.9, 149.1, 148.0, 144.6, 130.6, 128.4, 125.7, 121.3, 120.8, 115.6, 115.5, 112.2, 111.5, 79.9, 56.05, 55.98, 43.4, 33.1, 31.4, 13.2; IR (Neat): ν = 2953, 2834, 1677, 1590, 1515, 1491, 1453, 1418, 1386, 1332, 1275, 1259, 1235, 1179, 1140, 1121, 1075, 1027; HRMS (ESI$^+$; MeCN/CH_2Cl_2): *m/z* calculated for $C_{21}H_{22}BrNO_4$ 432.0810, found 432.0815.

4-(3,4-Dimethoxyphenethyl)-2-isobutyl-3-oxo-3,4-dihydro-2H-benzo[b][1,4]oxazine-7-carbonitrile (**6q**). Compound **6q** was prepared according to the general procedure D. Purification on a column of silica

gel with a gradient of ethyl acetate in petroleum ether (from 90/10 to 80/20) as eluent gave the desired product (98 mg, 83%) as an off-white solid. R_f = 0.4 (petroleum ether/ethyl acetate 70/30); mp 123–124 °C; ^{1}H-NMR (500 MHz, $CDCl_3$, 25 °C): δ = 7.32 (dd, 1H, *J* = 8.4, 1.7 Hz), 7.24 (d, 1H, *J* = 1.7 Hz), 6.97 (d, 1H, *J* = 8.4 Hz), 6.79 (d, 1H, *J* = 8.1 Hz), 6.73 (dd, 1H, *J* = 8.1, 1.8 Hz), 6.70 (d, 1H, *J* = 1.8 Hz), 4.63 (dd, 1H, *J* = 9.6, 4.4 Hz), 4.17 (dt, 1H, *J* = 14.1, 7.8 Hz), 4.08 (dt, 1H, *J* = 14.1, 7.8 Hz), 3.86 (s, 3H), 3.85 (s, 3H), 2.88 (t, 2H, *J* = 7.6 Hz), 1.94–1.82 (m, 1H), 1.66 (ddd, 1H, *J* = 14.6, 8.7, 4.2 Hz), 1.61 (ddd, 1H, *J* = 14.6, 8.7, 4.2 Hz), 0.97 (d, 3H, *J* = 6.4 Hz), 0.96 (d, 3H, *J* = 6.4 Hz); ^{13}C-NMR (125 MHz, $CDCl_3$, 25 °C): δ = 166.1, 149.2, 148.2, 144.1, 133.0, 130.1, 127.1, 121.0, 120.9, 118.3, 115.0, 112.2, 111.6, 107.0, 75.9, 56.1, 56.0, 43.2, 38.9, 33.0, 24.5, 23.1, 21.6; IR (Neat): ν = 2959, 2226, 1686, 1606, 1508, 1456, 1421, 1395, 1367, 1334, 1289, 1259, 1237, 1183, 1159, 1136, 1027 cm^{-1}; HRMS (ESI$^+$; MeCN/CH_2Cl_2): *m*/*z* calculated $C_{23}H_{27}N_2O_4$ 395.1971, found 395.1987.

4-Cyclohexyl-2-phenethyl-2H-benzo[b][1,4]oxazin-3(4H)-one (**6r**). Compound **6r** was prepared according to the general procedure D. Purification on a column of silica gel with ethyl acetate in petroleum ether (95/5) as eluent gave the desired product (85 mg, 84%) as a yellow oil. R_f = 0.4 (petroleum ether/ethyl acetate 95/5); ^{1}H-NMR (300 MHz, $CDCl_3$, 25 °C): δ = 7.31–7.10 (m, 6H), 7.07–6.95 (m, 3H), 4.35 (dd, 1H, *J* = 9.2, 4.3 Hz), 4.18 (tt, 1H, *J* = 12.3, 3.7 Hz), 2.97–2.73 (m, 2H), 2.44–2.26 (m, 2H), 2.26–1.99 (m, 2H), 1.94–1.82 (m, 2H), 1.82–1.63 (m, 3H), 1.46–1.21 (m, 3H); ^{13}C-NMR (75 MHz, $CDCl_3$, 25 °C): δ = 167.9, 145.4, 141.1, 130.0, 128.7, 128.5, 126.1, 123.9, 122.6, 117.8, 116.1, 76.9, 57.0, 31.6, 31.2, 29.6, 29.5, 26.5, 25.5; IR (Neat): ν = 3027, 2930, 2854, 1675, 1604, 1589, 1495, 1453, 1412, 1359, 1297, 1269, 1245, 1178, 1150, 1122, 1059; HRMS (ESI$^+$; MeCN/CH_2Cl_2): *m*/*z* calculated for $C_{22}H_{25}NO_2$ 336.1964, found 336.1961.

4-(Tert-butyl)-7-chloro-2-ethyl-2H-benzo[b][1,4]oxazin-3(4H)-one (**6s**). Compound **6s** was prepared according to the general procedure D. Purification on a column of silica gel with ethyl acetate in petroleum ether (98/2) as eluent gave the desired product (76 mg, 95%) as a yellow oil. R_f = 0.5 (petroleum ether/ethyl acetate 95/5); ^{1}H-NMR (500 MHz, $CDCl_3$, 25 °C): δ = 7.07 (d, 1H, *J* = 8.7 Hz), 7.03 (d, 1H, *J* = 2.3 Hz), 6.96 (dd, 1H, *J* = 8.7, 2.3 Hz), 4.22 (dd, 1H, *J* = 8.2, 4.0 Hz), 1.91 (dqd, 1H, *J* = 14.4, 7.5, 4.2 Hz), 1.77 (dquint, 1H, *J* = 14.4, 7.5 Hz), 1.62 (s, 9H), 1.04 (t, 3H, *J* = 7.4 Hz); ^{13}C-NMR (125 MHz, $CDCl_3$, 25 °C): δ = 172.9, 149.2, 129.4, 128.9, 121.8, 121.7, 118.3, 82.3, 59.1, 29.9, 23.7, 9.5; IR (Neat): ν = 2972, 2936, 1683, 1579, 1489, 1418, 1398, 1355, 1337, 1276, 1245, 1202, 1190, 1122, 1096, 1079, 1055, 1017; HRMS (ESI$^+$; MeCN/CH_2Cl_2): *m*/*z* calculated for $C_{14}H_{18}ClNO_2$ 268.1104, found 268.1098.

7-Chloro-2-(methoxymethyl)-2-methyl-4-phenethyl-2H-benzo[b][1,4]oxazin-3(4H)-one (**6t**). Compound **6t** was prepared according to the general procedure D. Purification on a column of silica gel with a gradient of ethyl acetate in petroleum ether (from 95/5 to 90/10) as eluent gave the desired product (77 mg, 74%) as a yellow oil. R_f = 0.62 (petroleum ether/ethyl acetate 90/10); ^{1}H-NMR (500 MHz, $CDCl_3$, 25 °C): δ = 7.33–7.28 (m, 2H), 7.25–7.21 (m, 3H), 7.00 (d, 1H, *J* = 2.3 Hz), 6.95 (dd, 1H, *J* = 8.6, 2.3 Hz), 6.79 (d, 1H, *J* = 8.6 Hz), 4.15 (dt, 1H, *J* = 13.9, 7.8 Hz), 4.06 (dt, 1H, *J* = 13.9, 7.8 Hz), 3.82 (d, 1H, *J* = 10.4 Hz), 3.58 (d, 1H, *J* = 10.4 Hz), 3.38 (s, 3H), 2.94 (t, 2H, *J* = 7.9 Hz), 1.40 (s, 3H); ^{13}C-NMR (125 MHz, $CDCl_3$, 25 °C): δ = 166.3, 144.2, 138.0, 129.0, 128.7, 128.6, 127.1, 126.8, 122.2, 118.1, 114.8, 80.9, 76.0, 60.0, 43.3, 33.3, 19.5; IR (Neat): ν = 2932, 1674, 1603, 1587, 1496, 1454, 1425, 1393, 1368, 1326, 1286, 1189, 1154, 1109, 1087, 1030; HRMS (ESI$^+$; MeCN/CH_2Cl_2): *m*/*z* calculated for $C_{19}H_{20}ClNO_3$ 346.1210, found 346.1206.

2-(Tert-butyl)-7-chloro-4-(4-chlorobenzyl)-2H-benzo[b][1,4]oxazin-3(4H)-one (**6u**). Compound **6u** was prepared according to the general procedure D. Purification on a column of silica gel with ethyl acetate in petroleum ether (95/5) as eluent gave the desired product (104 mg, 95%) as an off-white solid. R_f = 0.3 (petroleum ether/ethyl acetate 95/5); mp 142–143 °C; ^{1}H-NMR (500 MHz, $CDCl_3$, 25 °C): δ = 7.30 (d, 2H, *J* = 8.5 Hz), 7.21 (d, 2H, *J* = 8.5 Hz), 6.99 (d, 1H, *J* = 2.2 Hz), 6.83 (dd, 1H, *J* = 8.6, 2.2 Hz), 6.70 (d, 1H, *J* = 8.6 Hz), 5.18 (d, 1H, *J* = 16.1 Hz), 5.00 (d, 1H, *J* = 16.1 Hz), 4.43 (s, 1H), 1.07 (s, 9H); ^{13}C-NMR (125 MHz, $CDCl_3$, 25 °C): δ = 164.3, 146.1, 134.8, 133.6, 129.2, 129.1, 128.4, 127.3, 122.0, 117.0, 115.7, 84.6, 44.9, 37.2, 26.7; IR (Neat): ν = 2966, 1671, 1582, 1494, 1463, 1421, 1391, 1369, 1329, 1277, 1186,

1142, 1085, 1054, 1015; HRMS (ESI+; MeCN/CH_2Cl_2): *m*/*z* calculated for $C_{19}H_{19}Cl_2NO_2$ 364.0871, found 364.0879.

4-Cyclohexyl-2-(methoxymethyl)-2-methyl-2H-benzo[b][1,4]oxazin-3(4H)-one (**6v**). Compound **6v** was prepared according to the general procedure D. Purification on a column of silica gel with ethyl acetate in petroleum ether (98/2) as eluent gave the desired product (73 mg, 83%) as a yellow oil. R_f = 0.4 (petroleum ether/ethyl acetate 96/4); ^{1}H-NMR (500 MHz, $CDCl_3$, 25 °C): δ = 7.17–7.10 (m, 1H), 7.05–6.95 (m, 3H), 4.18 (tt, 1H, *J* = 12.4, 3.8 Hz), 3.83 (d, 1H, *J* = 10.4 Hz), 3.60 (d, 1H, *J* = 10.4 Hz), 3.43 (s, 3H), 2.50–2.27 (m, 2H), 1.96–1.66 (m, 5H), 1.49–1.18 (m, 3H), 1,34 (s, 3H); ^{13}C-NMR (75 MHz, $CDCl_3$, 25 °C): δ = 168.6, 144.3, 129.6, 123.9, 122.3, 118.5, 115.6, 80.9, 75.7, 60.2, 57.2, 29.7, 29.2, 26.7, 26.6, 25.7, 18.9. IR (Neat): (cm^{-1}) 2930, 2854, 1674, 1607, 1497, 1452, 1411, 1357, 1327, 1299, 1277, 1248, 1204, 1109, 1045; HRMS (ESI+; MeCN/CH_2Cl_2): *m*/*z* calculated for $C_{17}H_{23}NO_3$ 290.1756, found 290.1754.

4-Cyclohexylspiro[benzo[b][1,4]oxazine-2,1′-cyclobutan]-3(4H)-one (**6w**). Compound **6w** was prepared according to the general procedure D. Purification on a column of silica gel with ethyl acetate in petroleum ether (98/2) as eluent gave the desired product (39 mg, 89%) as a yellow solid. R_f = 0.7 (petroleum ether/ethyl acetate 95/5); mp 87–88 °C; ^{1}H-NMR (300 MHz, $CDCl_3$, 25 °C): δ = 7.15–7.08 (m, 1H), 7.05–6.93 (m, 3H), 4.18 (tt, 1H, *J* = 12.3, 3.7 Hz), 2.59–2.47 (m, 2H), 2.44–2.28 (m, 2H), 2.28–2.15 (m, 2H), 2.02–1.65 (m, 7H), 1.46–1.21 (m, 3H); ^{13}C-NMR (75 MHz, $CDCl_3$, 25 °C): δ = 169.2, 144.7, 130.2, 123.7, 122.7, 118.5, 116.0, 80.2, 56.9, 31.0, 29.6, 26.6, 25.7, 13.4; IR (Neat): ν = 2943, 2857, 1672, 1607, 1491, 1448, 1406, 1358, 1325, 1297, 1256, 1244, 1147, 1036 cm^{-1}; HRMS (ESI+; MeCN/CH_2Cl_2): *m*/*z* calculated for $C_{17}H_{22}NO_2$ 272.1651, found 272.1643.

4-Cyclohexyl-2-ethyl-2H-pyrido[3,2-b][1,4]oxazin-3(4H)-one (**6x**). Compound **6x** was prepared according to the general procedure D. Purification on a column of silica gel with a gradient of ethyl acetate in petroleum ether (from 98/2 to 95/5) as eluent gave the desired product (44 mg, 56%) as a yellow solid. R_f = 0.6 (petroleum ether/ethyl acetate 90/10); mp 64–65 °C; ^{1}H-NMR (300 MHz, $CDCl_3$, 25 °C): δ = 7.99 (dd, 1H, *J* = 4.8, 1.4 Hz), 7.21 (dd, 1H, *J* = 7.9, 1.4 Hz), 6.90 (dd, 1H, *J* = 7.9, 4.8 Hz), 4.88 (tt, 1H, *J* = 12.3, 3.6 Hz), 4.42 (dd, 1H, *J* = 8.2, 4.6 Hz), 2.62–2.41 (m, 2H), 1.98–1.75 (m, 4H), 1.75–1.62 (m, 3H), 1.48–1.20 (m, 3H), 1.04 (t, 3H, *J* = 7.4 Hz); ^{13}C-NMR (75 MHz, $CDCl_3$, 25 °C): δ = 167.5, 142.8, 140.6, 140.3, 123.9, 119.1, 78.8, 53.9, 29.2, 29.1, 26.6, 25.5, 24.3, 9.5; IR (Neat): ν = 2967, 2921, 2849, 1673, 1595, 1456, 1407, 1365, 1333, 1269, 1240, 1193, 1109, 1064 cm^{-1}; HRMS (ESI+; MeCN/CH_2Cl_2): *m*/*z* calculated for $C_{15}H_{21}N_2O_2$ 261.1603, found 261.1604.

1-Cyclohexyl-3-isopropyl-1H-pyrido[2,3-b][1,4]oxazin-2(3H)-one (**6y**). Compound **6y** was prepared according to the general procedure D. Purification on a column of silica gel with a gradient of ethyl acetate in petroleum ether (from 80/20 to 70/30) as eluent gave the desired product (43 mg, 71%) as a yellow oil. R_f = 0.4 (petroleum ether/ethyl acetate 70/30); ^{1}H-NMR (300 MHz, $CDCl_3$, 25 °C): δ = 7.90 (dd, 1H, *J* = 4.8, 1.5 Hz), 7.42 (dd, 1H, *J* = 7.9, 1.5 Hz), 6.97 (dd, 1H, *J* = 7.9, 4.8 Hz), 4.43 (d, 1H, *J* = 5.7 Hz), 4.20 (tt, 1H, *J* = 12.3, 3.7 Hz), 2.39–2.16 (m, 3H), 1.95–1.84 (m, 2H), 1.84–1.65 (m, 3H), 1.48–1.18 (m, 3H), 1.09 (d, 3H, *J* = 6.9 Hz), 1.00 (d, 3H, *J* = 6.9 Hz); ^{13}C-NMR (75 MHz, $CDCl_3$, 25 °C): δ = 166.2, 152.6, 141.5, 124.2, 123.3, 118.3, 82.7, 56.4, 30.3, 29.5, 29.1, 26.43, 26.40, 25.4, 18.8, 17.4; IR (Neat): ν = 2966, 2934, 2857, 1678, 1579, 1454, 1413, 1364, 1320, 1240, 1214, 1133, 1074, 1056, 1019 cm^{-1}; HRMS (ESI+; MeCN/CH_2Cl_2): *m*/*z* calculated for $C_{16}H_{23}N_2O_2$ 275.1760, found 275.1762.

2-(4-Chlorophenyl)-N-cyclohexyl-2-oxoacetamide (**7**). The general procedure D applied to the compound 2f didn't give the desired product. Instead, the compound 7 was formed and was isolated as a yellow solid (29 mg, 46%) after purification on a column of silica gel with a gradient of ethyl acetate in petroleum ether (from 99/1 to 98/2) as eluent. R_f = 0.5 (petroleum ether/ethyl acetate 95/5); mp 101–102 °C; ^{1}H-NMR (300 MHz, $CDCl_3$, 25 °C): δ = 8.32 (d, 2H, *J* = 8.8 Hz), 7.44 (d, 2H, *J* = 8.8 Hz), 7.00 (br d, 1H, *J* = 5.7 Hz), 3.91–3.75 (m, 1H), 2.03–1.91 (m, 2H), 1.83–1.70 (m, 2H), 1.70–1.59 (m, 1H), 1.50–1.12 (m, 5H); ^{13}C-NMR (75 MHz, $CDCl_3$, 25 °C): δ = 186.8, 160.5, 141.3, 132.8, 131.9, 128.9, 48.6, 32.8, 25.5,

24.8; IR (Neat): ν = 3381, 2936, 2856, 1655, 1583, 1527, 1448, 1397, 1283, 1263, 1246, 1205, 1169, 1092, 1005 cm^{-1}; HRMS (ESI$^+$; MeCN/CH_2Cl_2): *m*/*z* calculated for $C_{14}H_{17}NO_2Cl$ 266.0948, found 266.0945.

4-(4-Chlorobenzyl)-2-((S)-2,6-dimethylhept-5-en-1-yl)-7-nitro-2H-benzo[b][1,4]oxazin-3(4H)-one (**8a**). Compound **8a** was prepared according to the general procedure D. Purification on a column of silica gel with a gradient of ethyl acetate in petroleum ether (from 95/5 to 80/20) as eluent gave the desired product (75 mg, 56%) as a 6:4 mixture of unseparable diastereomers (orange oil). R_f = 0.4 (petroleum ether/ethyl acetate 90/10); ^{1}H-NMR (500 MHz, $CDCl_3$, 25 °C): δ = 7.86 (d, 1H, *J* = 2.5 Hz), 7.85 (d, 1H, *J* = 2.5 Hz), 7.83 (dd, 1H, *J* = 8.8, 2.5 Hz), 7.32 (d, 2H, *J* = 8.2 Hz), 7.15 (dd, 2H, *J* = 8.2 Hz), 6.90 (dd, 1H, *J* = 8.8, 1.0 Hz), 5.24 (d, 0.4H, *J* = 9.0 Hz), 5.21 (d, 0.6H, *J* = 9.0 Hz), 5.13–5.02 (m, 2H), 4.87–4.82 (m, 1H), 2.12–1.94 (m, 2H), 1.94–1.79 (m, 2H), 1.78–1.67 (m, 0.6H), 1.70 (s, 1.8H), 1.66 (s, 1.2H), 1.61 (s, 1.2H), 1.55 (s, 1.8H), 1.53–1.45 (m, 0.6H), 1.40–1.19 (m, 1.8H), 1.03 (d, 1.2H, *J* = 6.1 Hz), 1.02 (d, 1.8H, *J* = 6.1 Hz); ^{13}C-NMR (75 MHz, $CDCl_3$, 25 °C): δ = 166.7, 166.5, 143.93, 143.87, 143.8, 134.4, 133.9, 133.7, 131.85, 131.78, 129.4, 128.0, 124.4, 124.2, 118.7, 114.9, 113.3, 76.1, 75.6, 44.9, 37.6, 37.4, 37.2, 36.1, 29.3, 28.4, 25.83, 25.76, 25.4, 25.3, 20.0, 18.8, 17.8, 17.7; IR (Neat): ν = 2962, 2919, 2853, 1694, 1600, 1522, 1500, 1439, 1384, 1337, 1301, 1246, 1147, 1092, 1059, 1014 cm^{-1}; HRMS (ESI$^+$; MeCN/CH_2Cl_2): *m*/*z* calculated for $C_{24}H_{28}ClN_2O_4$ 443.1738, found 443.1736.

4-Cyclohexyl-2-ethyl-7-nitro-2H-benzo[b][1,4]oxazin-3(4H)-one (**8b**). Compound **8b** was prepared according to the general procedure D. Purification on a column of silica gel with ethyl acetate in petroleum ether (90/10) as eluent gave the desired product (58 mg, 64%) as a yellow solid. R_f = 0.4 (petroleum ether/ethyl acetate 90/10); mp 92–93 °C; ^{1}H-NMR (300 MHz, $CDCl_3$, 25 °C): δ = 7.92 (dd, 1H, *J* = 9.0, 2.4 Hz), 7.86 (d, 1H, *J* = 2.4 Hz), 7.22 (d, 1H, *J* = 9.0 Hz), 4.41 (dd, 1H, *J* = 8.5, 4.5 Hz), 4.23 (tt, 1H, *J* = 12.4, 3.4 Hz), 2.42–2.24 (m, 2H), 1.98–1.85 (m, 3H), 1.85–1.70 (m, 4H), 1.49–1.24 (m, 3H), 1.06 (t, 3H, *J* = 7.4 Hz); ^{13}C-NMR (75 MHz, $CDCl_3$, 25 °C): δ = 167.4, 145.3, 143.3, 135.7, 118.4, 115.5, 113.4, 79.2, 57.5, 29.5, 29.4, 26.4, 25.4, 23.7, 9.5; IR (Neat): ν = 3094, 2919, 2853, 1692, 1598, 1510, 1495, 1456, 1411, 1336, 1307, 1276, 1258, 1247, 1207, 1152, 1135, 1114, 1079, 1052 cm^{-1}; HRMS (ESI$^+$; MeCN/CH_2Cl_2): *m*/*z* calculated for $C_{18}H_{24}N_3O_4$ 346.1767, found 346.1774.

2-Isopropyl-4-(4-methoxybenzyl)-7-nitro-2H-benzo[b][1,4]oxazin-3(4H)-one (**8c**). Compound **8c** was prepared according to the general procedure D. Purification on a column of silica gel with a gradient of ethyl acetate in petroleum ether (from 98/2 to 90/10) as eluent gave the desired product (73 mg, 68%) as a yellow solid. R_f = 0.4 (petroleum ether/ethyl acetate 90/10); mp 107–108 °C; ^{1}H-NMR (500 MHz, $CDCl_3$, 25 °C): δ = 7.85 (d, 1H, *J* = 2.5 Hz), 7.80 (dd, 1H, *J* = 8.9, 2.5 Hz), 7.16 (d, 2H, *J* = 8.7 Hz), 6.97 (d, 1H, *J* = 8.9 Hz), 6.87 (d, 2H, *J* = 8.7 Hz), 5.25 (d, 1H, *J* = 15.8 Hz), 5.02 (d, 1H, *J* = 15.8 Hz), 4.50 (d, 1H, *J* = 6.5 Hz), 3.78 (s, 3H), 2.32 (oct, 1H, *J* = 6.8 Hz), 1.13 (d, 3H, *J* = 6.8 Hz), 1.08 (d, 3H, *J* = 6.8 Hz); ^{13}C-NMR (125 MHz, $CDCl_3$, 25 °C): δ = 165.4, 159.3, 144.4, 143.5, 134.5, 128.0, 127.2, 118.4, 115.0, 114.6, 112.8, 82.0, 55.4, 45.0, 30.0, 18.7, 17.6; IR (Neat): ν = 3064, 2964, 2923, 1684, 1599, 1511, 1462, 1447, 1384, 1368, 1334, 1306, 1293, 1253, 1236, 1189, 1178, 1153, 1111, 1086, 1032 cm^{-1}; HRMS (ESI$^+$; MeCN/CH_2Cl_2): *m*/*z* calculated for $C_{19}H_{21}N_2O_5$ 357.1450, found 357.1455.

2-(Tert-butyl)-7-nitro-4-phenethyl-2H-benzo[b][1,4]oxazin-3(4H)-one (**8d**). Compound **8d** was prepared according to the general procedure D. Purification on a column of silica gel with petroleum ether/ethyl acetate 95/5 as eluent gave the desired product (87 mg, 82%) as a yellow solid. R_f = 0.5 (petroleum ether/ethyl acetate 90/10); mp 123–124 °C; ^{1}H-NMR (500 MHz, $CDCl_3$, 25 °C): δ = 7.89 (dd, 1H, *J* = 8.9, 2.5 Hz), 7.84 (d, 1H, *J* = 2.5 Hz), 7.35–7.29 (m, 2H), 7.28–7.23 (m, 3H), 6.96 (d, 1H, *J* = 8.9 Hz), 4.38 (s, 1H), 4.29 (ddd, 1H, *J* = 14.1, 9.6, 6.3 Hz), 4.11 (ddd, 1H, *J* = 14.1, 9.6, 6.3 Hz), 3.04–2.91 (m, 2H), 1.04 (s, 9H); ^{13}C-NMR (75 MHz, $CDCl_3$, 25 °C): δ = 163.7, 145.2, 143.5, 137.5, 134.1, 128.89, 128.86, 127.1, 118.2, 113.8, 112.2, 84.3, 43.4, 37.2, 33.5, 26.5; IR (Neat): ν = 3063, 3028, 2971, 2872, 1684, 1597, 1516, 1498, 1478, 1457, 1446, 1387, 1371, 1334, 1312, 1298, 1280, 1254, 1229, 1191, 1145, 1085, 1052, 1026 cm^{-1}; HRMS (ESI$^+$; MeCN/CH_2Cl_2): *m*/*z* calculated for $C_{20}H_{23}N_2O_4$ 355.1658, found 355.1643.

4-(4-Methoxybenzyl)-2-(2-methylpent-4-en-2-yl)-7-nitro-2H-benzo[b][1,4]oxazin-3(4H)-one (**8e**). Compound **8e** was prepared according to the general procedure D. Purification on a column of silica gel with ethyl acetate in petroleum ether (90/10) as eluent gave the desired product (70 mg, 59%) as a yellow oil. R_f = 0.5 (petroleum ether/ethyl acetate 90/10); ^{1}H-NMR (300 MHz, $CDCl_3$, 25 °C): δ = 7.82 (d, 1H, J = 2.5 Hz), 7.78 (dd, 1H, J = 8.8, 2.5 Hz), 7.20 (d, 2H, J = 8.6 Hz), 6.99 (d, 1H, J = 8.8 Hz), 6.86 (d, 2H, J = 8.6 Hz), 5.94–5.76 (m, 1H), 5.19 (d, 1H, J = 15.7 Hz), 5.16–5.10 (m, 2H), 5.07 (d, 1H, J = 15.7 Hz), 4.57 (s, 1H), 3.78 (s, 3H), 2.32 (dd, 1H, J = 13.5, 7.6 Hz), 2.21 (dd, 1H, J = 13.5, 7.6 Hz), 1.08 (s, 3H), 1.02 (s, 3H); ^{13}C-NMR (75 MHz, $CDCl_3$, 25 °C): δ = 164.4, 159.3, 145.3, 143.5, 134.3, 133.6, 128.3, 127.3, 119.0, 118.1, 114.9, 114.5, 111.9, 82.6, 55.4, 45.1, 43.7, 40.1, 24.0, 23.4; IR (Neat): ν = 2964, 2933, 1686, 1639, 1599, 1513, 1464, 1384, 1337, 1295, 1243, 1176, 1176, 1113, 1085, 1035 cm^{-1}; HRMS (ESI$^+$; MeCN/CH_2Cl_2): m/z calculated for $C_{22}H_{25}N_2O_5$ 397.1763, found 397.1756.

2-(Tert-butyl)-4-cyclohexyl-7-nitro-2H-benzo[b][1,4]oxazin-3(4H)-one (**8f**). Compound **8f** was prepared according to the general procedure D. Purification on a column of silica gel with ethyl acetate in petroleum ether (90/10) as eluent gave the desired product (70 mg, 70%) as a yellow solid. R_f = 0.5 (petroleum ether/ethyl acetate 90/10); mp 107–108 °C; ^{1}H-NMR (300 MHz, $CDCl_3$, 25 °C): δ = 7.88 (dd, 1H, J = 9.0, 2.5 Hz), 7.82 (d, 1H, J = 2.5 Hz), 7.18 (d, 1H, J = 9.0 Hz), 4.38–4.22 (m, 1H), 4.29 (s, 1H), 2.50–2.23 (m, 2H), 1.97–1.86 (m, 2H), 1.86–1.68 (m, 3H), 1.50–1.17 (m, 3H), 1.00 (s, 9H); ^{13}C-NMR (75 MHz, $CDCl_3$, 25 °C): δ = 165.4, 146.1, 143.3, 135.2, 118.0, 115.1, 112.4, 84.8, 57.3, 37.0, 29.8, 29.0, 26.65, 26.58, 26.4, 25.4; IR (Neat): ν = 3124, 3093, 2988, 2968, 2926, 2855, 1686, 1596, 1509, 1495, 1477, 1466, 1451, 1410, 1360, 1339, 1309, 1291, 1262, 1243, 1190, 1148, 1080, 1056, 1021 cm^{-1}; HRMS (ESI$^+$; MeCN/CH_2Cl_2): m/z calculated for $C_{18}H_{25}N_2O_4$ 333.1814, found 333.1814.

7-Nitro-2,4-diphenethyl-2H-benzo[b][1,4]oxazin-3(4H)-one (**8g**). Compound **8g** was prepared according to the general procedure D. Purification on a column of silica gel with a gradient of ethyl acetate in petroleum ether (from 95/5 to 90/10) as eluent gave the desired product (79 mg, 65%) as a yellow solid. R_f = 0.3 (petroleum ether/ethyl acetate 90/10); mp 79–80 °C; ^{1}H-NMR (500 MHz, $CDCl_3$, 25 °C): δ = 7.92 (dd, 1H, J = 8.9, 2.6 Hz), 7.86 (d, 1H, J = 2.6 Hz), 7.33–7.27 (m, 4H), 7.25–7.18 (m, 6H), 6.97 (d, 1H, J = 8.9 Hz), 4.55 (dd, 1H, J = 9.1, 4.2 Hz), 4.23 (ddd, 1H, J = 14.2, 8.6, 7.1 Hz), 4.13 (ddd, 1H, J = 14.2, 8.6, 6.6 Hz), 3.00–2.90 (m, 2H), 2.88–2.77 (m, 2H), 2.27–2.18 (m, 1H), 2.17–2.08 (m, 1H); ^{13}C-NMR (75 MHz, $CDCl_3$, 25 °C): δ = 165.6, 144.0, 143.4, 140.3, 137.5, 134.5, 128.9, 128.7, 127.1, 126.4, 118.7, 114.2, 113.3, 76.1, 43.4, 33.4, 32.0, 30.9; IR (Neat): ν = 3086, 3027, 2932, 1695, 1598, 1514, 1495, 1454, 1387, 1336, 1296, 1276, 1246, 1182, 1137, 1109, 1073, 1053, 1029 cm^{-1}; HRMS (ESI$^+$; MeCN/CH_2Cl_2): m/z calculated for $C_{24}H_{23}N_2O_4$ 403.1658, found 403.1664.

4-(3,4-Dimethoxyphenethyl)-2-((S)-2,6-dimethylhept-5-en-1-yl)-7-nitro-2H-benzo[b][1,4]oxazin-3(4H)-one (**8h**). Compound **8h** was prepared according to the general procedure D. Purification on a column of silica gel with a gradient of ethyl acetate in petroleum ether (from 90/10 to 80/20) as eluent gave the desired product (107 mg, 74%) as a 1:1 mixture of unseparable diastereomers (yellow solid). R_f = 0.3 (petroleum ether/ethyl acetate 80/20); ^{1}H-NMR (500 MHz, $CDCl_3$, 25 °C): δ = 7.94 (dd, 1H, J = 8.9, 2.6 Hz), 7.85 (d, 1H, J = 2.6 Hz), 7.84 (d, 1H, J = 2.6 Hz), 7.00 (d, 1H, J = 8.9 Hz), 6.99 (d, 1H, J = 8.9 Hz), 6.79 (d, 1H, J = 8.0 Hz), 6.74 (br dd, 1H, J = 8.0, 1.9 Hz), 6.72 (br t, 1H, J = 1.9 Hz), 5.09 (t, 0.5H, J = 7.1 Hz), 5.04 (t, 0.5H, J = 7.1 Hz), 4.71–4.66 (m, 1H), 4.24–4.08 (m, 2H), 3.87 (s, 3H), 3.85 (s, 3H), 2.90 (m, 2H), 2.09–1.90 (m, 2H), 1.83–1.74 (m, 2H), 1.69 (s, 1.5H), 1.66 (s, 1.5H), 1.63–1.56 (m, 0.5H), 1.60 (s, 1.5H), 1.55 (s, 1.5H), 1.50–1.41 (m, 0.5H), 1.36–1.23 (m, 1.5H), 1.23–1.16 (m, 0.5H), 0.99 (d, 1.5H, J = 6.1 Hz), 0.98 (d, 1.5H, J = 6.1 Hz); ^{13}C-NMR (75 MHz, $CDCl_3$, 25 °C): δ = 166.2, 166.0, 149.2, 148.2, 143.93, 143.86, 143.5, 134.58, 134.55, 131.8, 131.7, 130.0, 124.4, 124.2, 120.9, 118.7, 114.2, 113.4, 112.1, 111.5, 76.0, 75.5, 56.0, 43.45, 43.43, 37.4, 37.0, 36.1, 33.1, 29.2, 28.4, 25.82, 25.76, 25.4, 25.3, 20.0, 18.8, 17.8, 17.7; IR (Neat): ν = 2961, 2916, 2851, 1693, 1597, 1502, 1466, 1454, 1444, 1419, 1395, 1328, 1305, 1290, 1277, 1257, 1236, 1192, 1184, 1157, 1135, 1079, 1059, 1025 cm^{-1}; HRMS (ESI$^+$; MeCN/CH_2Cl_2): m/z calculated for $C_{27}H_{35}N_2O_6$ 483.2495, found 483.2516.

4-(4-Chlorobenzyl)-2-ethyl-7-nitro-2H-benzo[b][1,4]oxazin-3(4H)-one (**8i**). Compound **8i** was prepared according to the general procedure D. Purification on a column of silica gel with a gradient of ethyl acetate in petroleum ether (from 98/2 to 90/10) as eluent gave the desired product (71 mg, 68%) as an off-white solid. R_f = 0.2 (petroleum ether/ethyl acetate 90/10); mp 148–149 °C; ^{1}H-NMR (500 MHz, $CDCl_3$, 25 °C): δ = 7.88 (d, 1H, *J* = 2.5 Hz), 7.82 (dd, 1H, *J* = 8.9, 2.5 Hz), 7.32 (d, 2H, *J* = 8.4 Hz), 7.15 (d, 2H, *J* = 8.4 Hz), 6.89 (d, 1H, *J* = 8.9 Hz), 5.22 (d, 1H, *J* = 16.2 Hz), 5.10 (d, 1H, *J* = 16.2 Hz), 4.69 (dd, 1H, *J* = 8.5, 4.5 Hz), 2.04 (dqd, 1H, *J* = 14.4, 7.4, 4.5 Hz), 1.95 (dquint, 1H, *J* = 14.4, 7.4 Hz), 1.14 (t, 3H, *J* = 7.4 Hz); ^{13}C-NMR (125 MHz, $CDCl_3$, 25 °C): δ = 166.1, 144.0, 143.6, 134.2, 133.8, 133.6, 129.3, 127.9, 118.5, 114.8, 113.2, 78.4, 44.8, 24.1, 9.4; IR (Neat): ν = 3090, 3069, 2979, 2931, 1688, 1597, 1515, 1492, 1441, 1389, 1326, 1310, 1299, 1283, 1246, 1198, 1154, 1132, 1109, 1092, 1078, 1061, 1039, 1015 cm^{-1}; HRMS (ESI$^+$; MeCN/CH_2Cl_2): *m*/*z* calculated for $C_{17}H_{15}ClN_2O_4$ 346.0720, found 346.0718.

4-Cyclohexyl-2-isopropyl-7-nitro-2H-benzo[b][1,4]oxazin-3(4H)-one (**8j**). Compound **8j** was prepared according to the general procedure D. Purification on a column of silica gel with a gradient of ethyl acetate in petroleum ether (from 95/5 to 80/20) as eluent gave the desired product (79 mg, 83%) as a white solid. R_f = 0.6 (petroleum ether/ethyl acetate 90/130); mp 83–84 °C; ^{1}H-NMR (500 MHz, $CDCl_3$, 25 °C): δ = 7.91 (dd, 1H, *J* = 9.0, 2.6 Hz), 7.85 (d, 1H, *J* = 2.6 Hz), 7.21 (d, 1H, *J* = 9.0 Hz), 4.26 (d, 1H, *J* = 6.3 Hz), 4.24 (tt, 1H, *J* = 12.1, 3.6 Hz), 2.42–2.29 (m, 2H), 2.19 (oct, 1H, *J* = 6.8 Hz), 1.95–1.88 (m, 2H), 1.84–1.70 (m, 3H), 1.45–1.34 (m, 2H), 1.33–1.24 (m, 1H), 1.06 (d, 3H, *J* = 6.8 Hz), 1.00 (d, 3H, *J* = 6.8 Hz); ^{13}C-NMR (125 MHz, $CDCl_3$, 25 °C): δ = 166.6, 145.5, 143.3, 135.5, 118.3, 115.3, 113.2, 82.7, 57.5, 29.6, 29.5, 29.2, 26.5, 26.4, 25.4, 18.6, 17.5; IR (Neat): ν = 2974, 2932, 2857, 1702, 1594, 1513, 1497, 1464, 1453, 1408, 1369, 1357, 1337, 1310, 1286, 1261, 1244, 1205, 1192, 1146, 1118, 1084, 1059, 1026 cm^{-1}; HRMS (ESI$^+$; MeCN/CH_2Cl_2): *m*/*z* calculated for $C_{17}H_{23}N_2O_4$ 319.1658, found 319.1642.

3. ^{1}H and ^{13}C-NMR Spectra of Compounds

1-(4-Chloro-2-nitrophenoxy)-N-cyclohexylcyclobutanecarboxamide (**1b**)

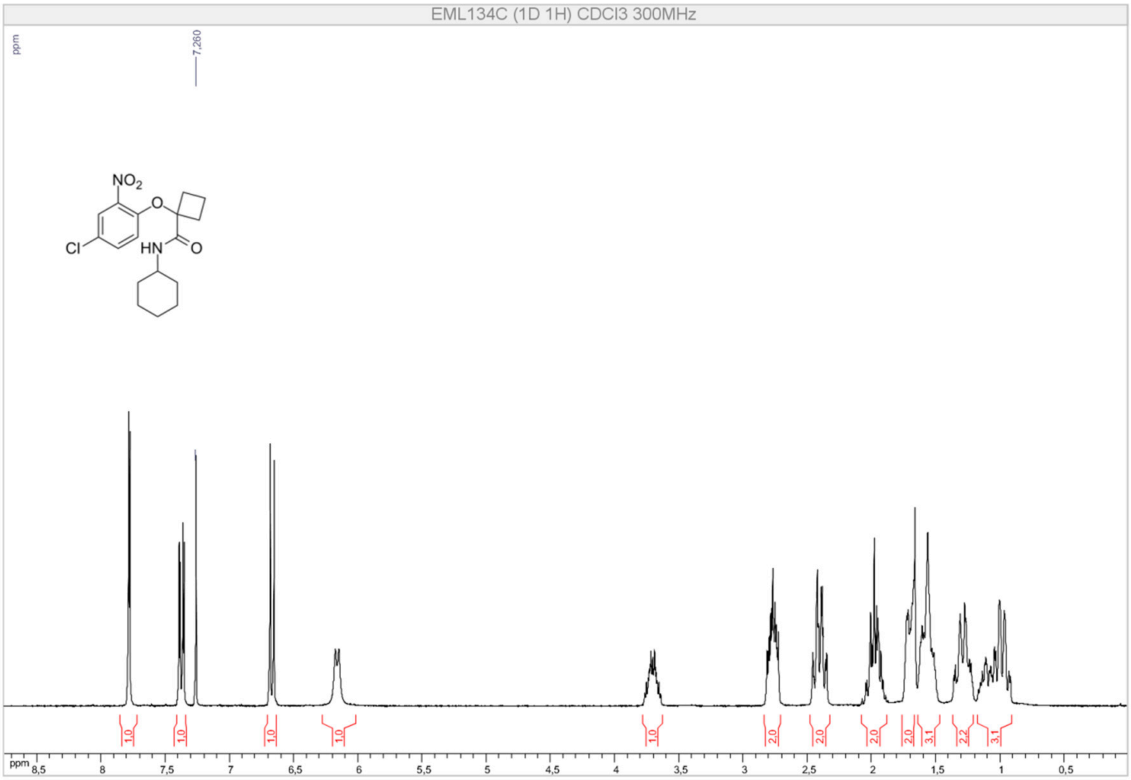

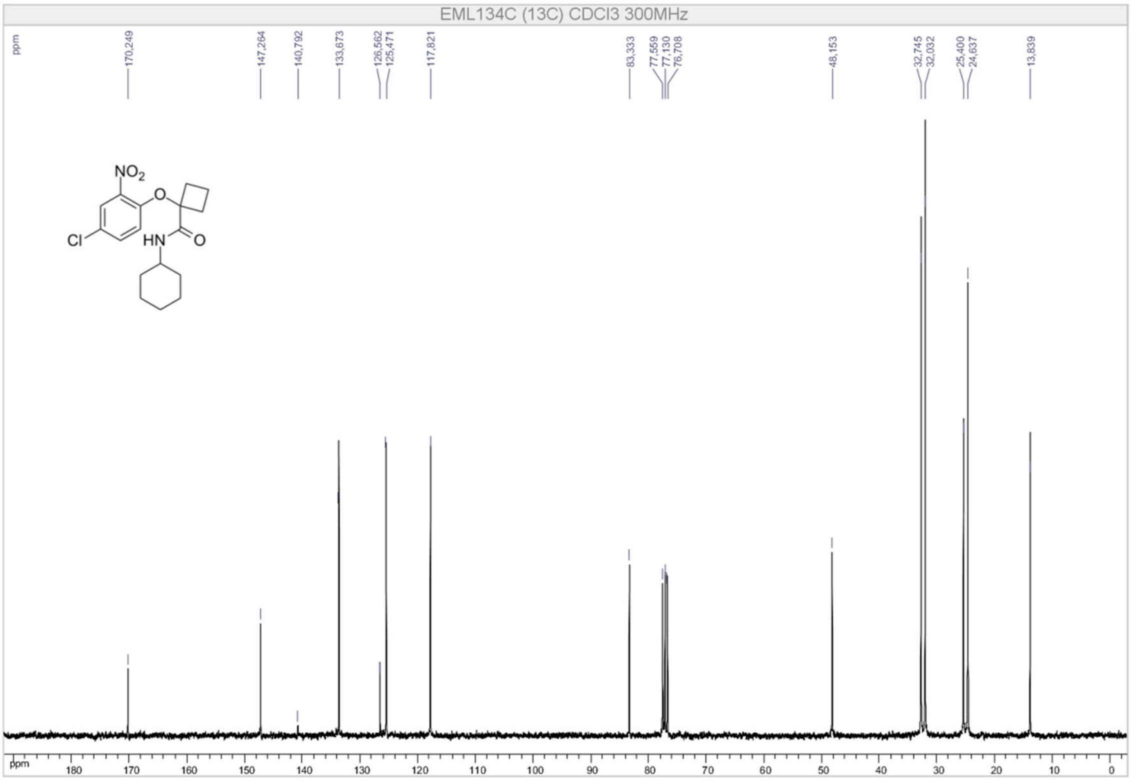

N-(4-Methoxybenzyl)-1-(2-nitrophenoxy)cyclobutanecarboxamide (**1c**)

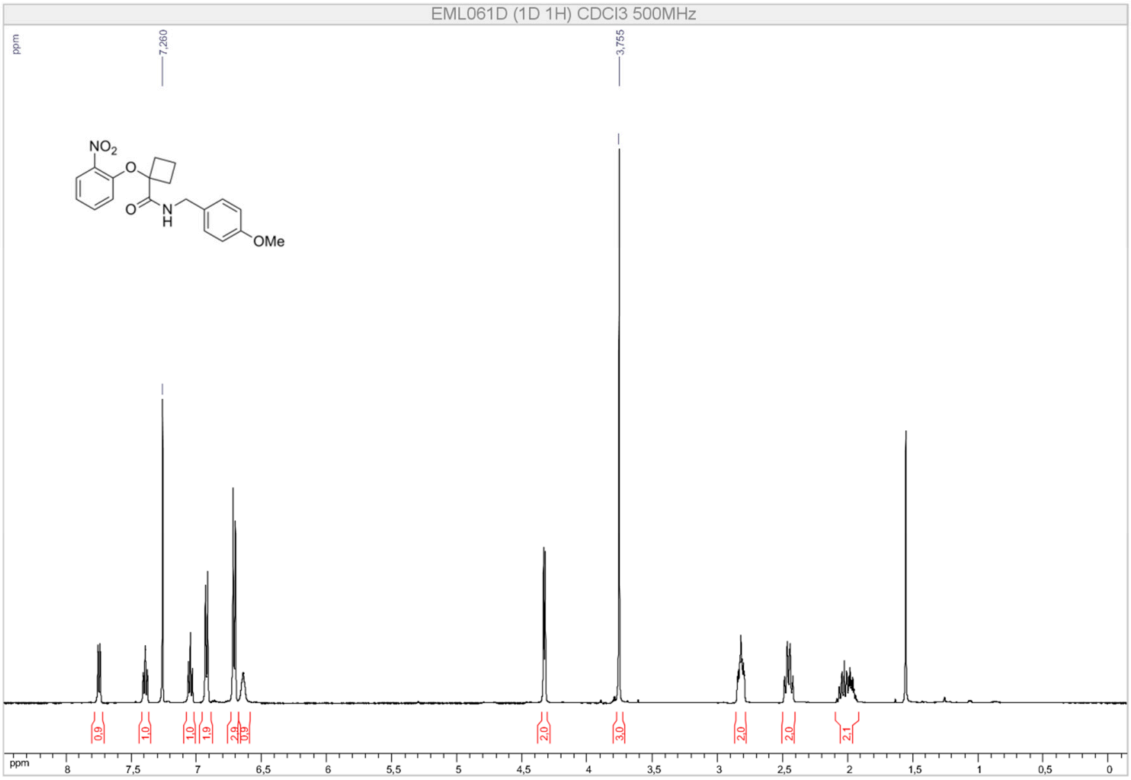

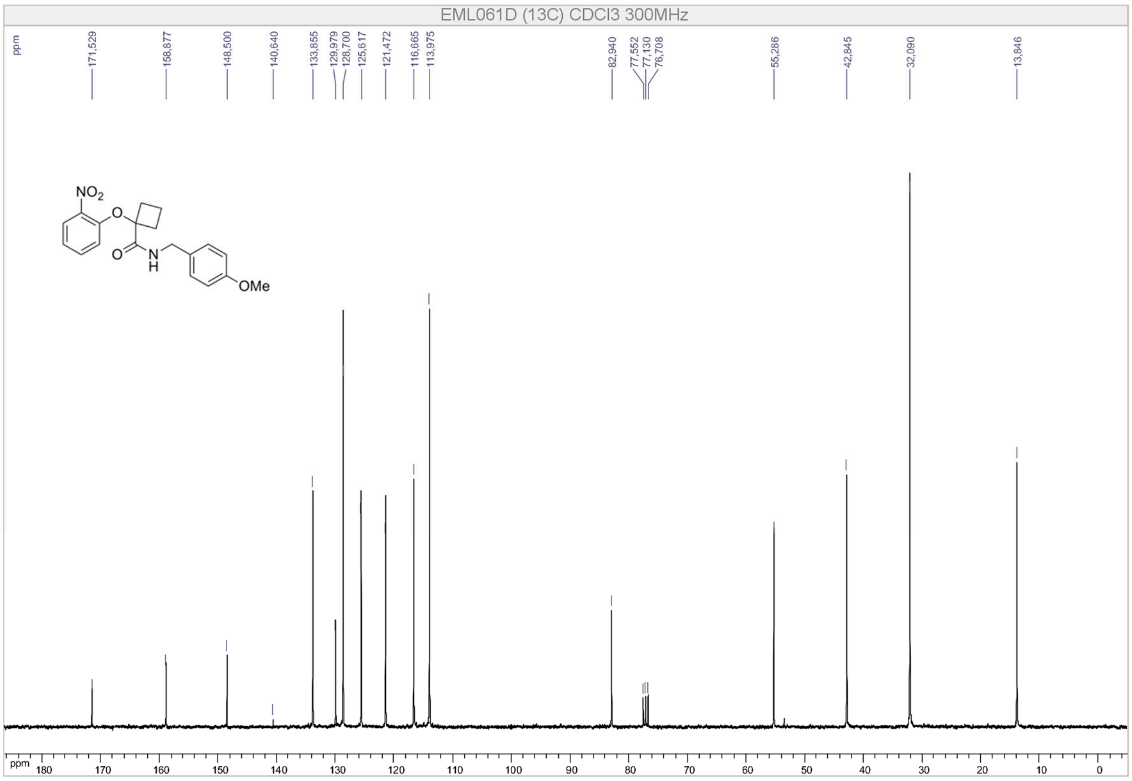

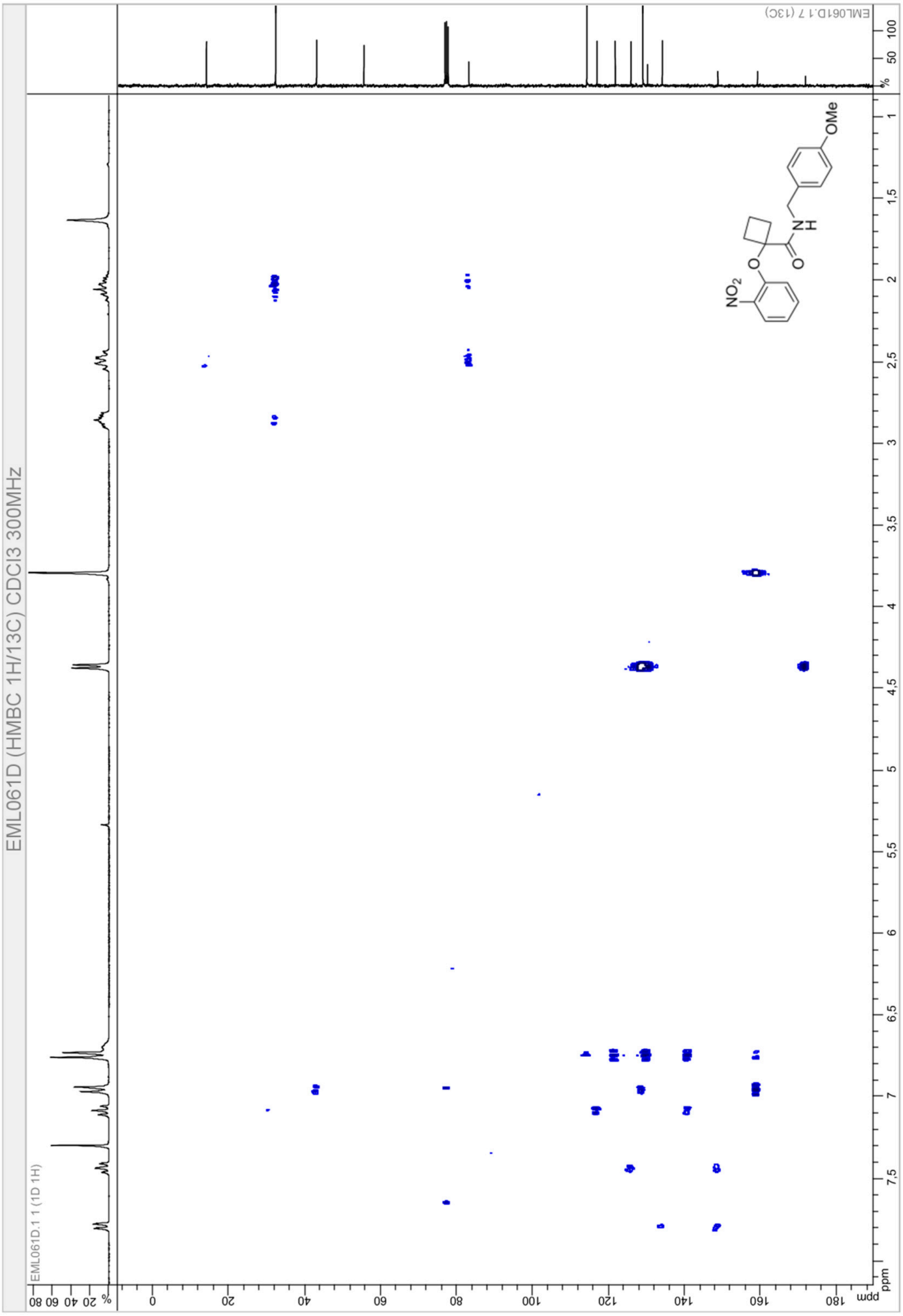
EML061D (HMBC 1H/13C) CDCl3 300MHz
EML061D.1 7 (13C)
EML061D.1 1 (1D 1H)
NO2
O
NH
OMe

1-(4-Bromo-2-nitrophenoxy)-N-(3,4-dimethoxyphenethyl)cyclobutanecarboxamide (**1d**)

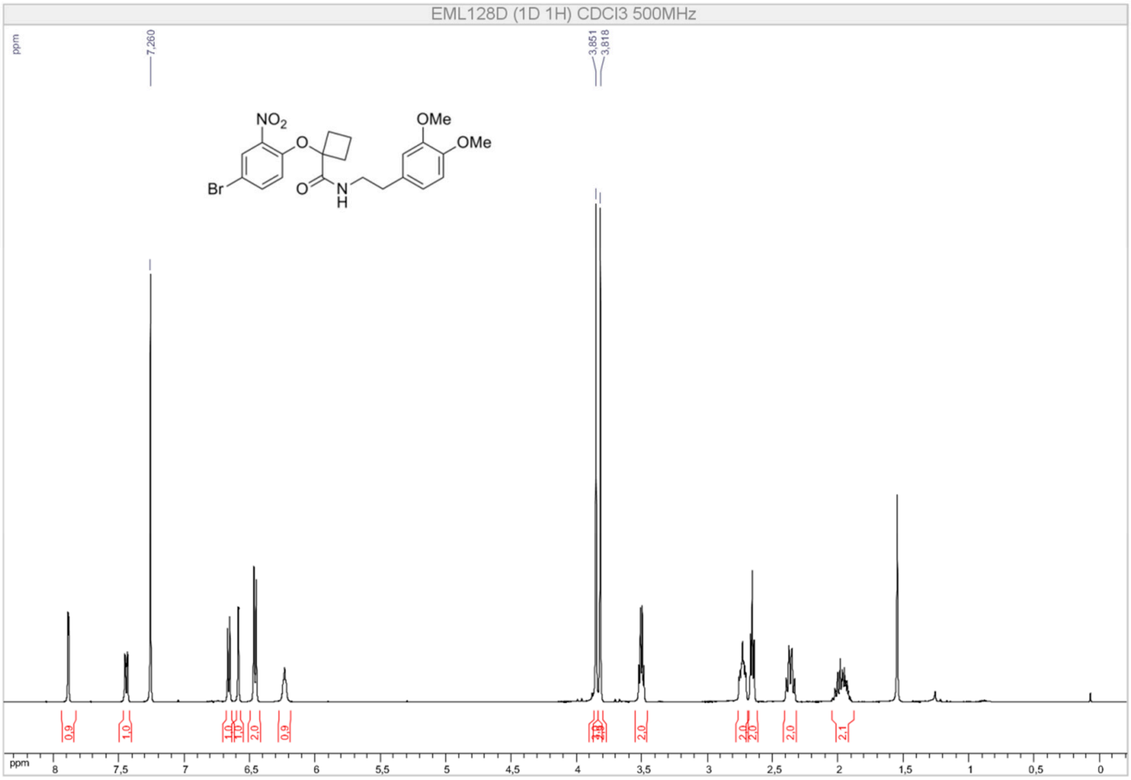

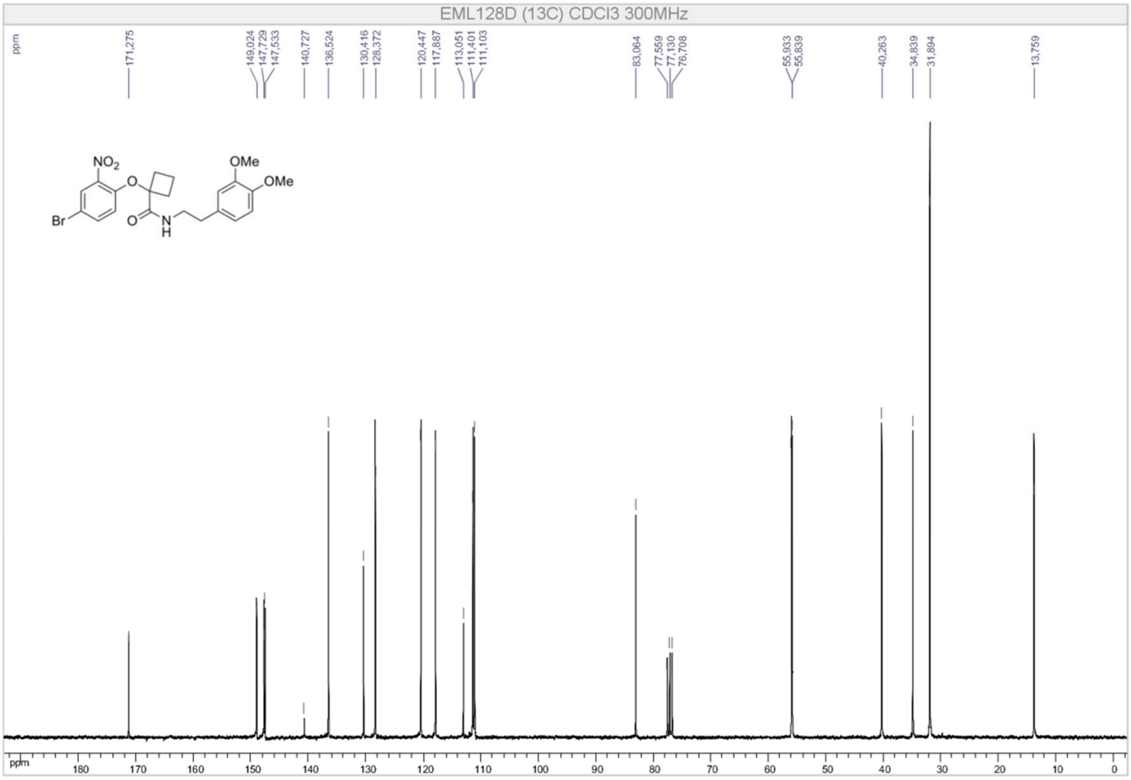

N-Cyclohexyl-3,3,3-trifluoro-2-methyl-2-(2-nitrophenoxy)propanamide (**1e**)

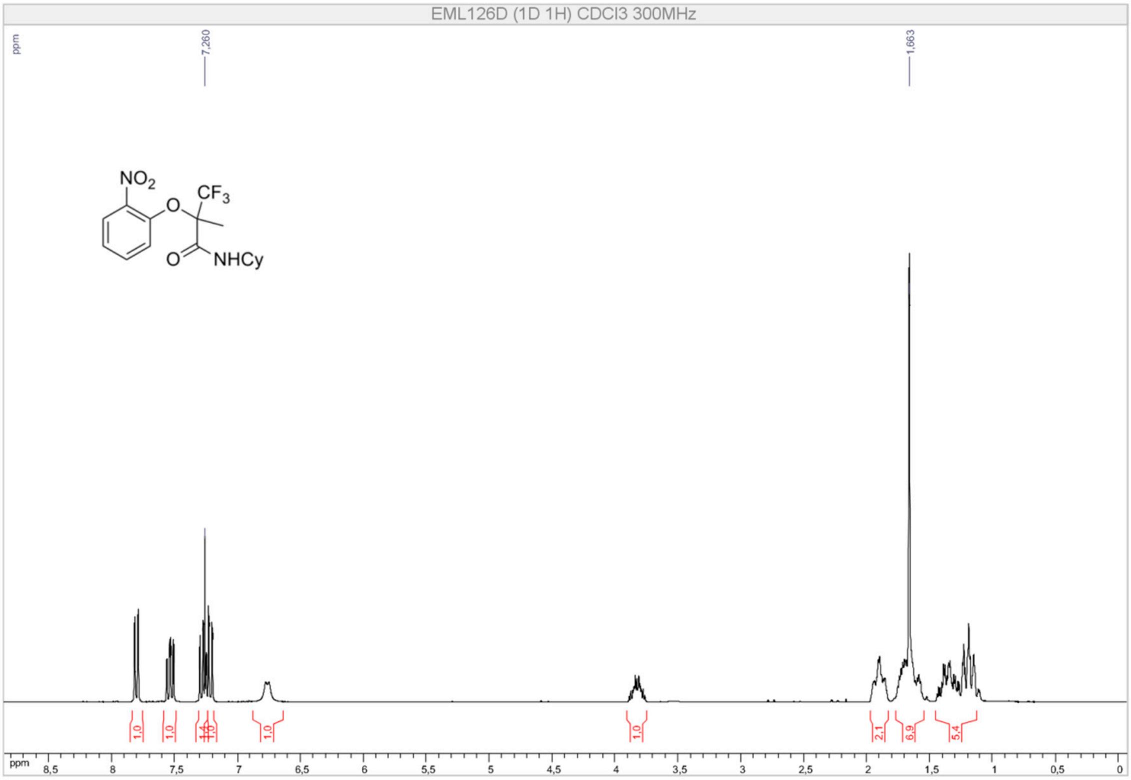

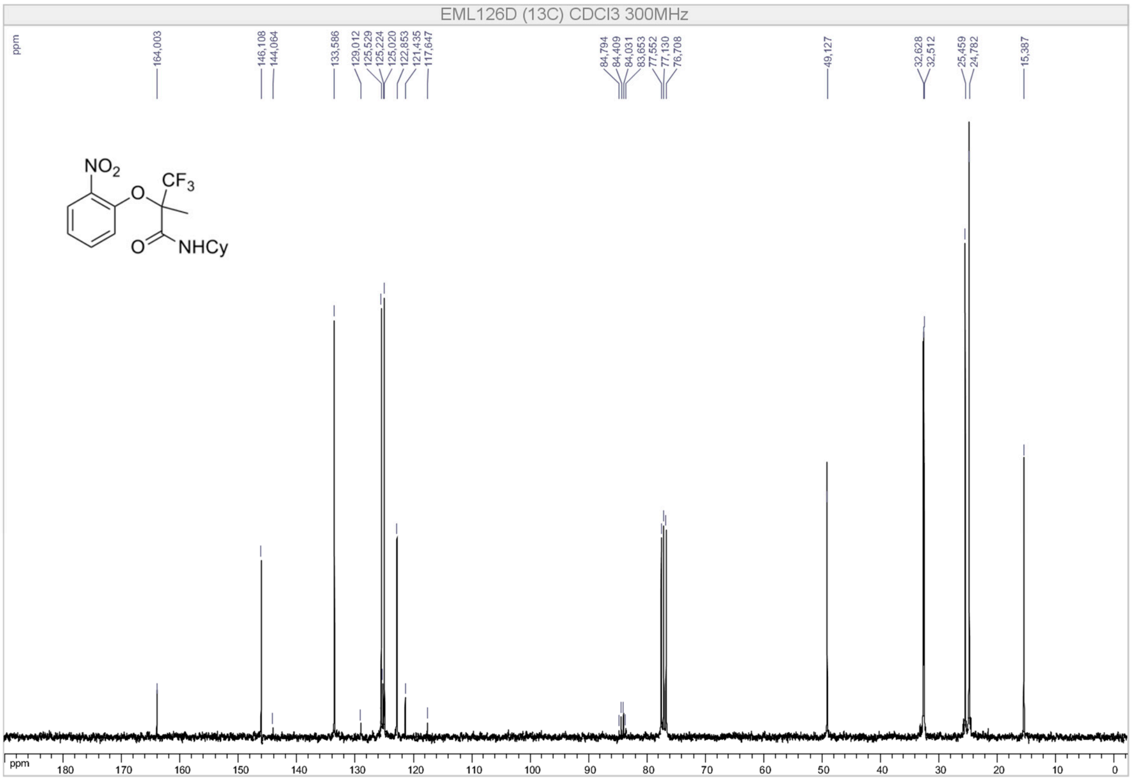

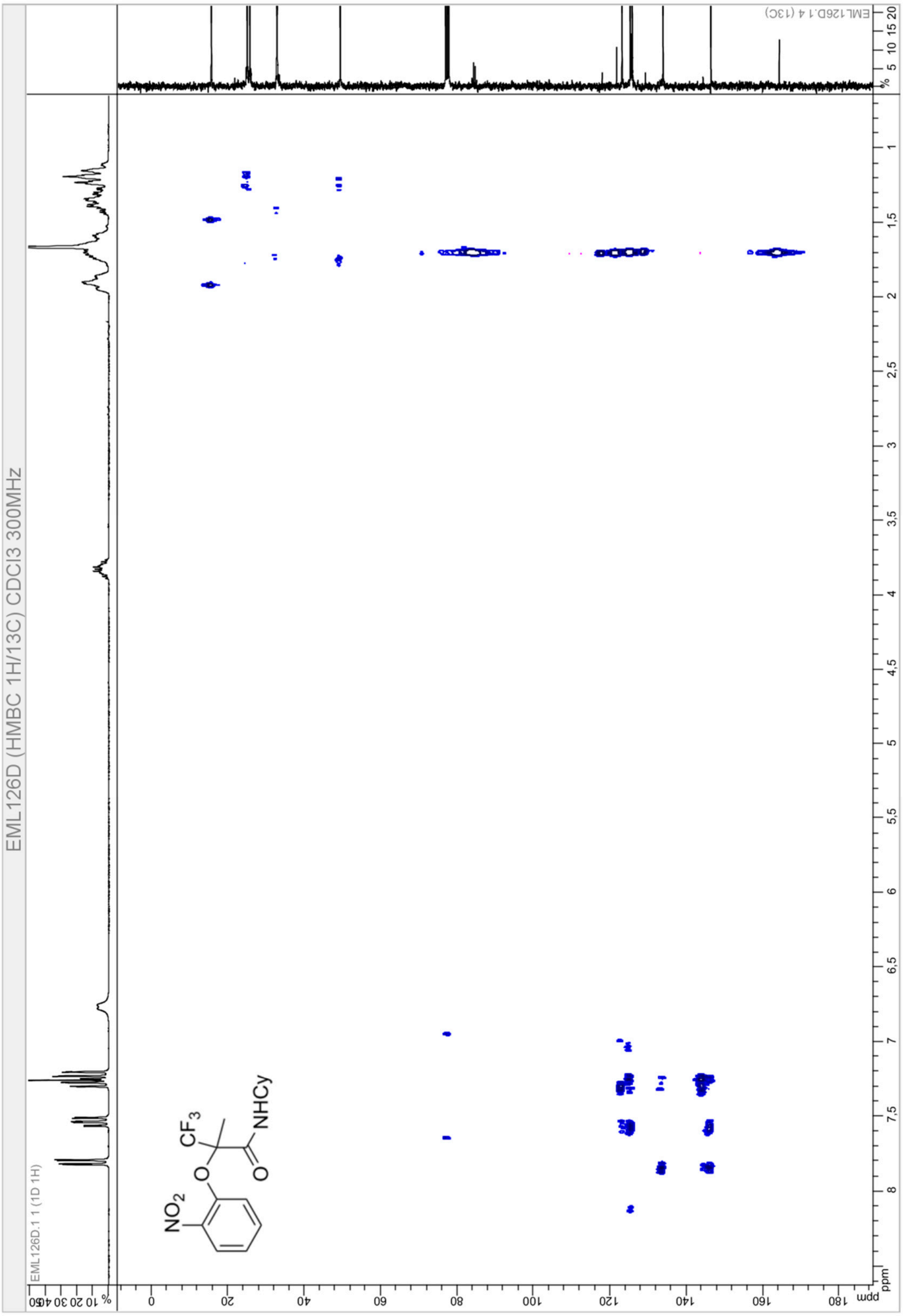
EML126D (HMBC 1H/13C) CDCl3 300MHz
EML126D.1 1 (1D 1H)
EML126D.1 4 (13C)
ppm

N-Cyclohexyl-3-methoxy-2-methyl-2-(2-nitrophenoxy)propanamide (**1f**)

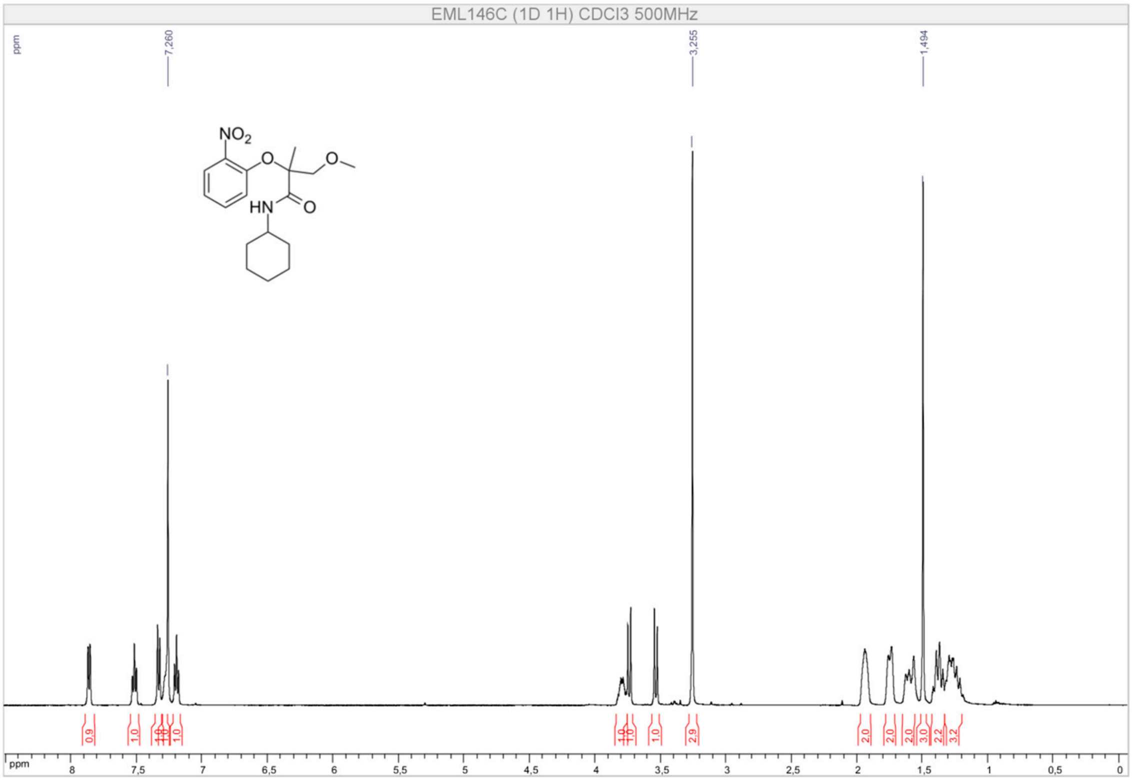

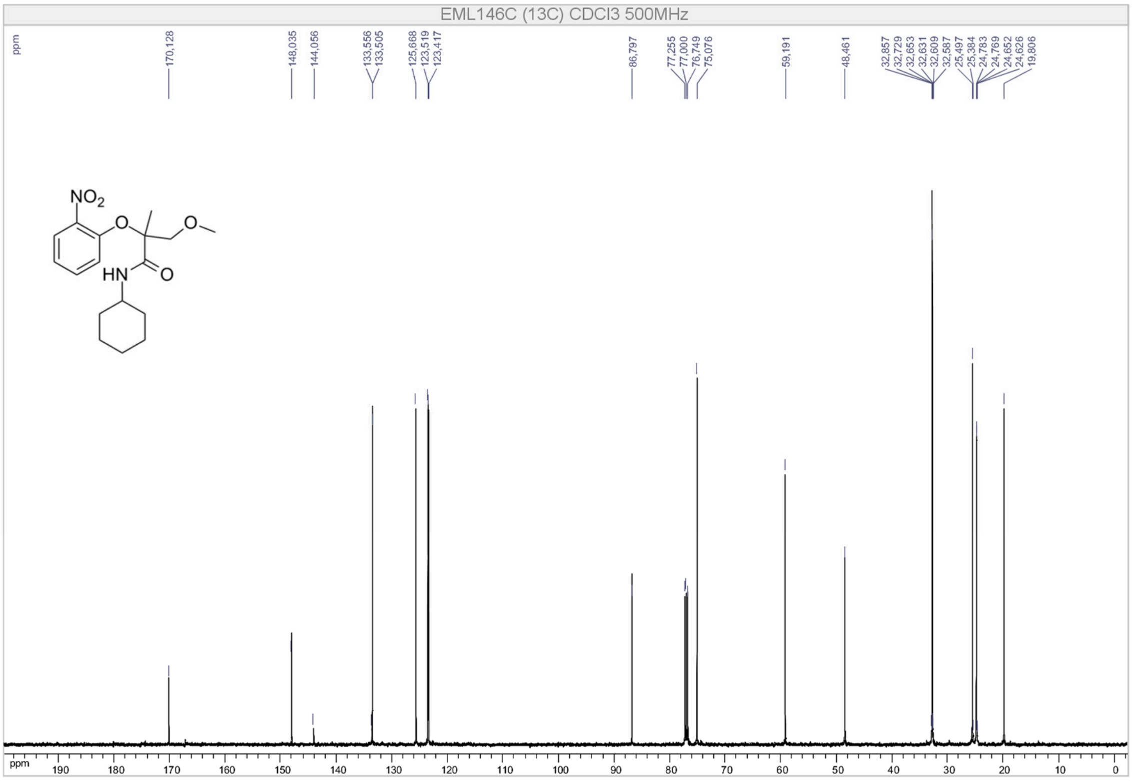

2-(4-Chloro-2-nitrophenoxy)-3-methoxy-2-methyl-N-phenethylpropanamide (**1g**)

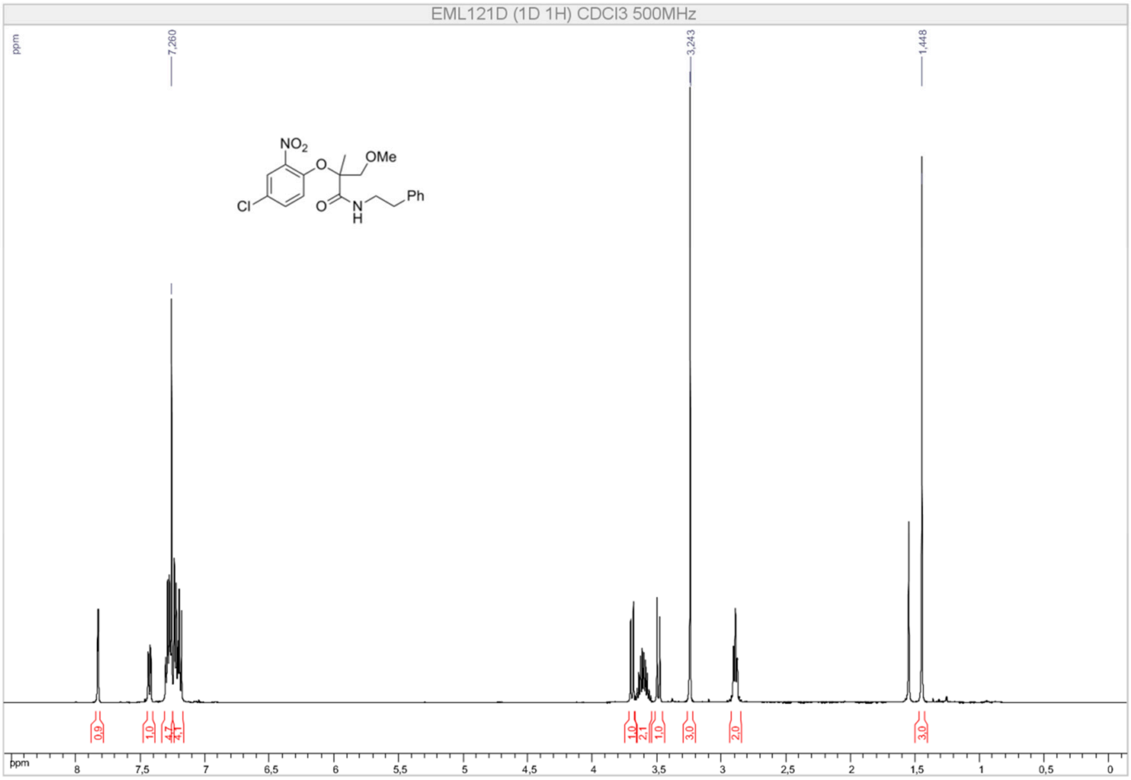

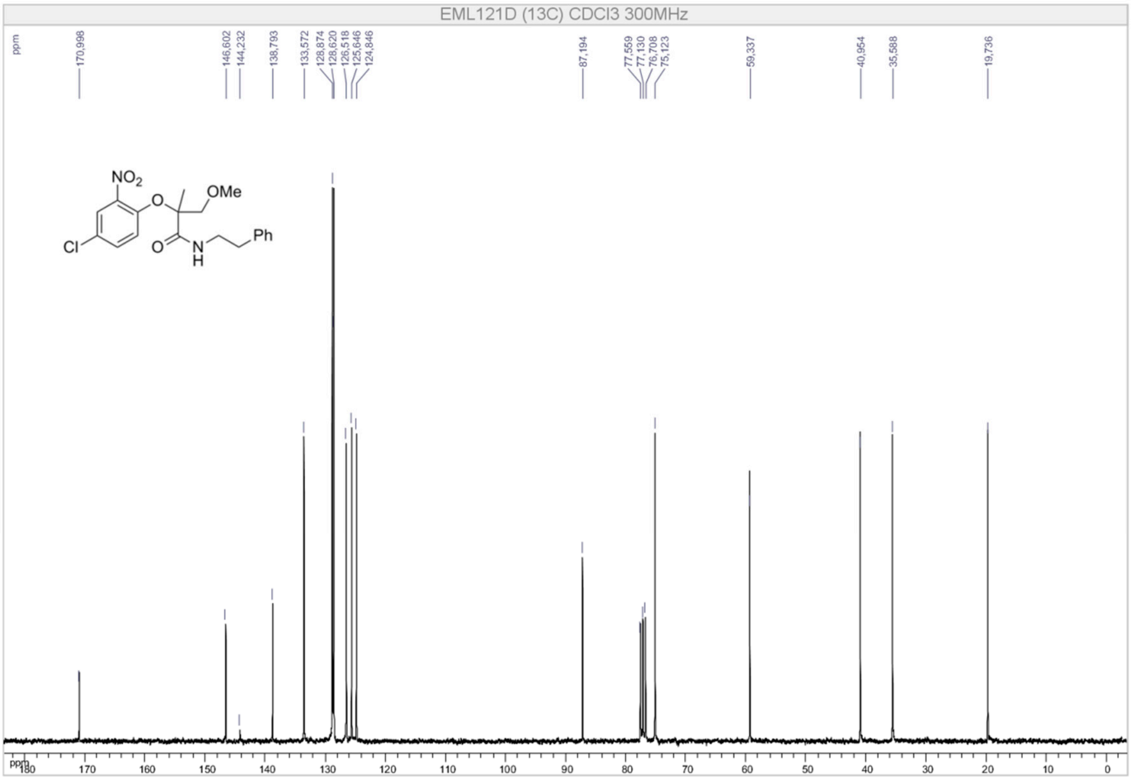

N-Cyclohexyl-2-(2-nitrophenoxy)butanamide (**2a**)

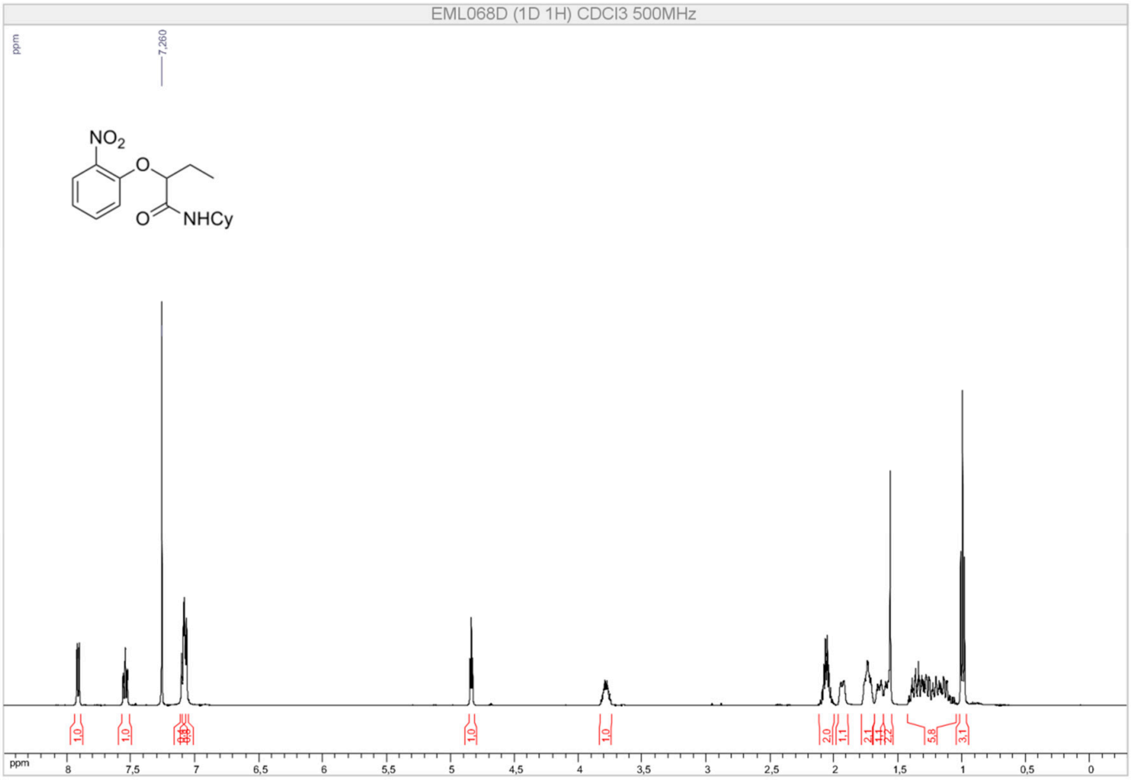

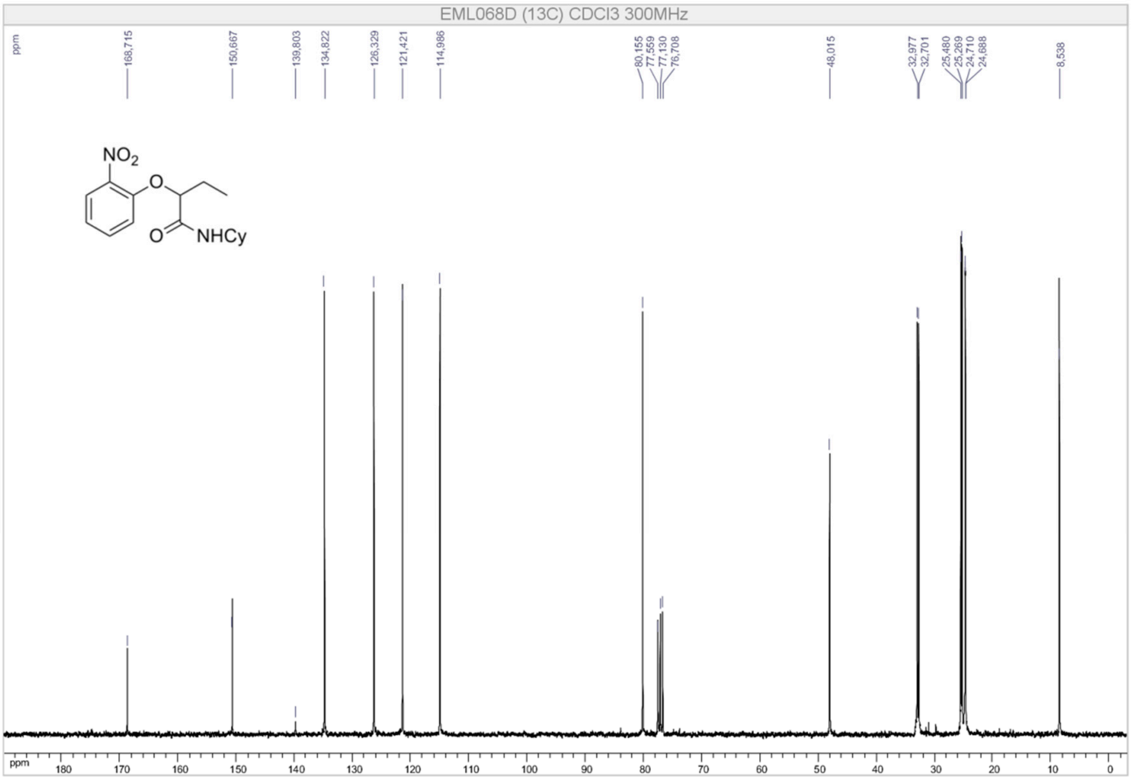

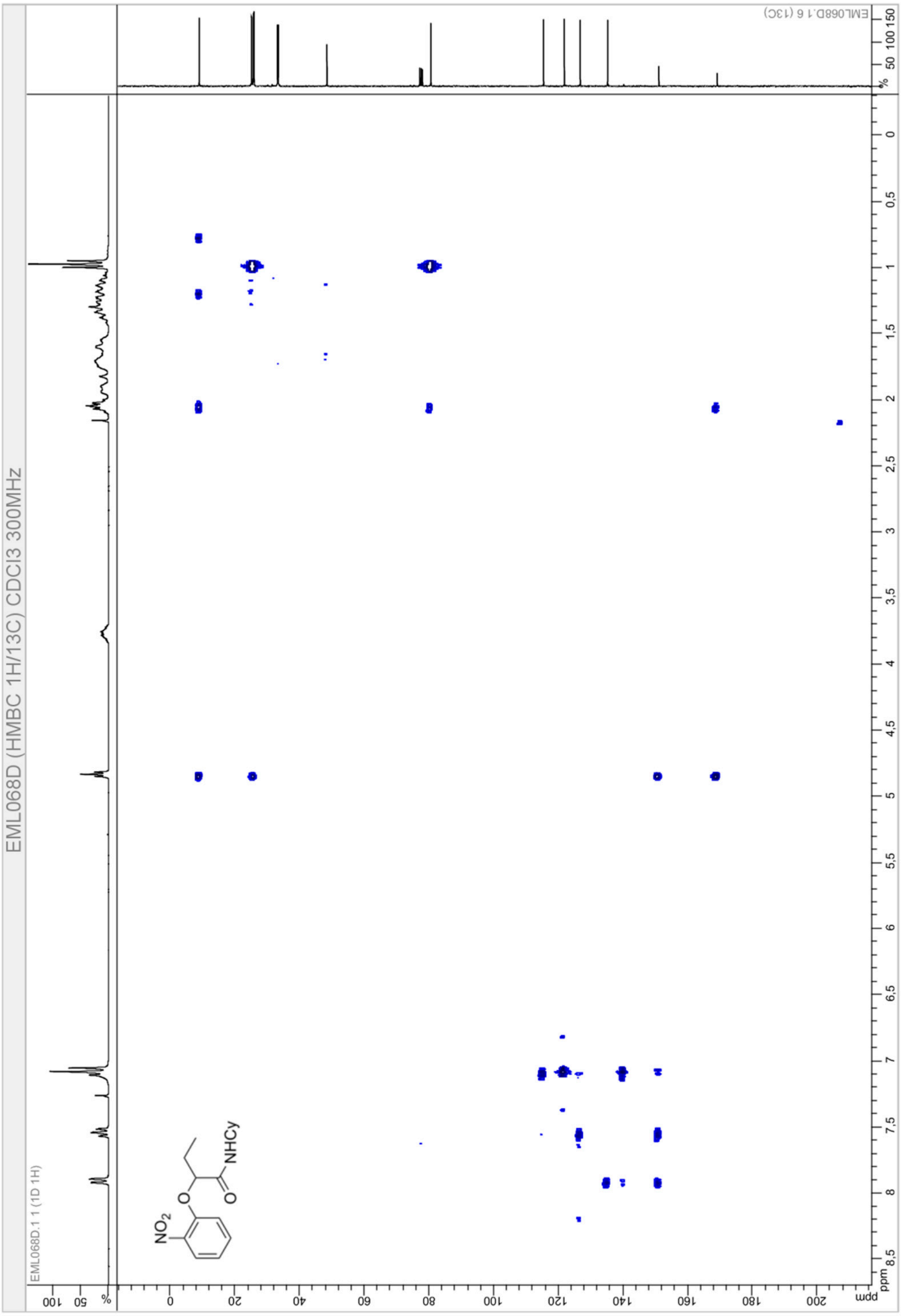
EML068D (HMBC 1H/13C) CDCl3 300MHz
EML068D.1 6 (13C)
EML068D.1 1 (1D 1H)
NO2
O
NHCy
ppm

N-Cyclohexyl-3-methyl-2-(2-nitrophenoxy)butanamide (**2b**)

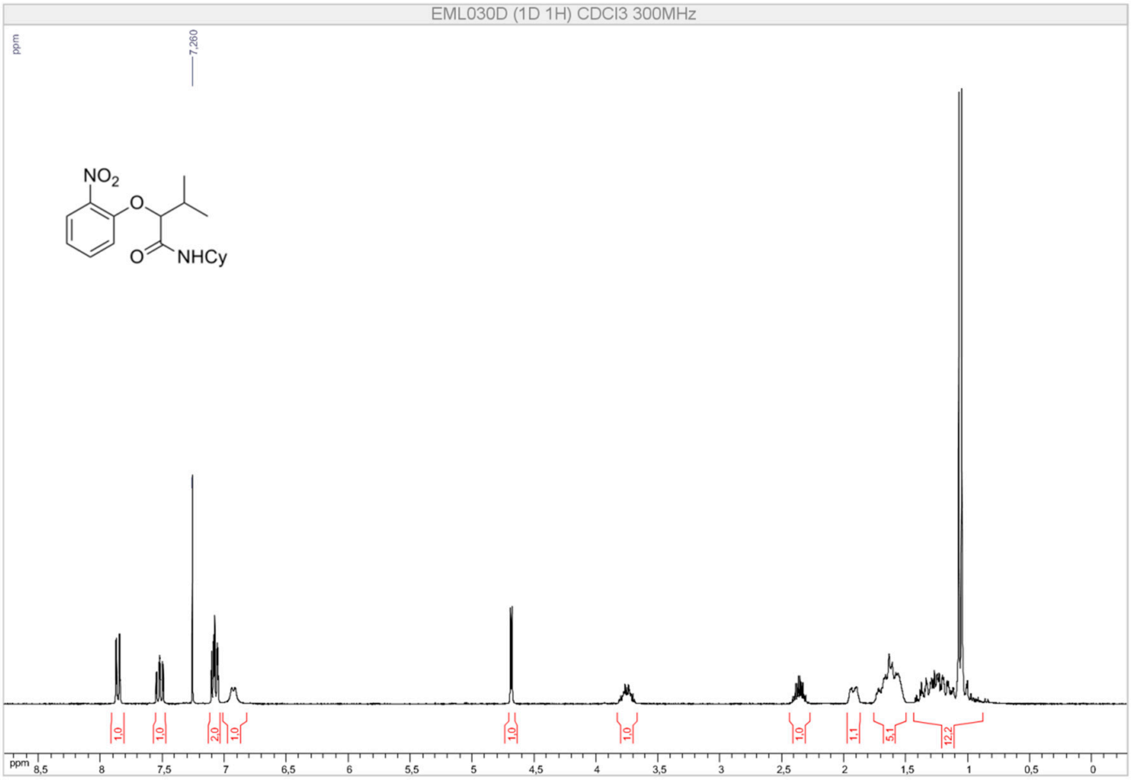

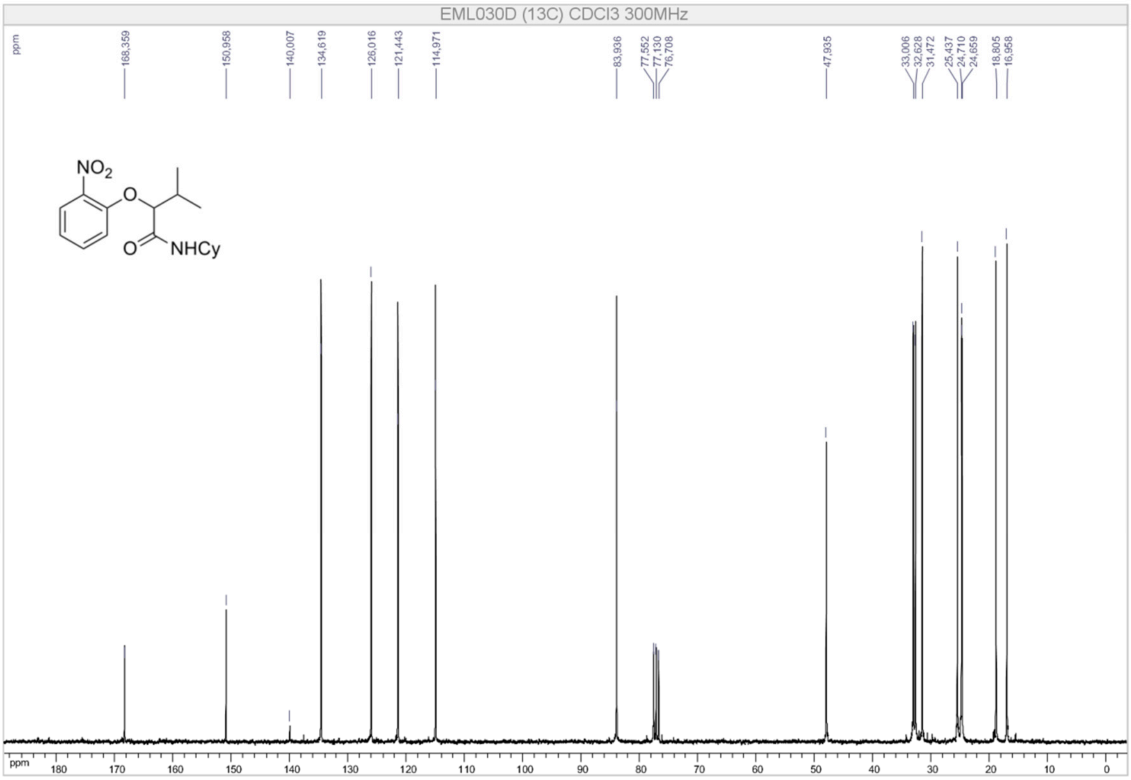

N-Cyclohexyl-3,3-dimethyl-2-(2-nitrophenoxy)butanamide (**2c**)

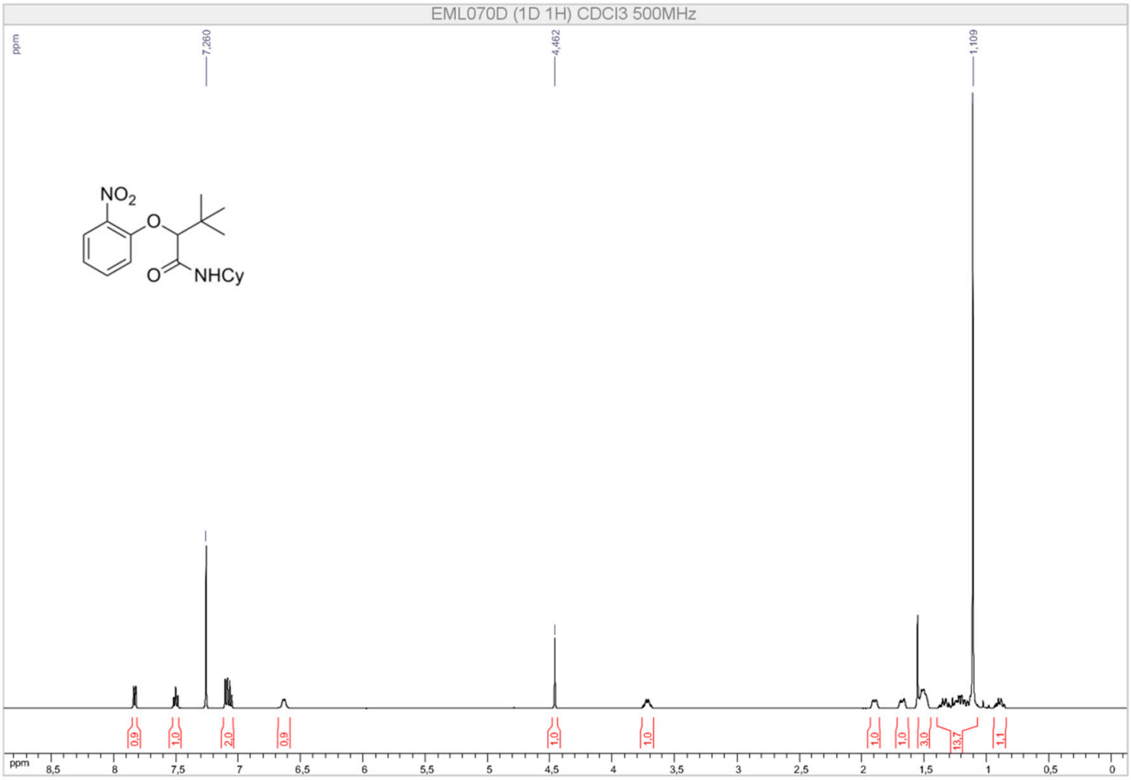

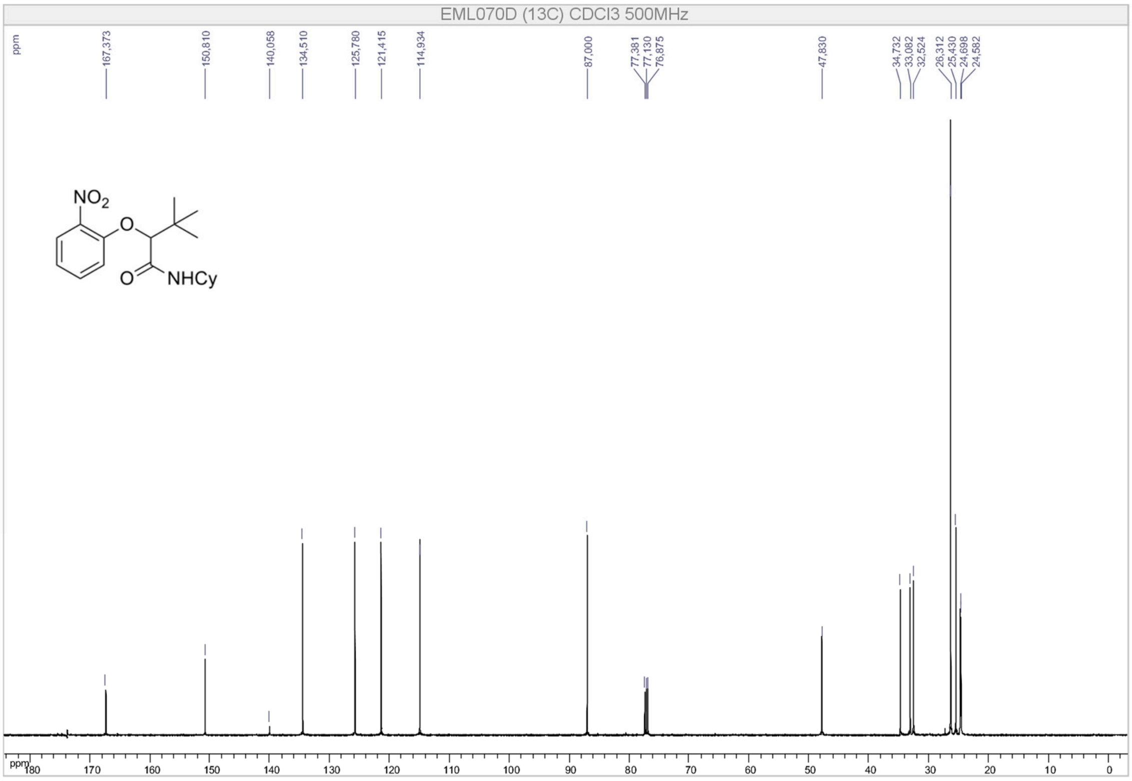

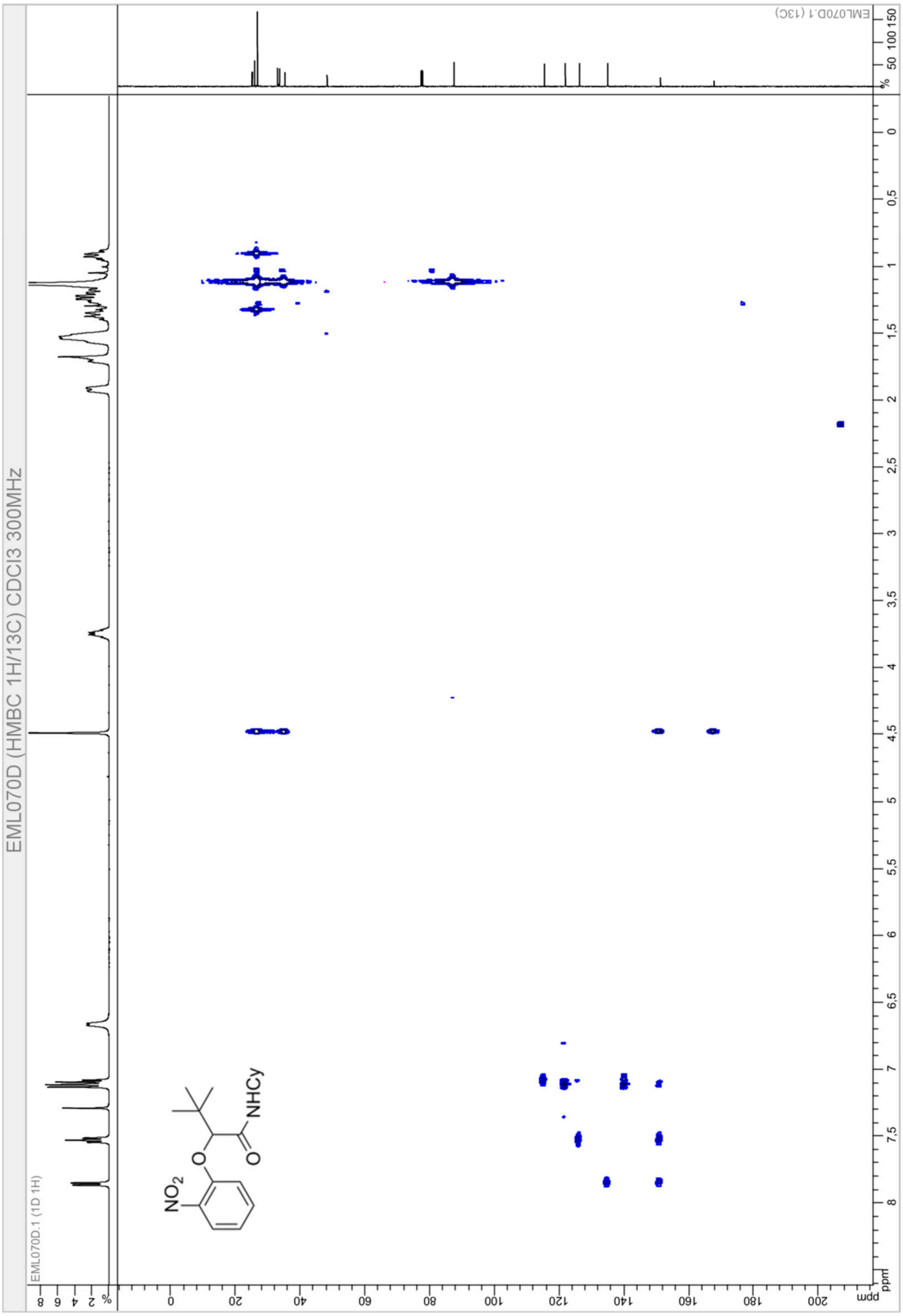
EML070D (HMBC 1H/13C) CDCl3 300MHz
EML070D.1 (13C)
EML070D.1 (1D 1H)
NO2
O
NHCy
ppm

N-Cyclohexyl-2-(2-nitrophenoxy)-4-phenylbutanamide (**2d**)

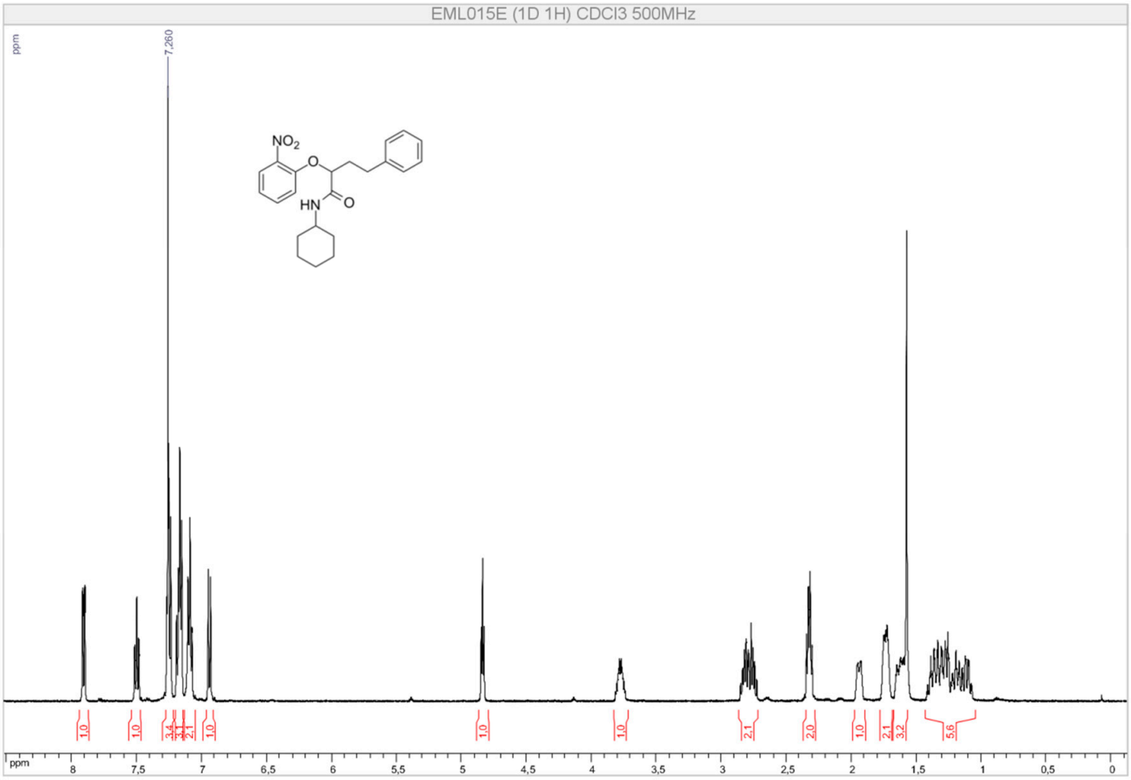

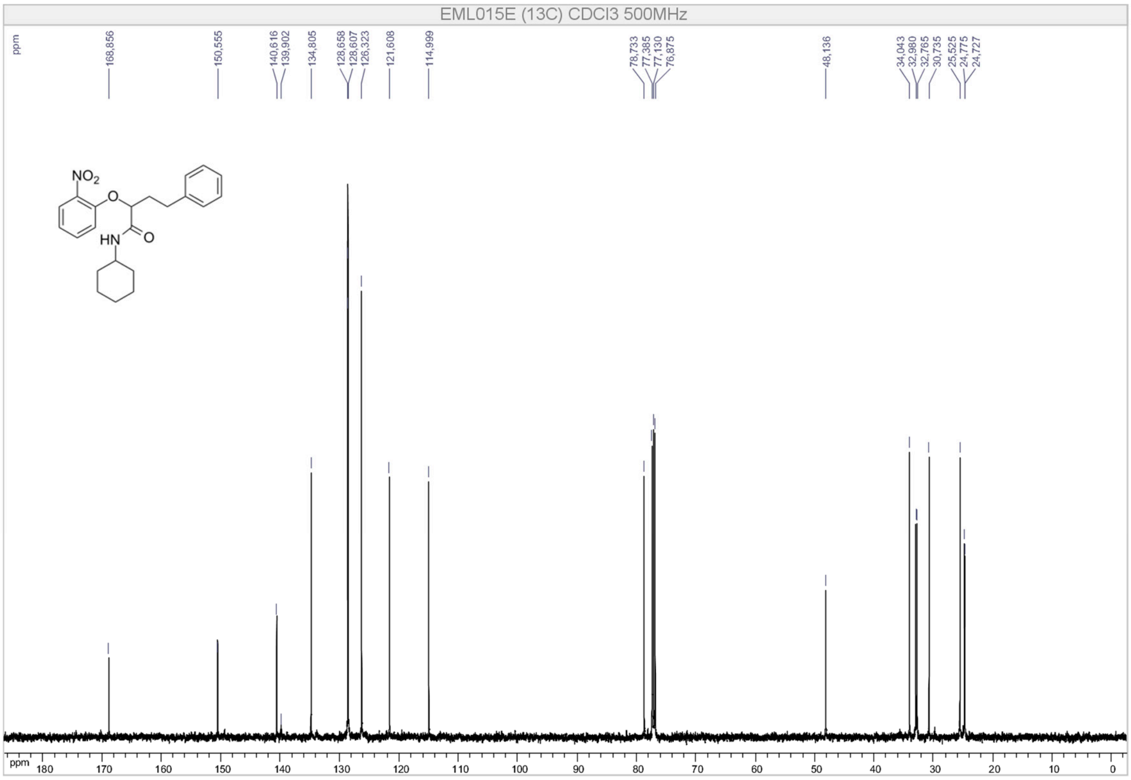

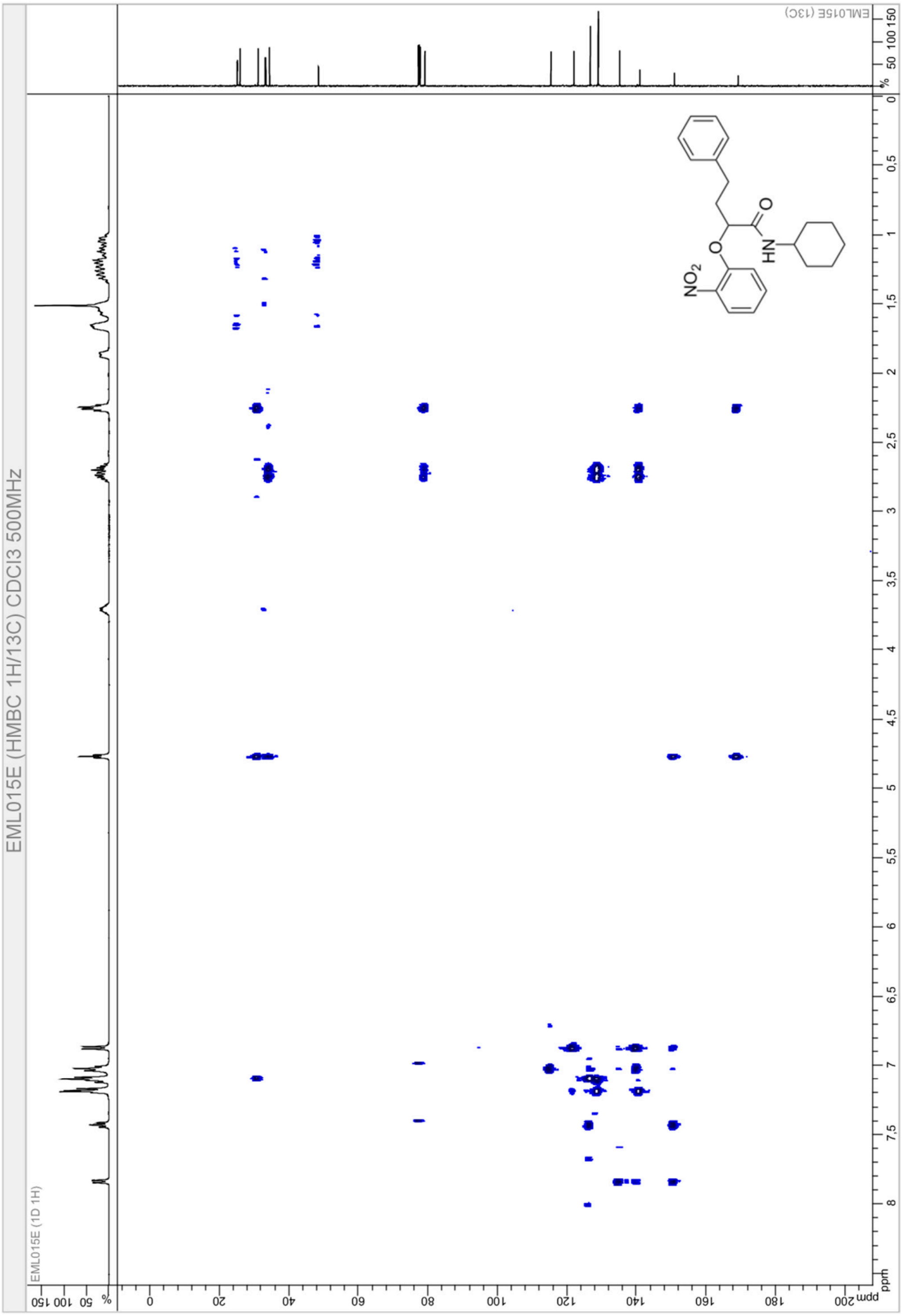
EML015E (HMBC 1H/13C) CDCl3 500MHz
EML015E (13C)
EML015E (1D 1H)
NO_2
O
O
HN
ppm

N-Cyclohexyl-2-(3-fluorophenyl)-2-(2-nitrophenoxy)acetamide (**2e**)

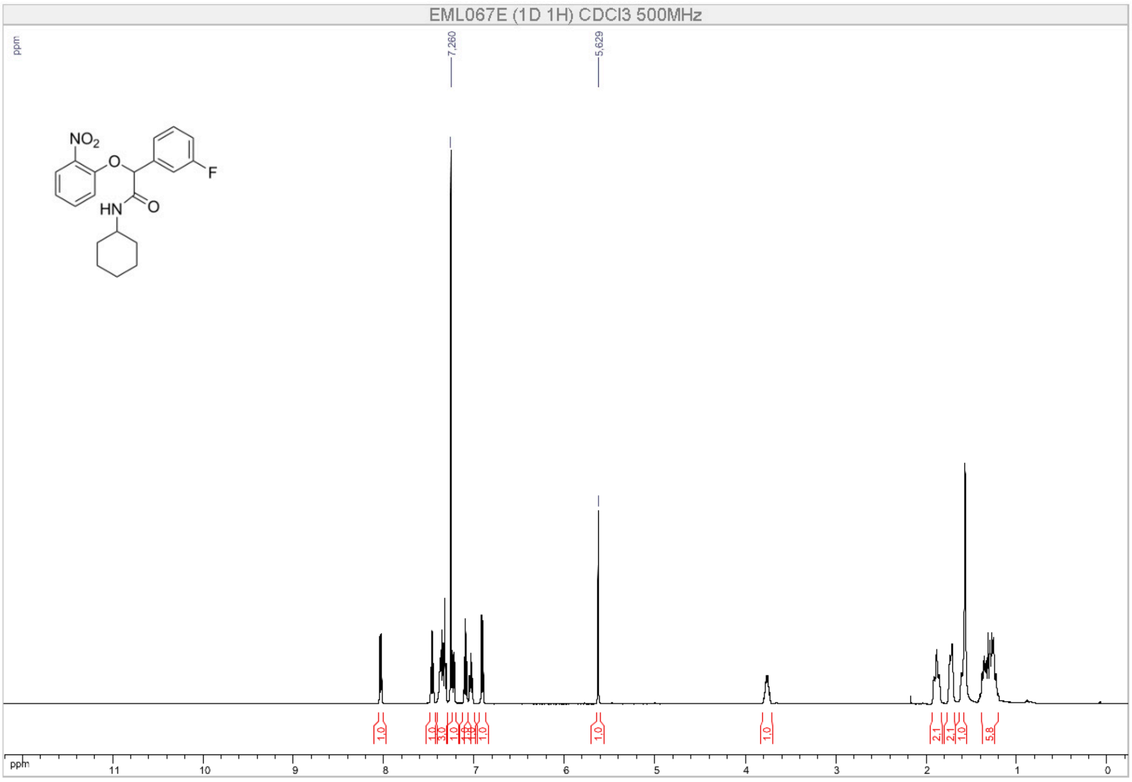

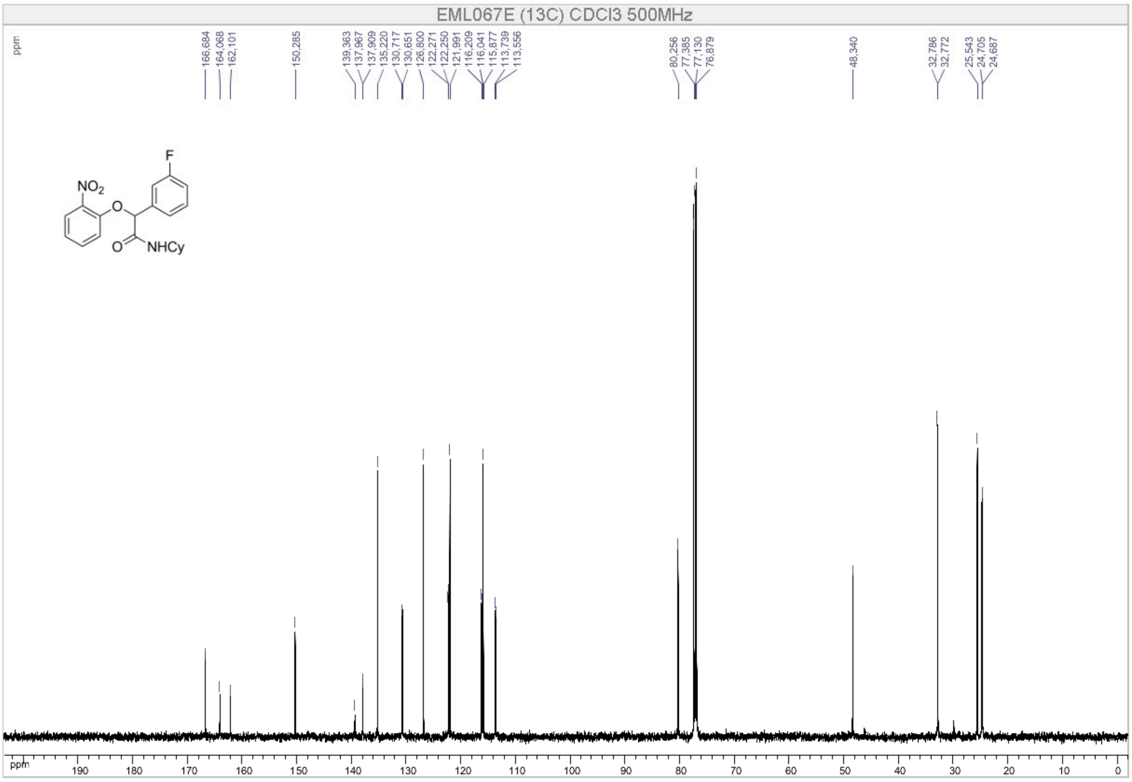

2-(4-Chlorophenyl)-N-cyclohexyl-2-(2-nitrophenoxy)acetamide (**2f**)

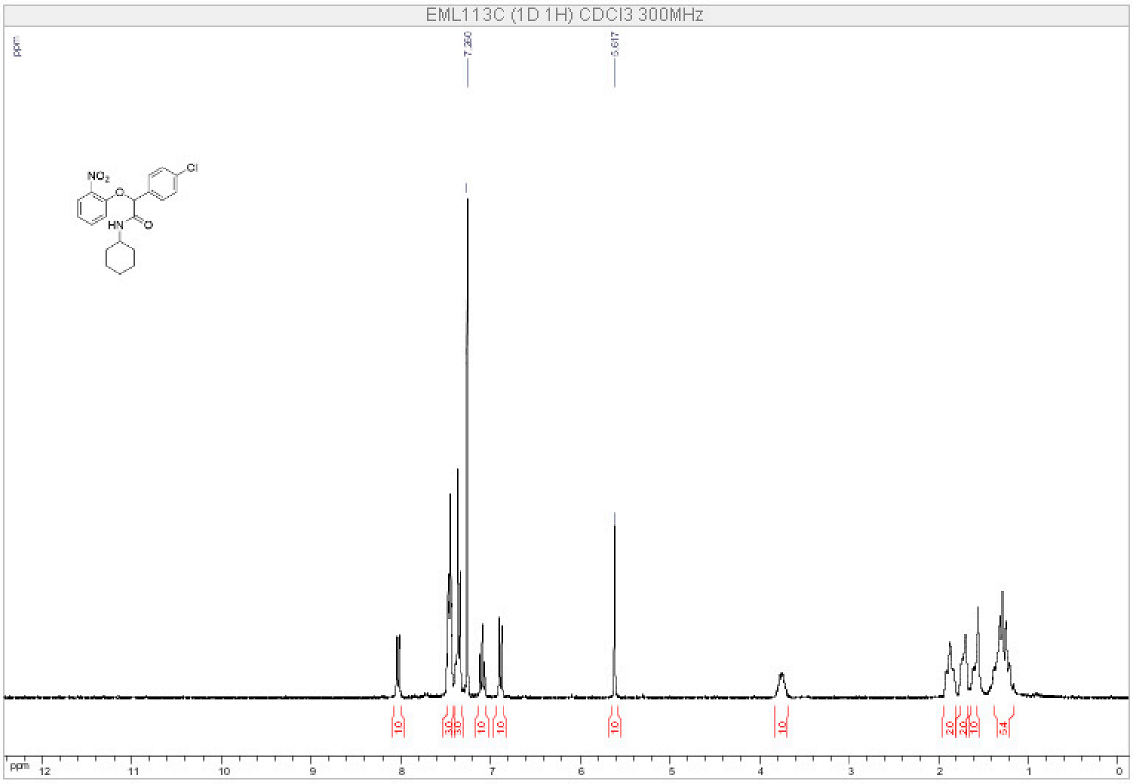

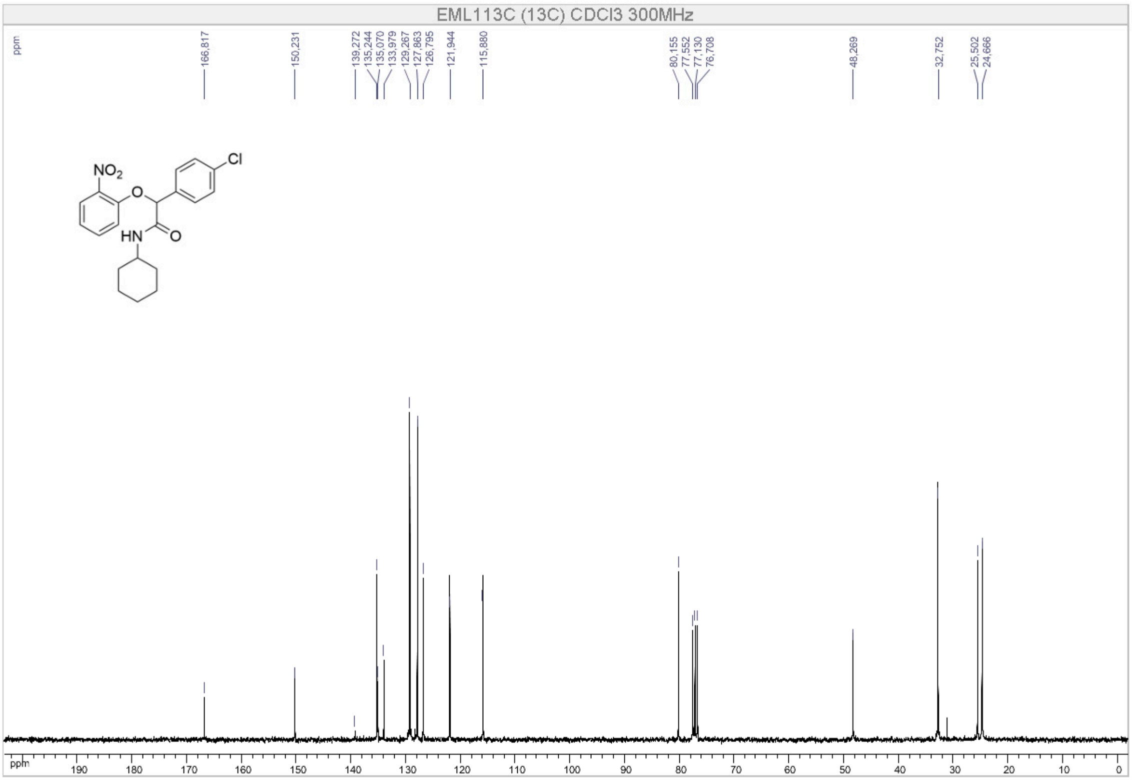

N-(4-Methoxybenzyl)-3,3-dimethyl-2-(4-methyl-2-nitrophenoxy)hex-5-enamide (**2g**)

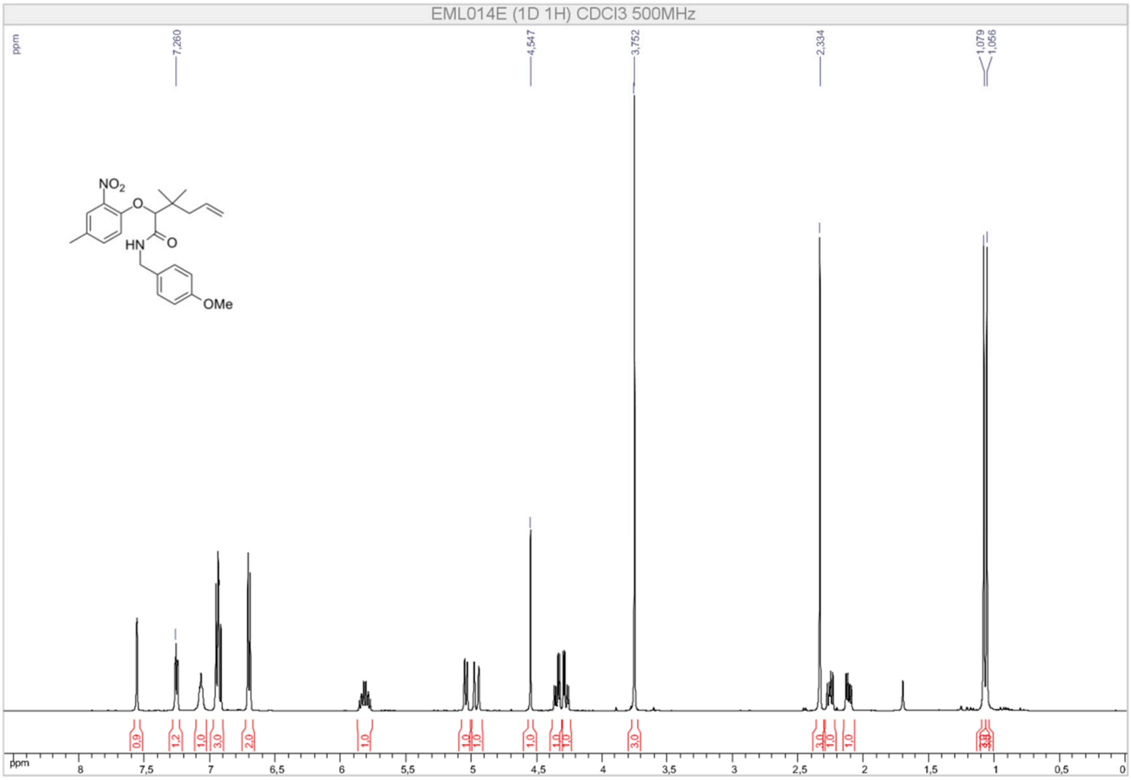

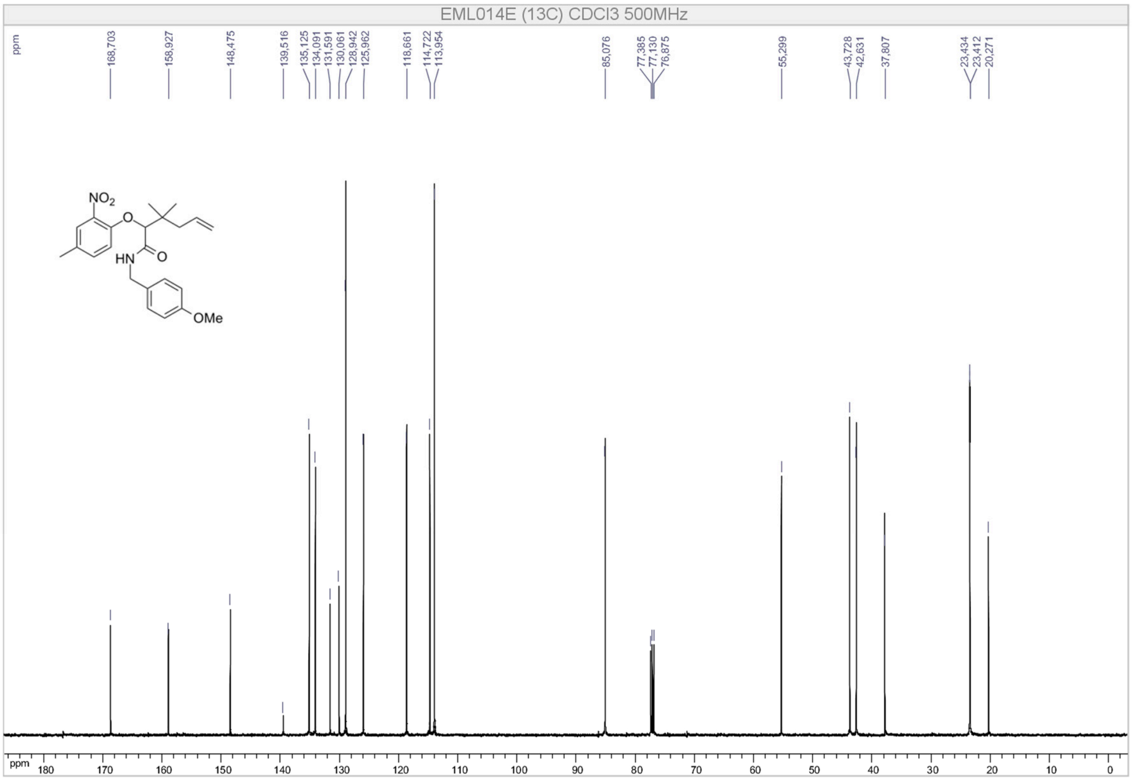

N-(4-Chlorobenzyl)-4-methyl-2-(4-methyl-2-nitrophenoxy)pentanamide (**2h**)

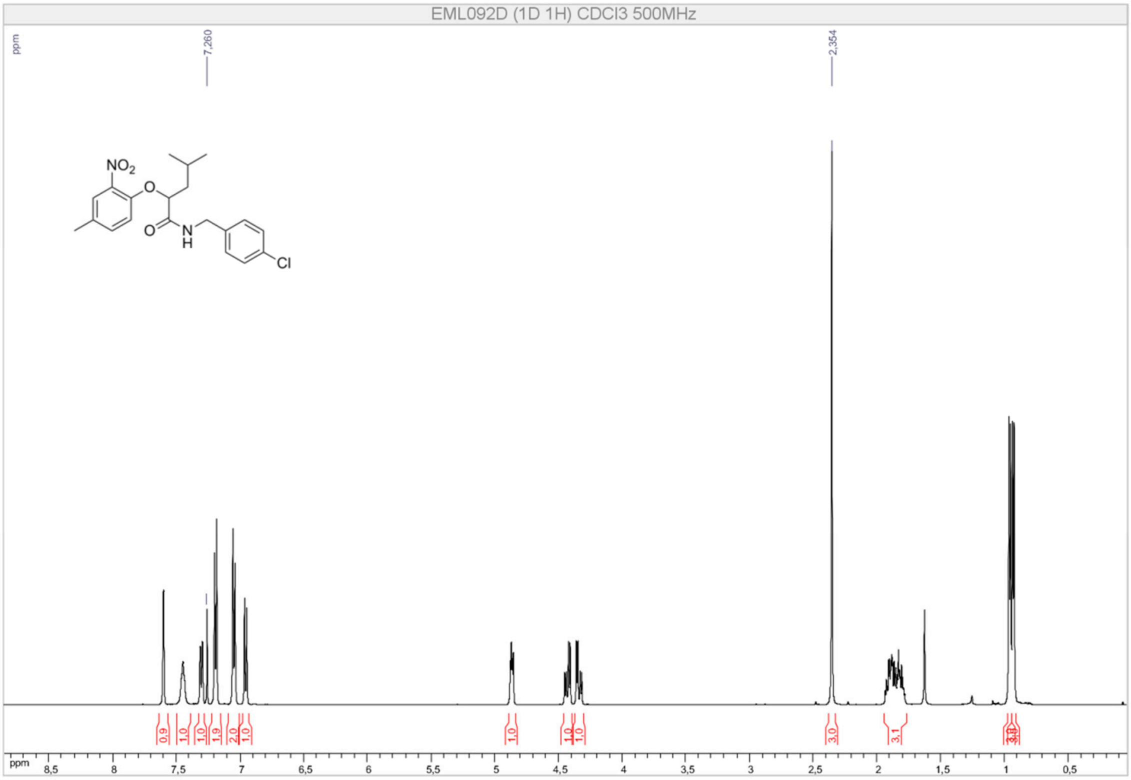

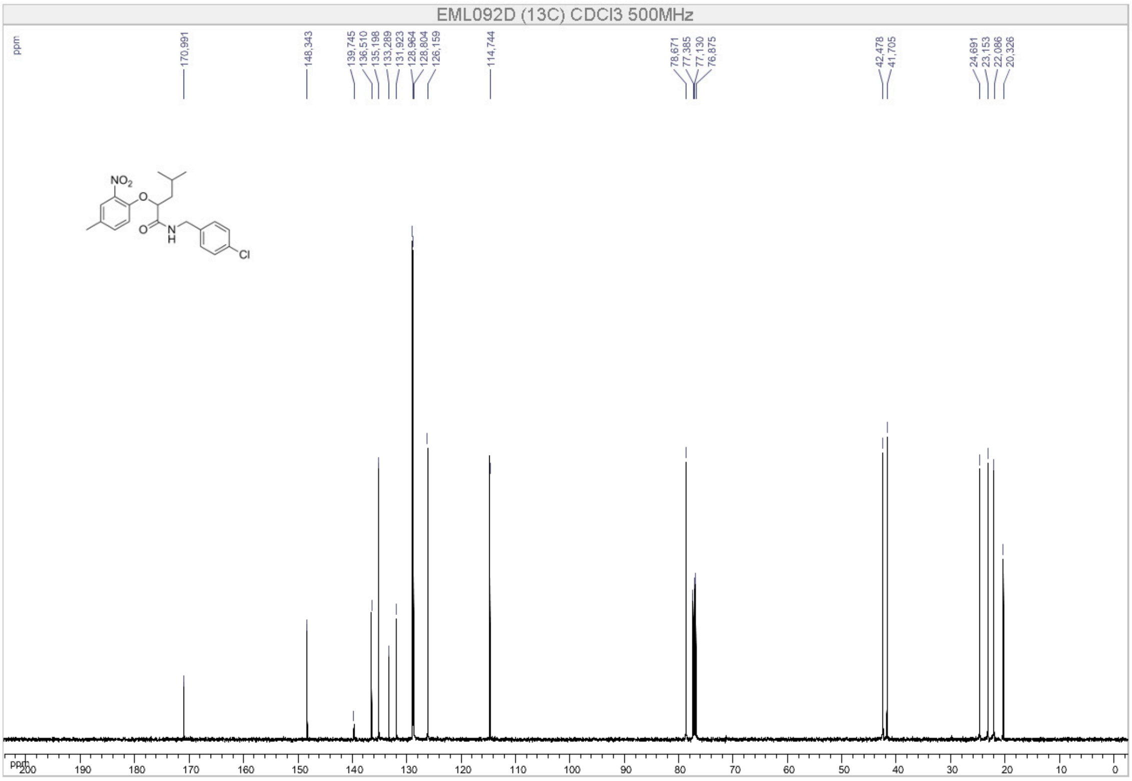

N-Cyclohexyl-2-(4-methoxyphenyl)-2-(4-methyl-2-nitrophenoxy)acetamide (**2i**)

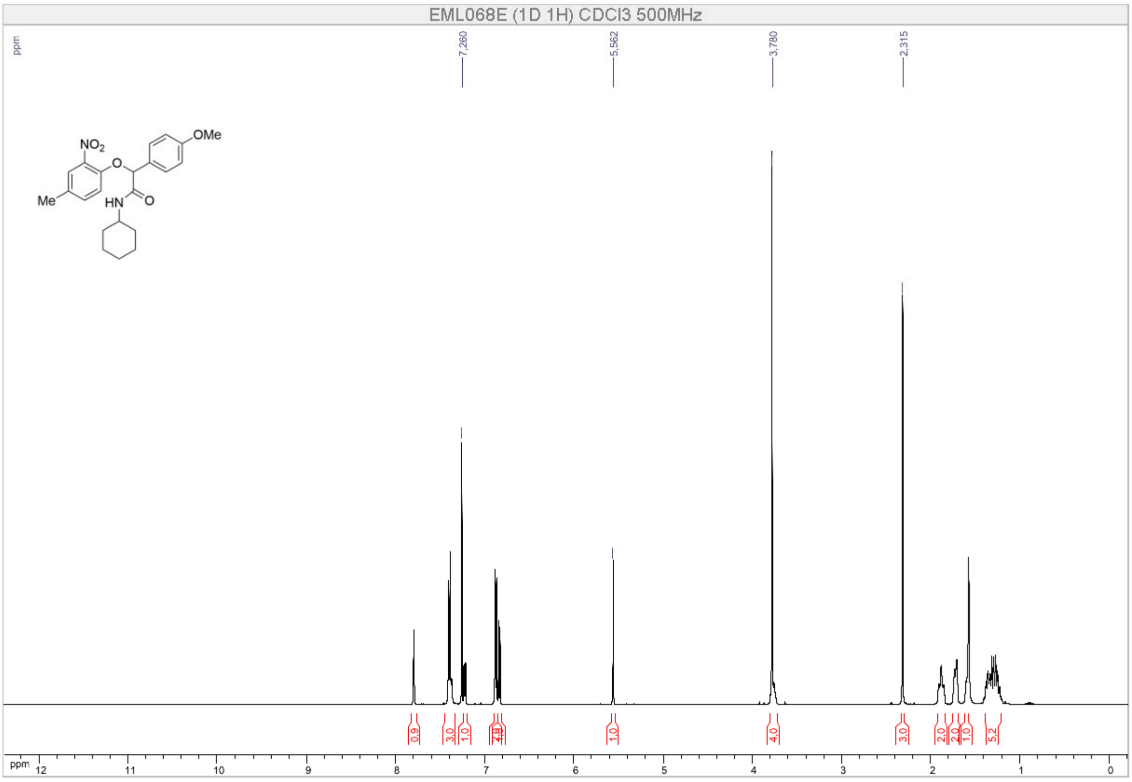

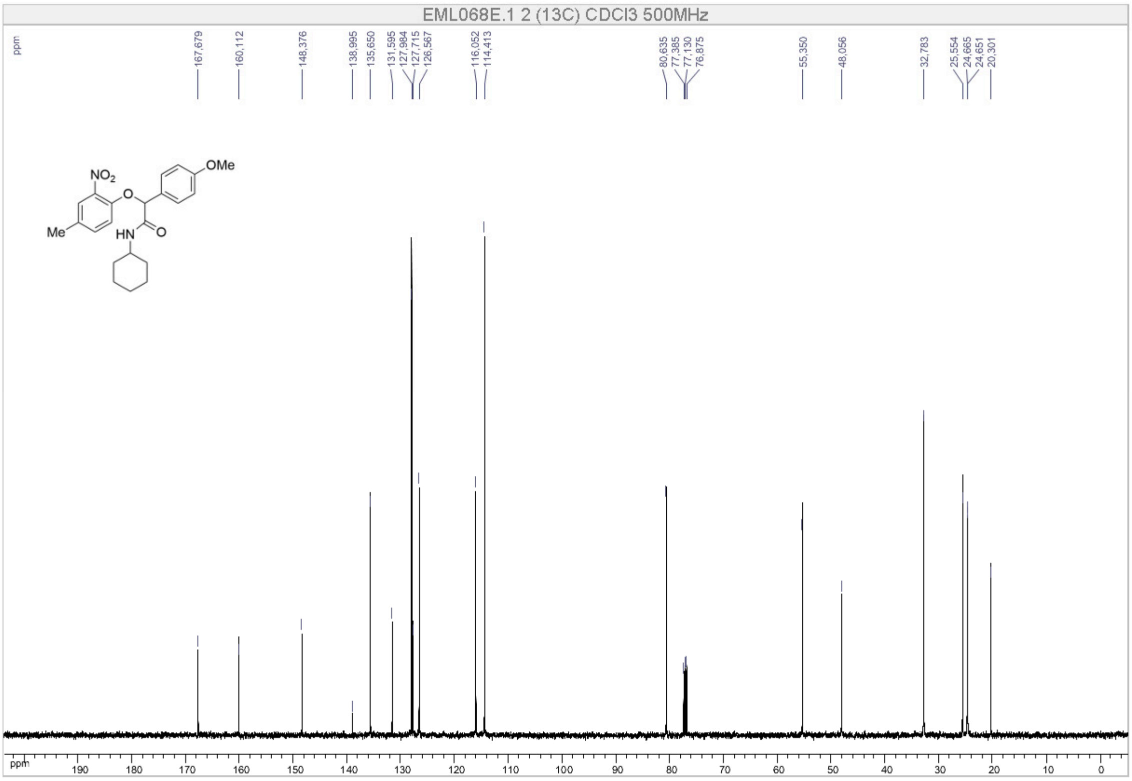

2-(4-Methoxy-2-nitrophenoxy)-3,3-dimethyl-N-phenethylbutanamide (**2j**)

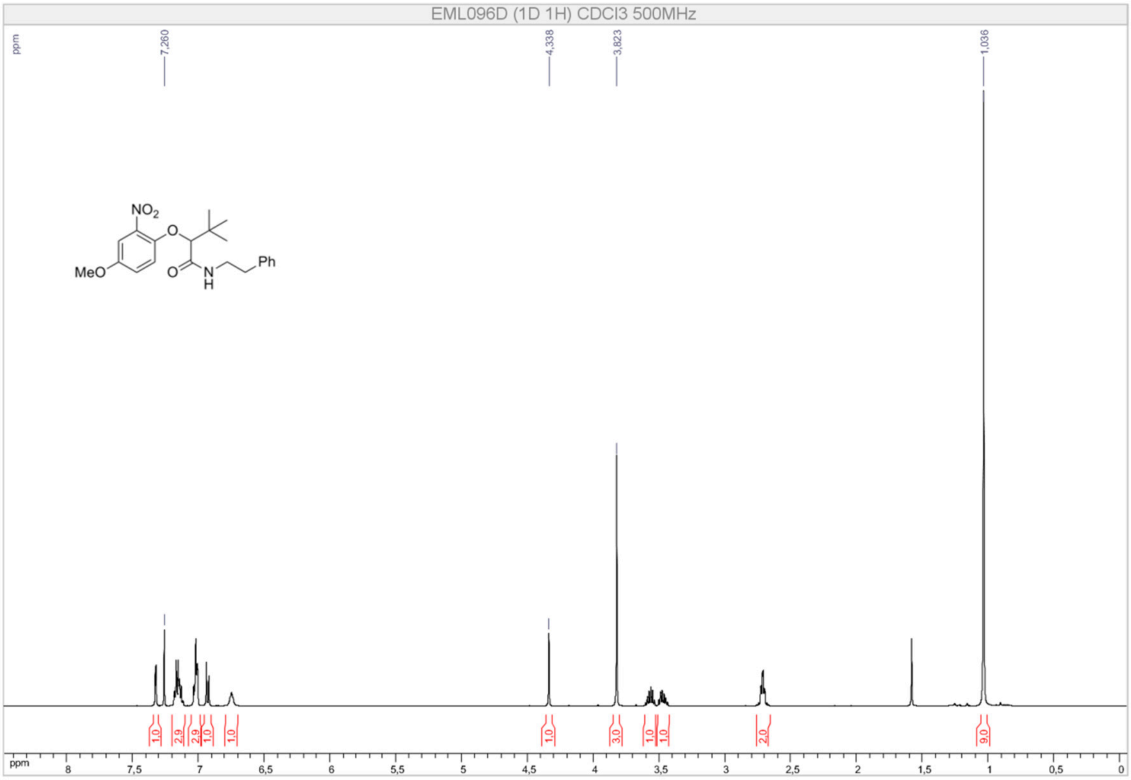

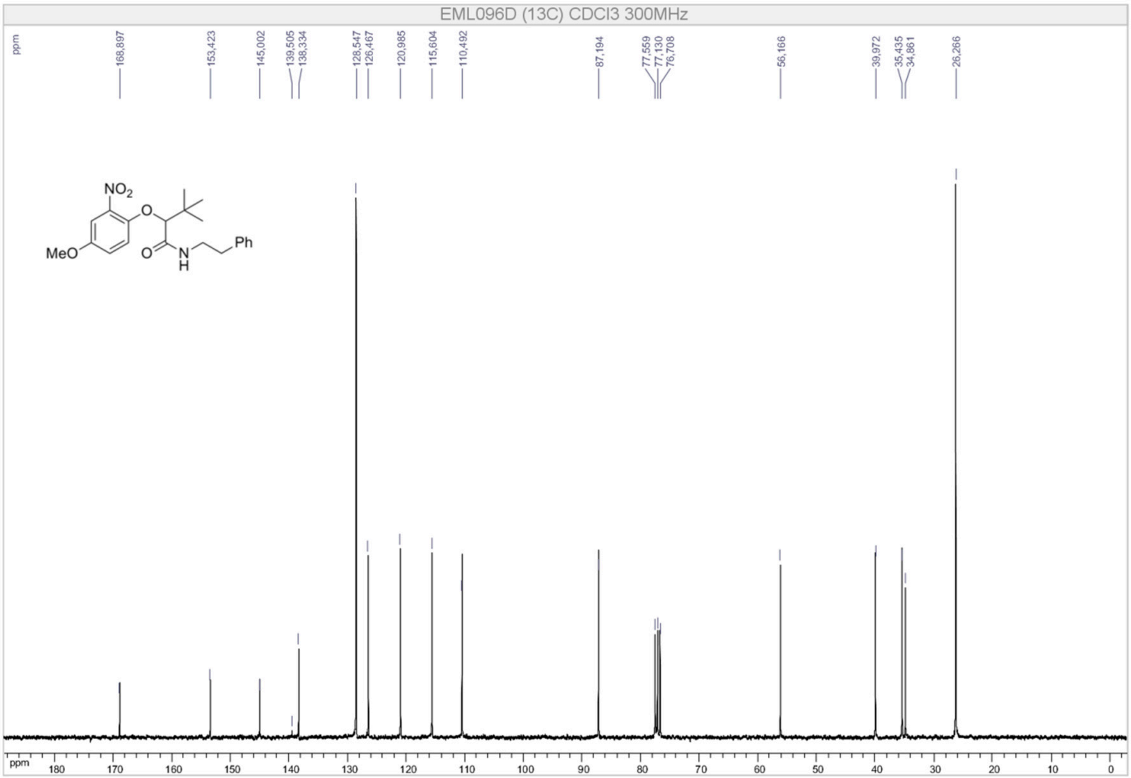

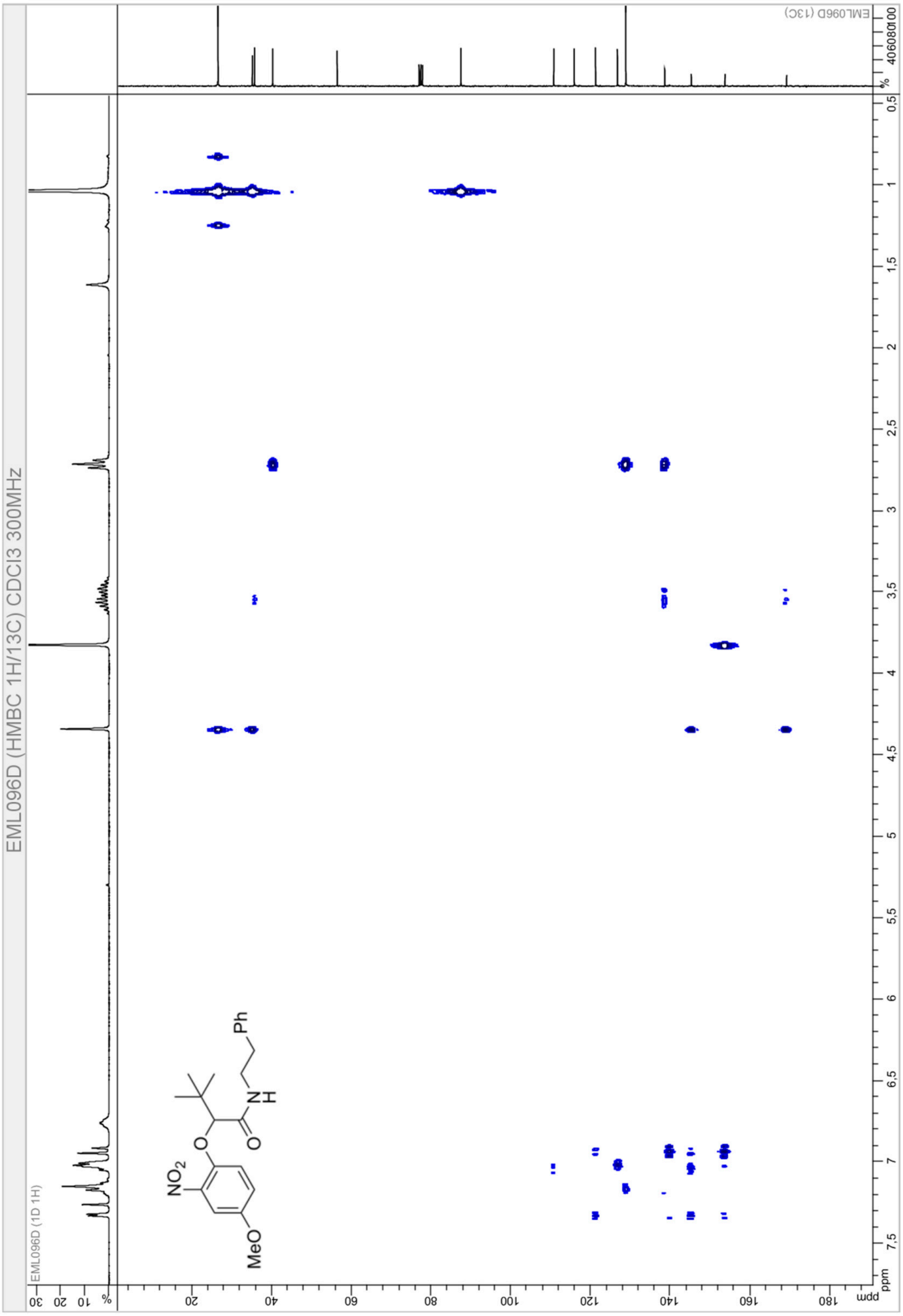
EML096D (HMBC 1H/13C) CDCl3 300MHz
EML096D (13C)
EML096D (1D 1H)
NO2
MeO
Ph
N
H
O
ppm

2-(2-Allyl-6-nitrophenoxy)-N-cyclohexyl-3-methylbutanamide (**2k**)

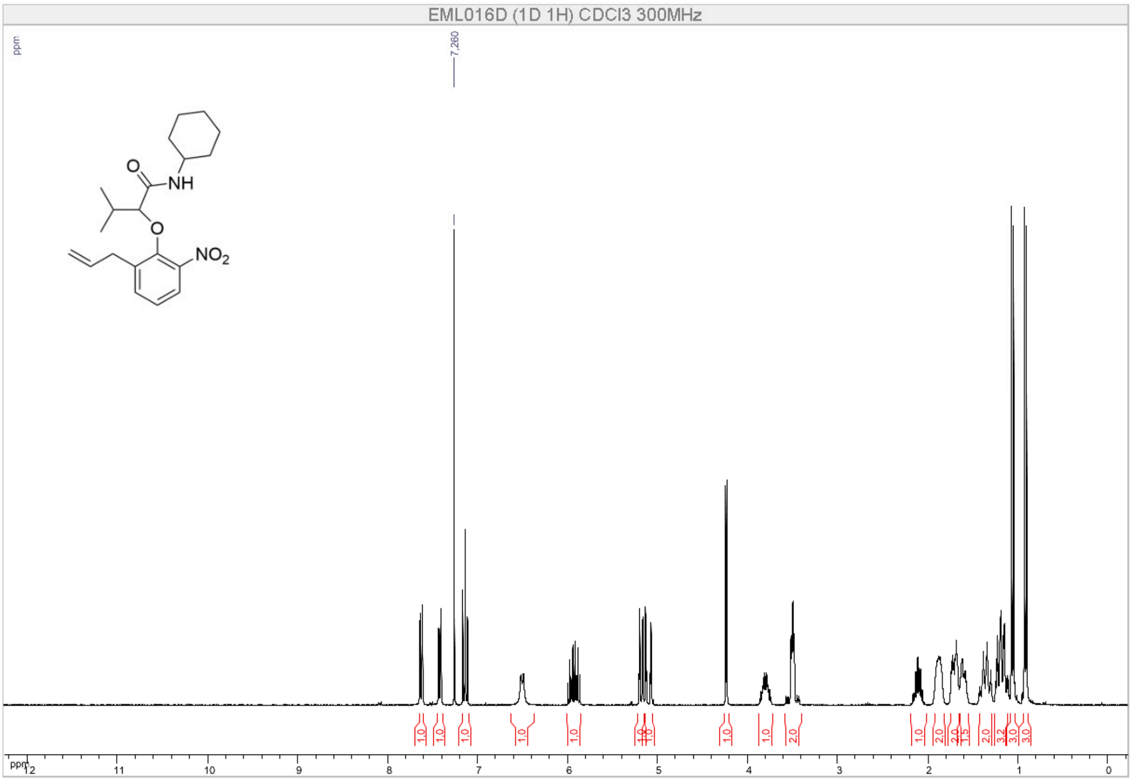

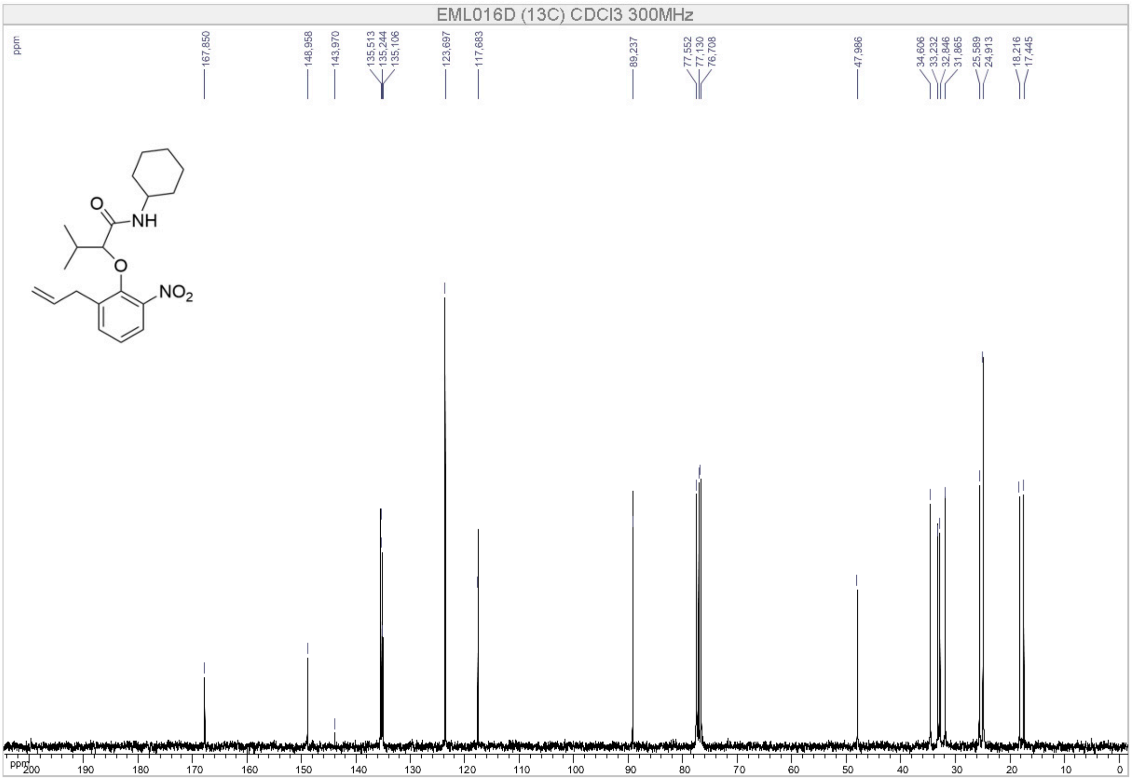

N-Cyclohexyl-2-(4-methoxy-2-nitrophenoxy)-3-methylbutanamide (**2l**)

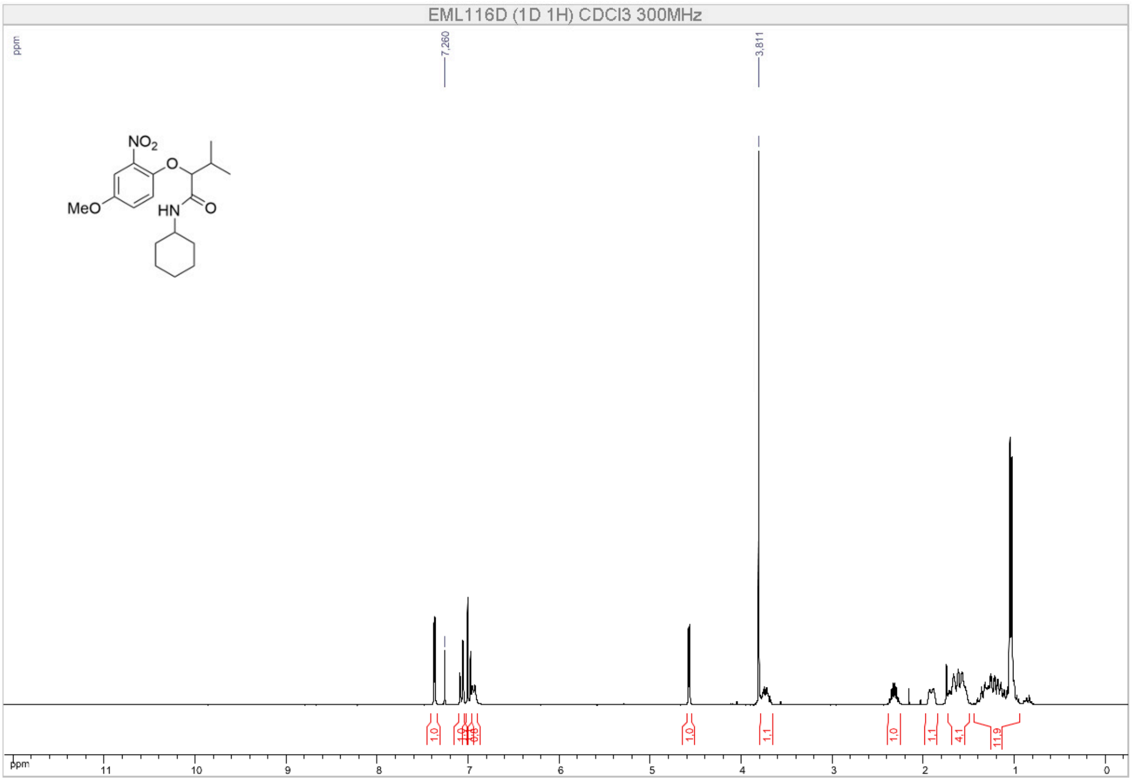

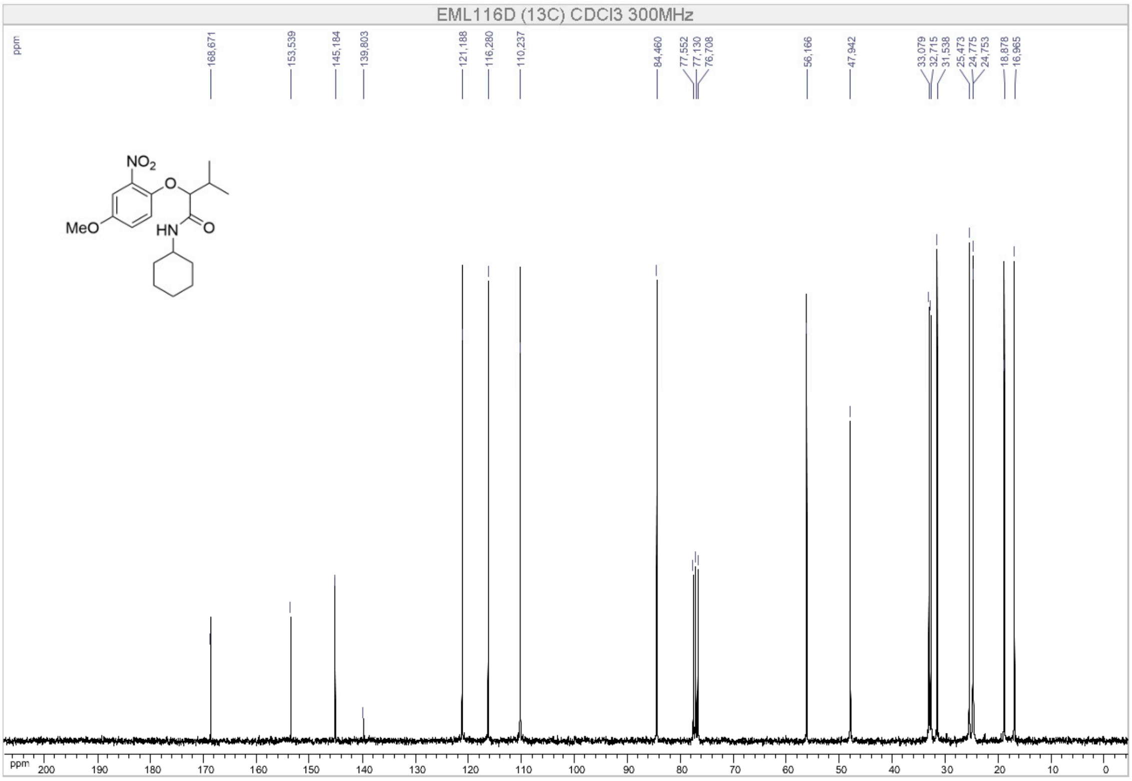

N-Cyclohexyl-2-(4-methoxy-2-nitrophenoxy)-3,3-dimethylbutanamide (**2m**)

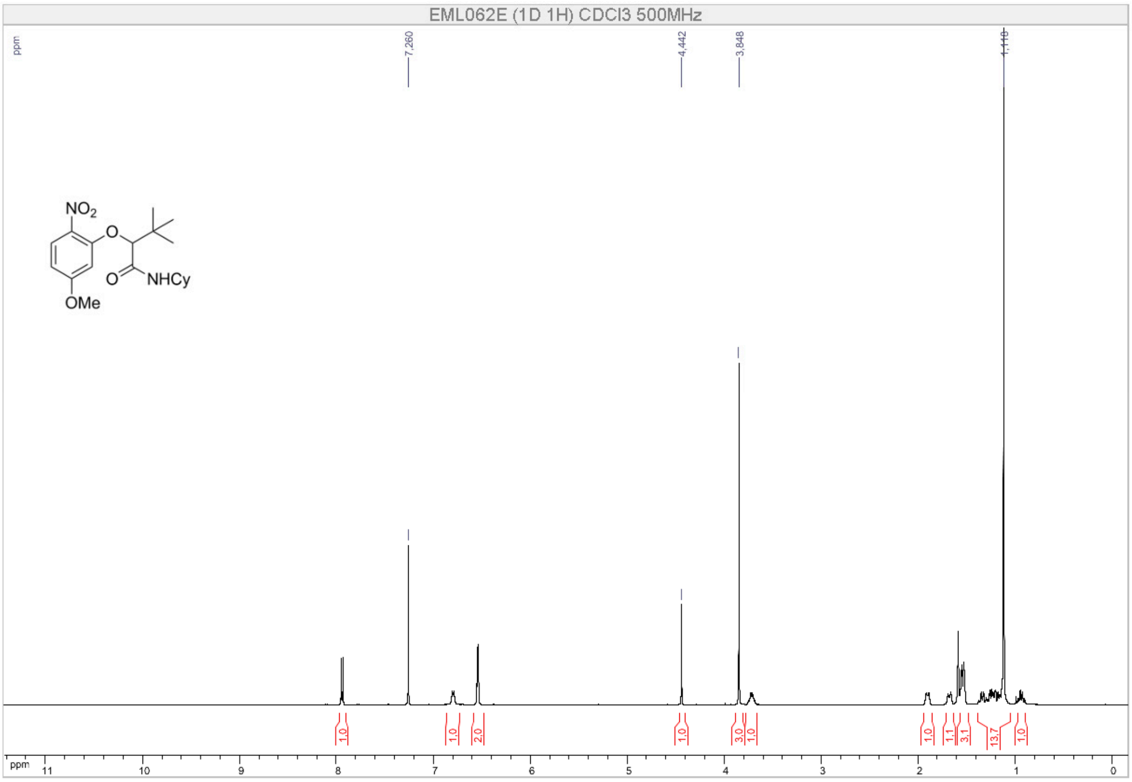

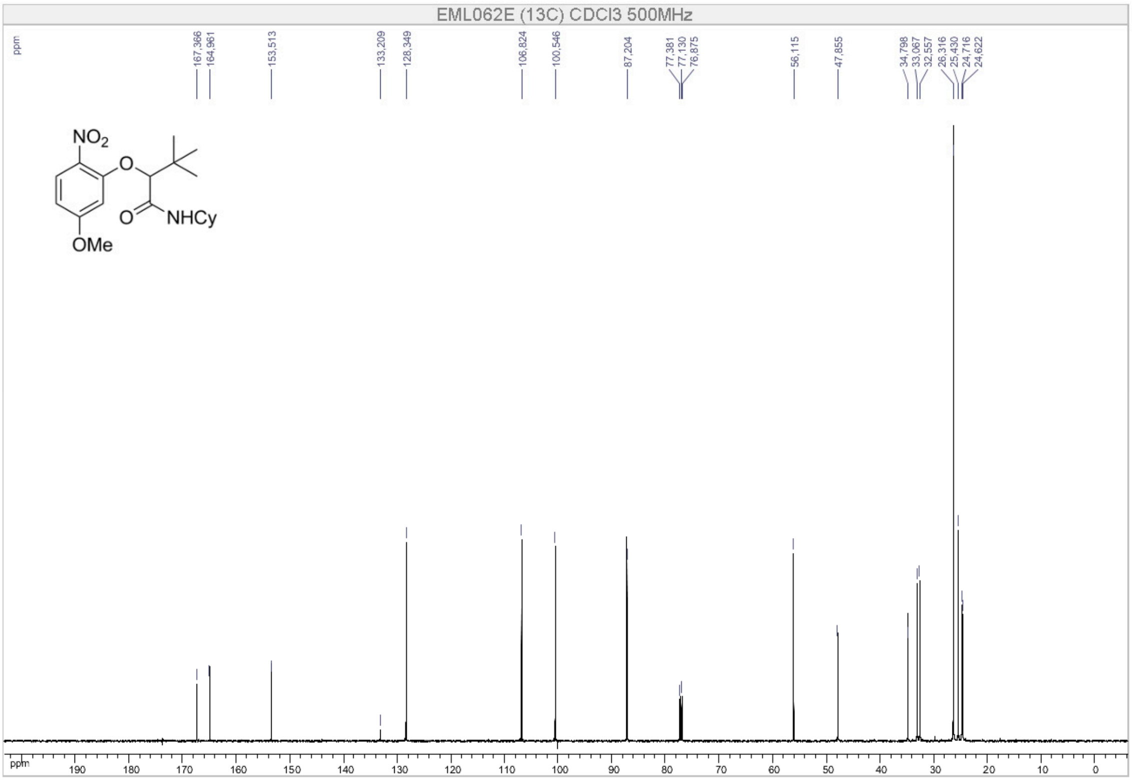

2-(4-Bromo-2-nitrophenoxy)-3,3-dimethyl-N-phenethylbutanamide (**2n**)

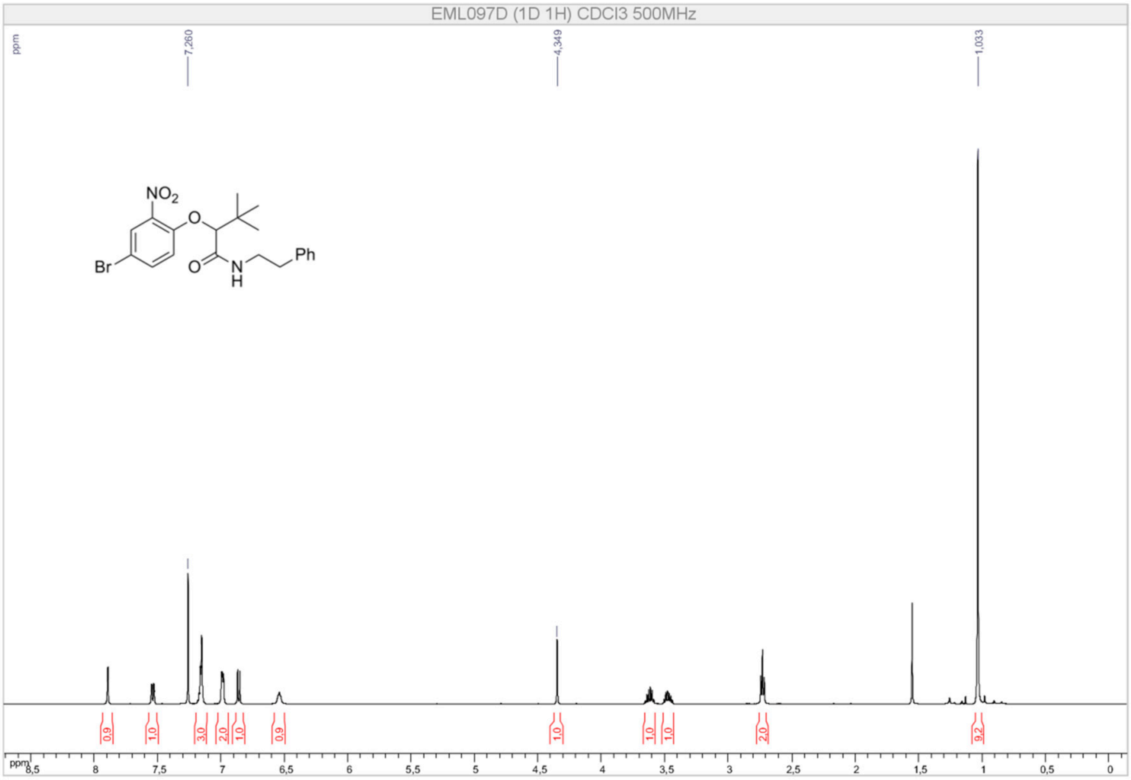

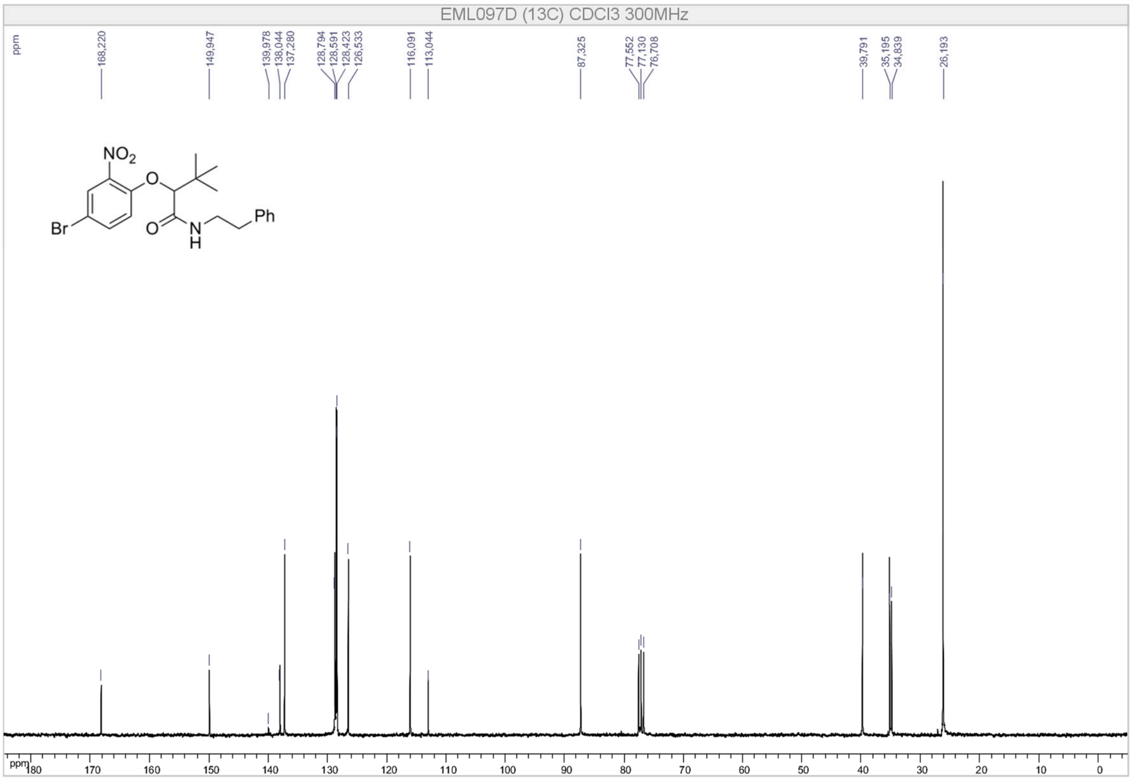

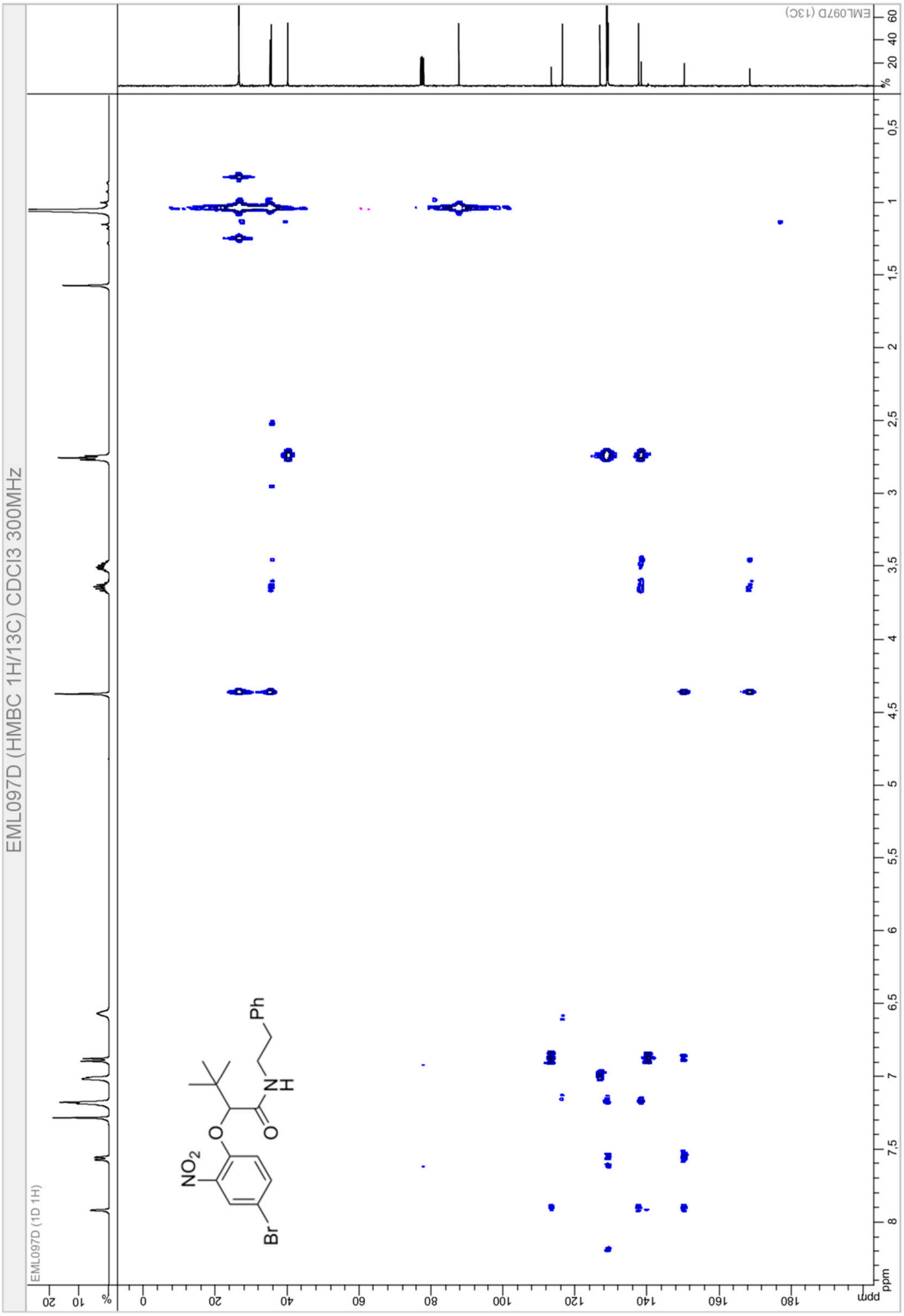
EML097D (HMBC 1H/13C) CDCl3 300MHz
EML097D (13C)
EML097D (1D 1H)
ppm
%
NO2
O
O
N
H
Ph
Br

2-(4-Bromo-2-nitrophenoxy)-N-(3,4-dimethoxyphenethyl)-3-methylbutanamide (**2o**)

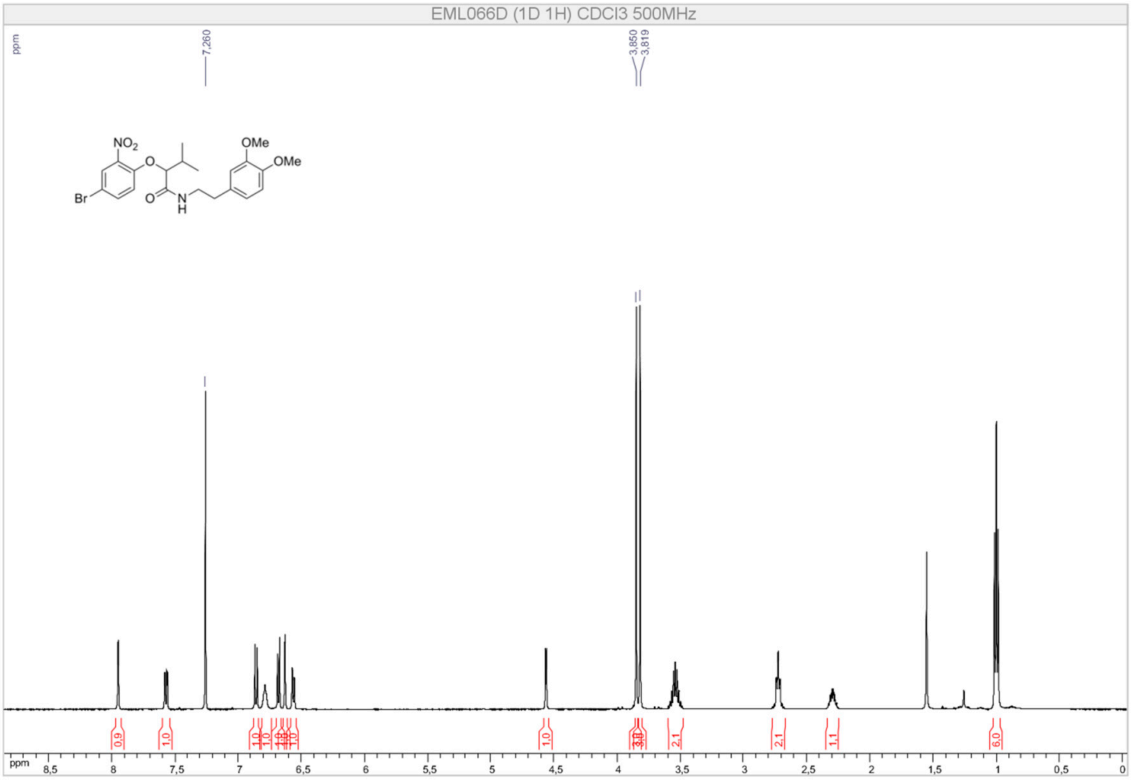

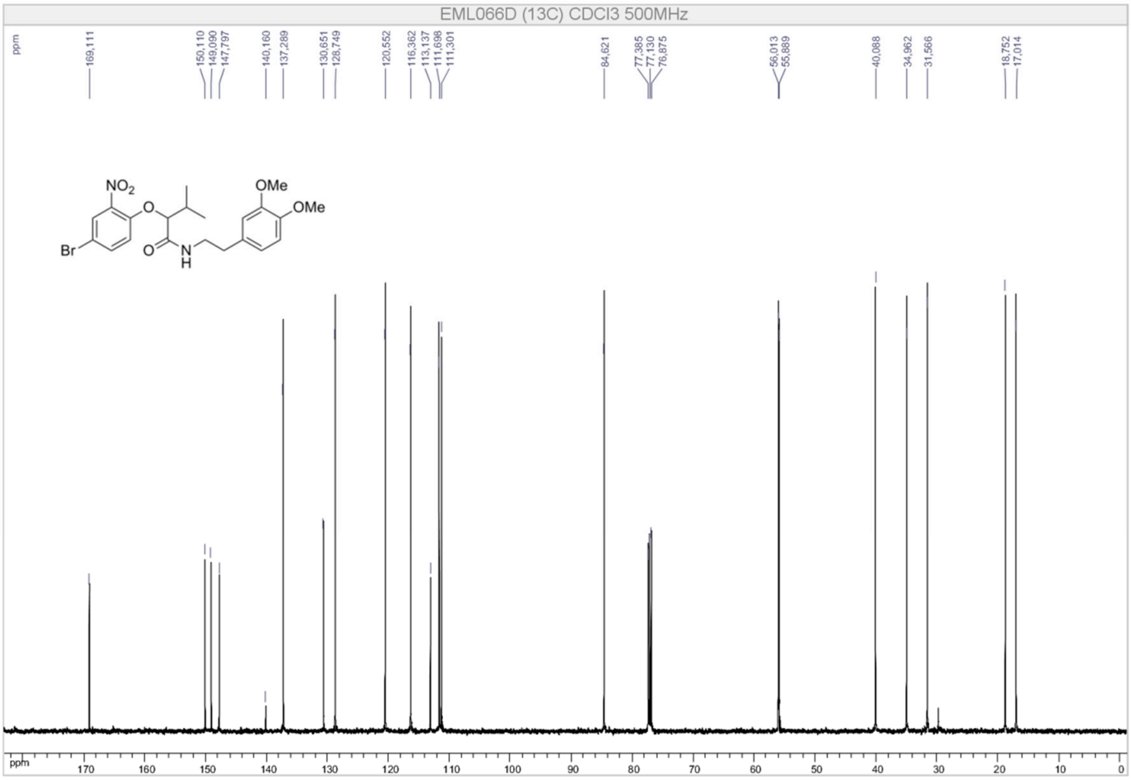

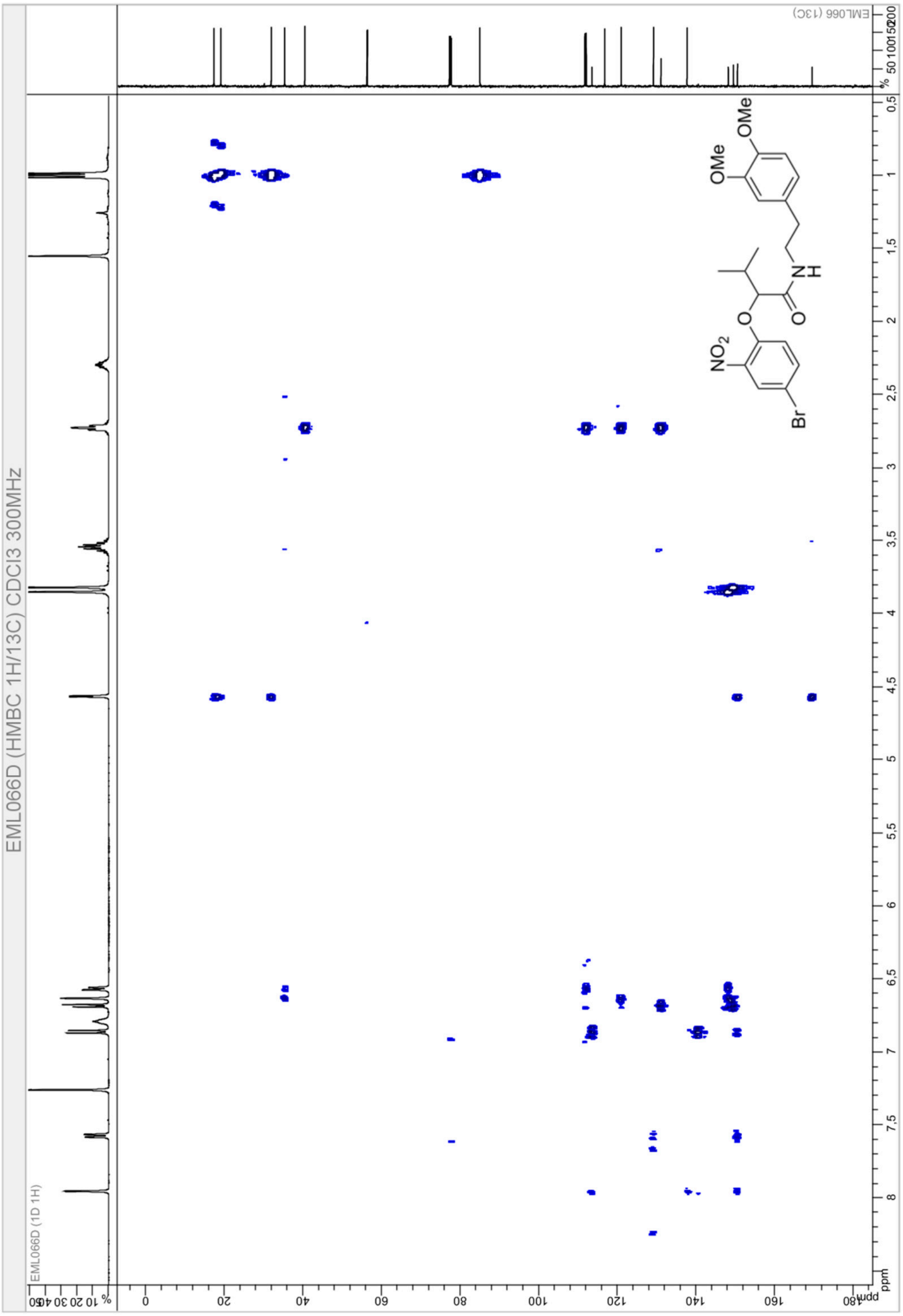
EML066D (HMBC 1H/13C) CDCl3 300MHz
EML066D (1D 1H)
EML066 (13C)
OMe
OMe
NO_2
O
O
N
H
Br
ppm

N-(Tert-butyl)-2-(4-chloro-2-nitrophenoxy)butanamide (**2p**)

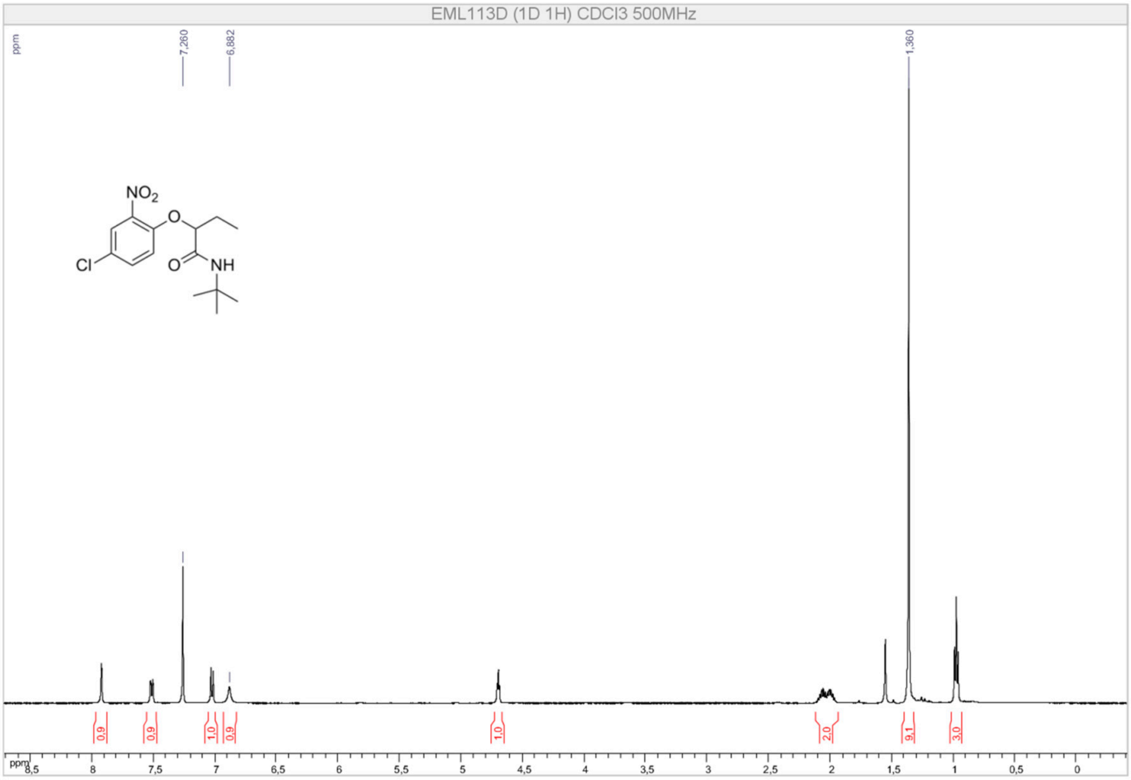

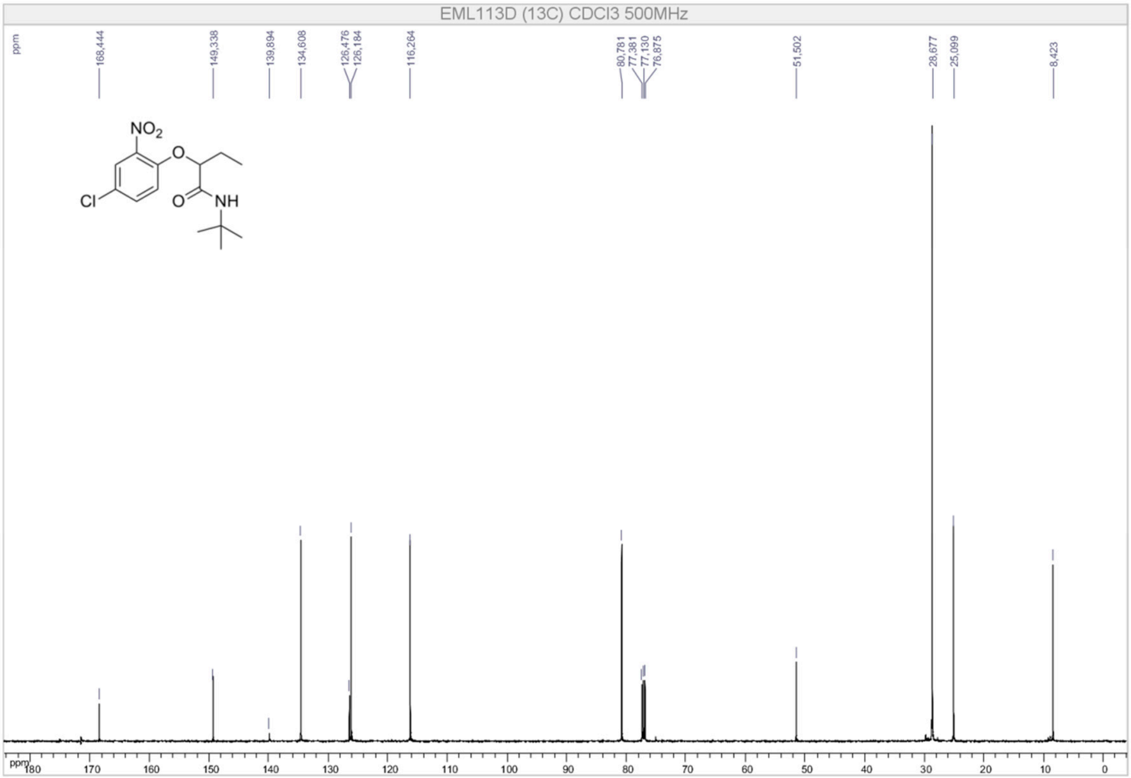

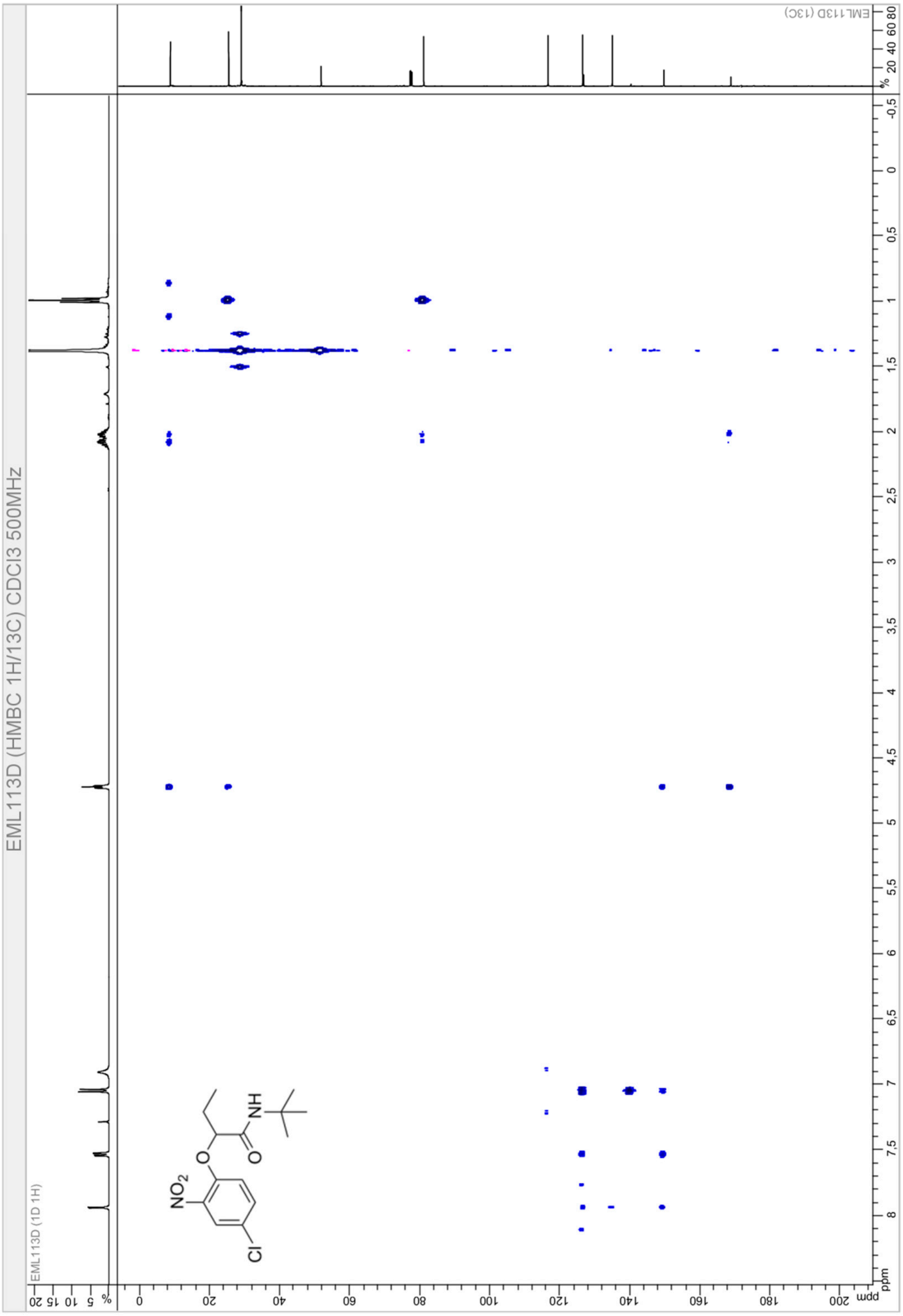
EML113D (HMBC 1H/13C) CDCl3 500MHz
EML113D (13C)
EML113D (1D 1H)
NO2
O
HN
O
Cl
ppm

N-(Tert-butyl)-2-(4-chloro-2-nitrophenoxy)-3-methylbutanamide (**2q**)

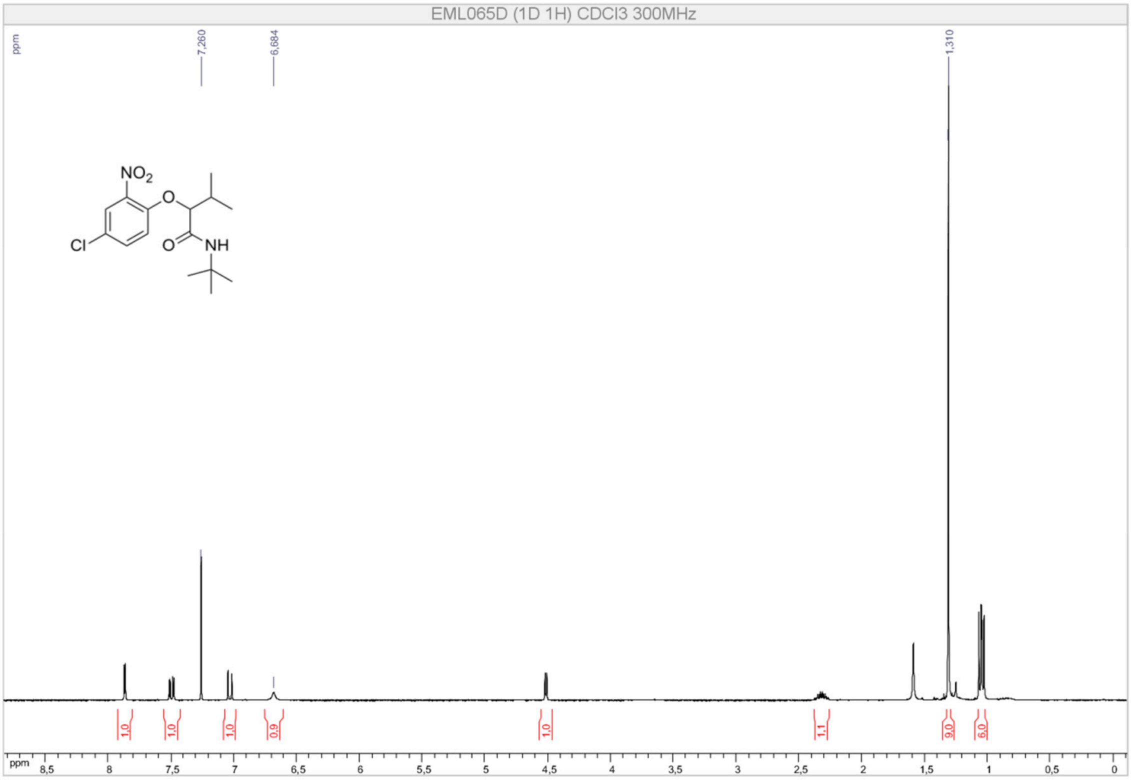

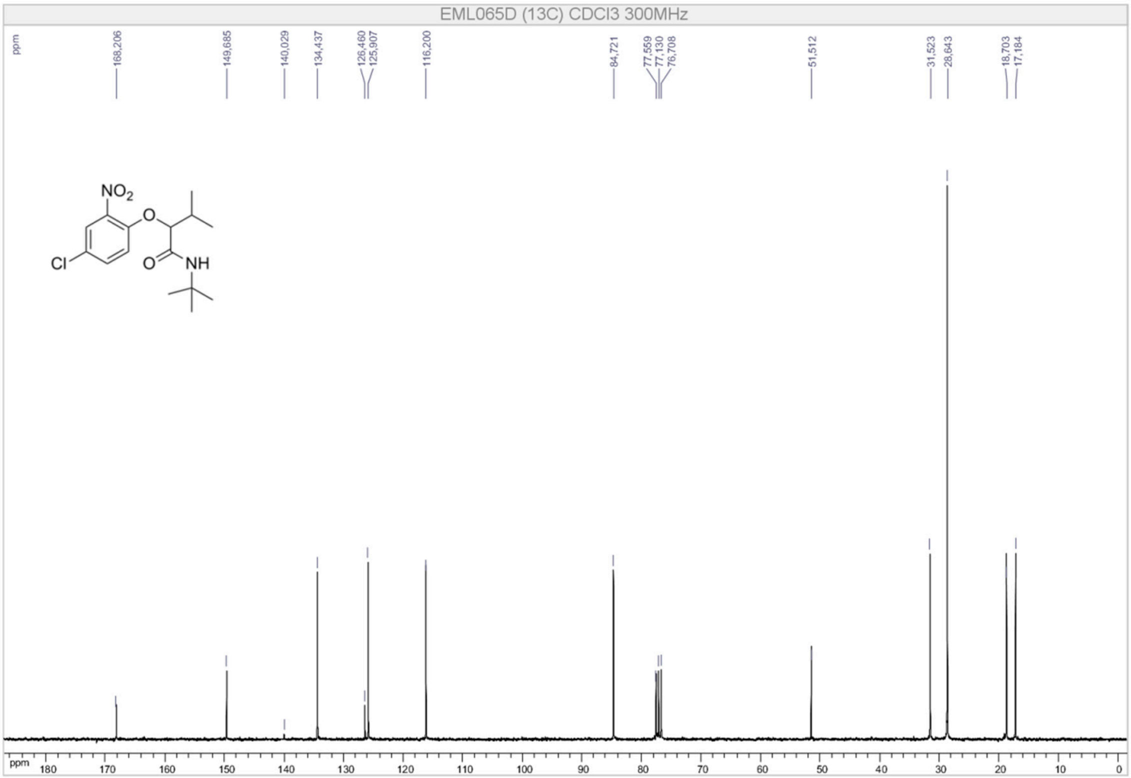

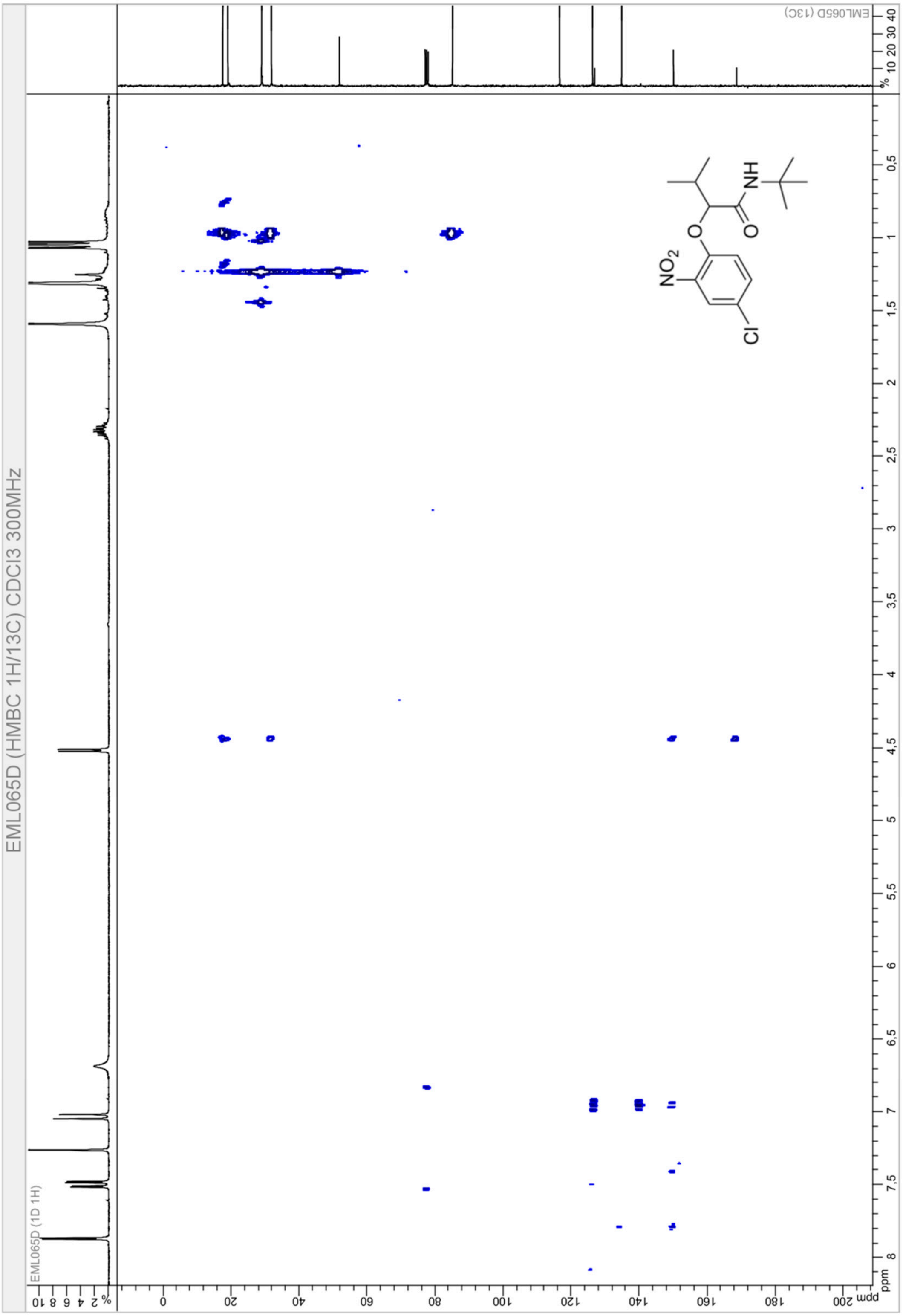
EML065D (HMBC 1H/13C) CDCl3 300MHz
EML065D (13C)
EML065D (1D 1H)
ppm
NO2
O
NH
Cl

2-(4-Chloro-2-nitrophenoxy)-N-(4-chlorobenzyl)-3,3-dimethylbutanamide (**2r**)

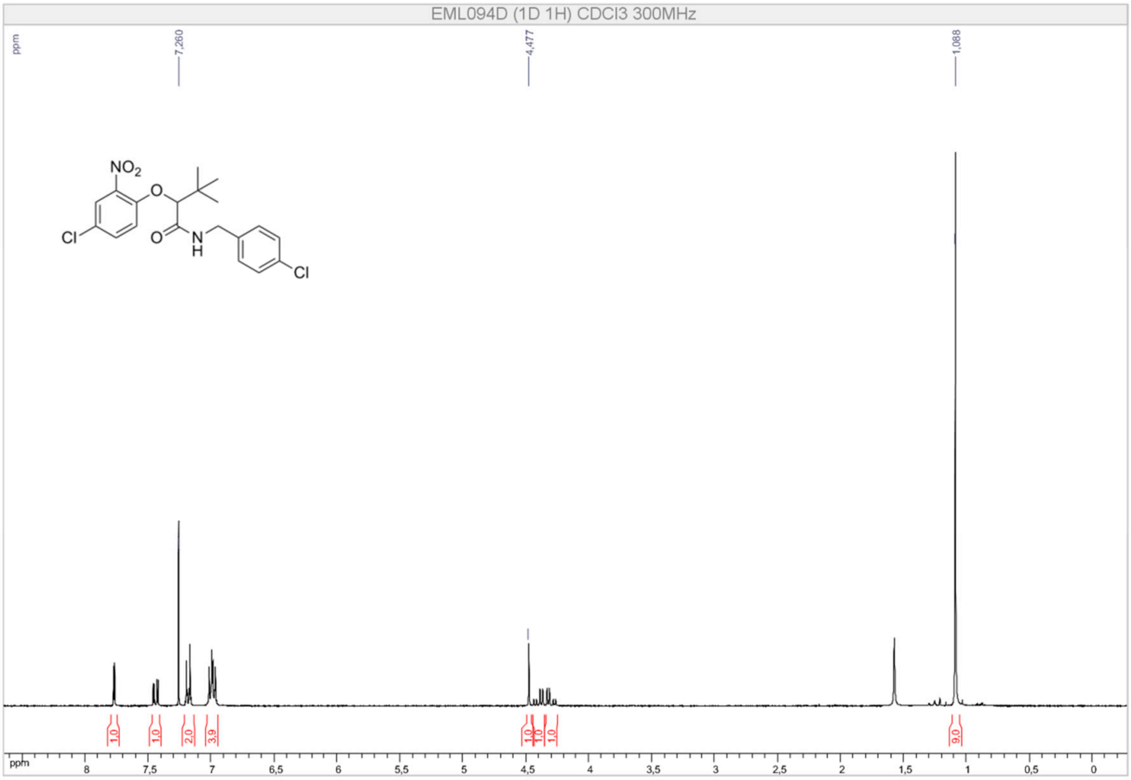

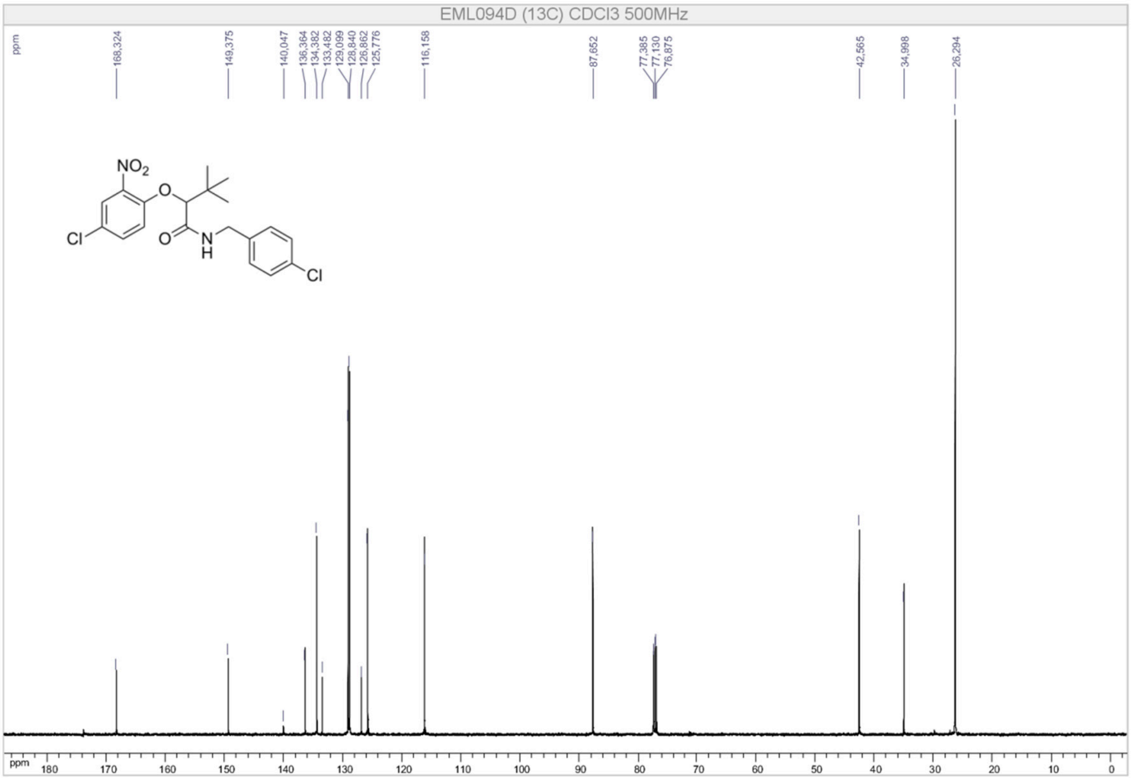

2-(4-Chloro-2-nitrophenoxy)-N-(4-methoxybenzyl)butanamide (**2s**)

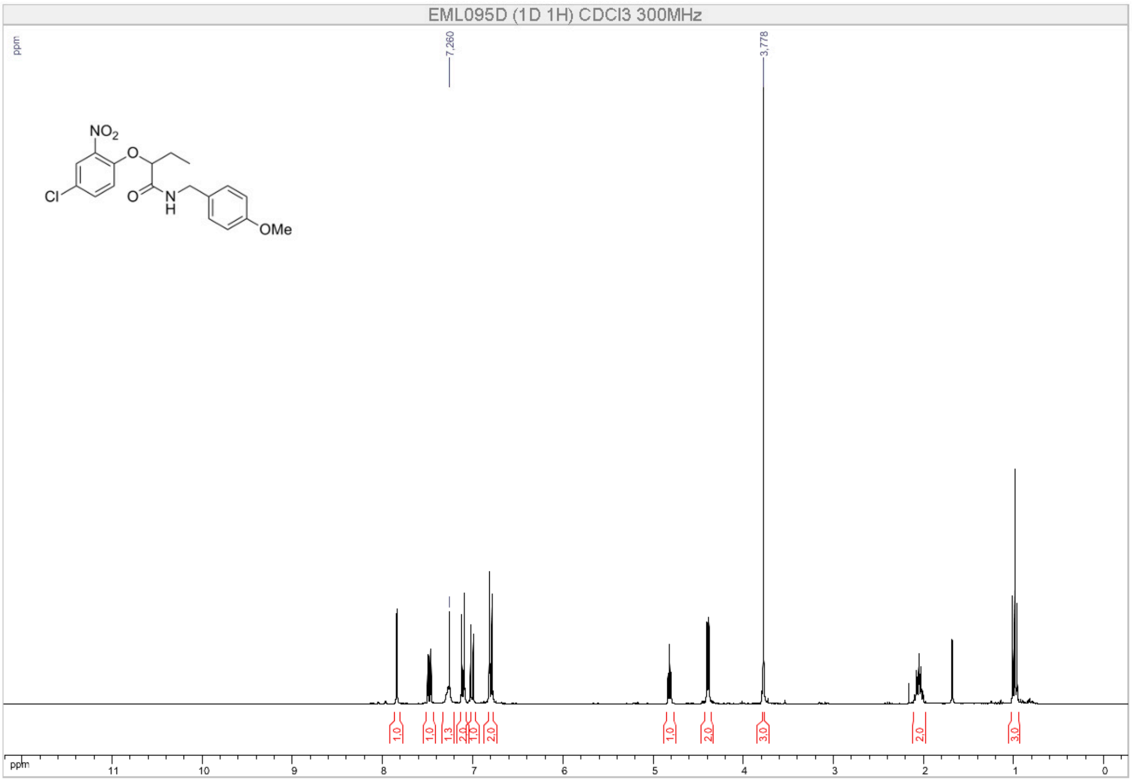

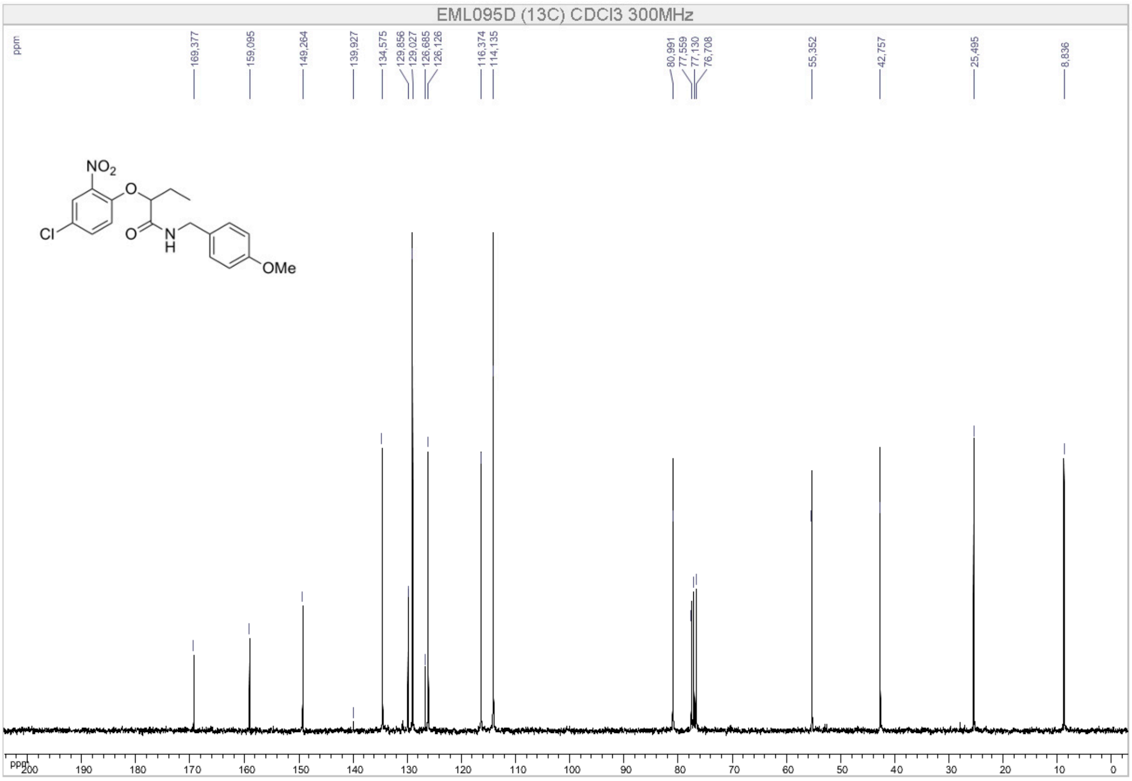

2-(4-Cyano-2-nitrophenoxy)-N-(3,4-dimethoxyphenethyl)-4-methylpentanamide (**2t**)

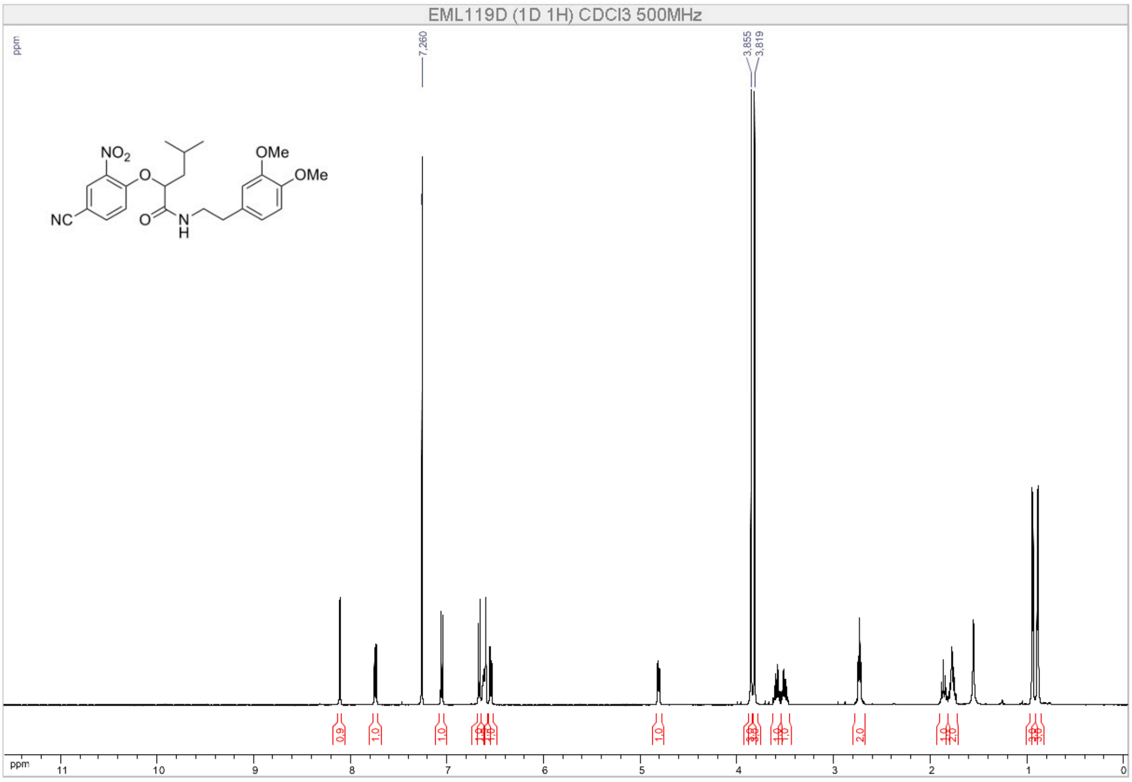

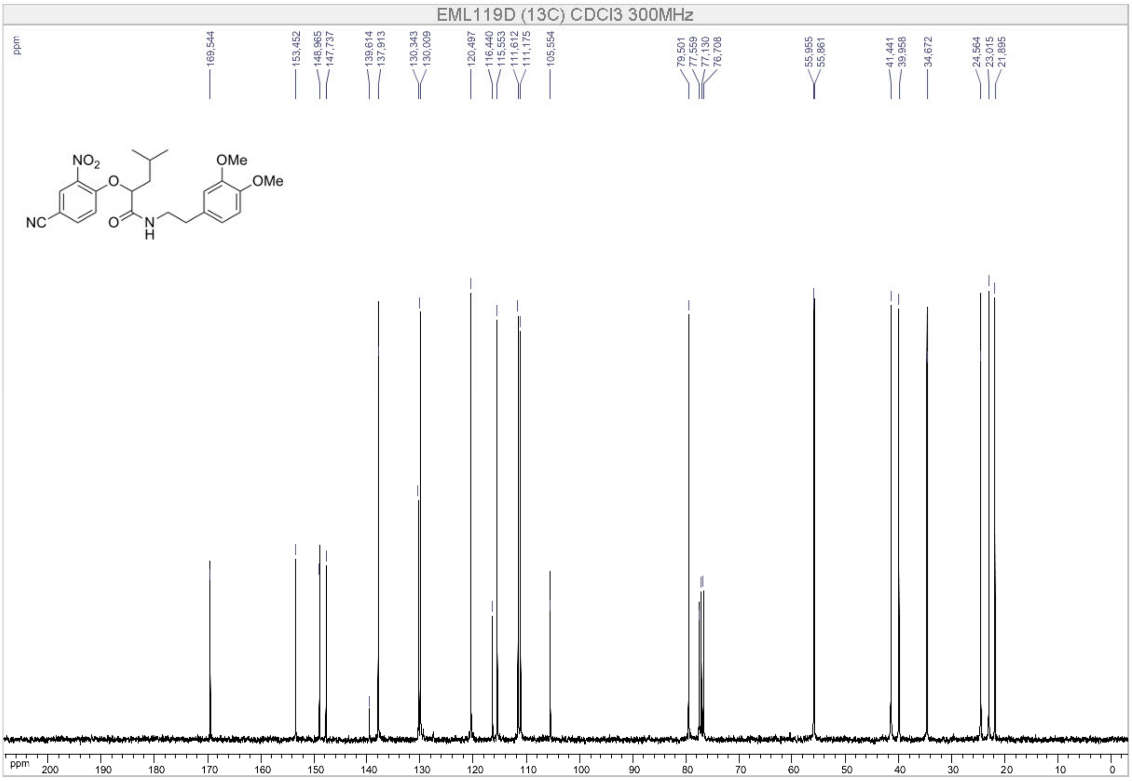

2-(4-Cyano-2-nitrophenoxy)-N-(4-methoxybenzyl)butanamide (**2u**)

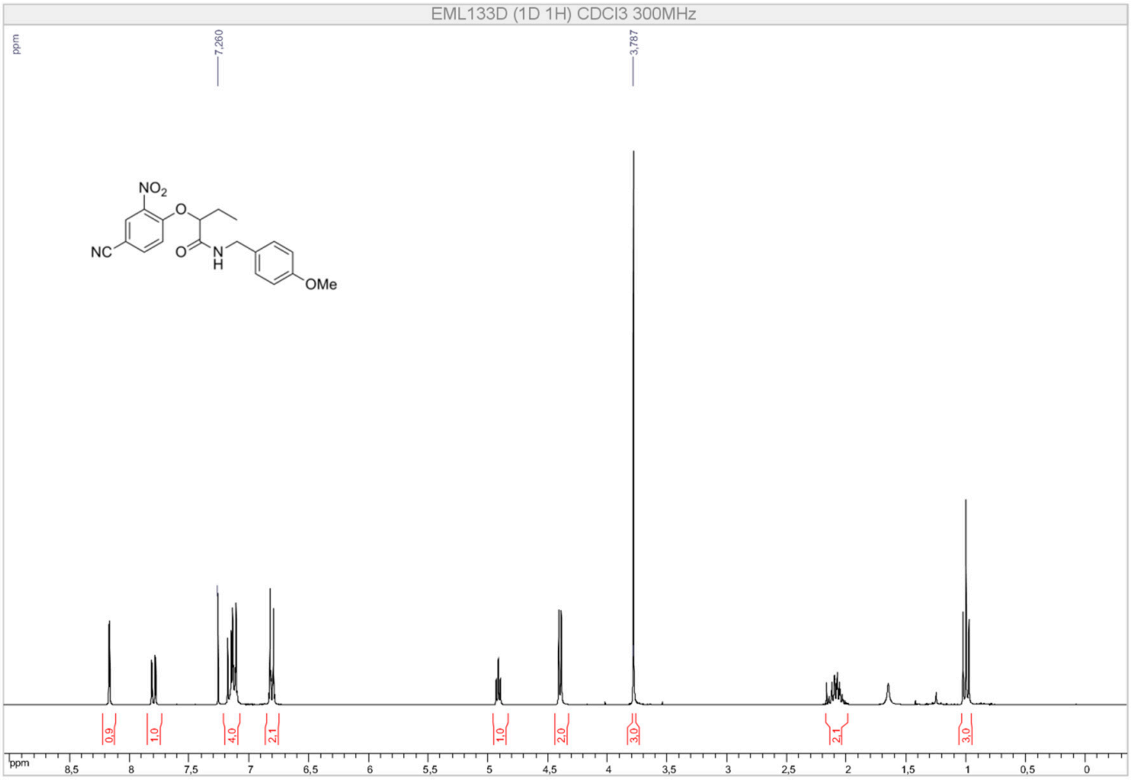

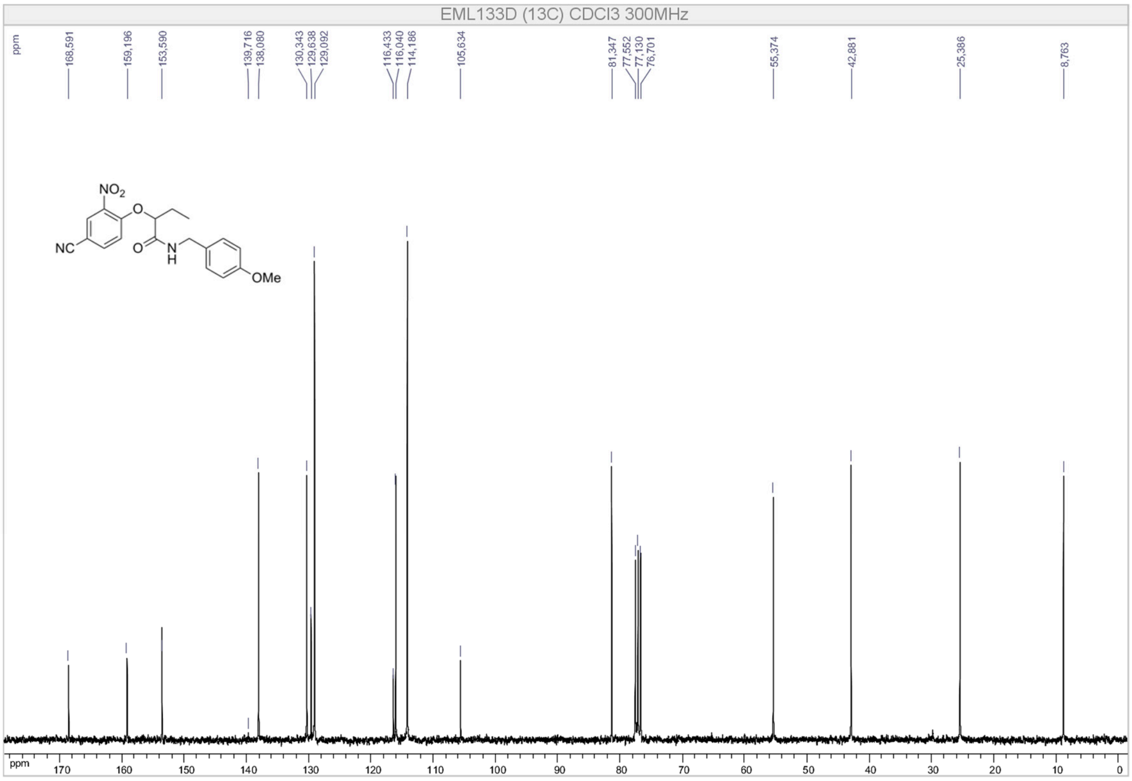

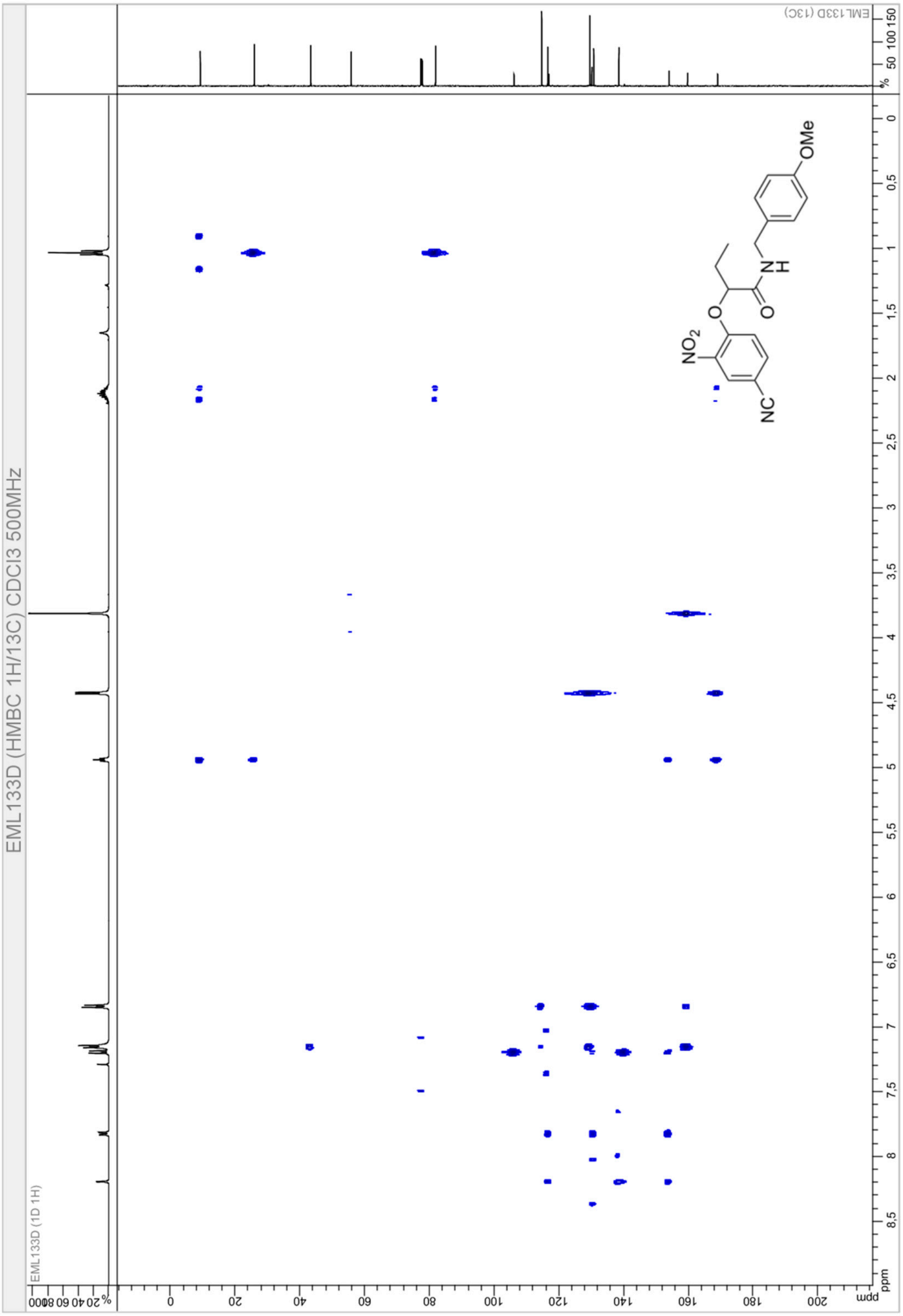
EML133D (HMBC 1H/13C) CDCl3 500MHz
EML133D (13C)
EML133D (1D 1H)
NO2
OMe
NC
O
N
H
ppm

4-Methyl-2-(2-nitro-4-(trifluoromethyl)phenoxy)-N-phenethylpentanamide (**2v**)

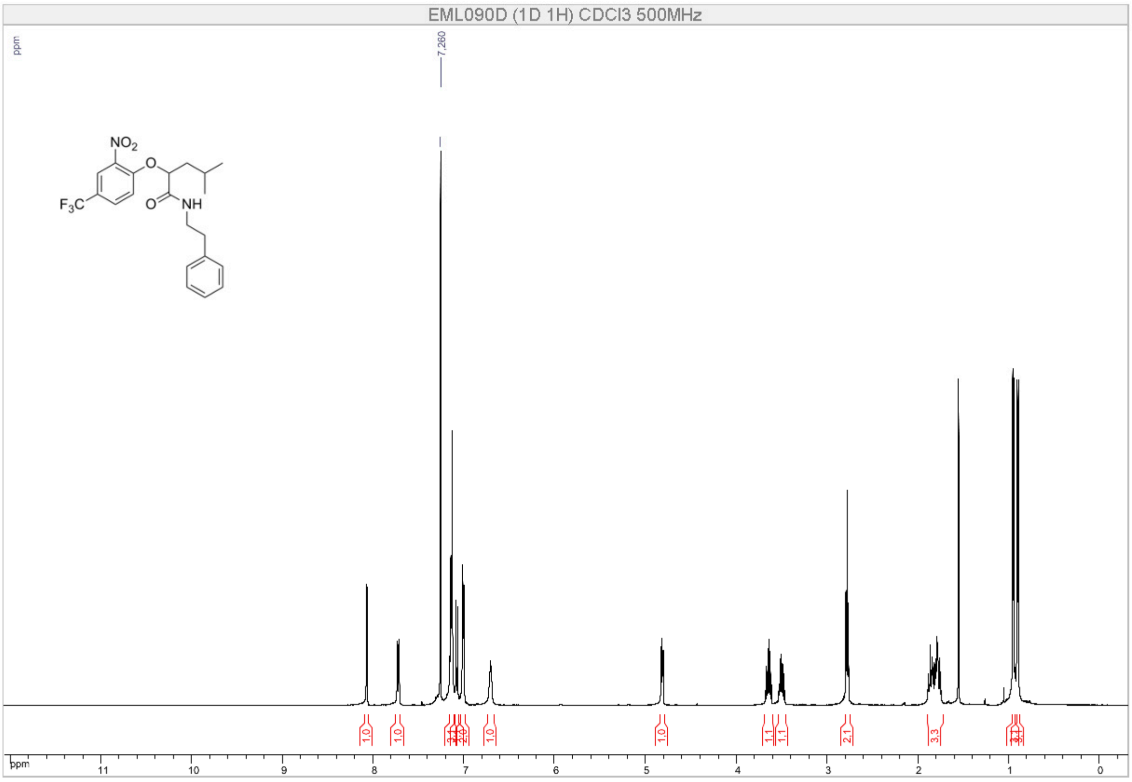

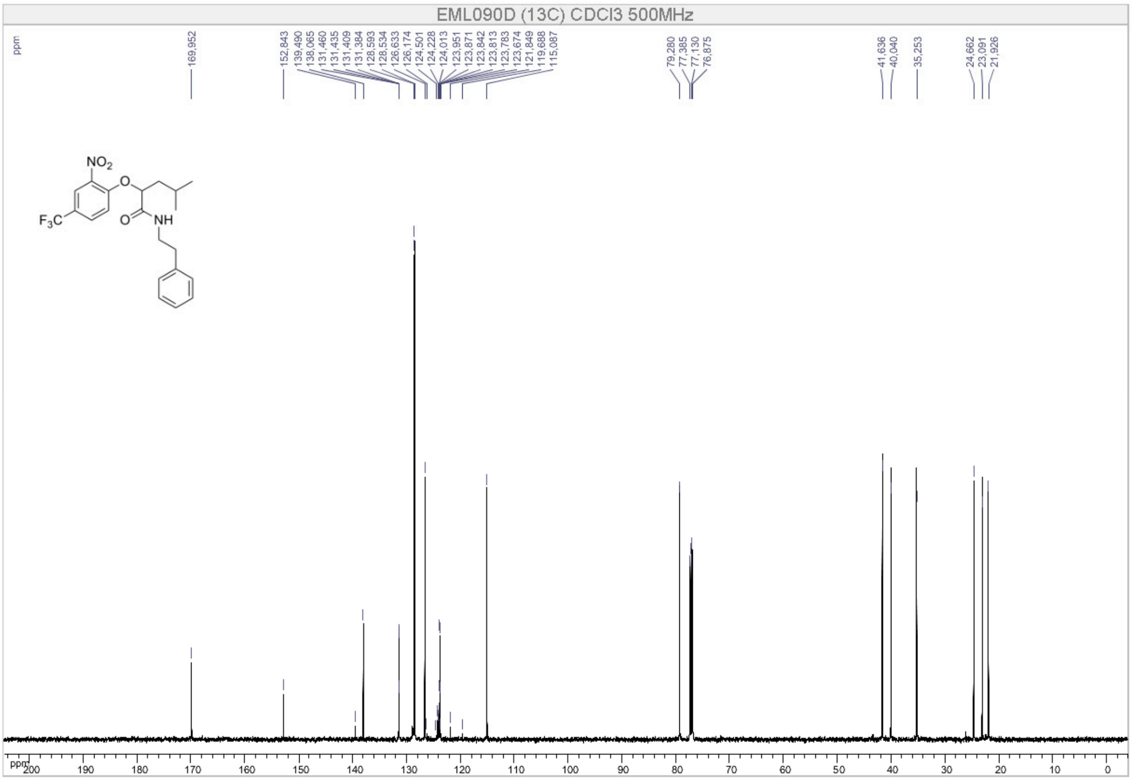

N-(3,4-Dimethoxyphenethyl)-4,8-dimethyl-2-(2-nitro-4-(trifluoromethyl)phenoxy)non-7-enamide (**2w**)

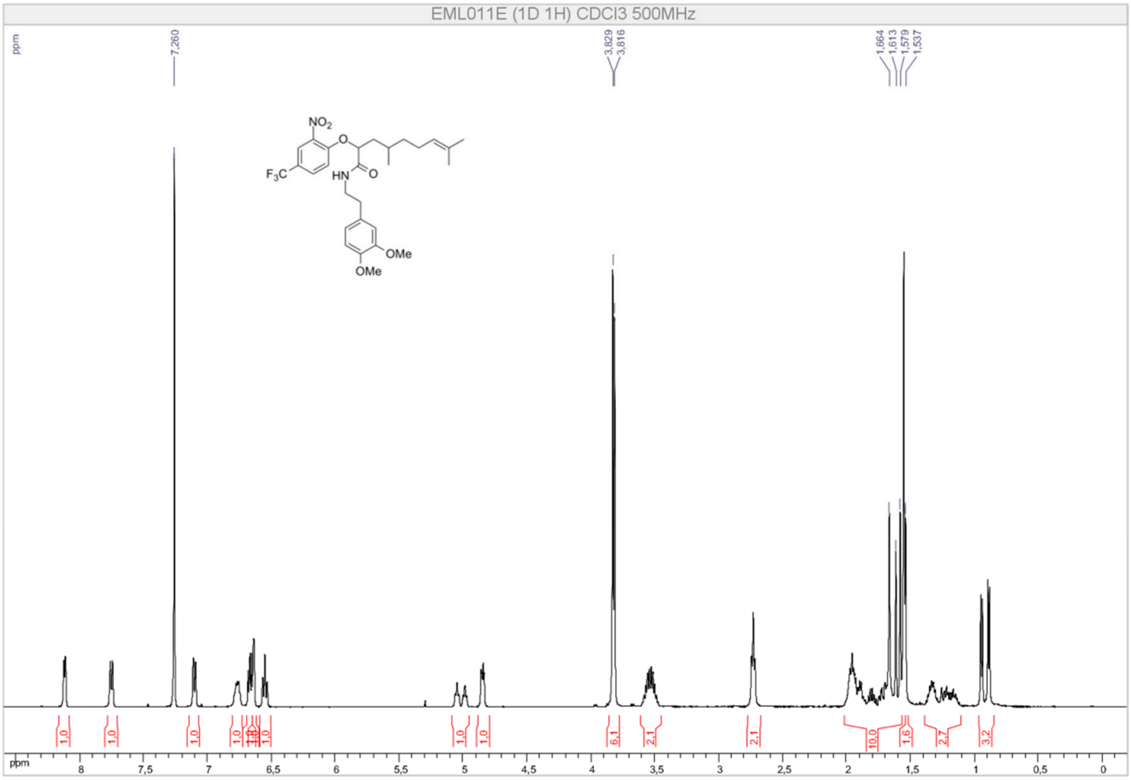

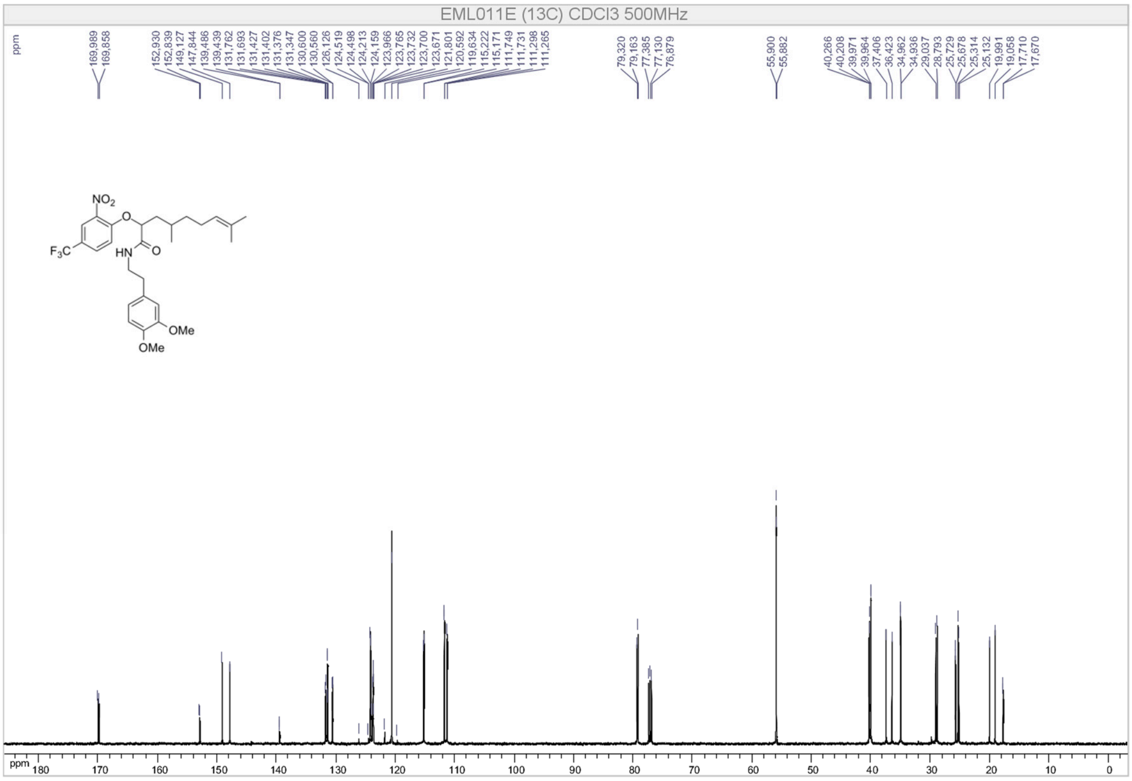

N-Cyclohexyl-2-((2-nitropyridin-3-yl)oxy)butanamide (**2xa**)

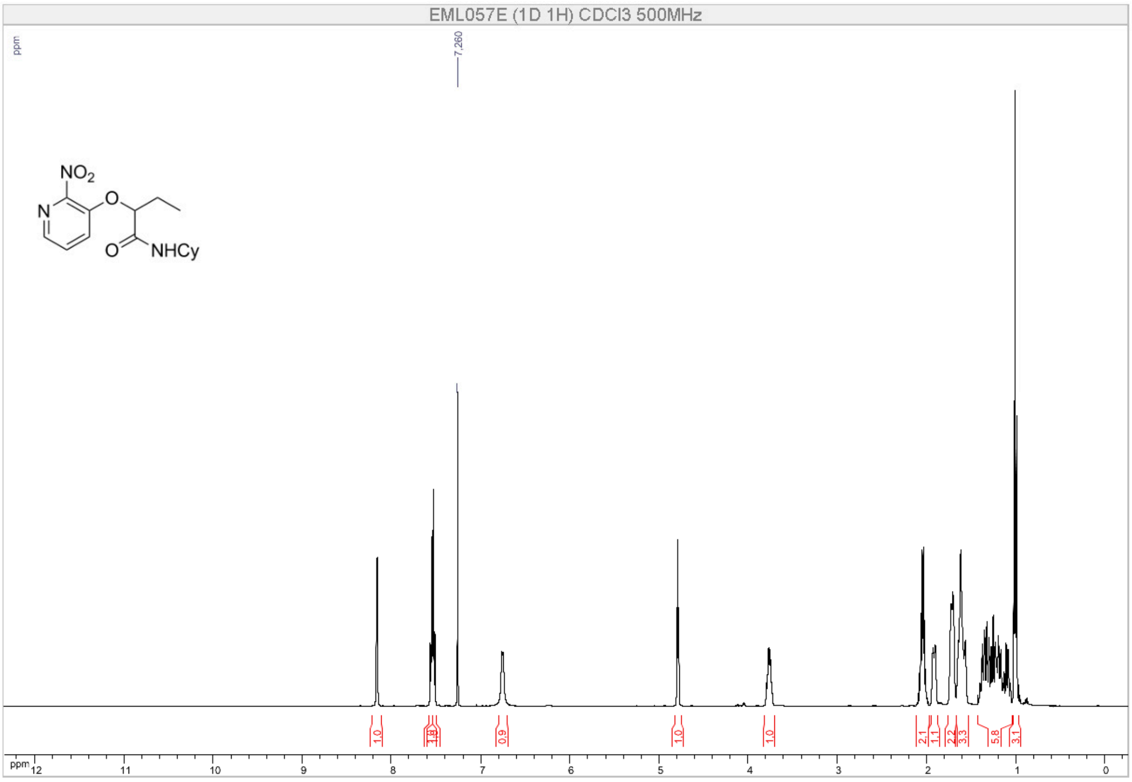

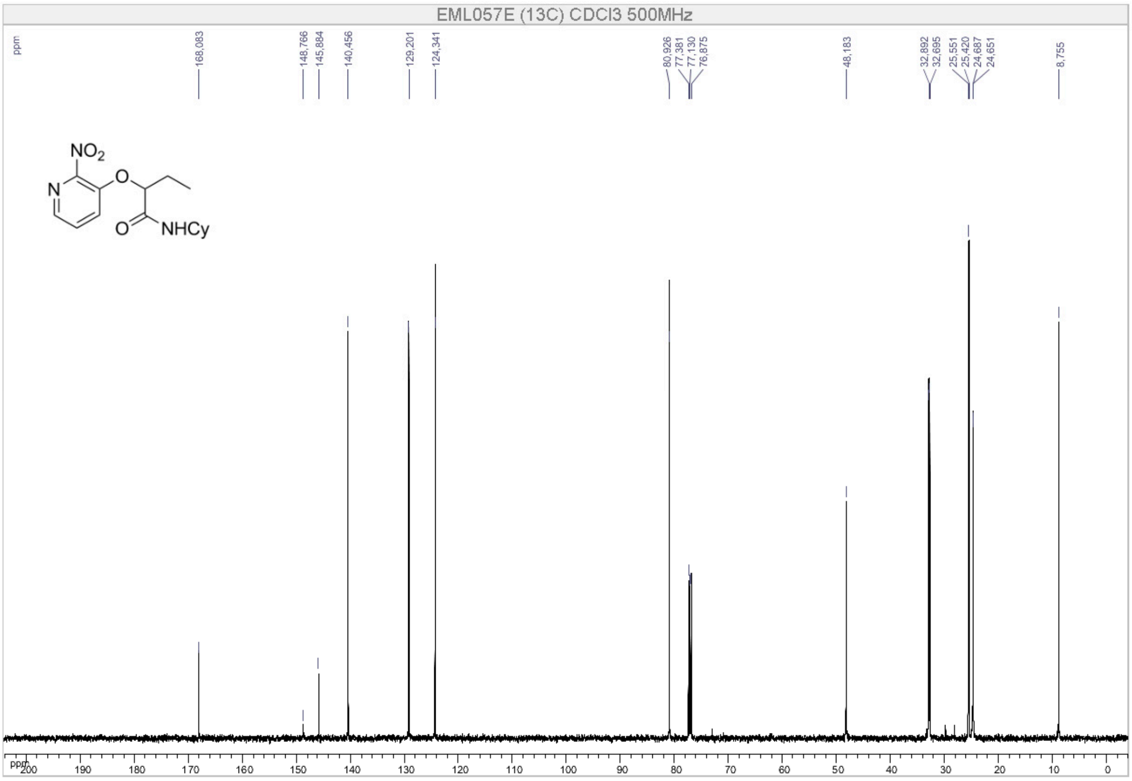

N-Cyclohexyl-3-methyl-2-((2-nitropyridin-3-yl)oxy)butanamide (**2xb**)

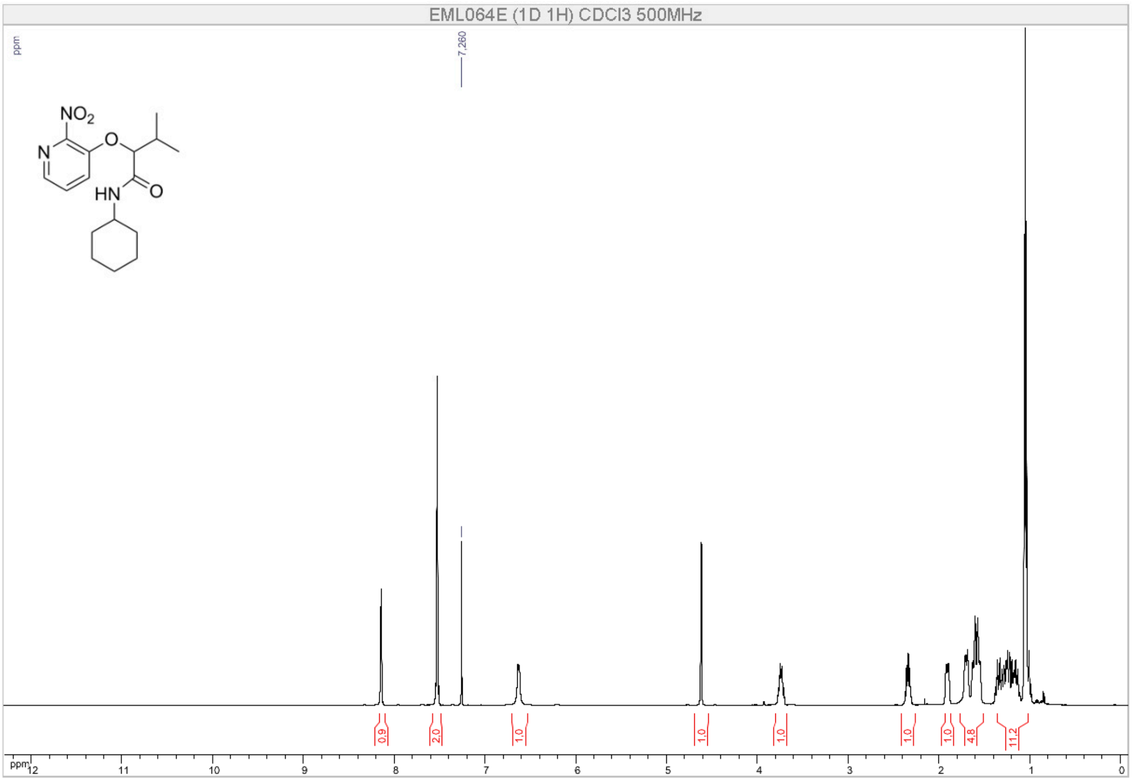

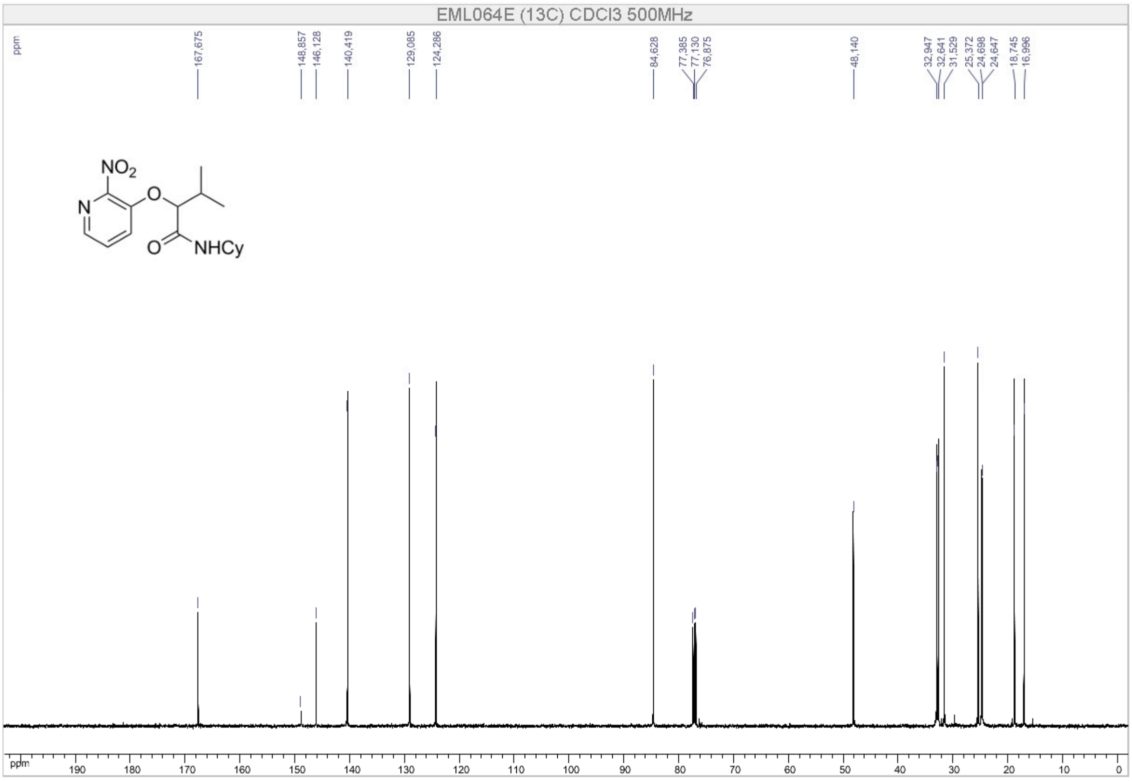

N-Cyclohexyl-2-((3-nitropyridin-2-yl)oxy)butanamide (**2ya**)

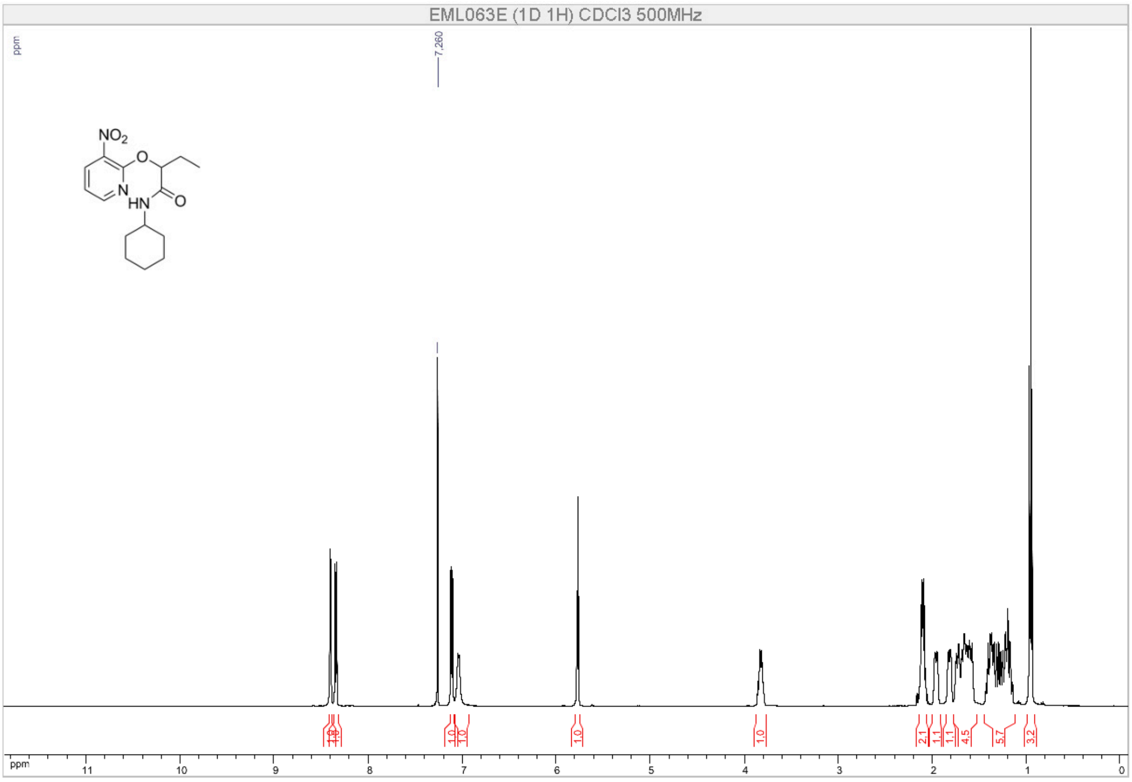

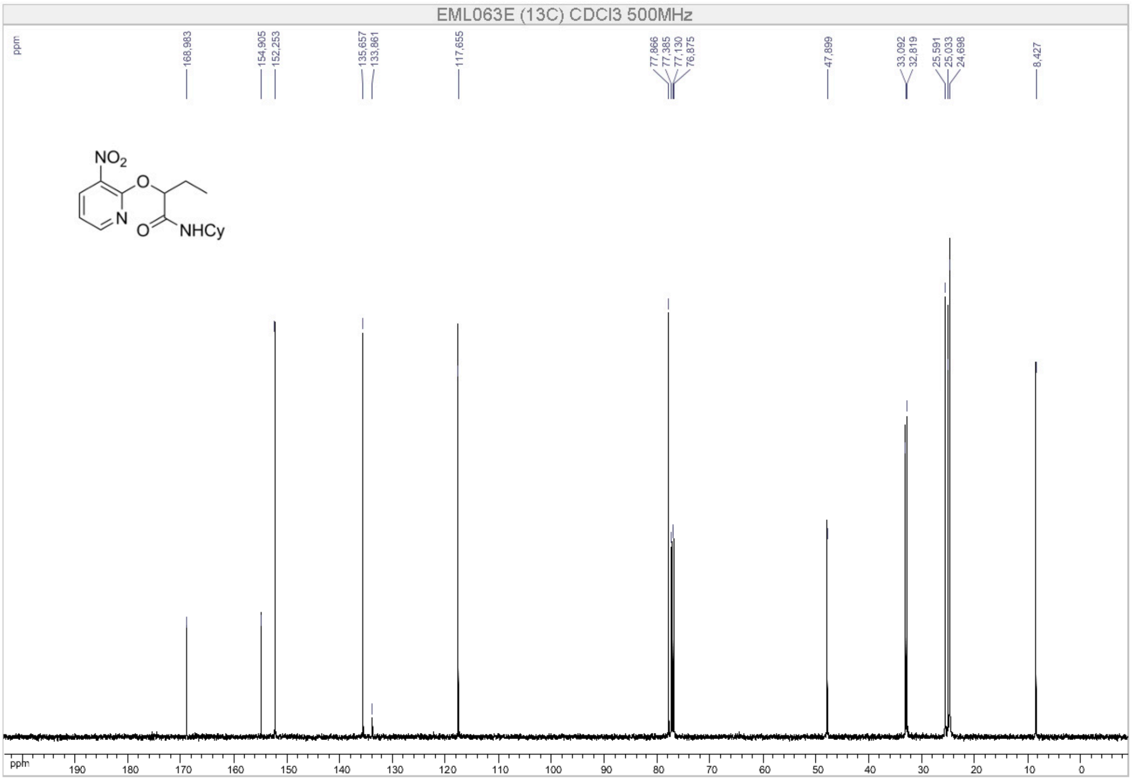

N-Cyclohexyl-3-methyl-2-((3-nitropyridin-2-yl)oxy)butanamide (**2yb**)

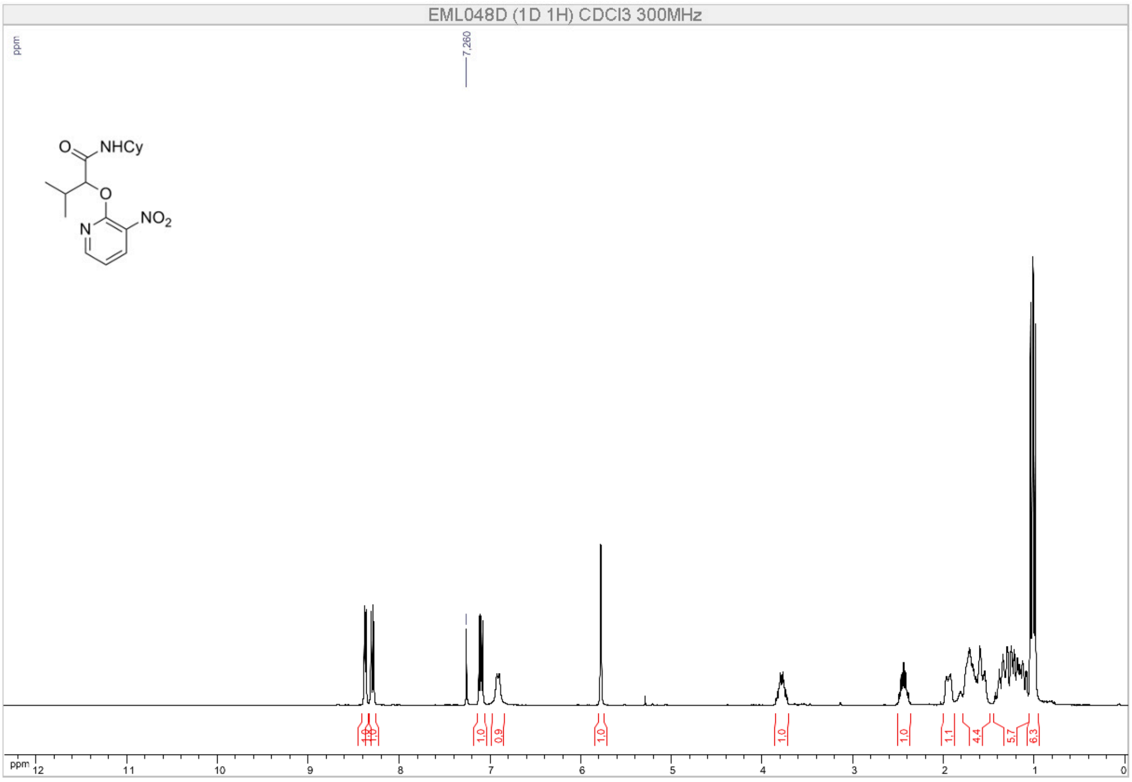

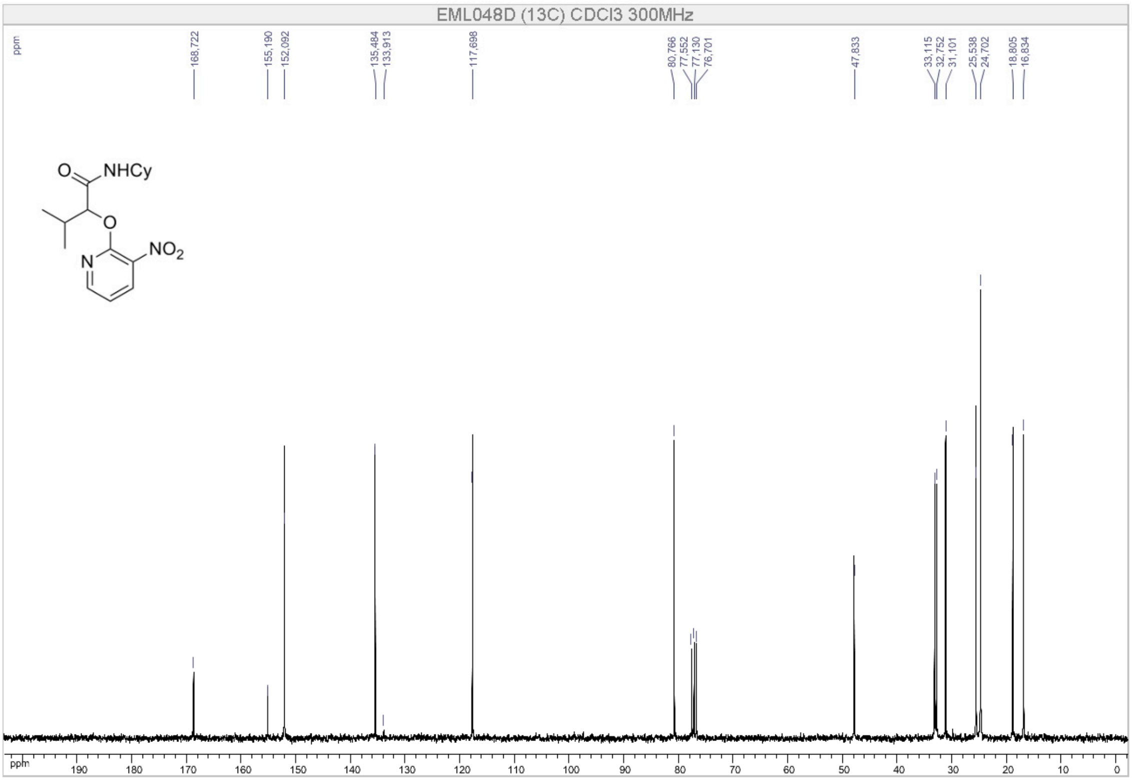

N-Cyclohexyl-3-methyl-2-(4-nitrophenoxy)butanamide (**2z**)

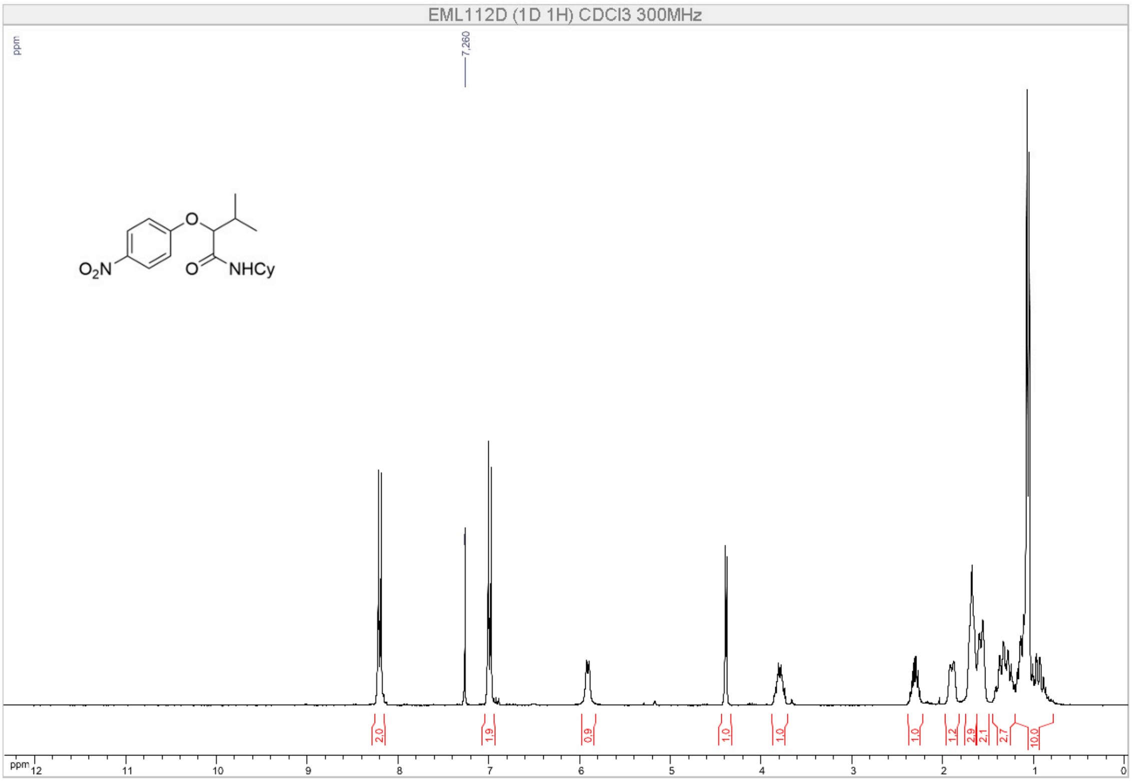

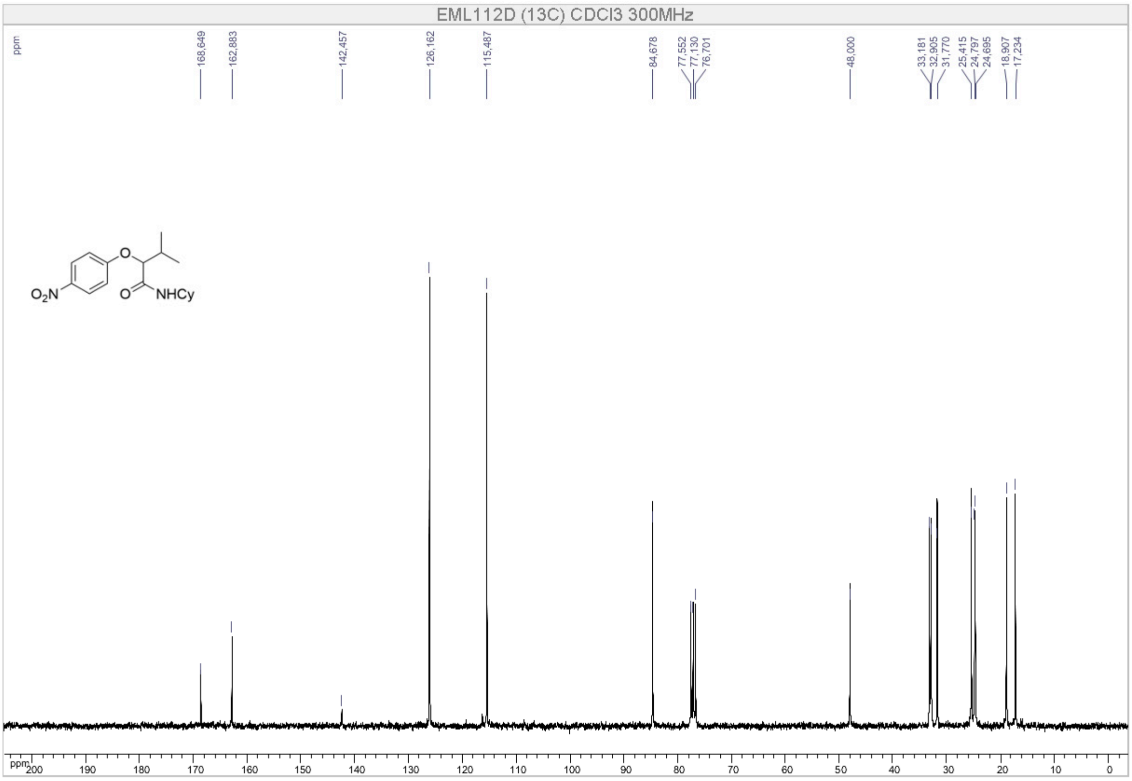

N-Cyclohexyl-2-(1-(2-nitrophenoxy)propoxy)butanamide (**3a**)

Diastereomer 1 (contaminated by Passerini-Smiles adduct)

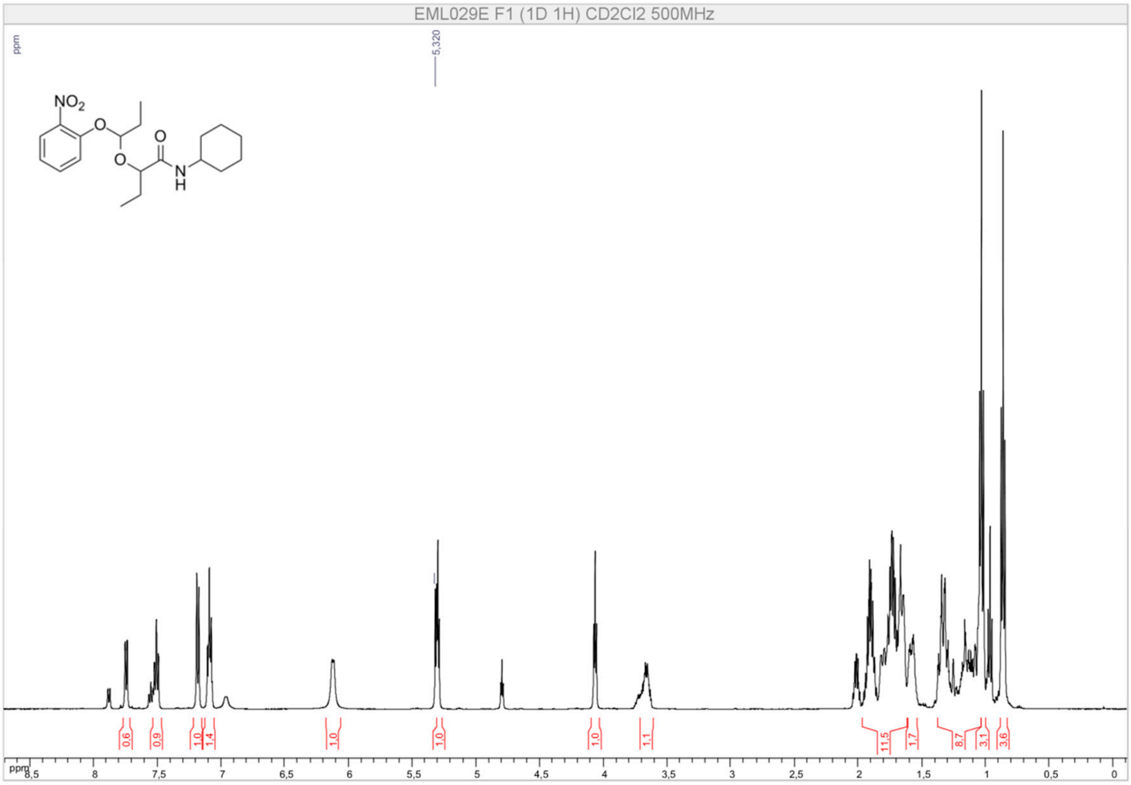

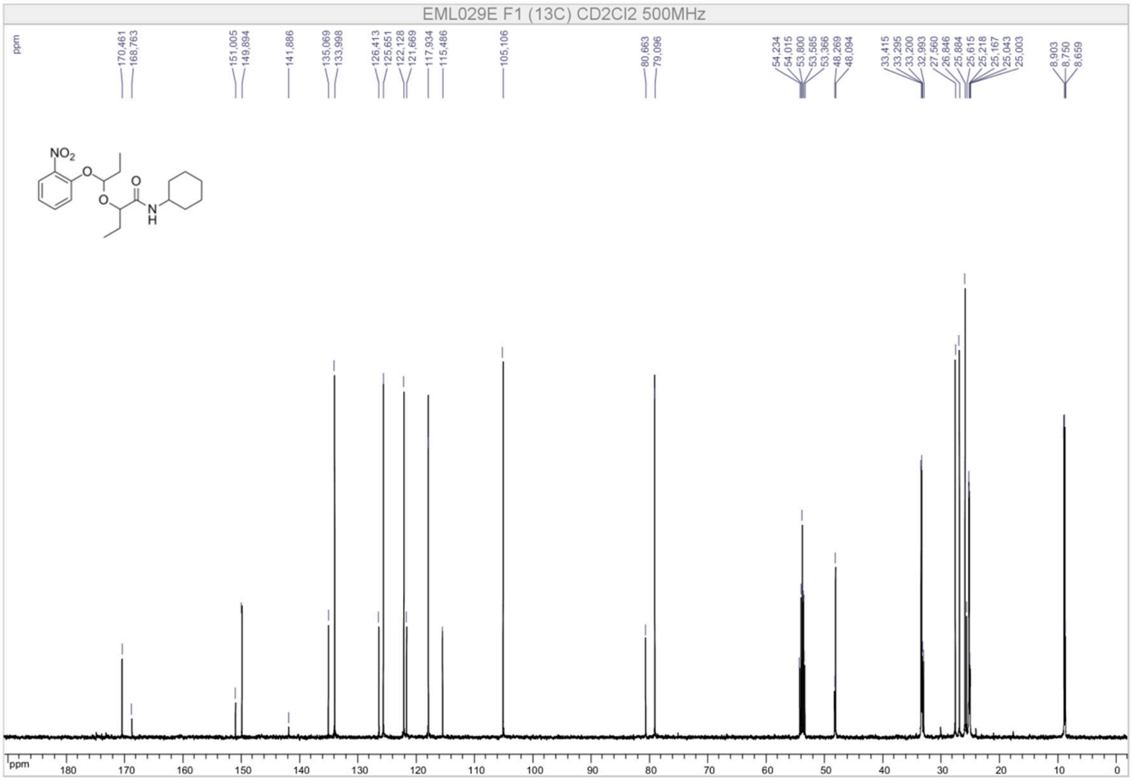

Diastereomer 2

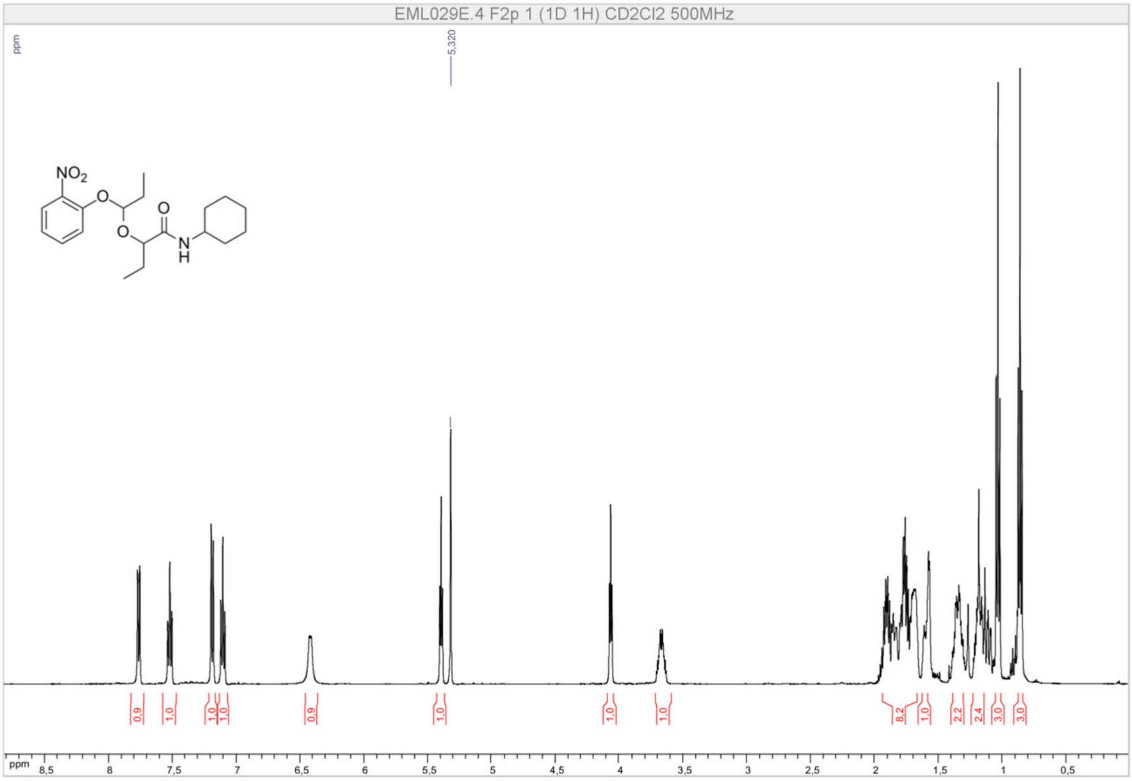

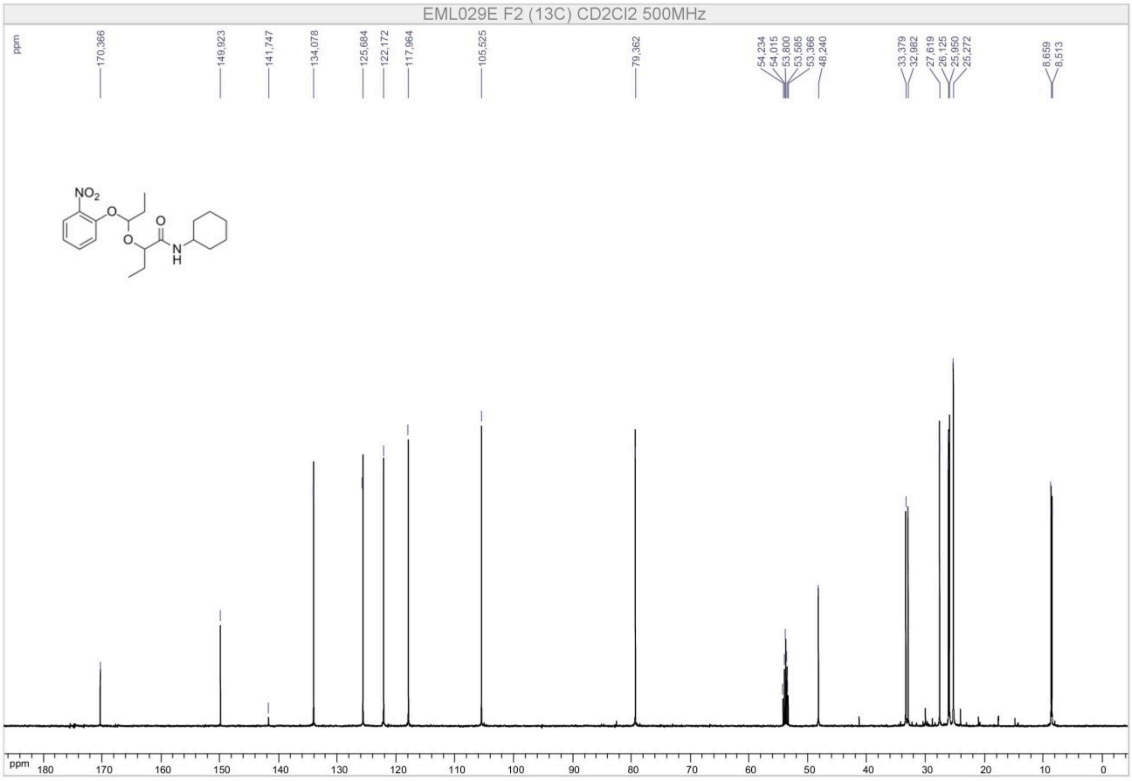

N-Cyclohexyl-2-(2-fluoro-4-nitrophenoxy)-3-methylbutanamide (**4a**)

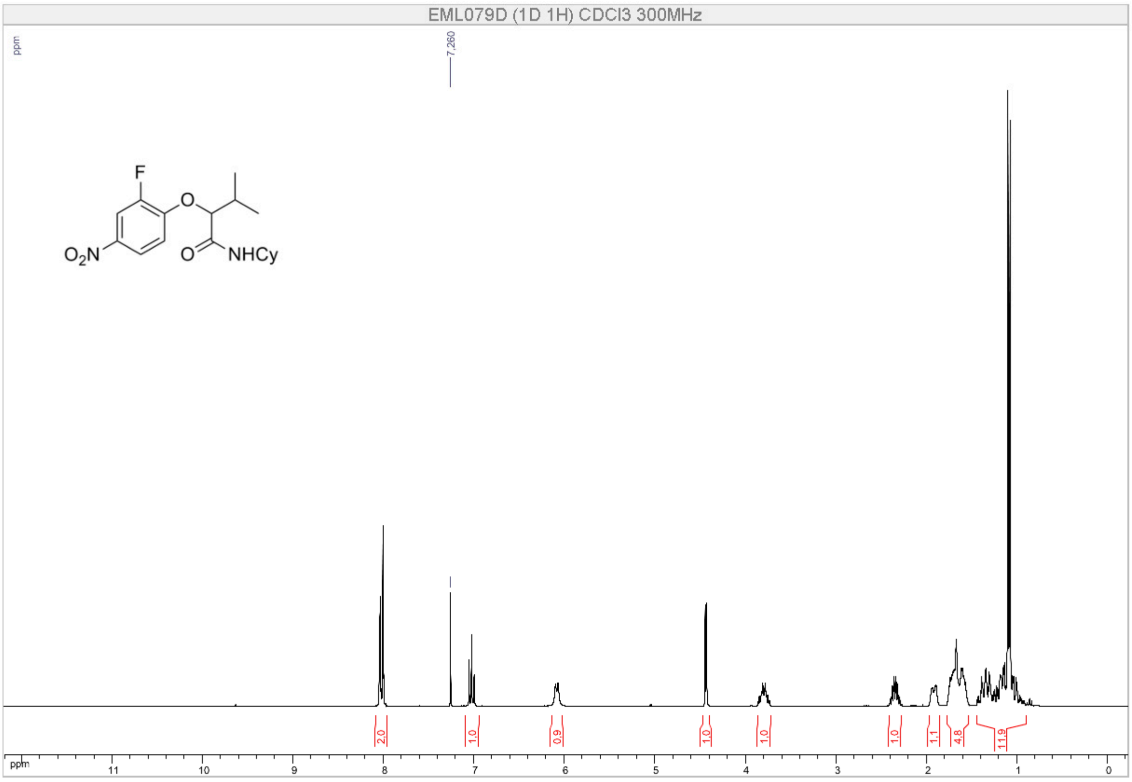

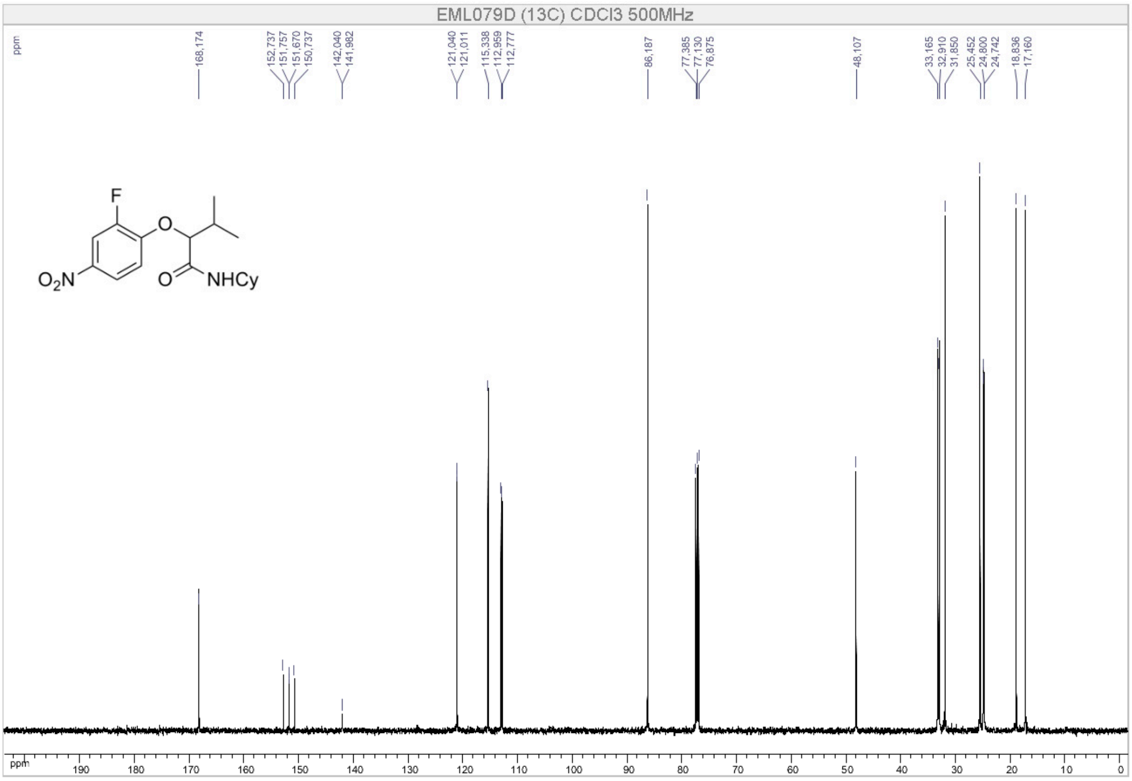

2-(2-Chloro-4-nitrophenoxy)-N-cyclohexyl-3-methylbutanamide (**4b**)

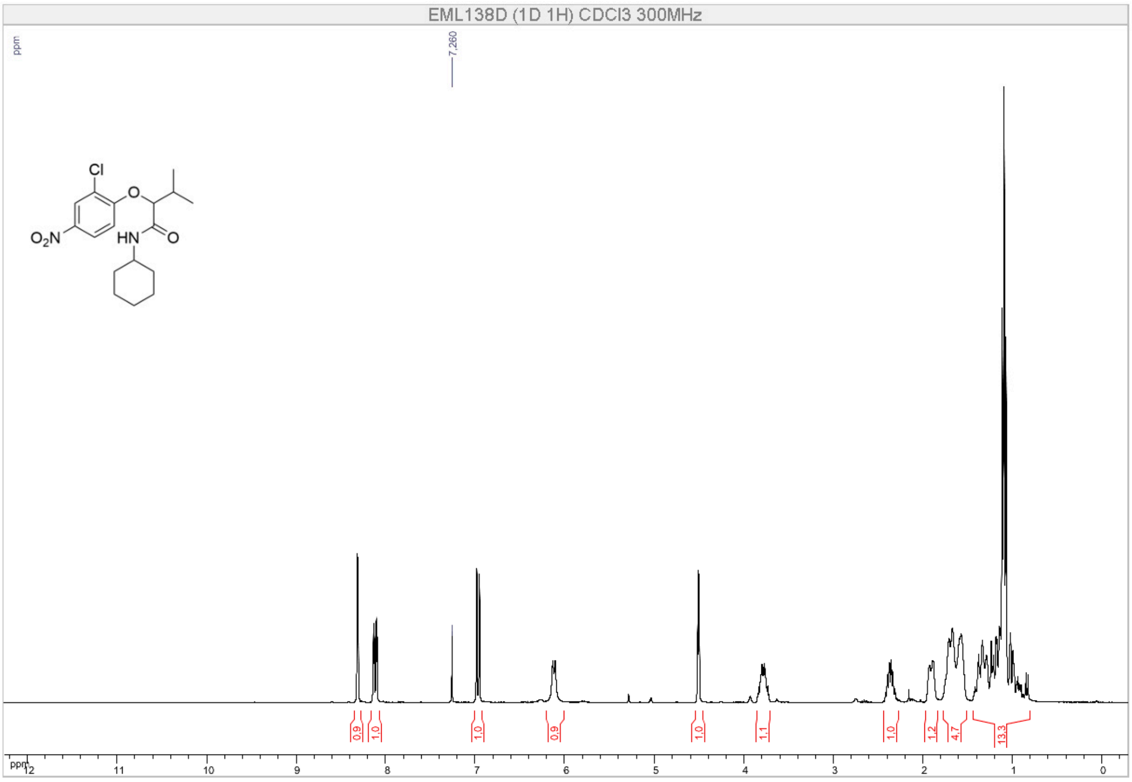

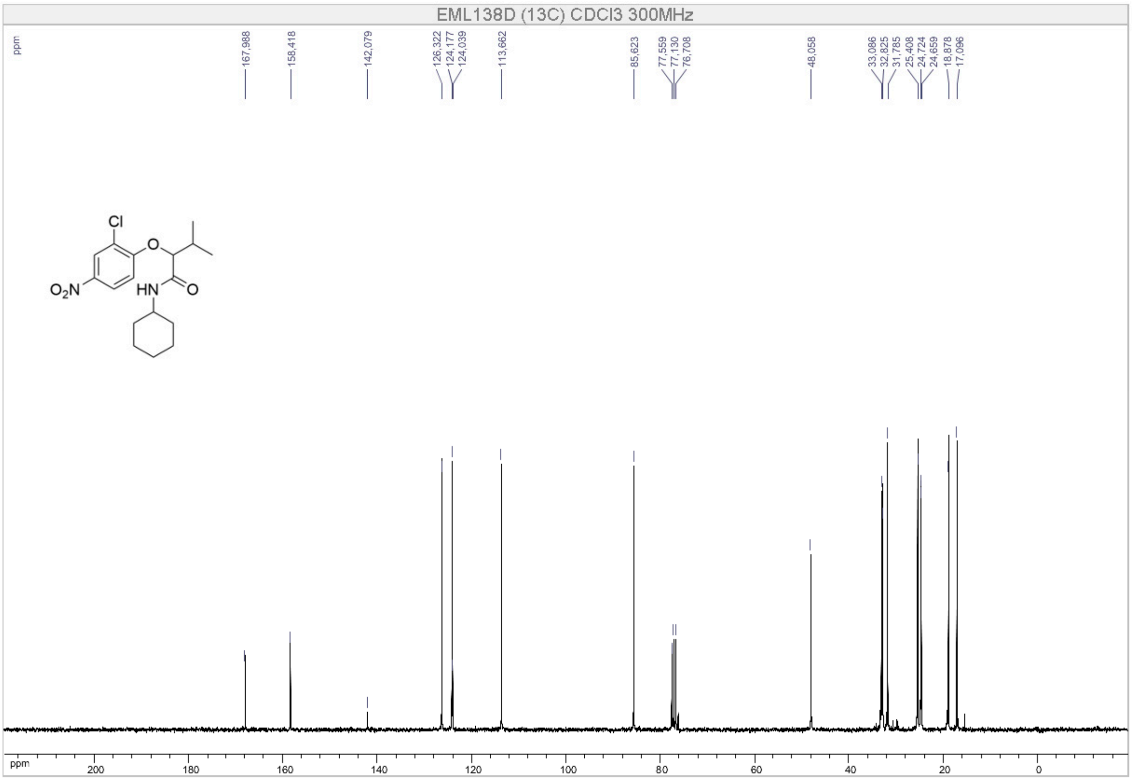

N-Cyclohexyl-2-(2,4-dinitrophenoxy)-3-methylbutanamide (**4c**)

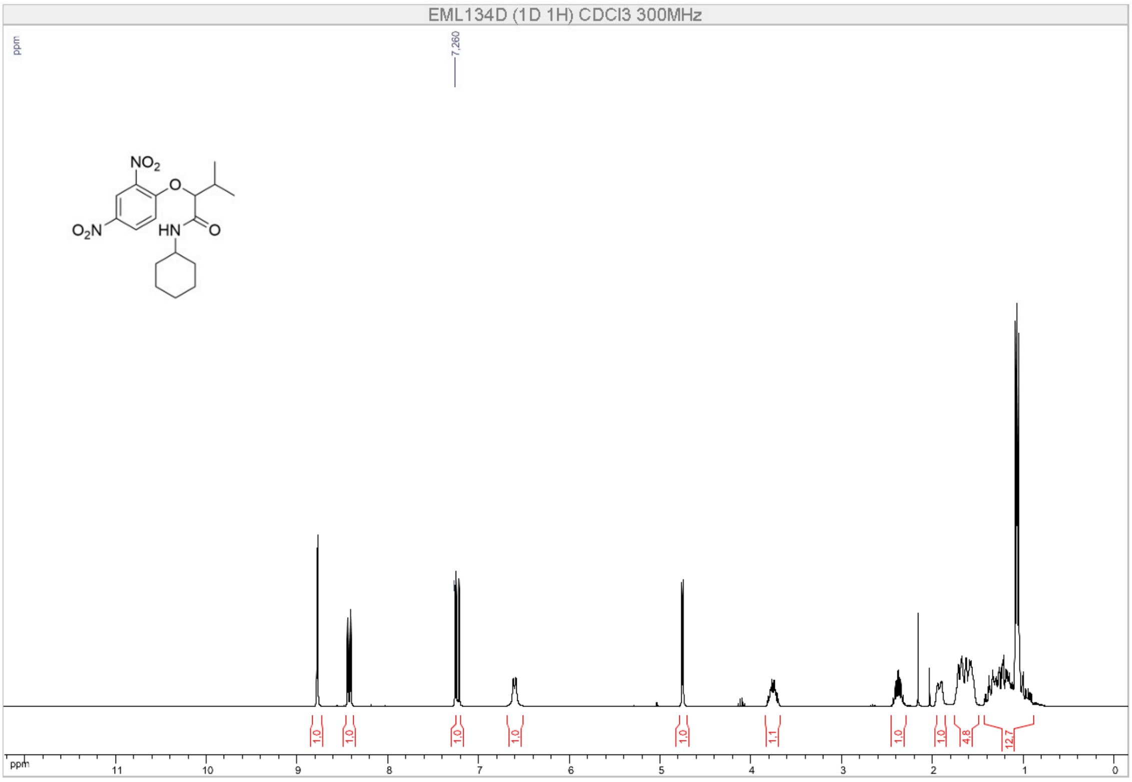

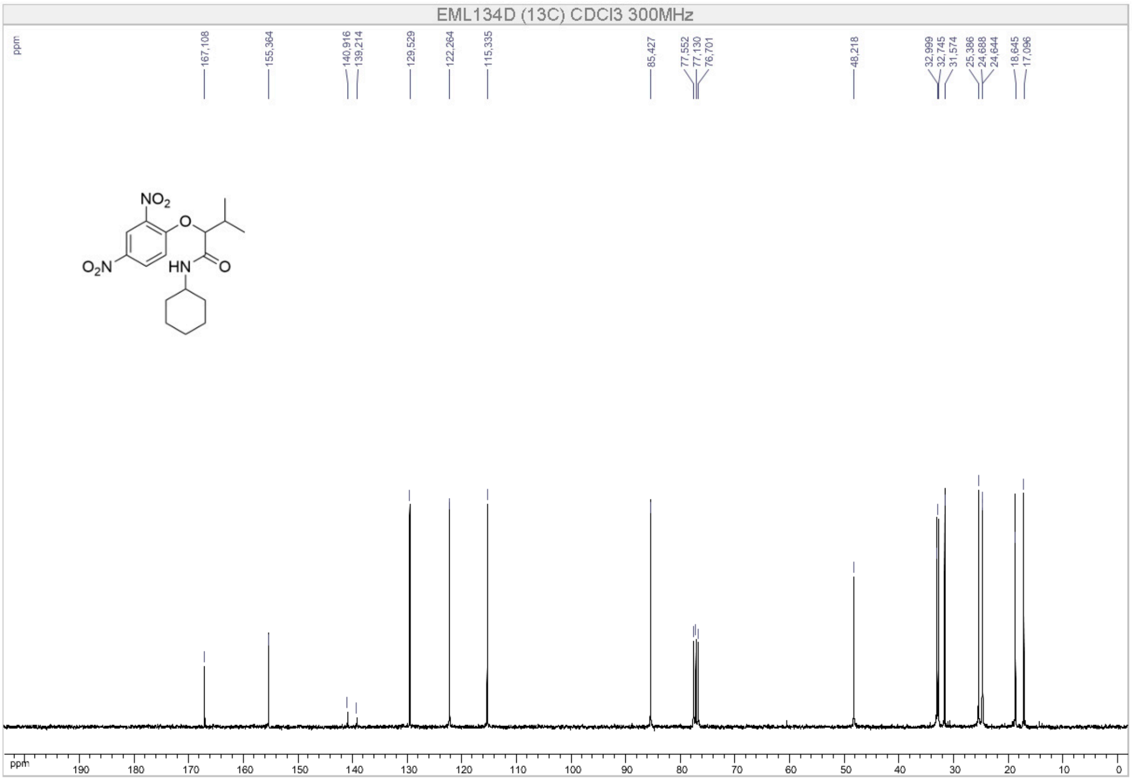

2-(2-Bromo-4-nitrophenoxy)-N-cyclohexyl-3,3-dimethylbutanamide (**4d**)

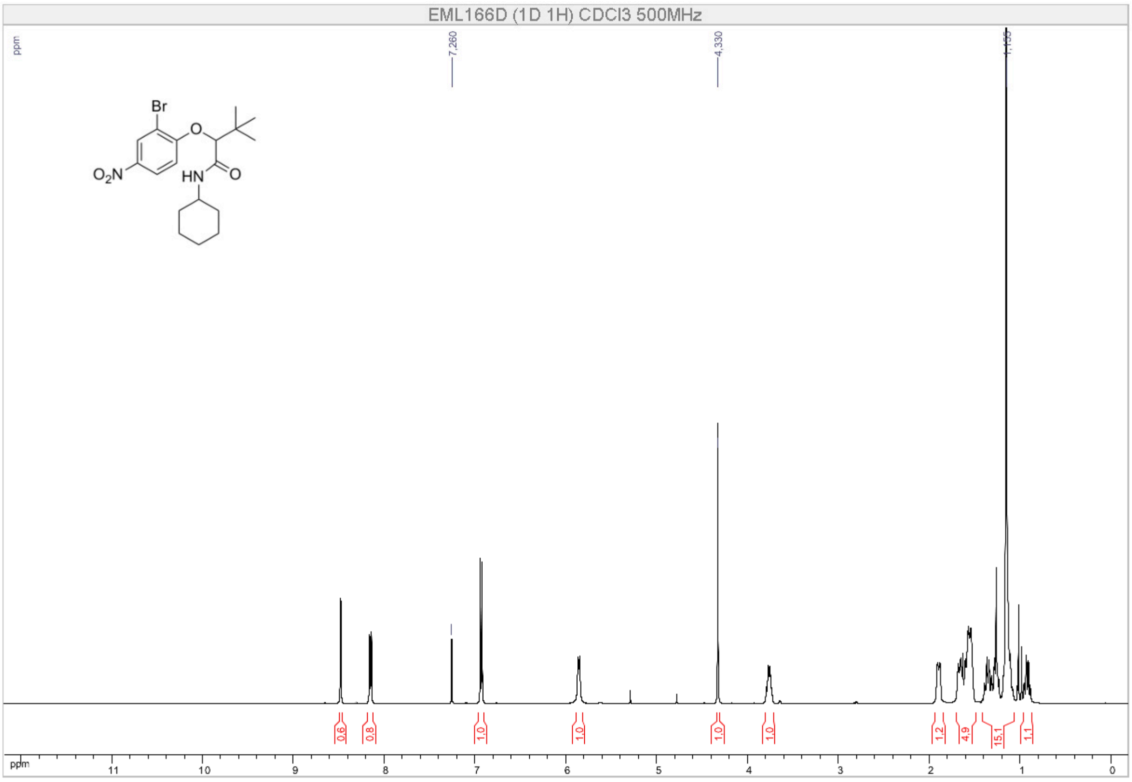

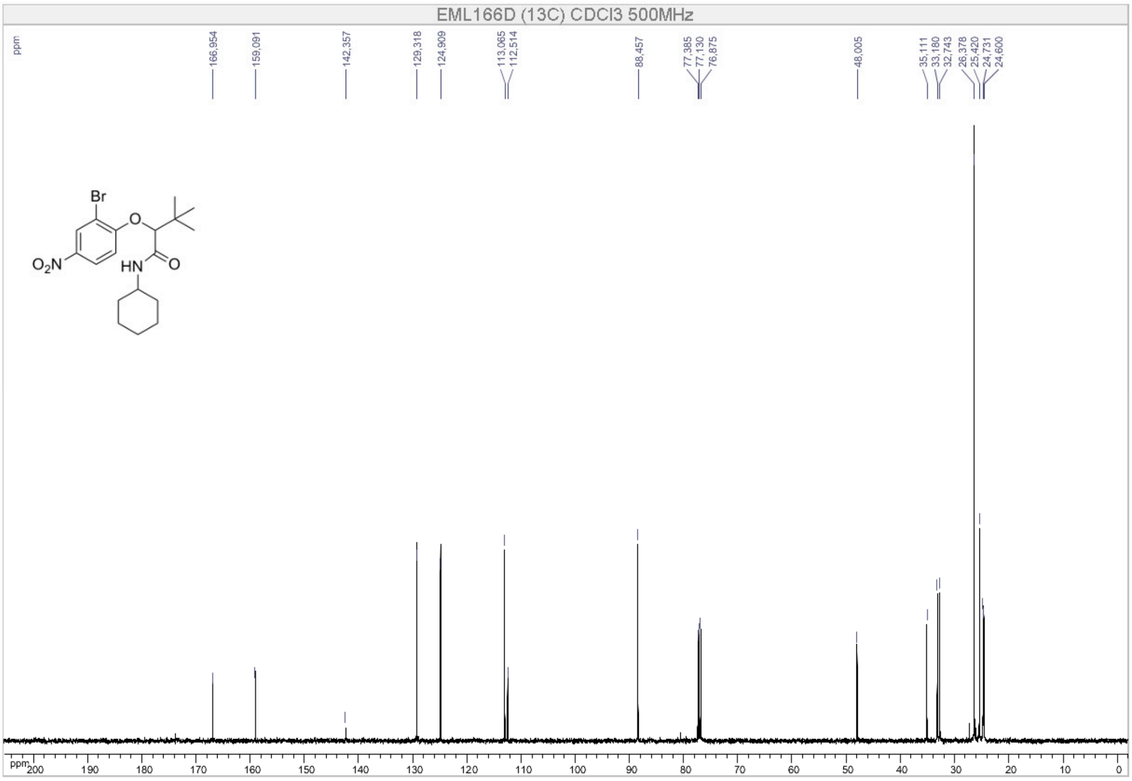

N-(4-Chlorobenzyl)-2-(2-fluoro-4-nitrophenoxy)-4,8-dimethylnon-7-enamide (**4e**)

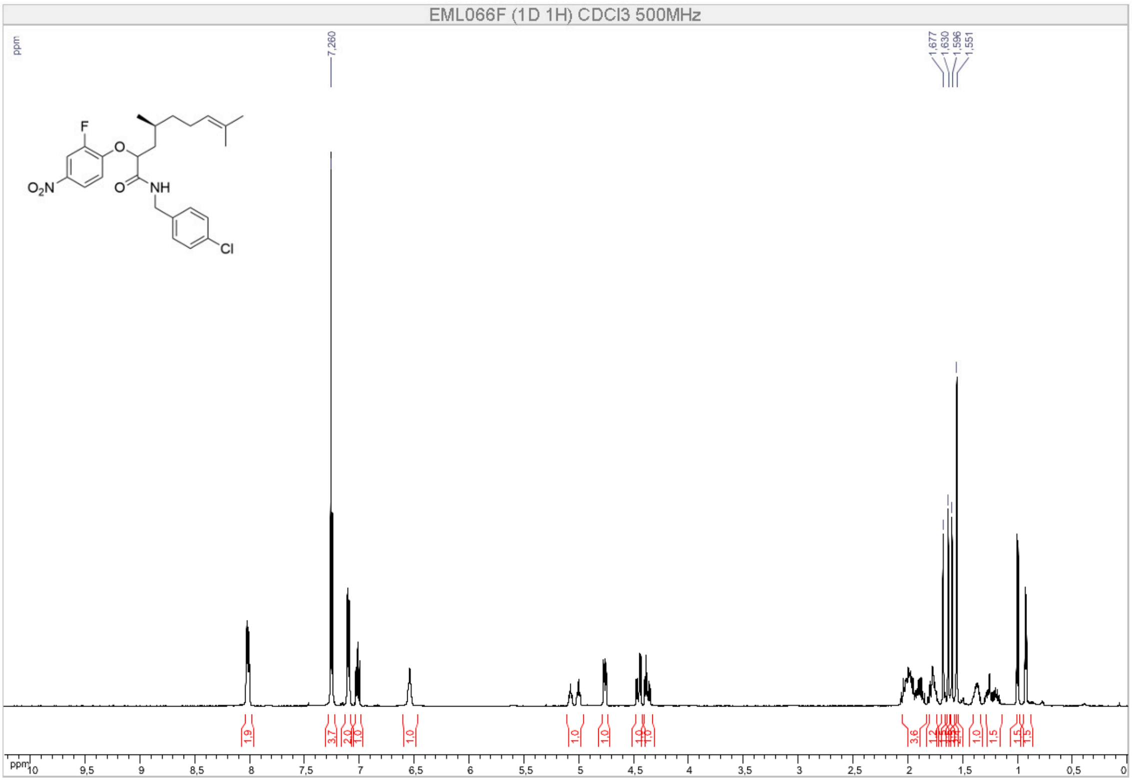

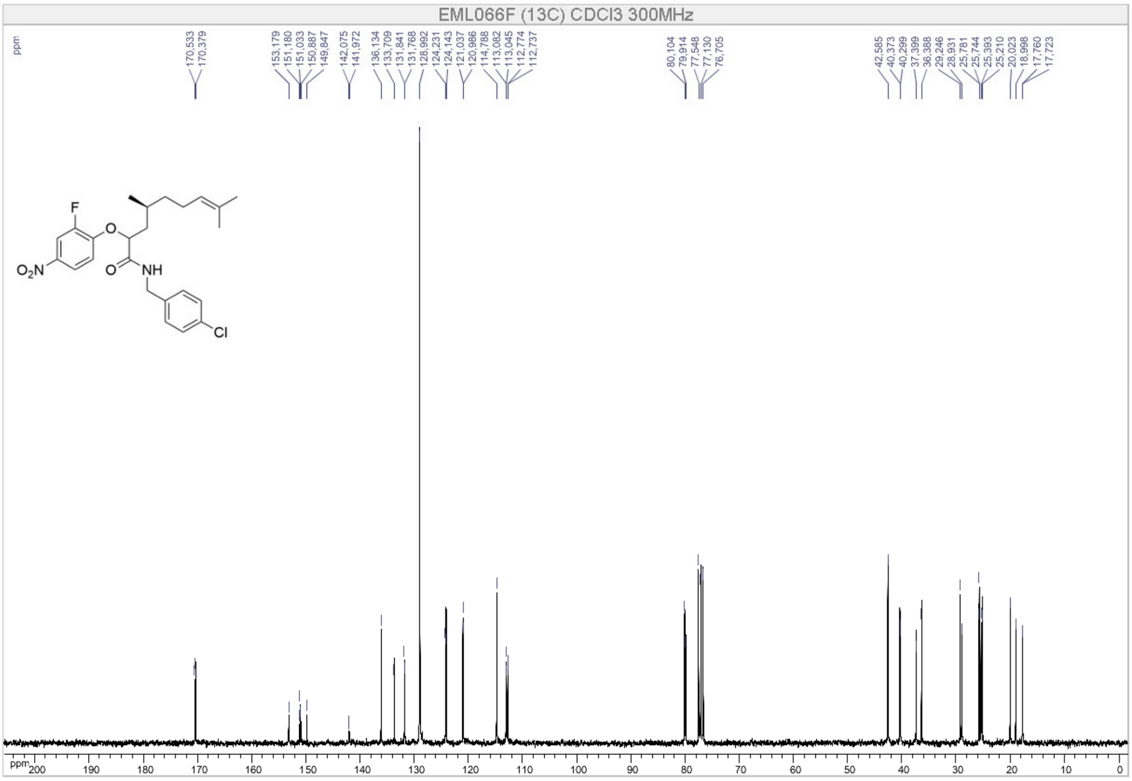

2-(2-Fluoro-4-nitrophenoxy)-N-phenethyl-4-phenylbutanamide (**4f**)

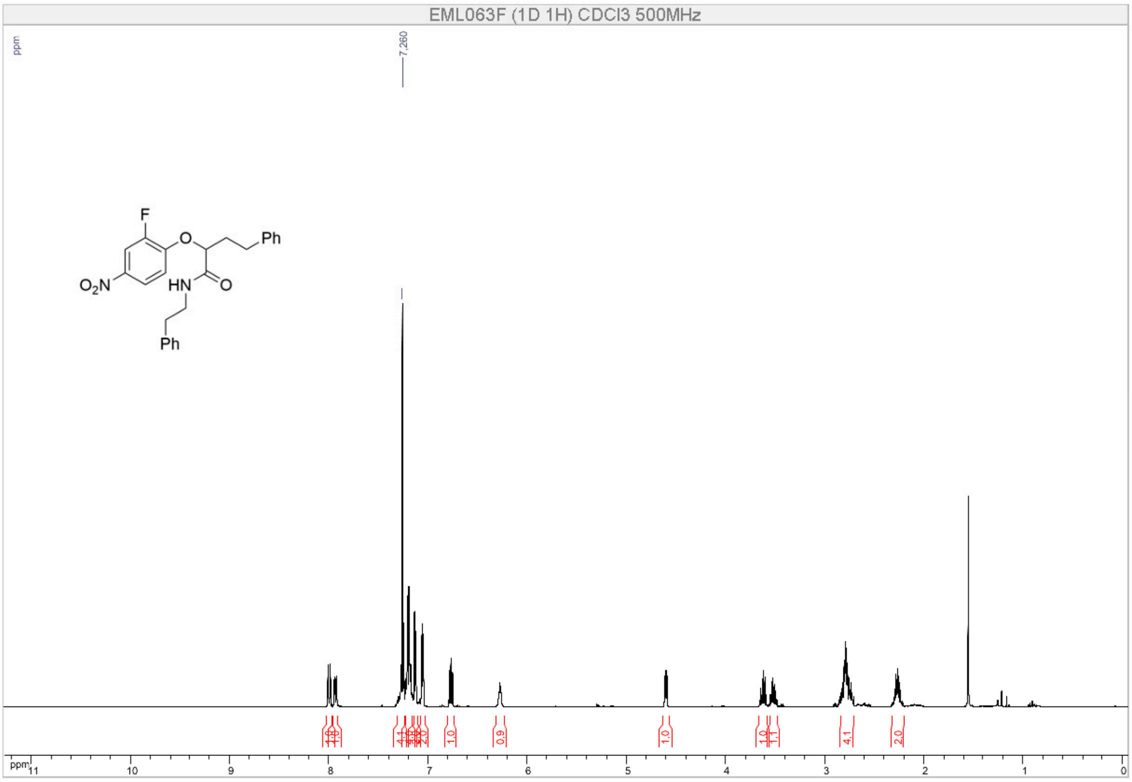

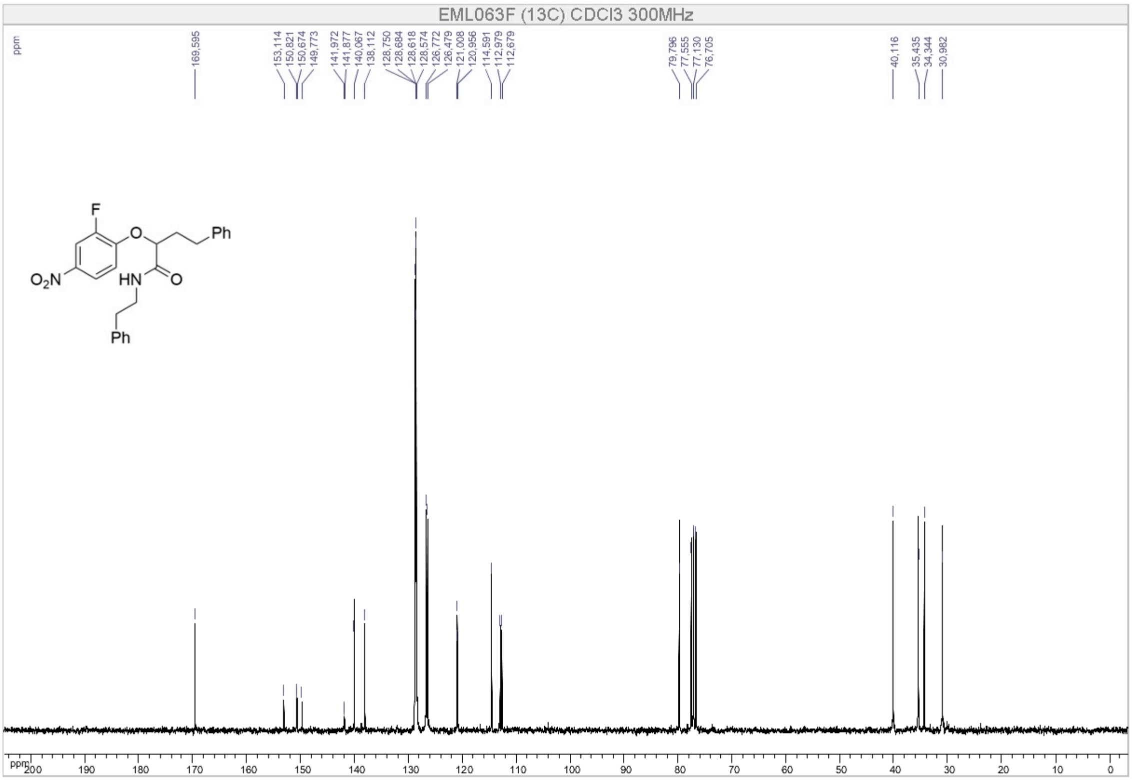

2-(2-Fluoro-4-nitrophenoxy)-N-(4-methoxybenzyl)-3-methylbutanamide (**4g**)

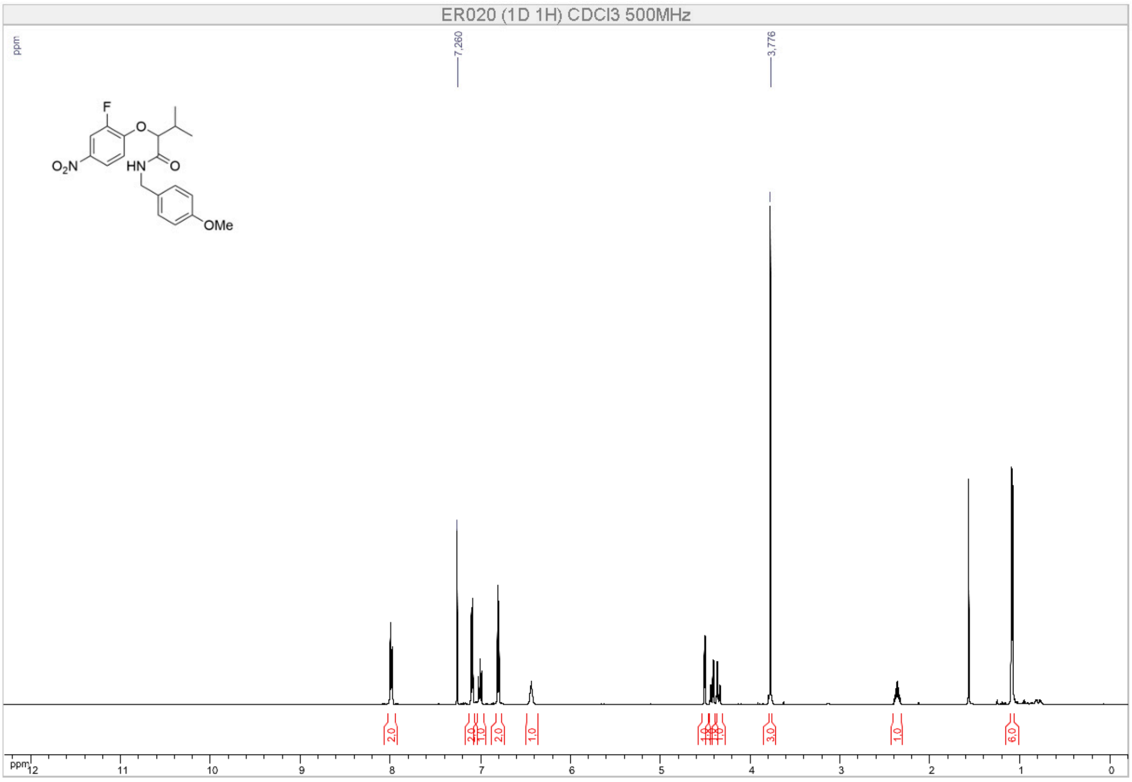

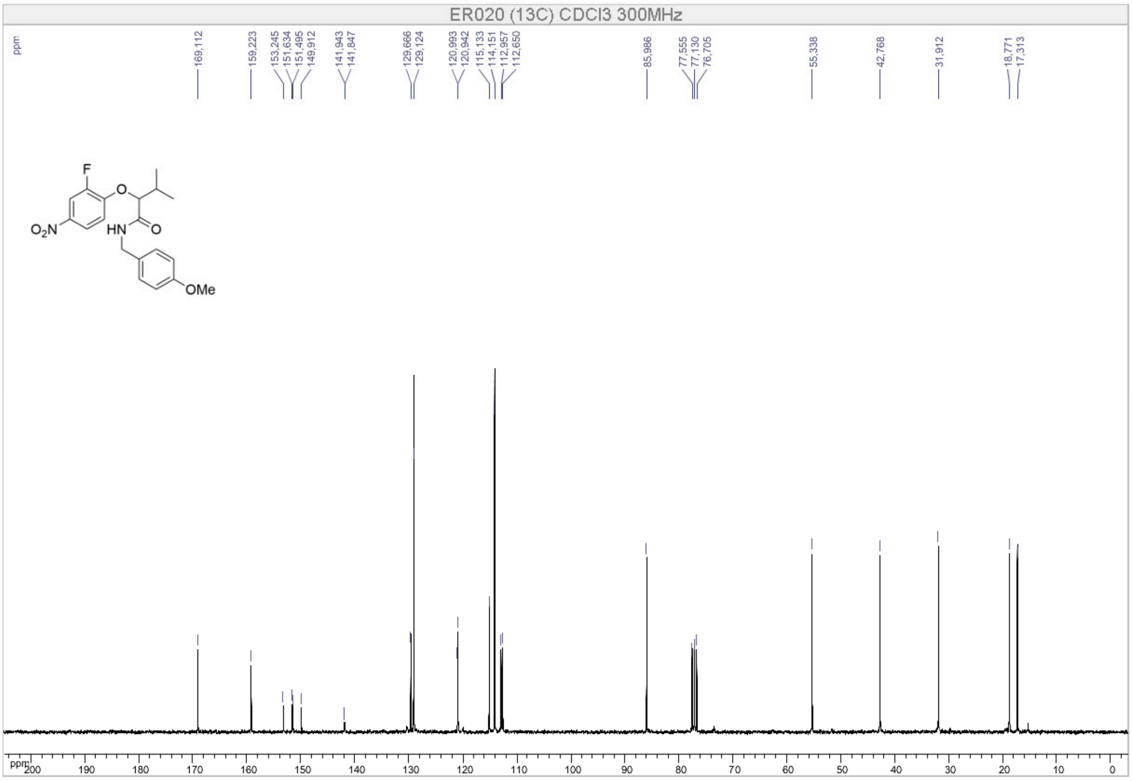

2-(2-Fluoro-4-nitrophenoxy)-3,3-dimethyl-N-phenethylbutanamide (**4h**)

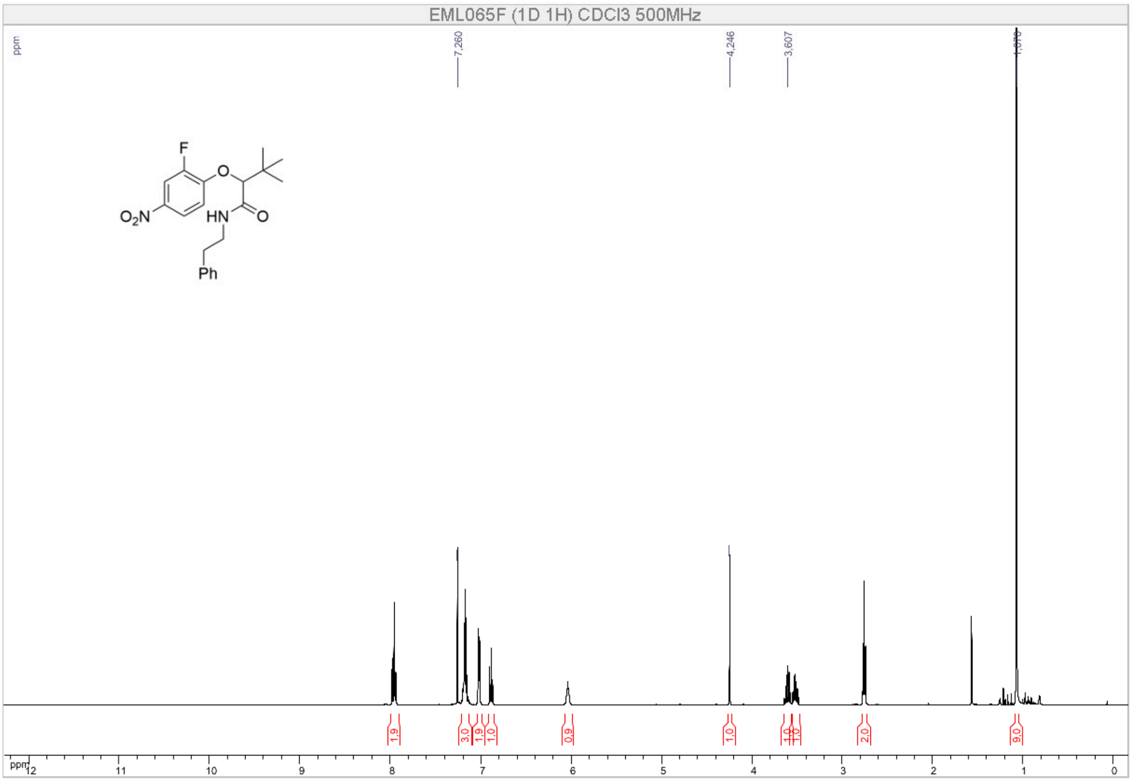

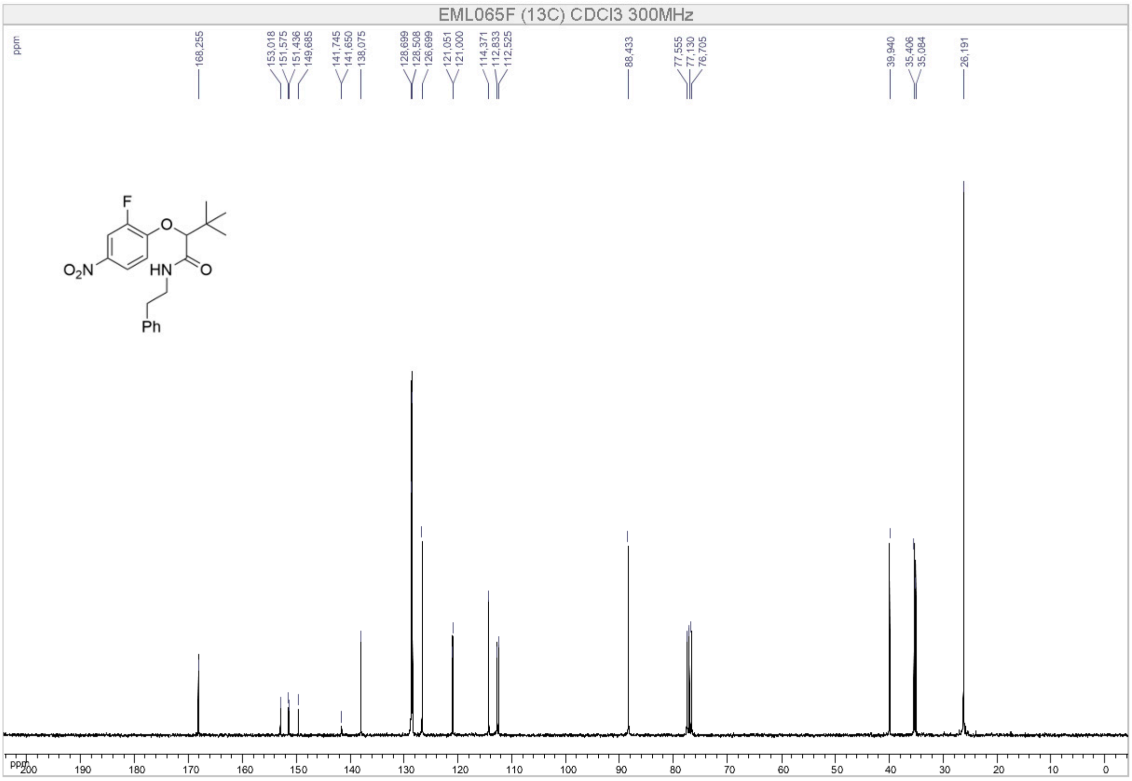

2-(2-Fluoro-4-nitrophenoxy)-N-(4-methoxybenzyl)-3,3-dimethylhex-5-enamide (**4i**)

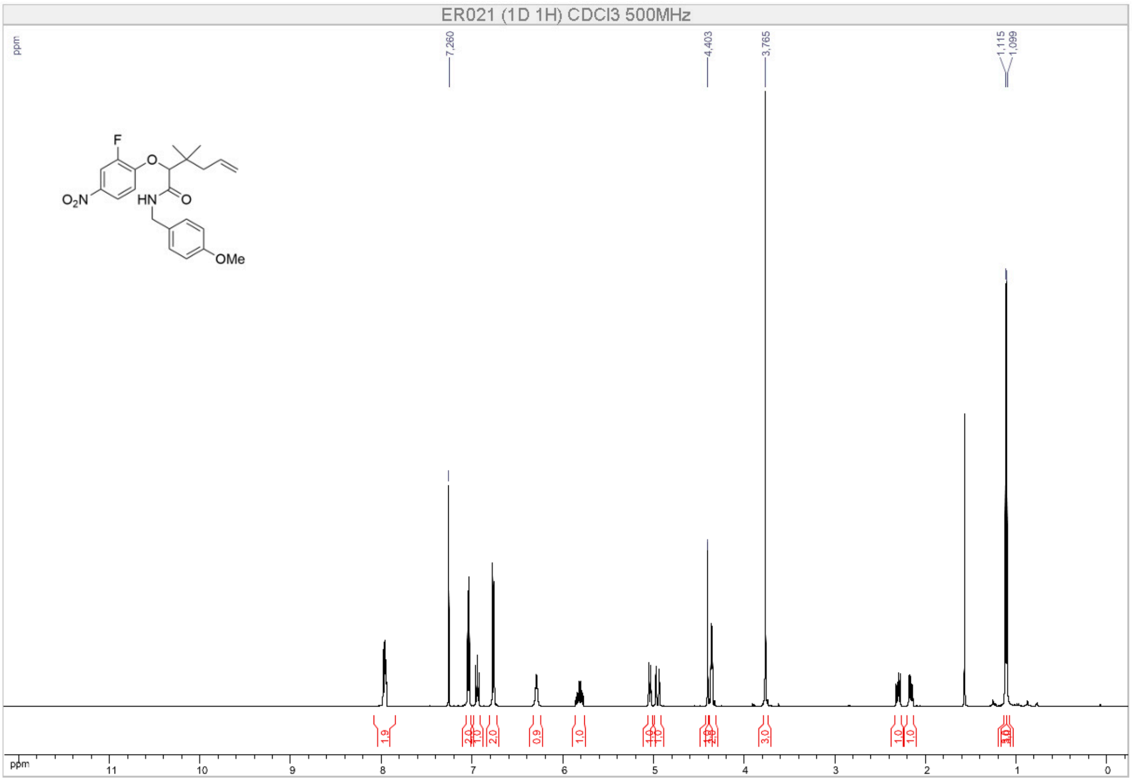

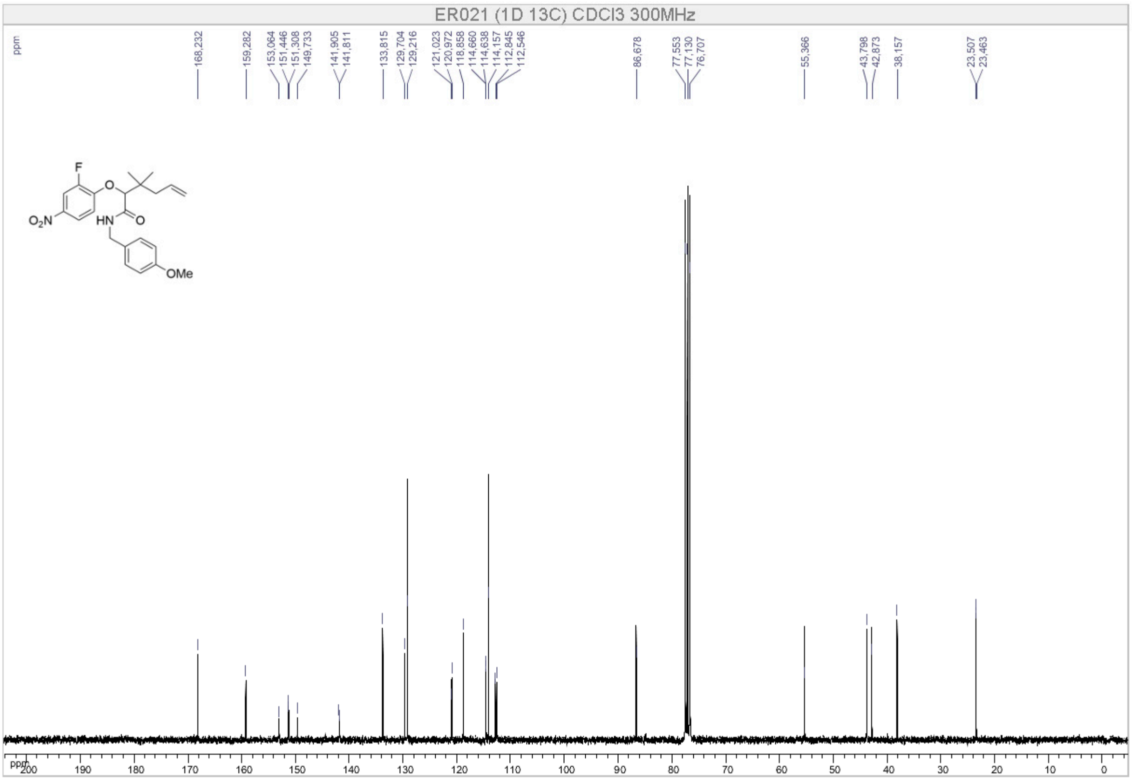

N-Cyclohexyl-2-(2-fluoro-4-nitrophenoxy)butanamide (**4j**)

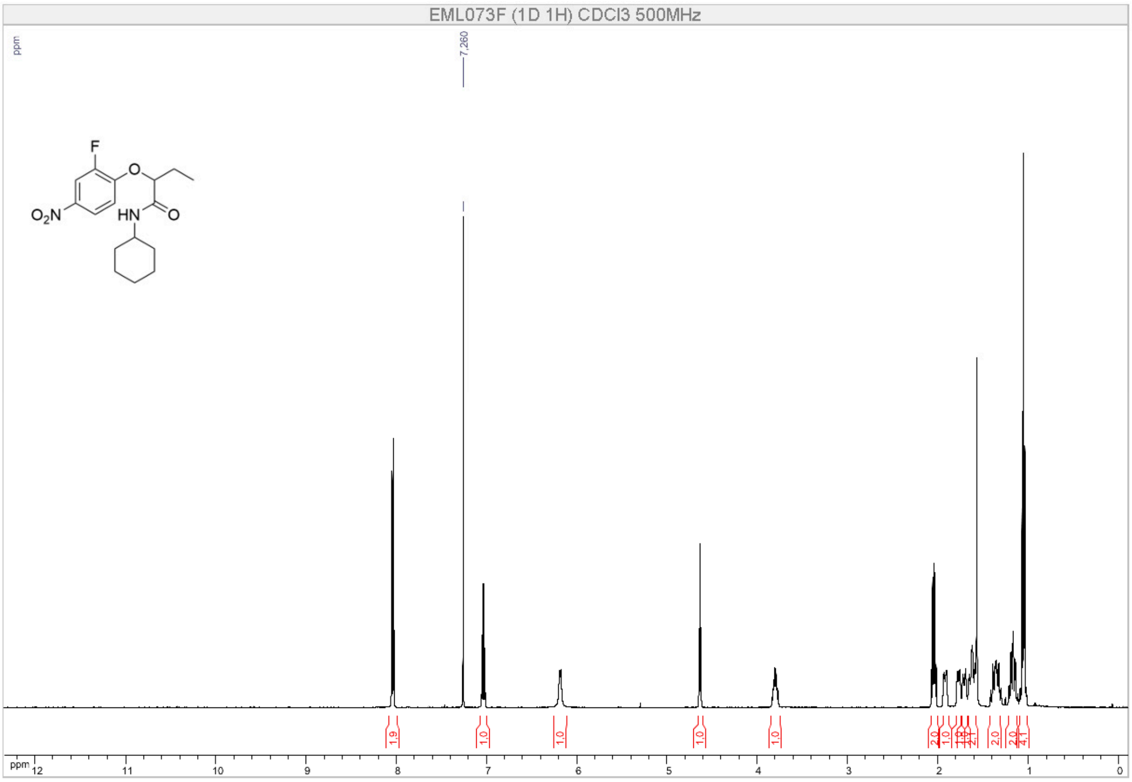

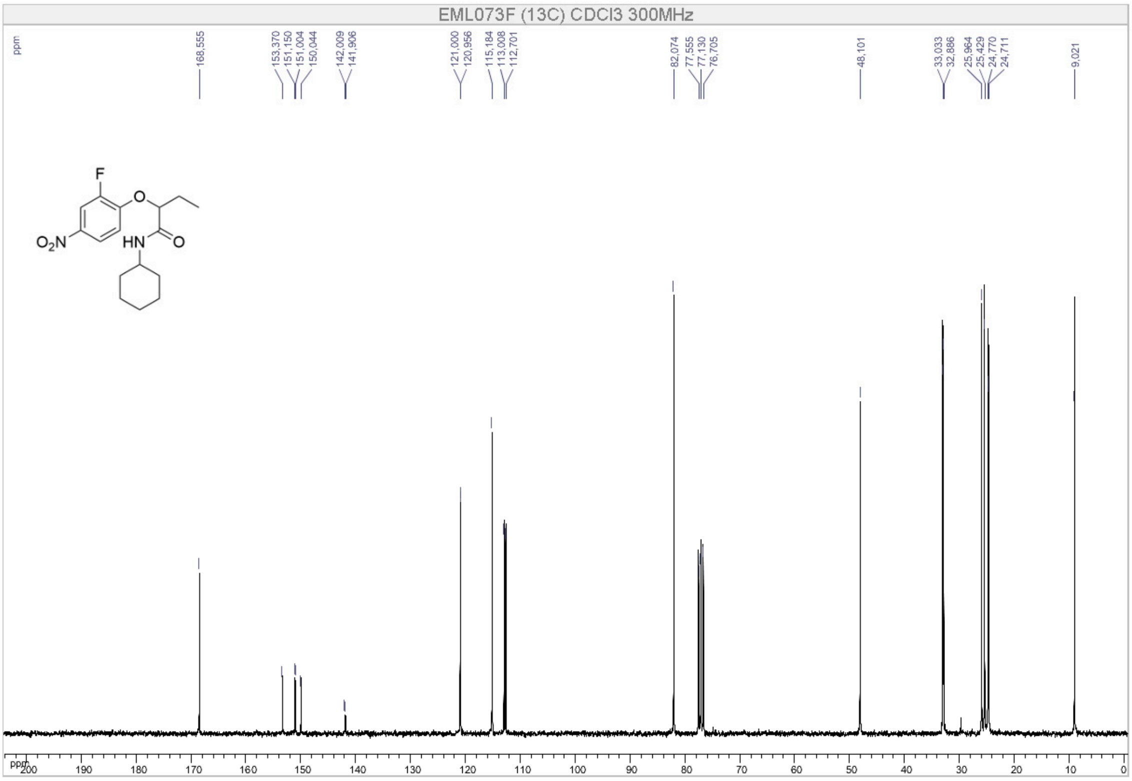

N-(3,4-Dimethoxyphenethyl)-2-(2-fluoro-4-nitrophenoxy)-4,8-dimethylnon-7-enamide (**4k**)

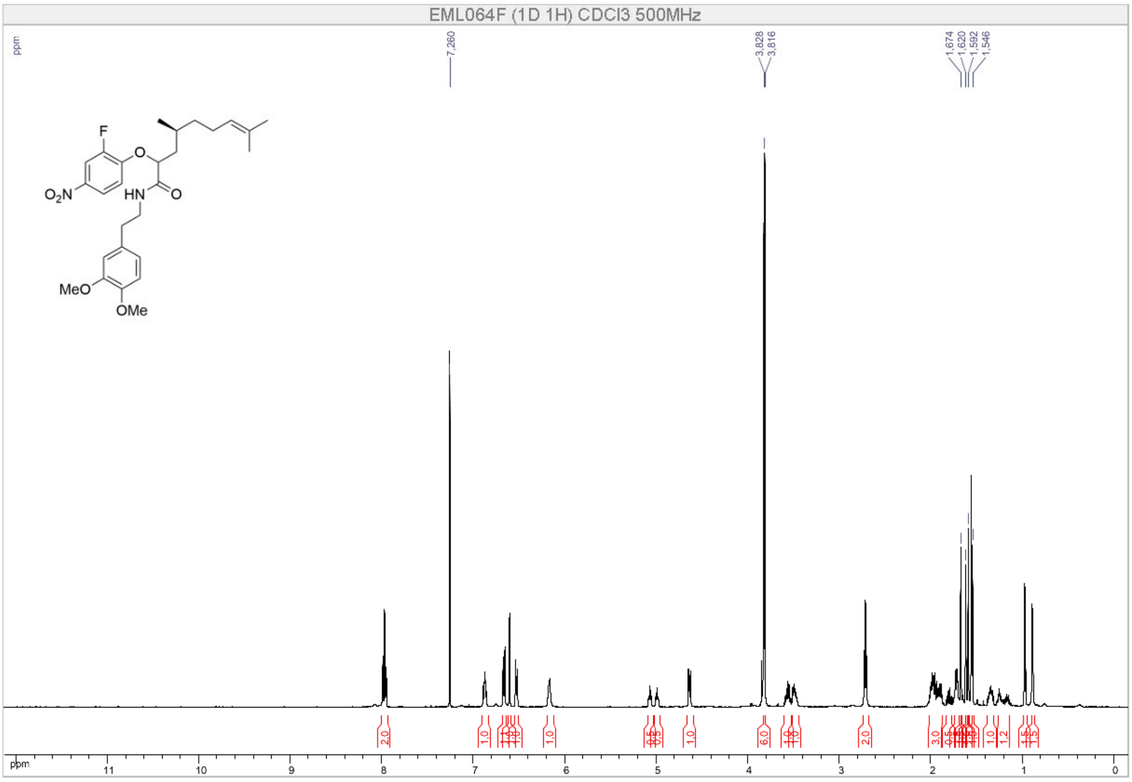

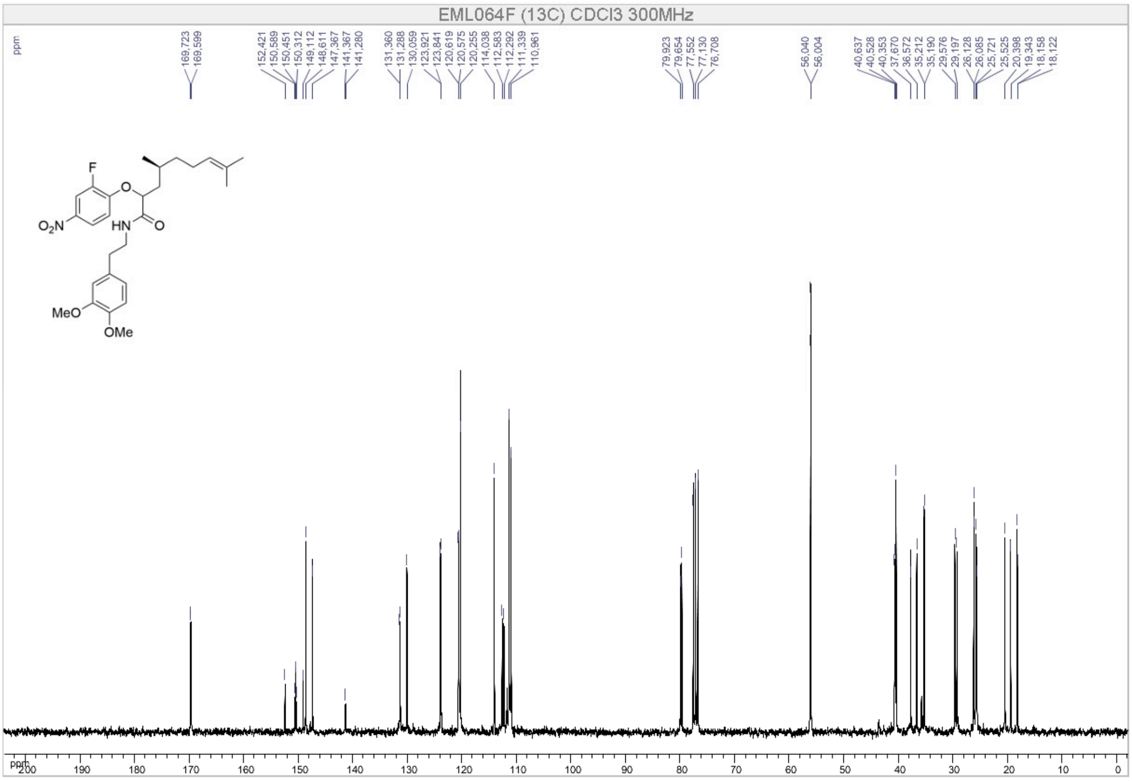

N-(4-Chlorobenzyl)-2-(2-fluoro-4-nitrophenoxy)butanamide (**4l**)

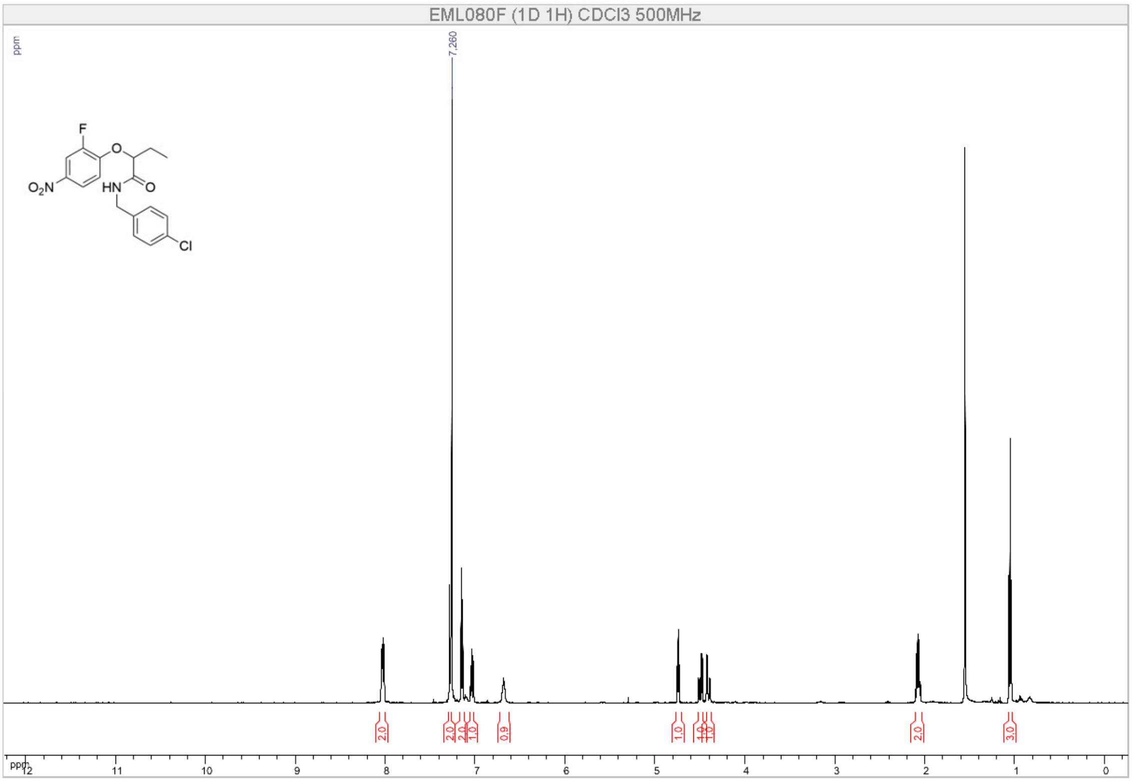

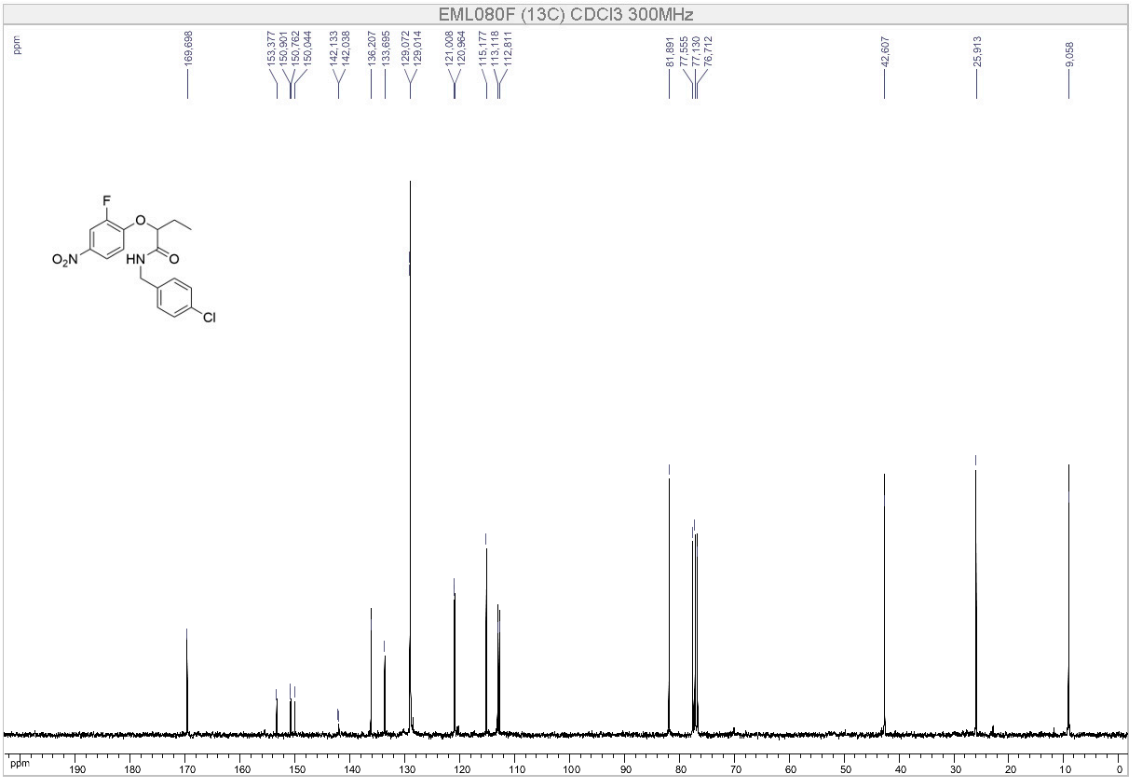

N-(Tert-butyl)-2-(2-fluoro-4-nitrophenoxy)-3-methylbutanamide (**4m**)

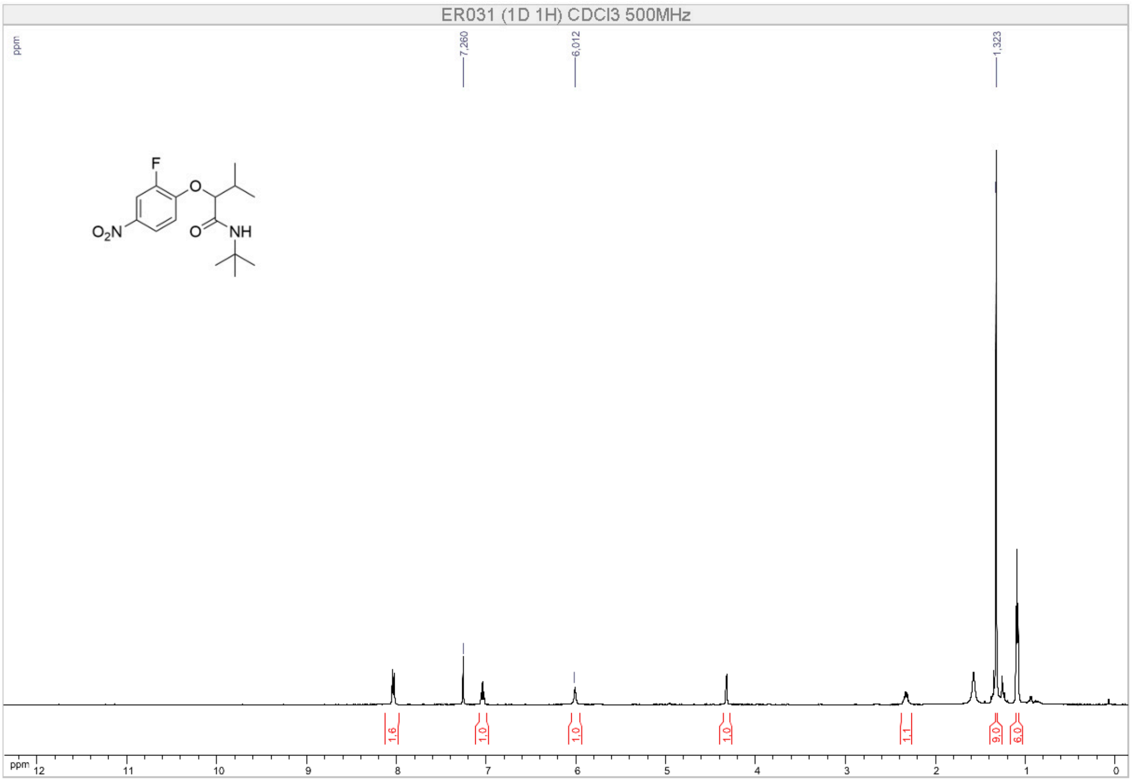

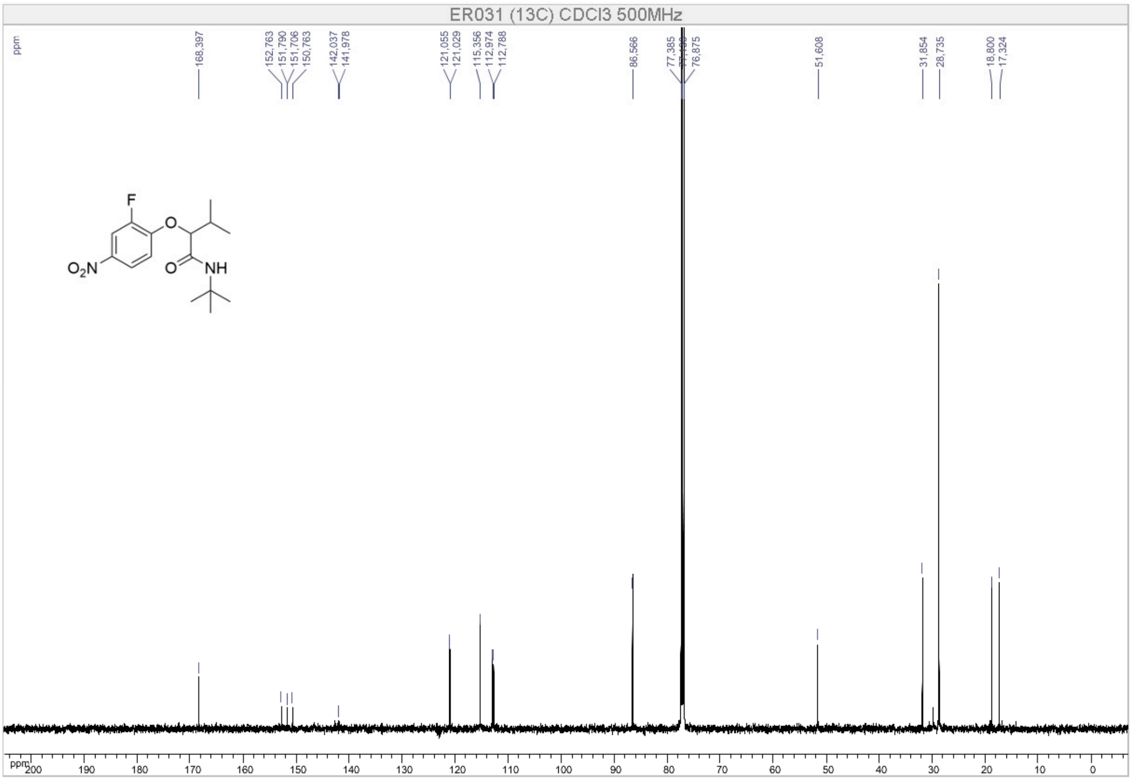

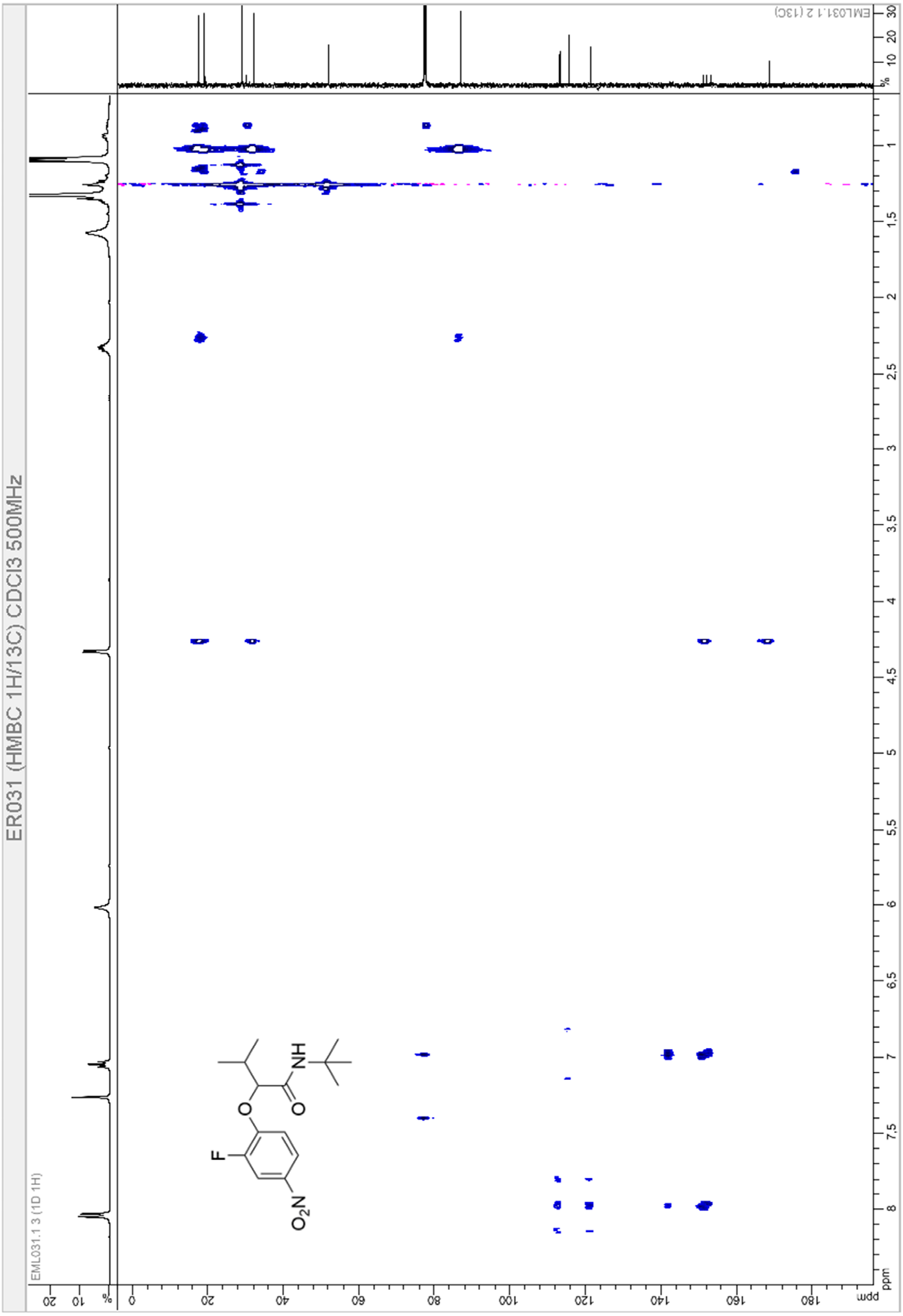
ER031 (HMBC 1H/13C) CDCl3 500MHz
EML031.1 2 (13C)
EML031.1 3 (1D 1H)
ppm
F
O
O₂N
NH
O

N-Cyclohexyl-2-(2-fluoro-4-nitrophenoxy)-3,3-dimethylbutanamide (**4n**)

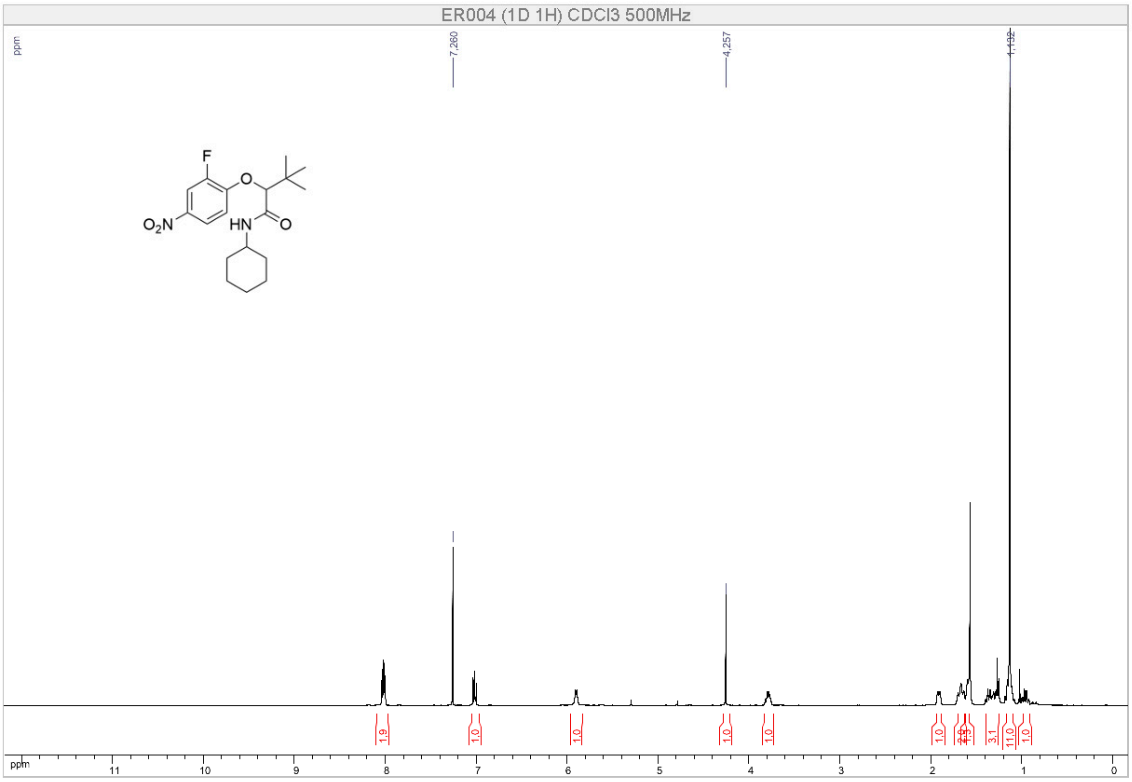

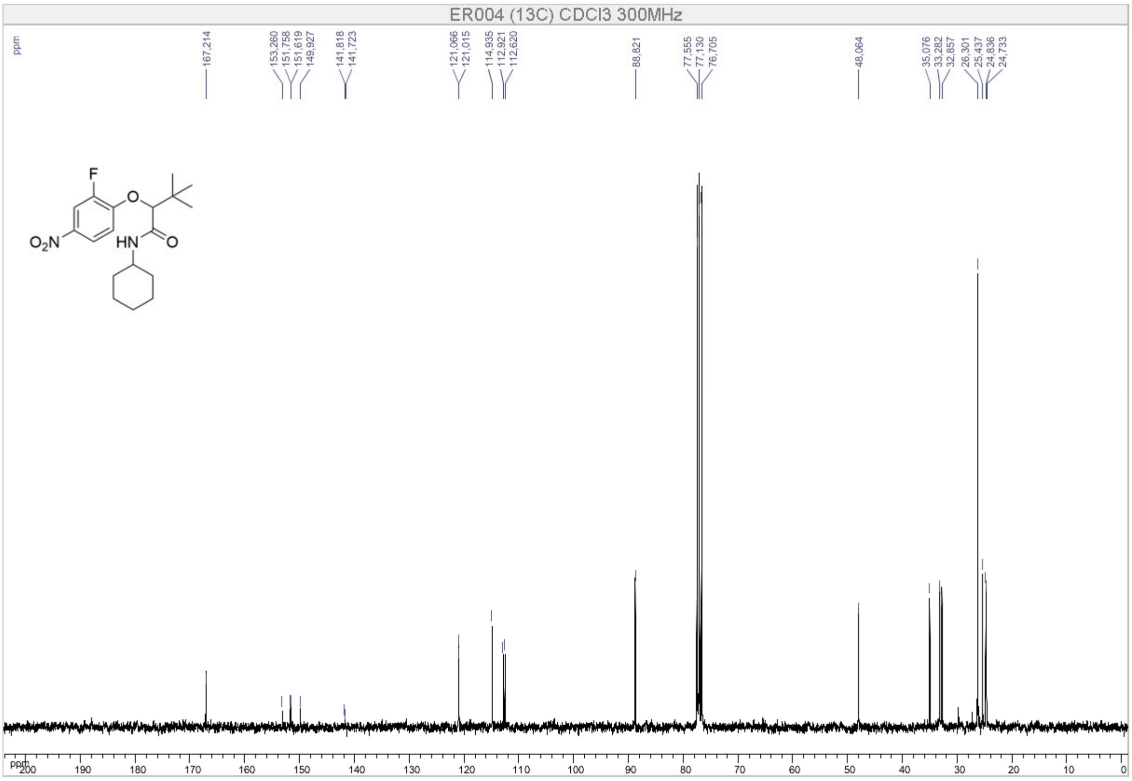

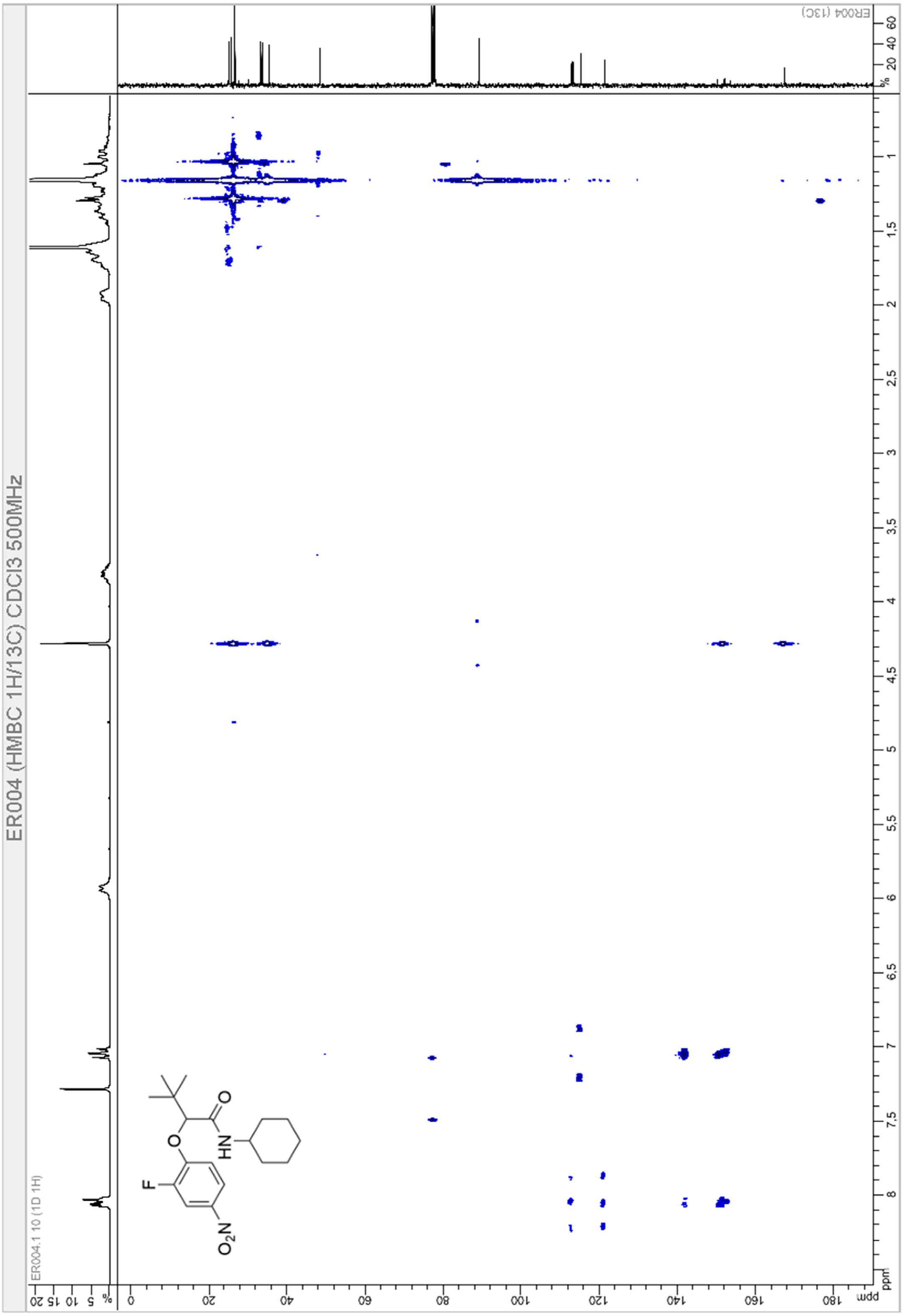
ER004 (HMBC 1H/13C) CDCl3 500MHz
ER004 (13C)
ER004.1.10 (1D 1H)
F
O
O
HN
O_2N
ppm

2-Isopropyl-2H-benzo[b][1,4]oxazin-3(4H)-one (**5a**)

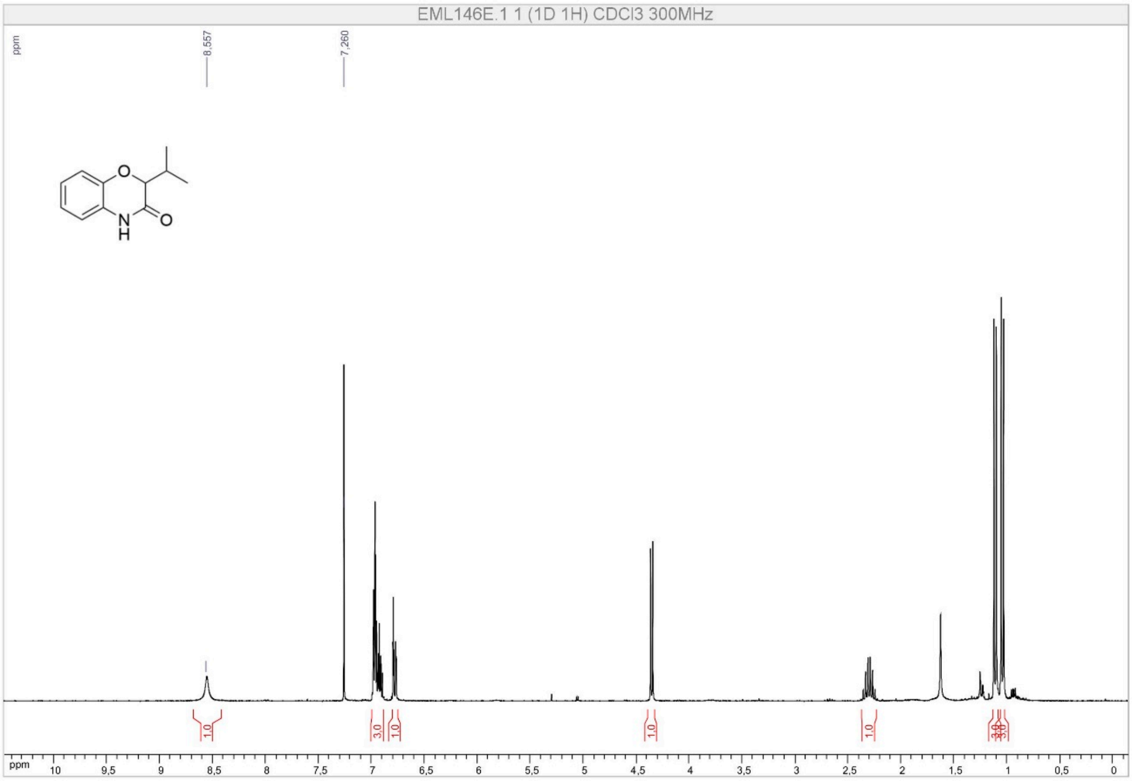

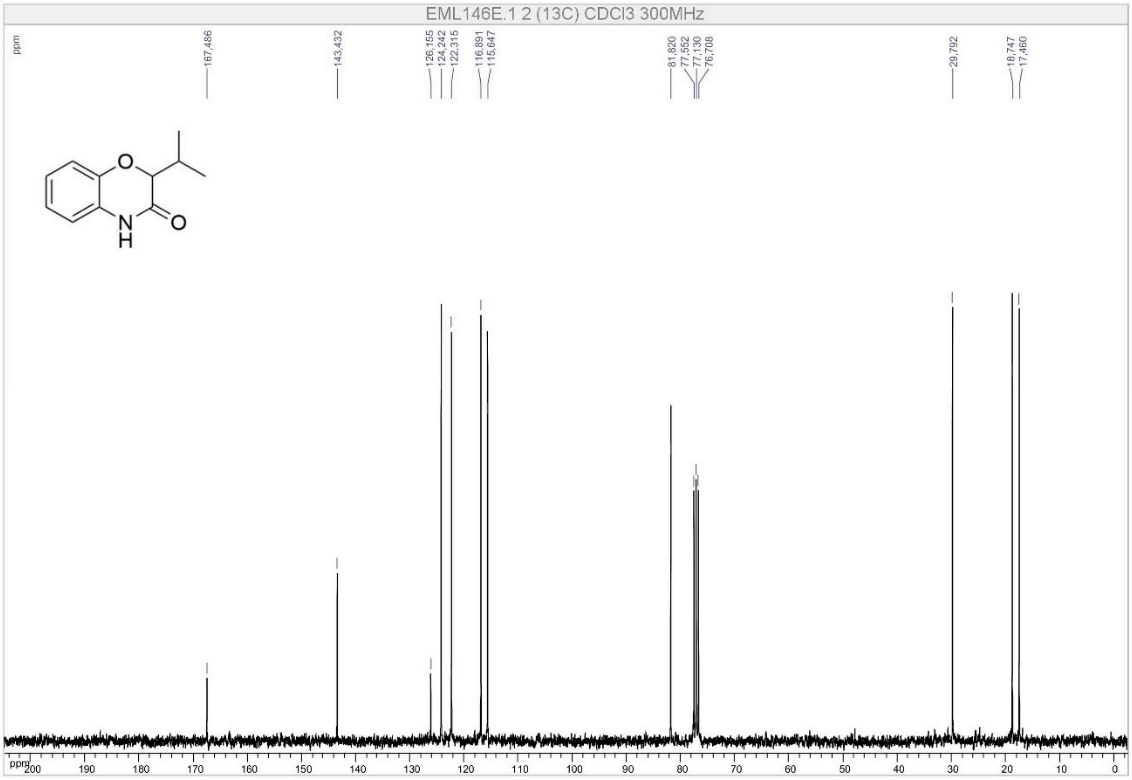

2-Phenethyl-2H-benzo[b][1,4]oxazin-3(4H)-one (**5b**)

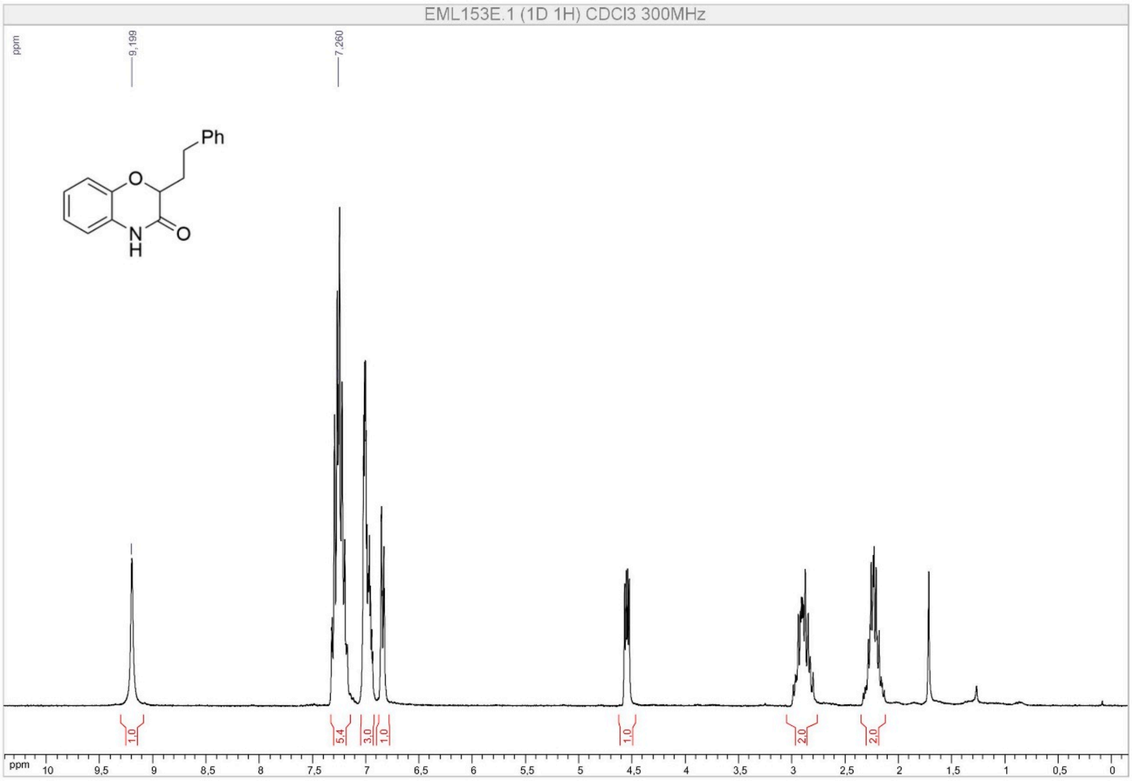

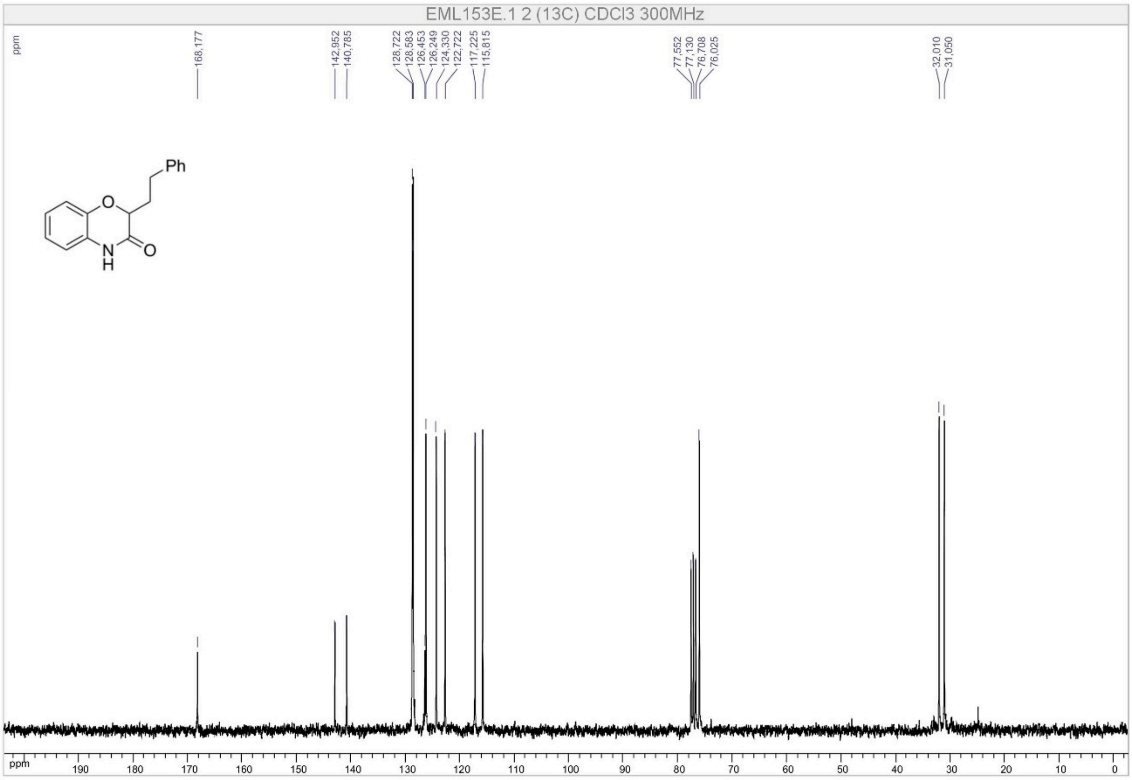

2-Isopropyl-6-methoxy-2H-benzo[b][1,4]oxazin-3(4H)-one (**5c**)

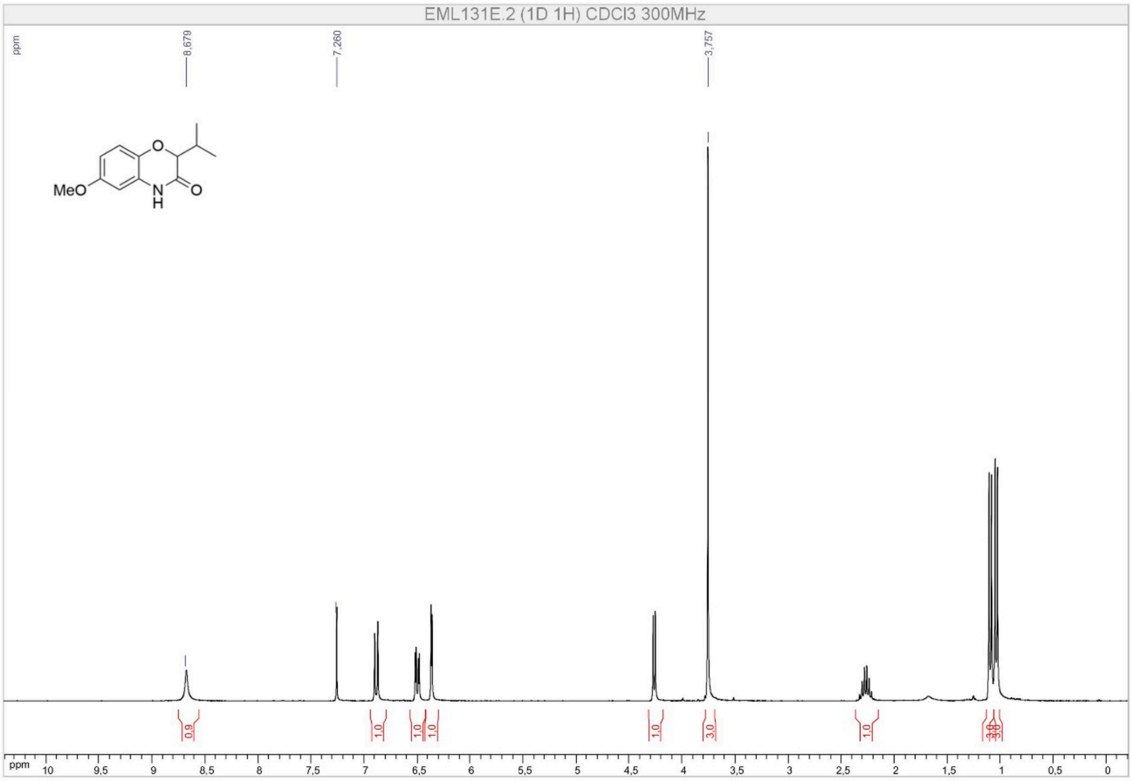

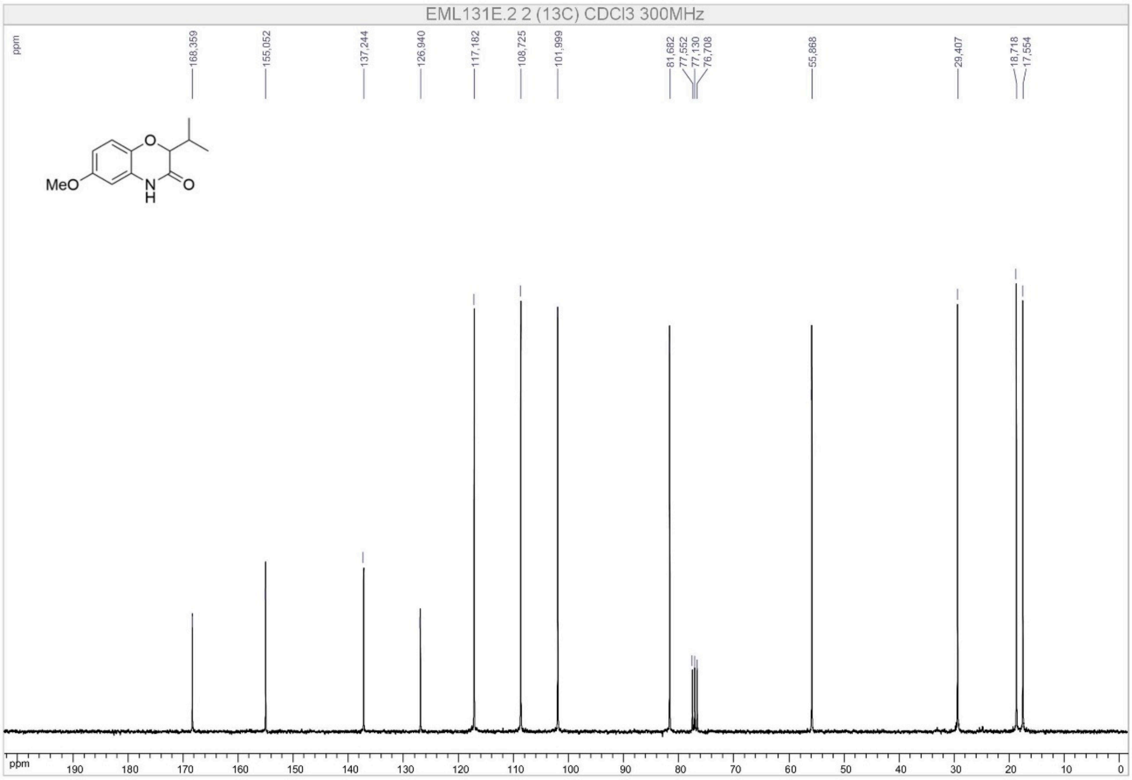

6-Chloro-2-ethyl-2H-benzo[b][1,4]oxazin-3(4H)-one (**5d**)

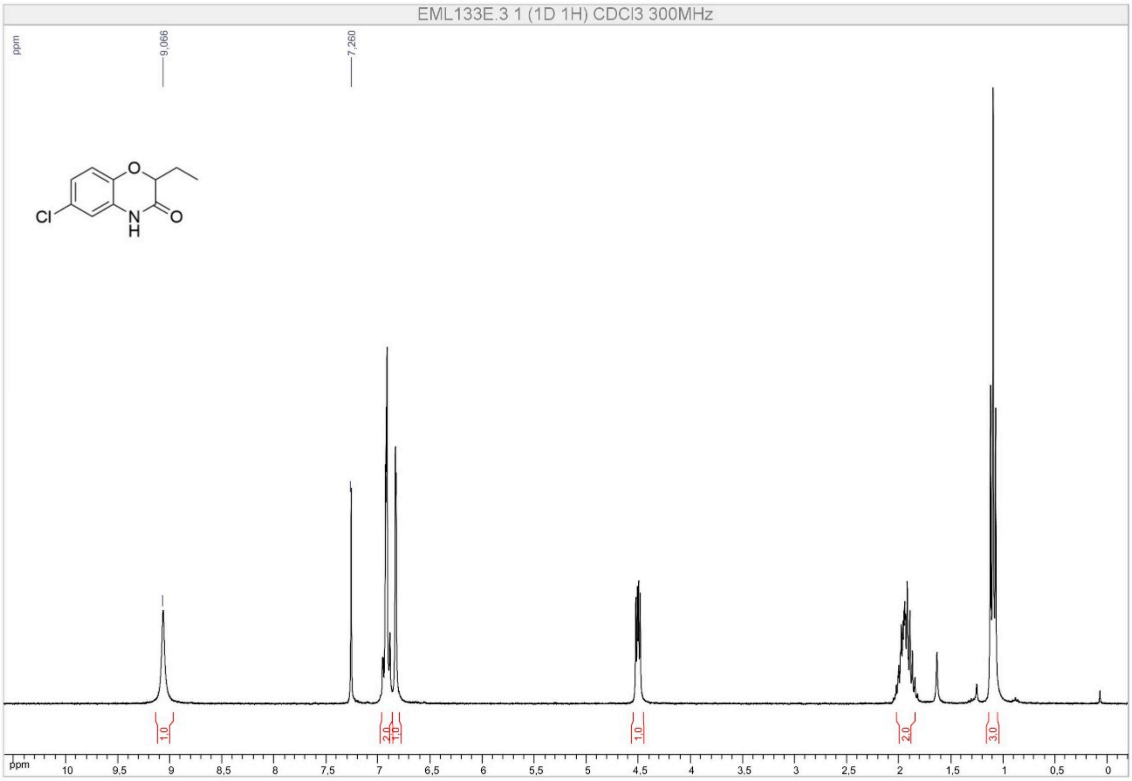

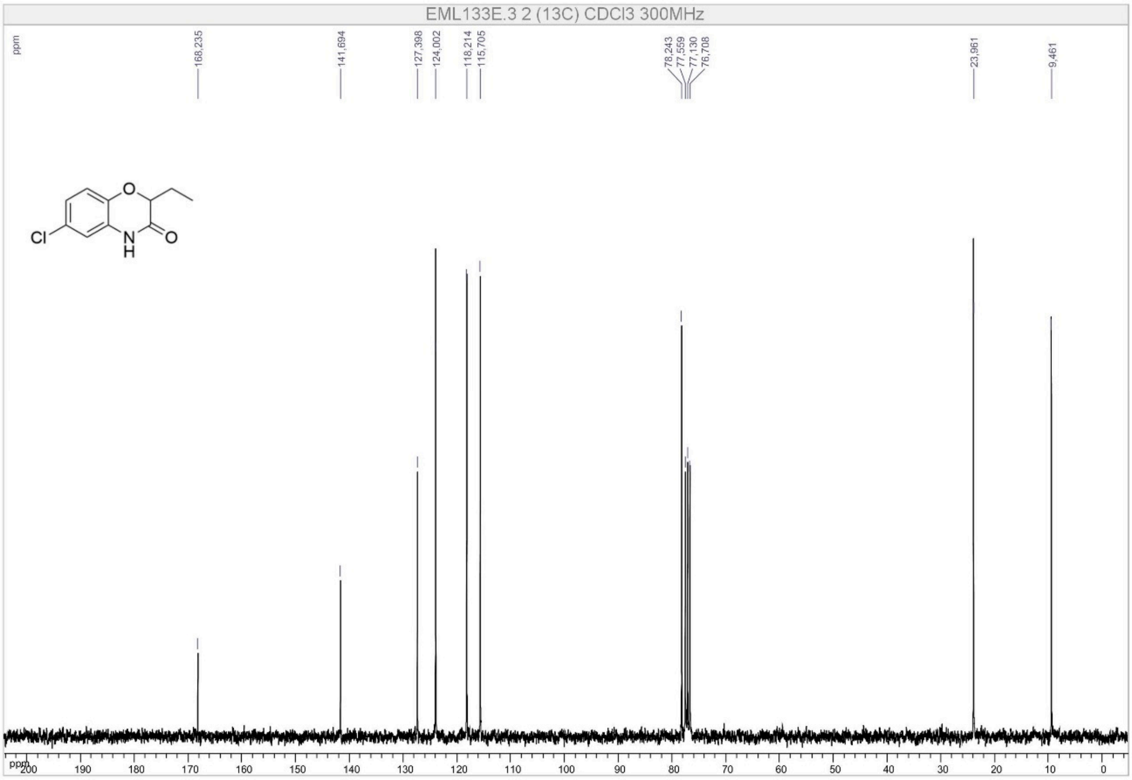

2-(4-Methoxyphenyl)-6-methyl-2H-benzo[b][1,4]oxazin-3(4H)-one (**5e**)

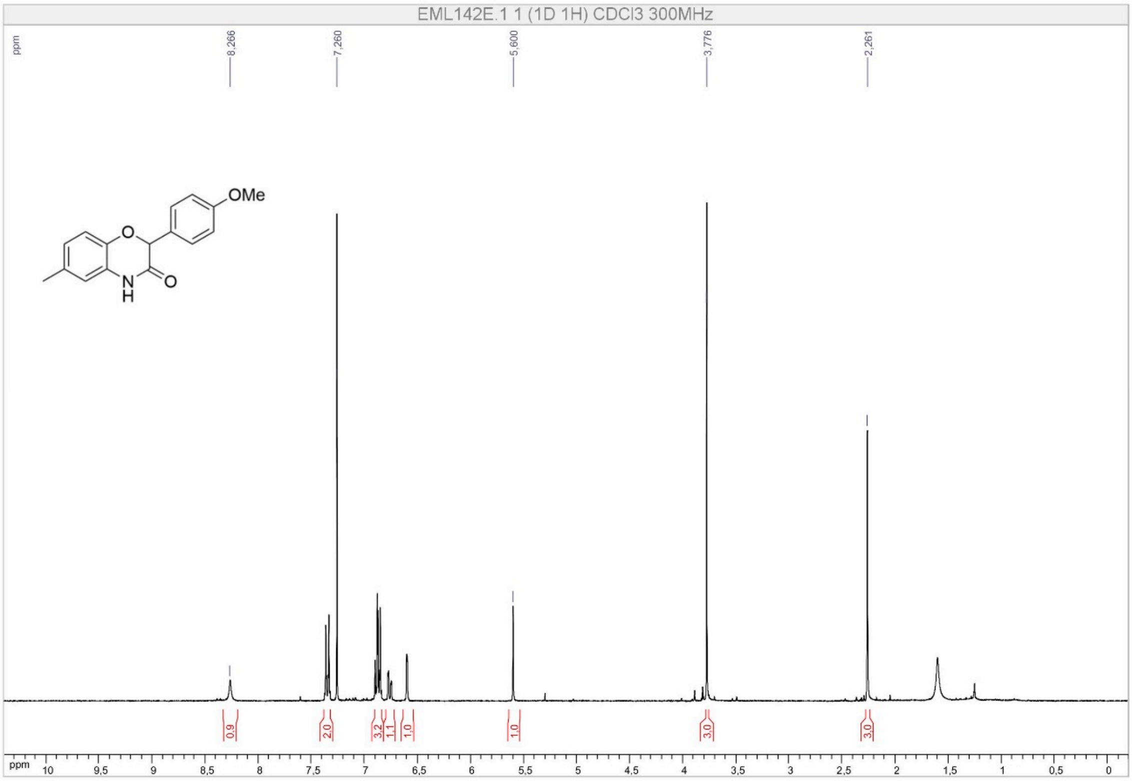

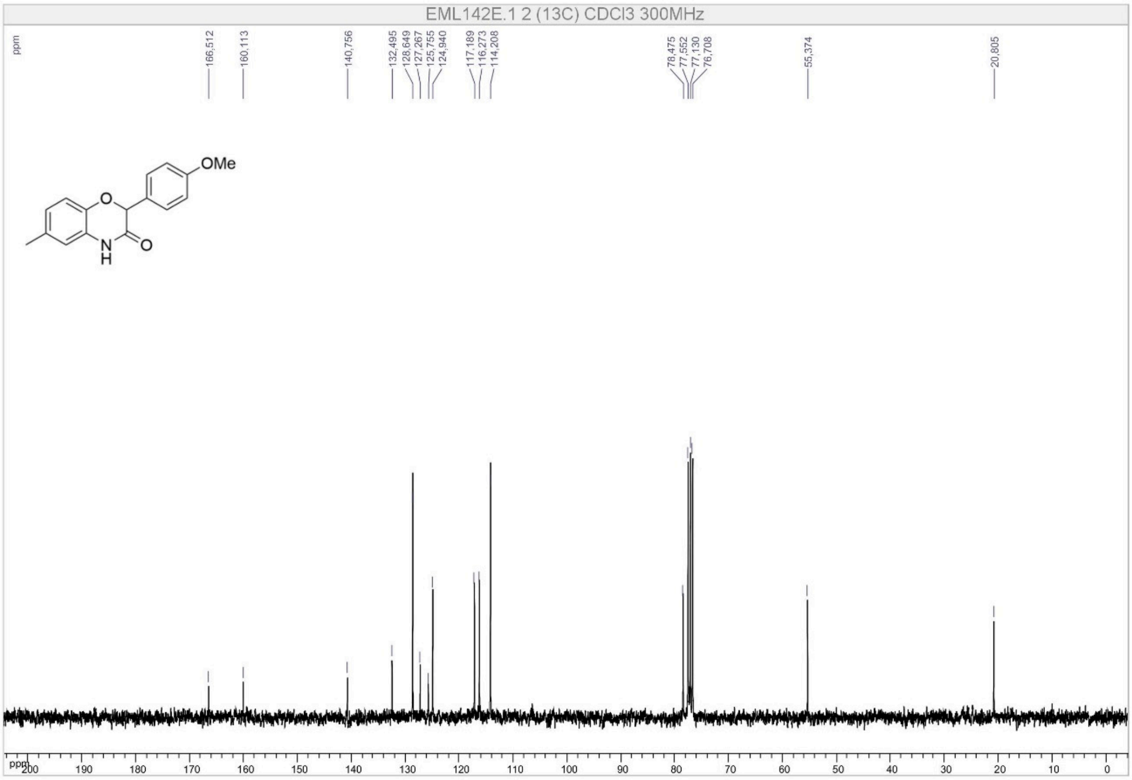

2-(3-Fluorophenyl)-2H-benzo[b][1,4]oxazin-3(4H)-one (**5f**)

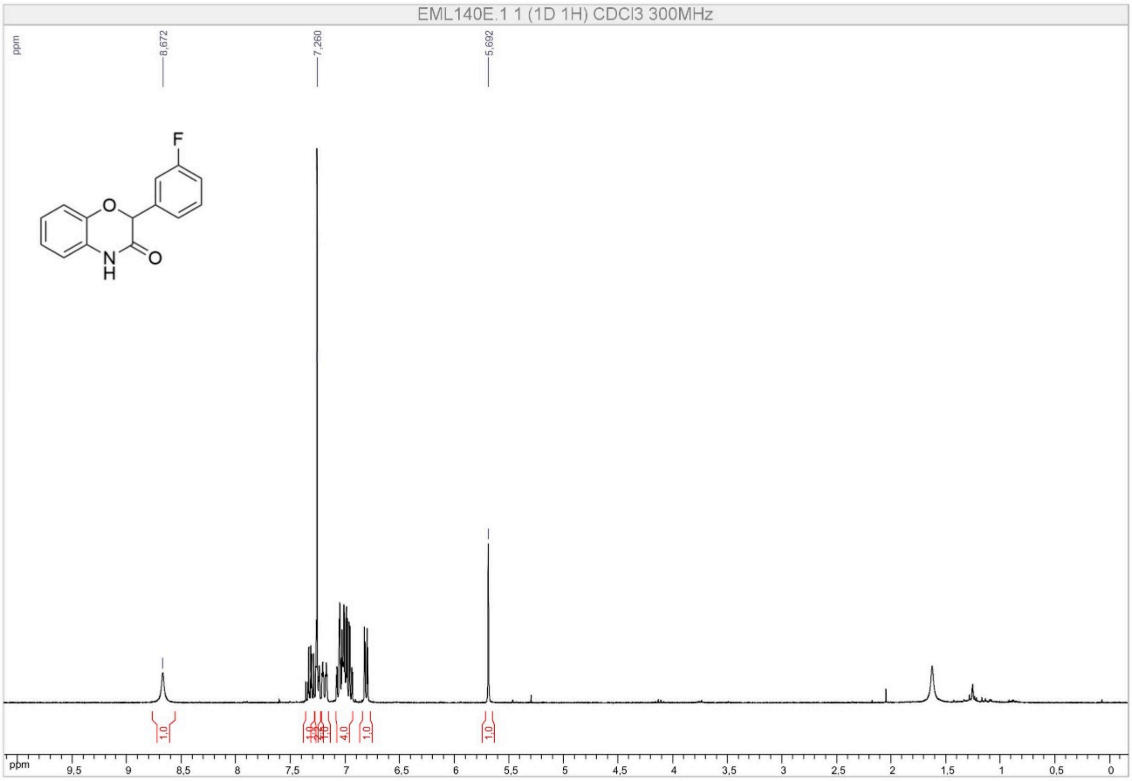

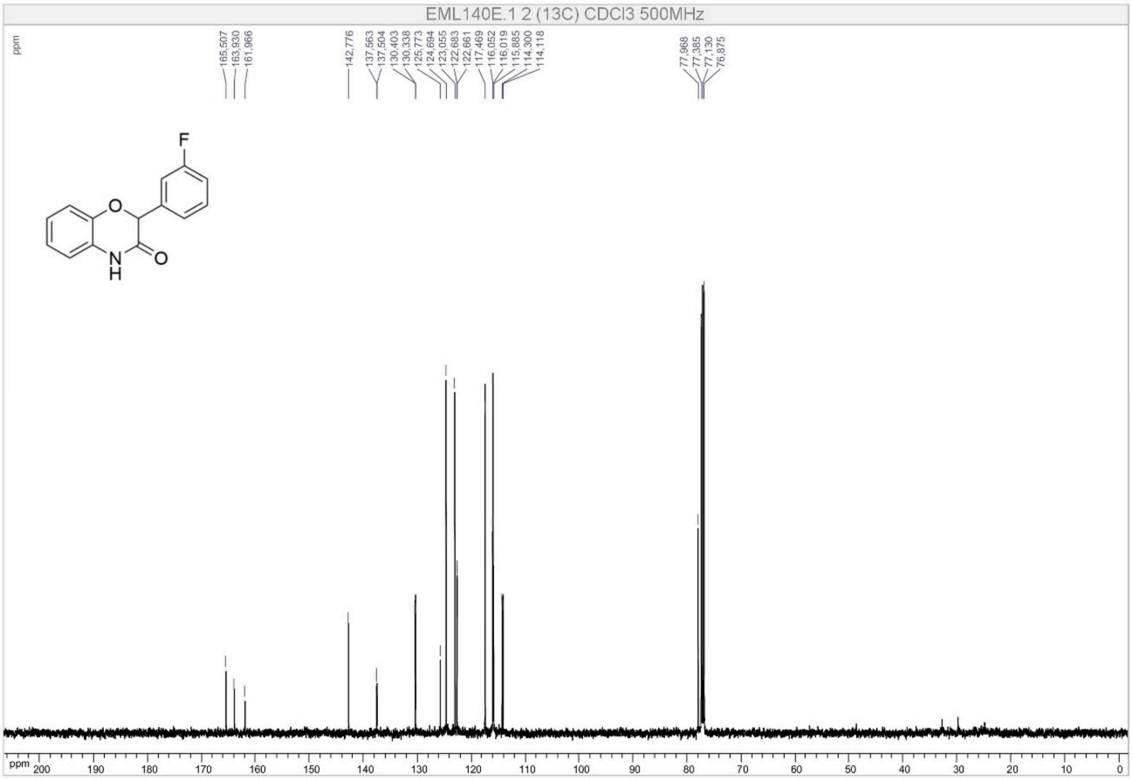

2-(Methoxymethyl)-2-methyl-2H-benzo[b][1,4]oxazin-3(4H)-one (**5g**)

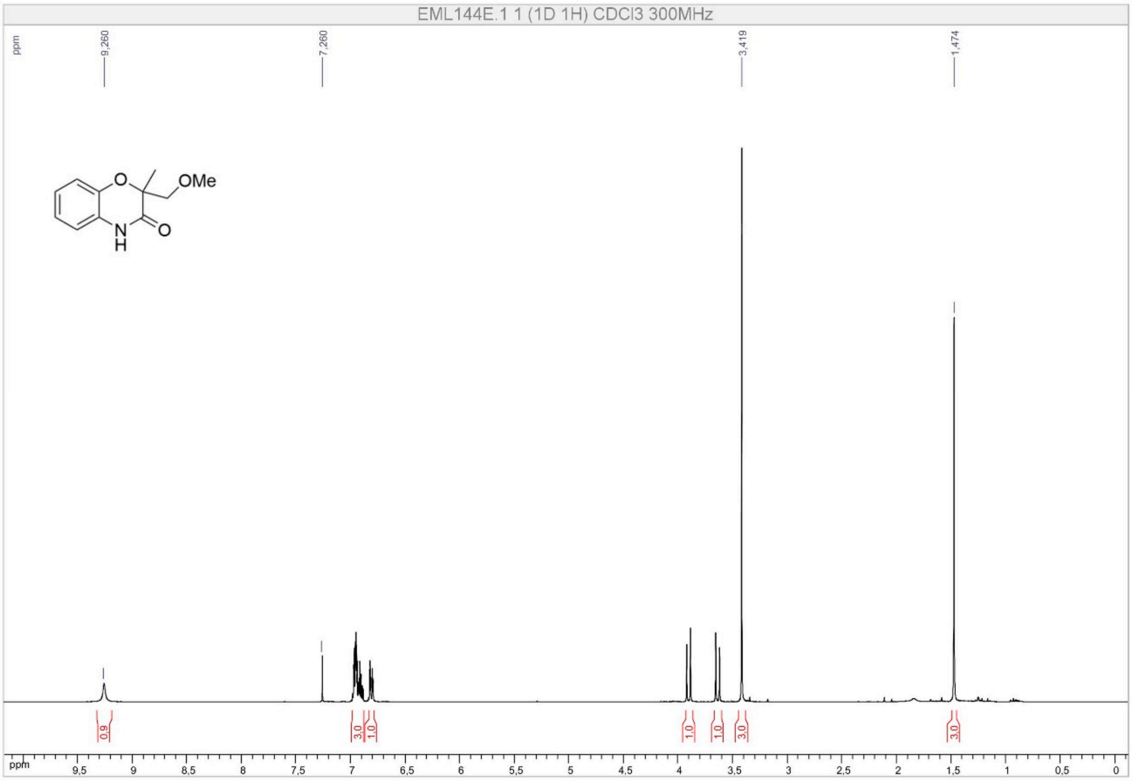

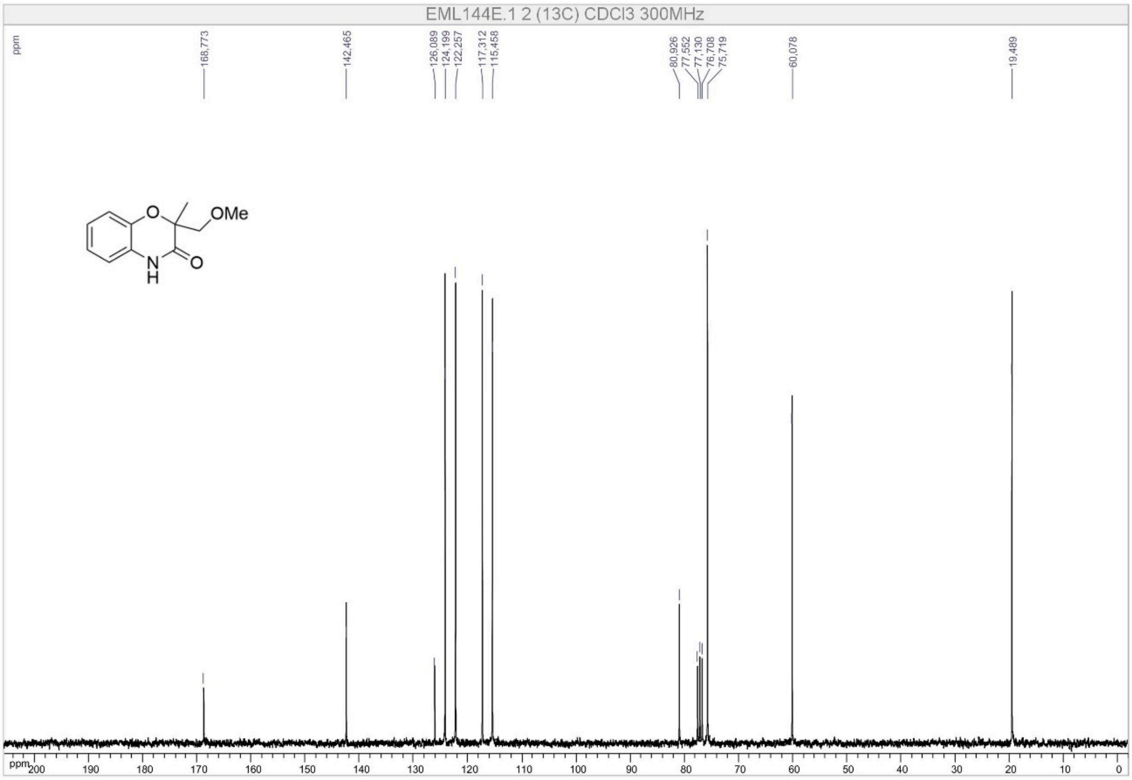

6-Bromospiro[benzo[b][1,4]oxazine-2,1′-cyclobutan]-3(4H)-one (**5h**)

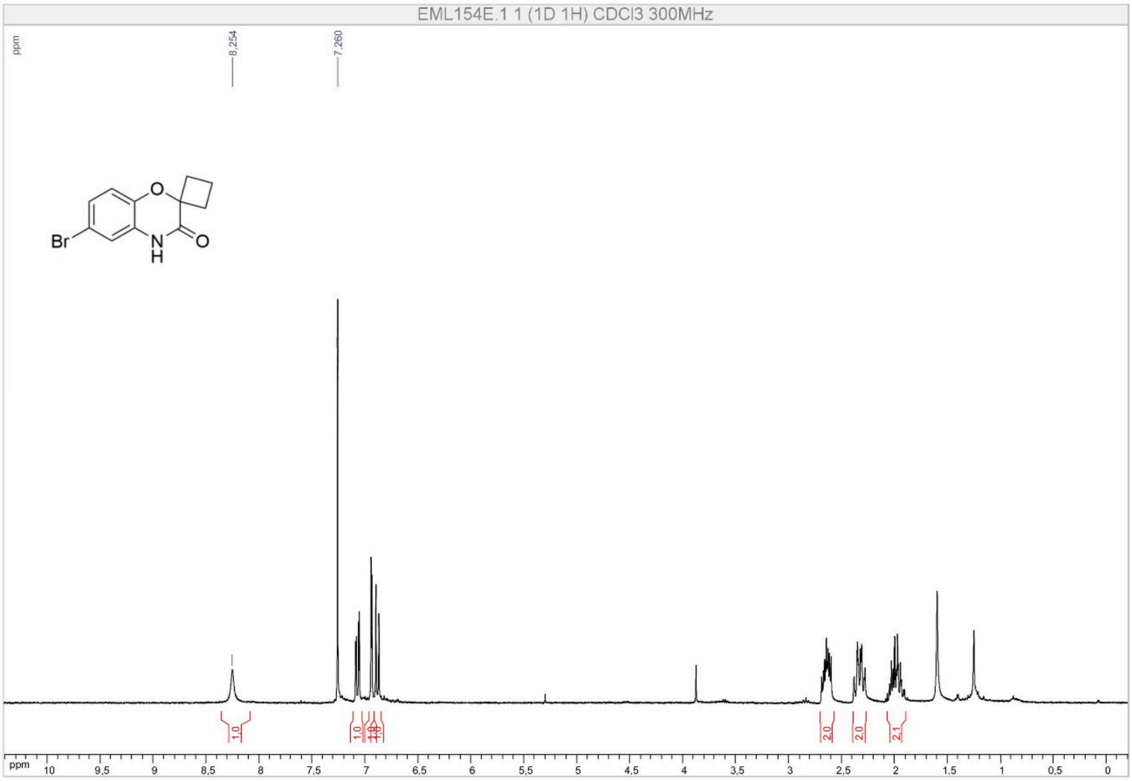

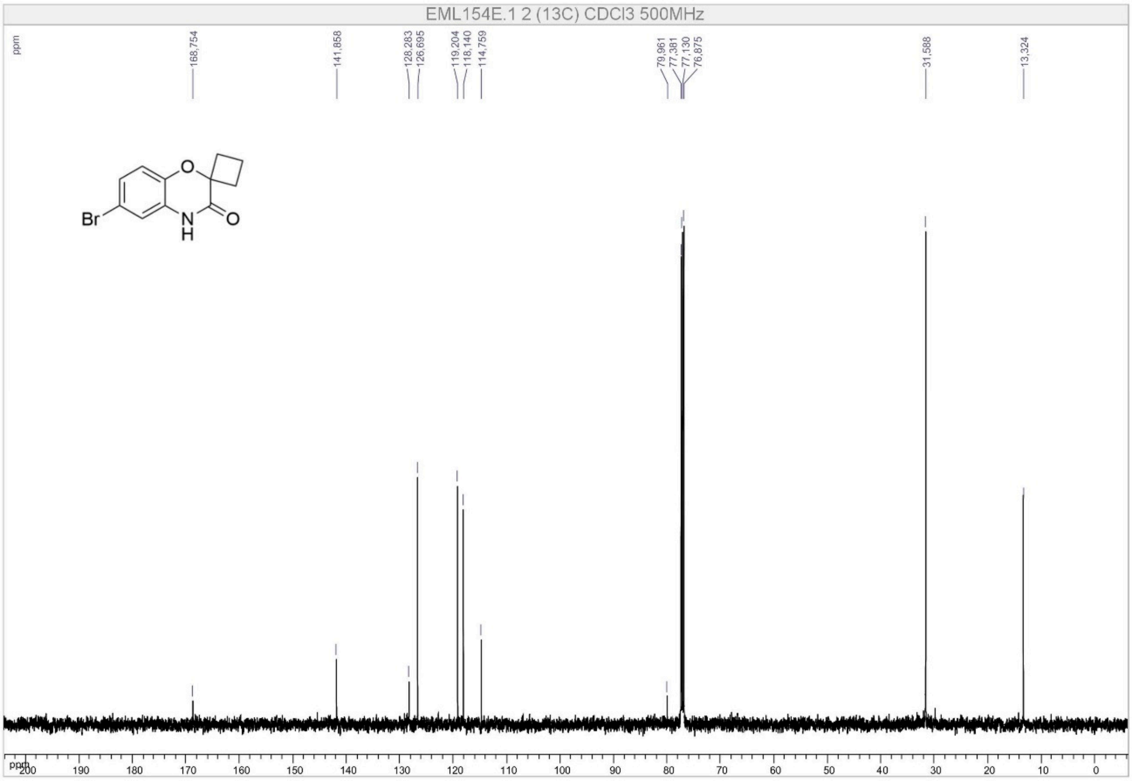

6-Chlorospiro[benzo[b][1,4]oxazine-2,1′-cyclobutan]-3(4H)-one (**5i**)

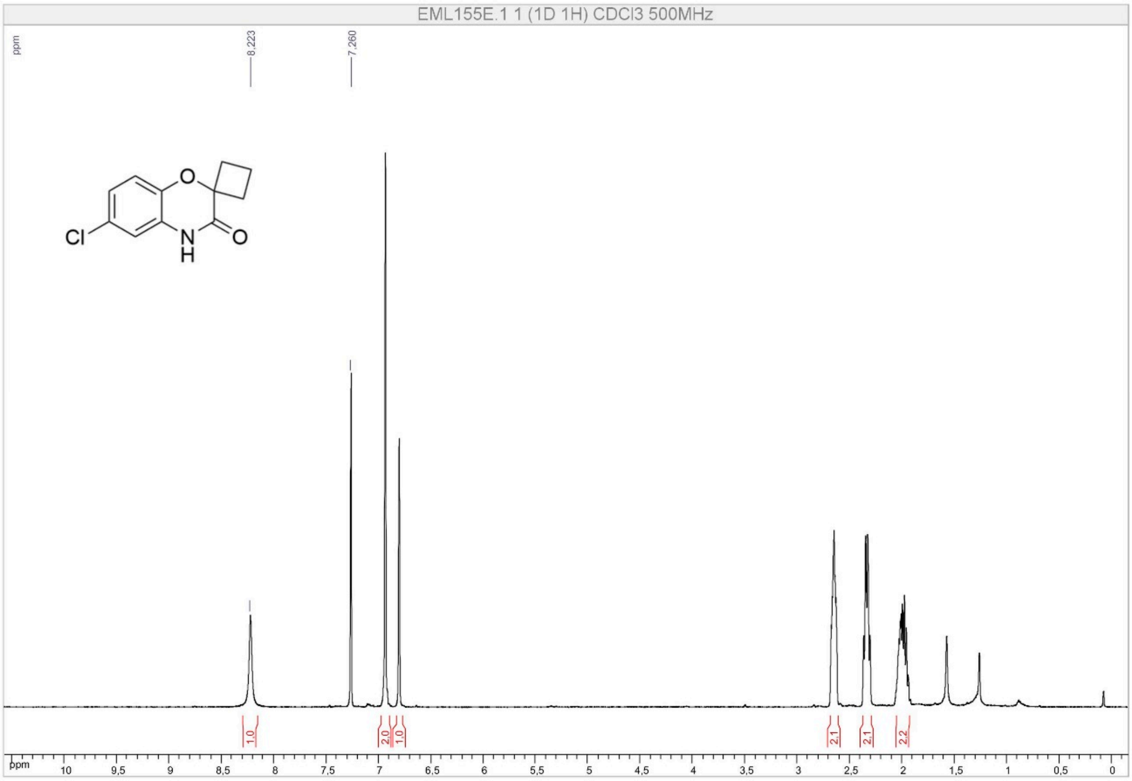

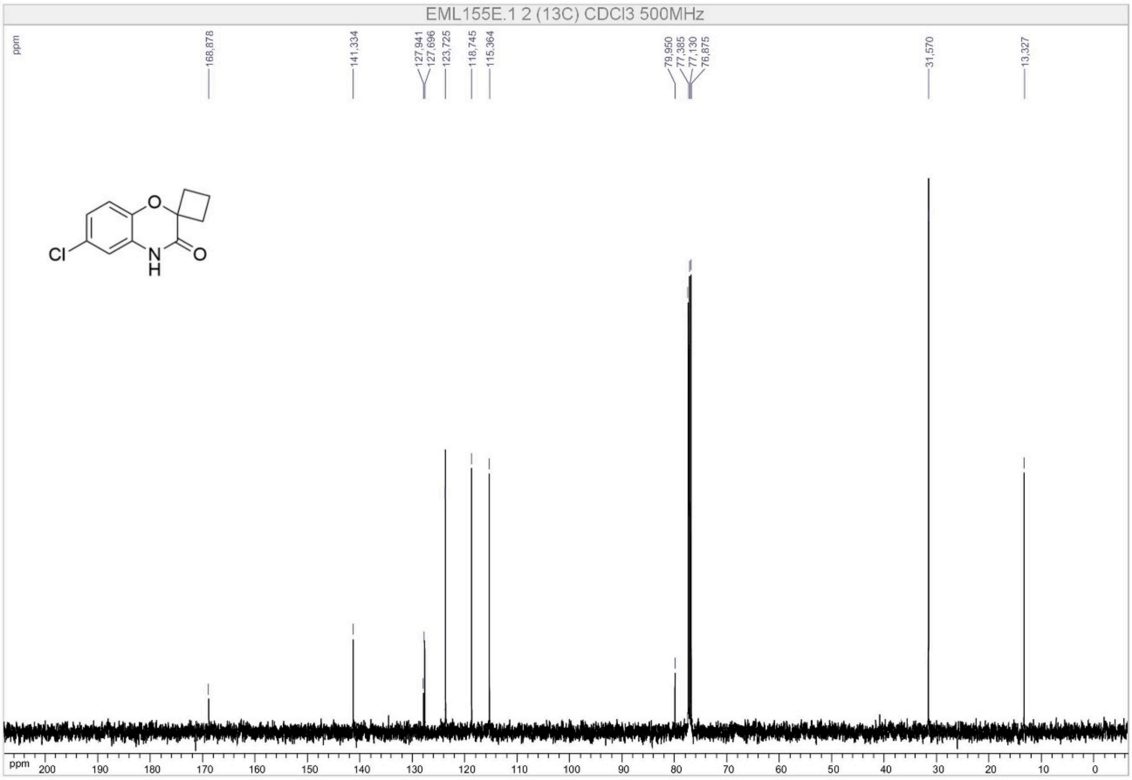

2-Methyl-2-(trifluoromethyl)-2H-benzo[b][1,4]oxazin-3(4H)-one (**5j**)

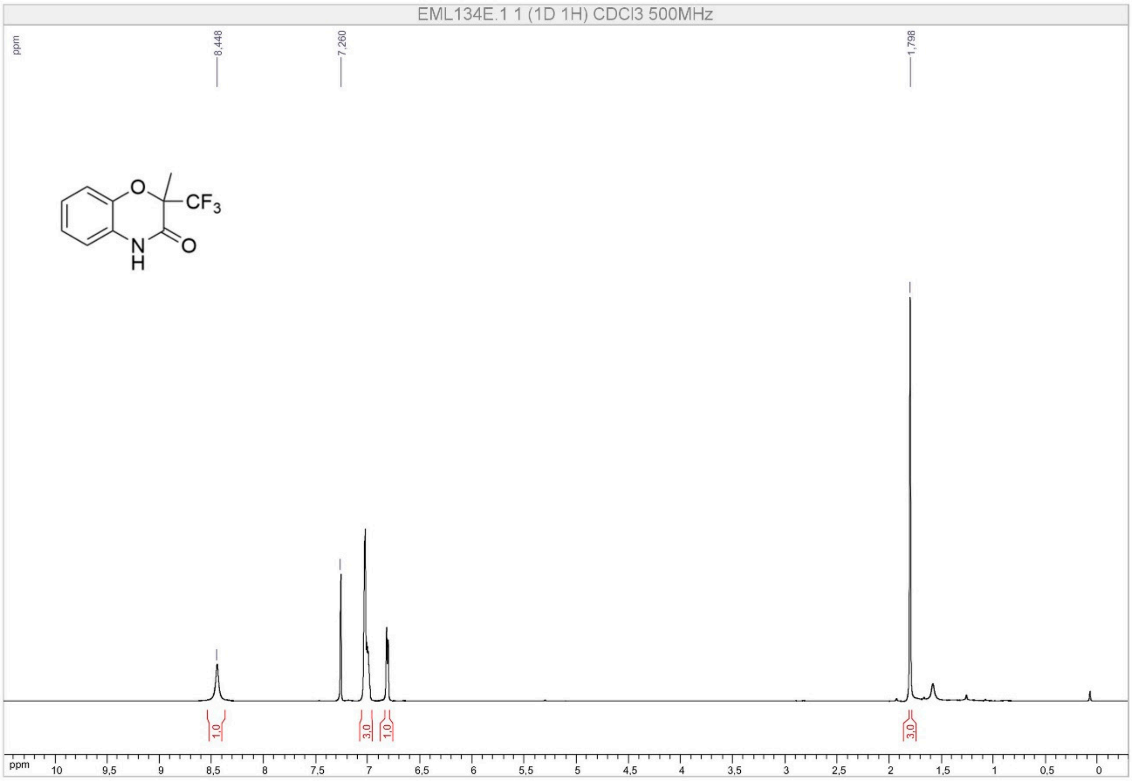

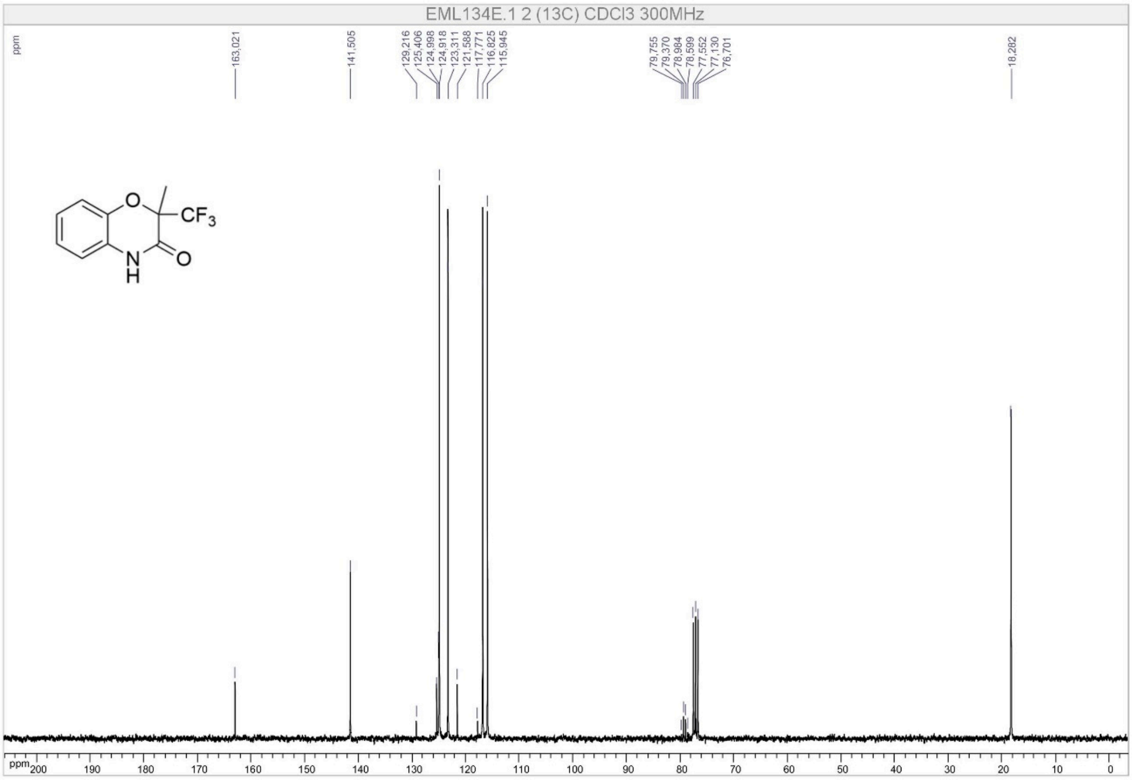

4-Cyclohexyl-2-isopropyl-2H-benzo[b][1,4]oxazin-3(4H)-one (**6a**)

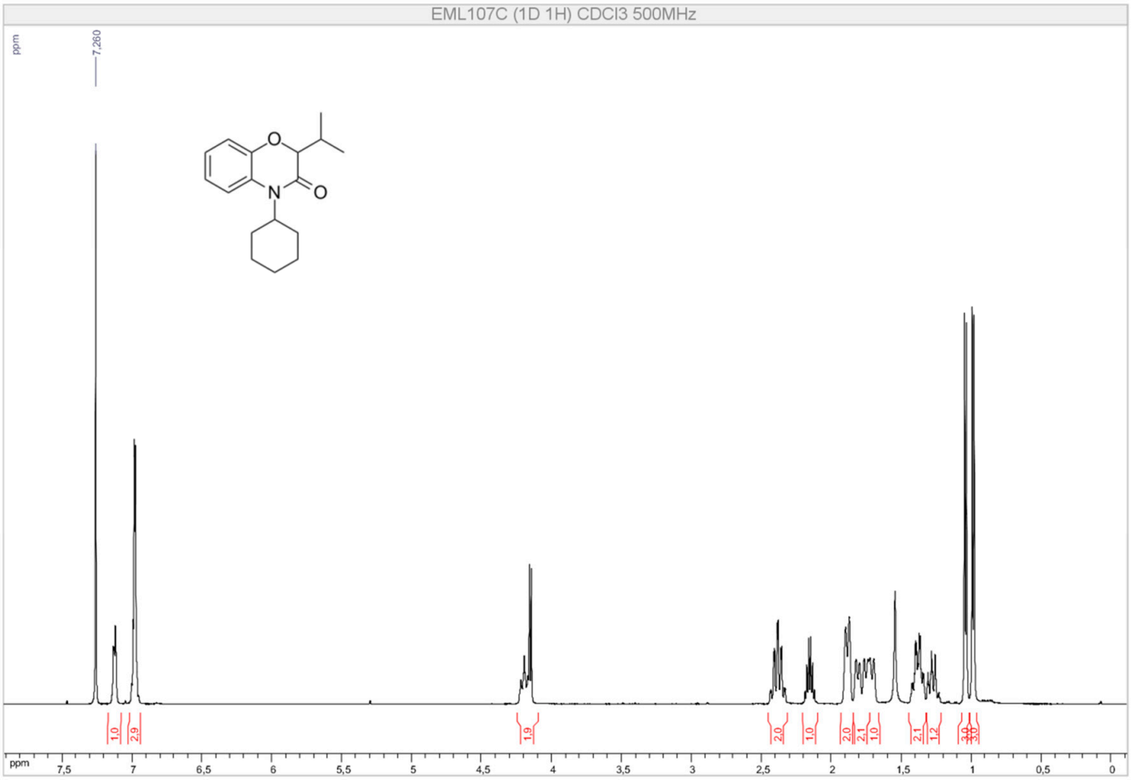

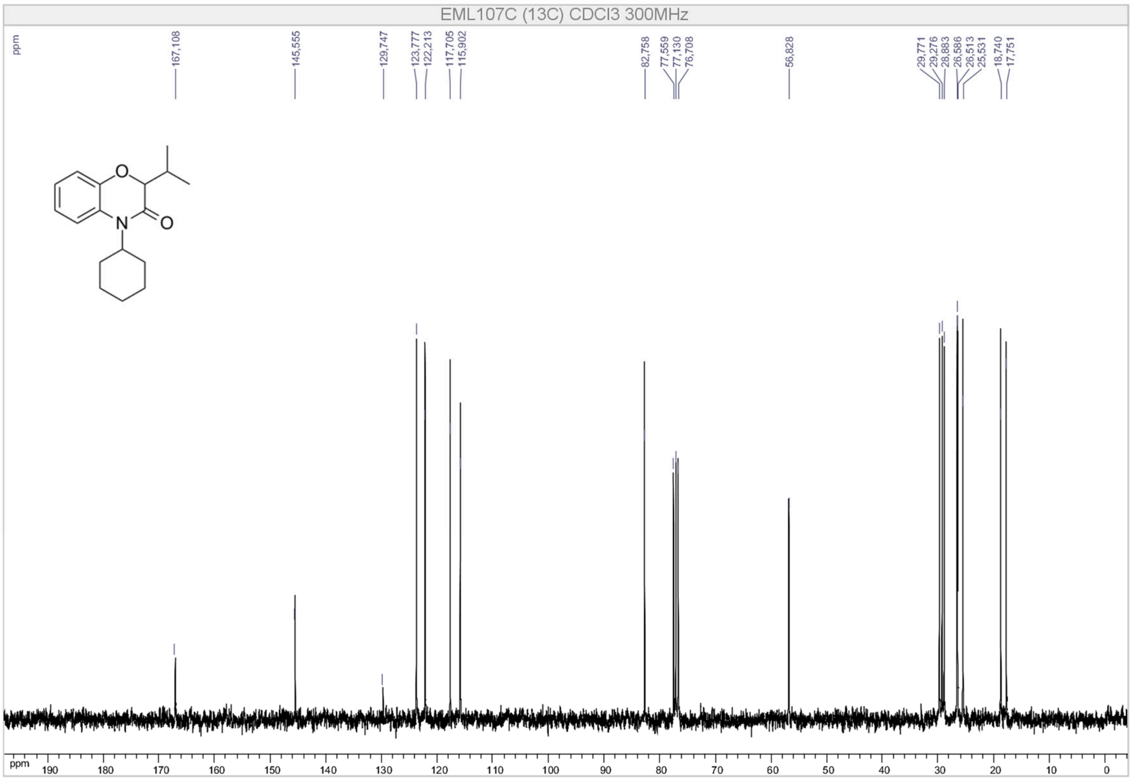

7-Chloro-4-cyclohexylspiro[benzo[b][1,4]oxazine-2,1′-cyclobutan]-3(4H)-one (**6b**)

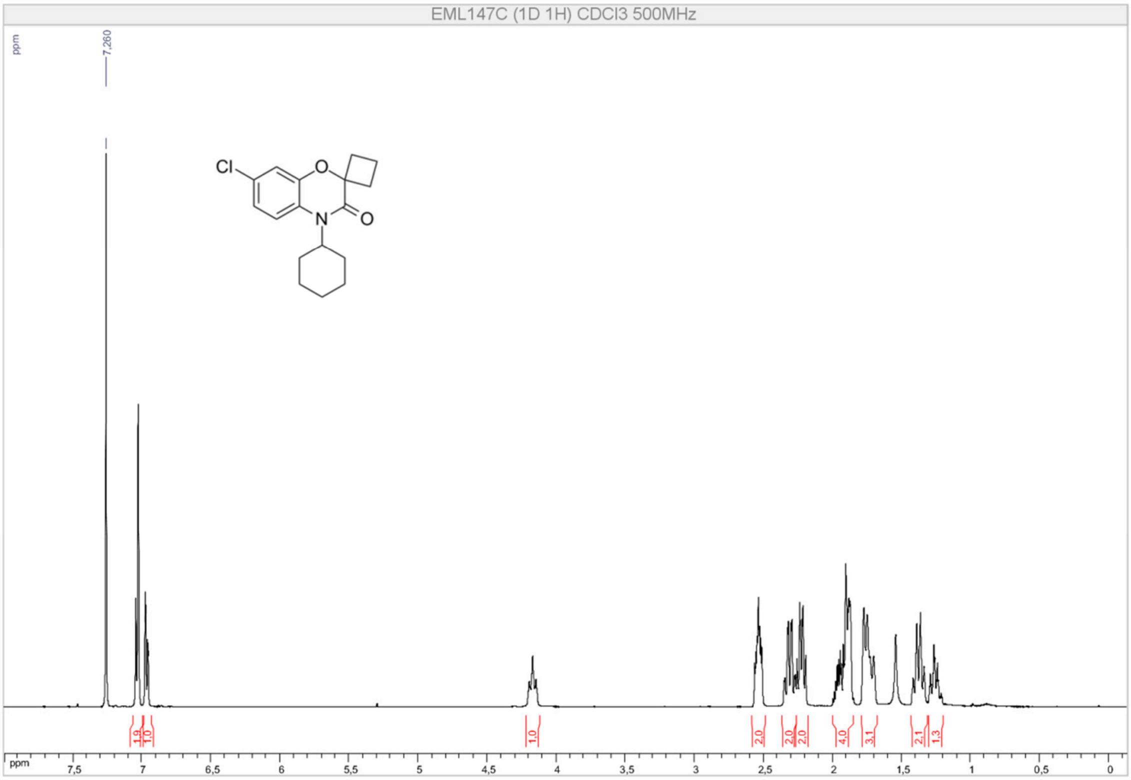

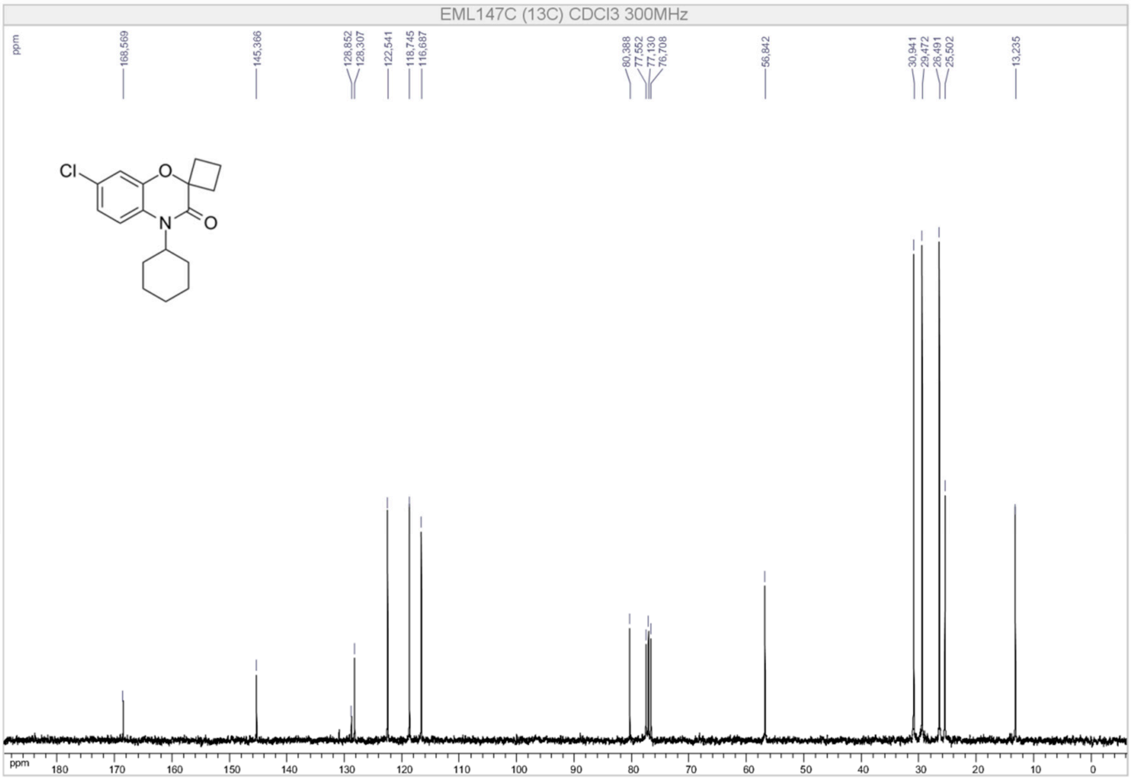

2-Isobutyl-4-phenethyl-7-(trifluoromethyl)-2H-benzo[b][1,4]oxazin-3(4H)-one (**6c**)

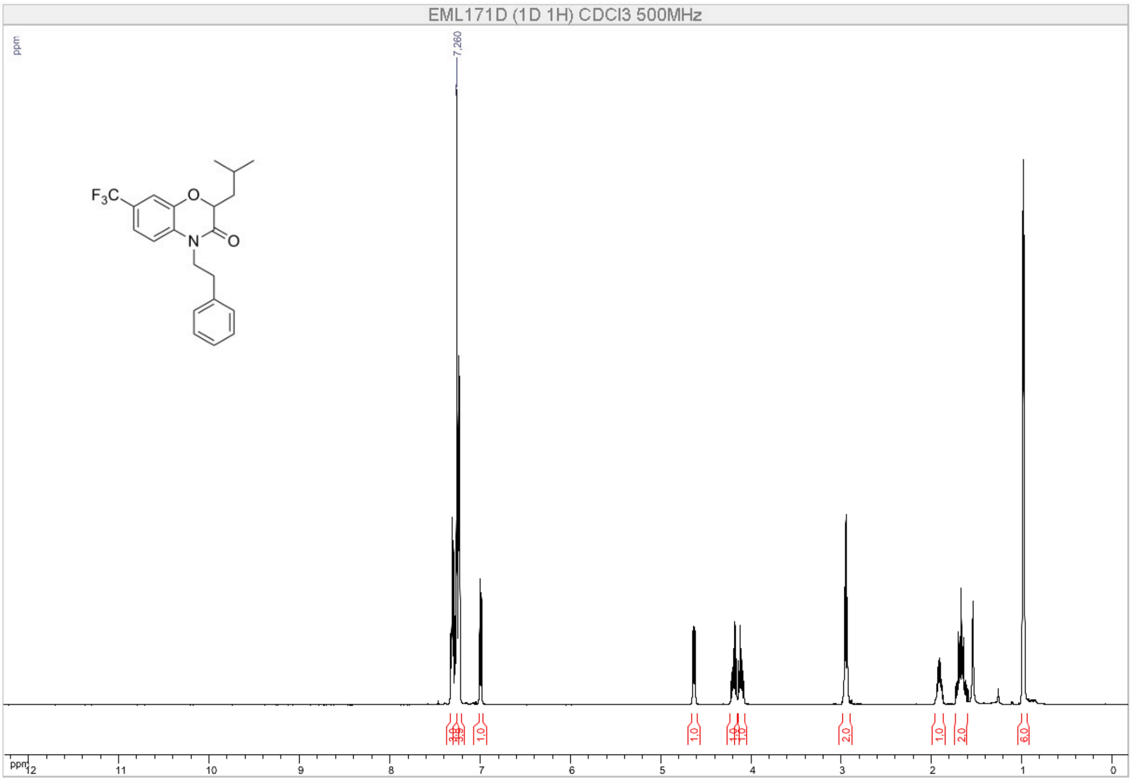

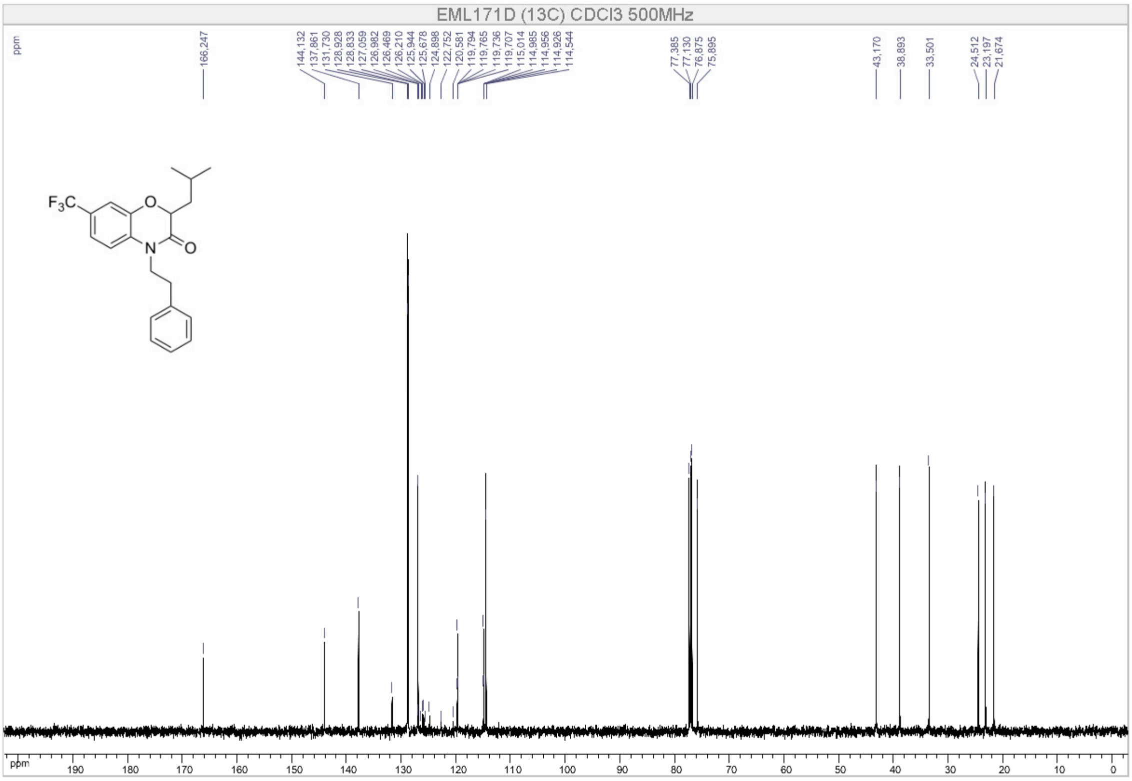

4-Cyclohexyl-2-methyl-2-(trifluoromethyl)-2H-benzo[b][1,4]oxazin-3(4H)-one (**6d**)

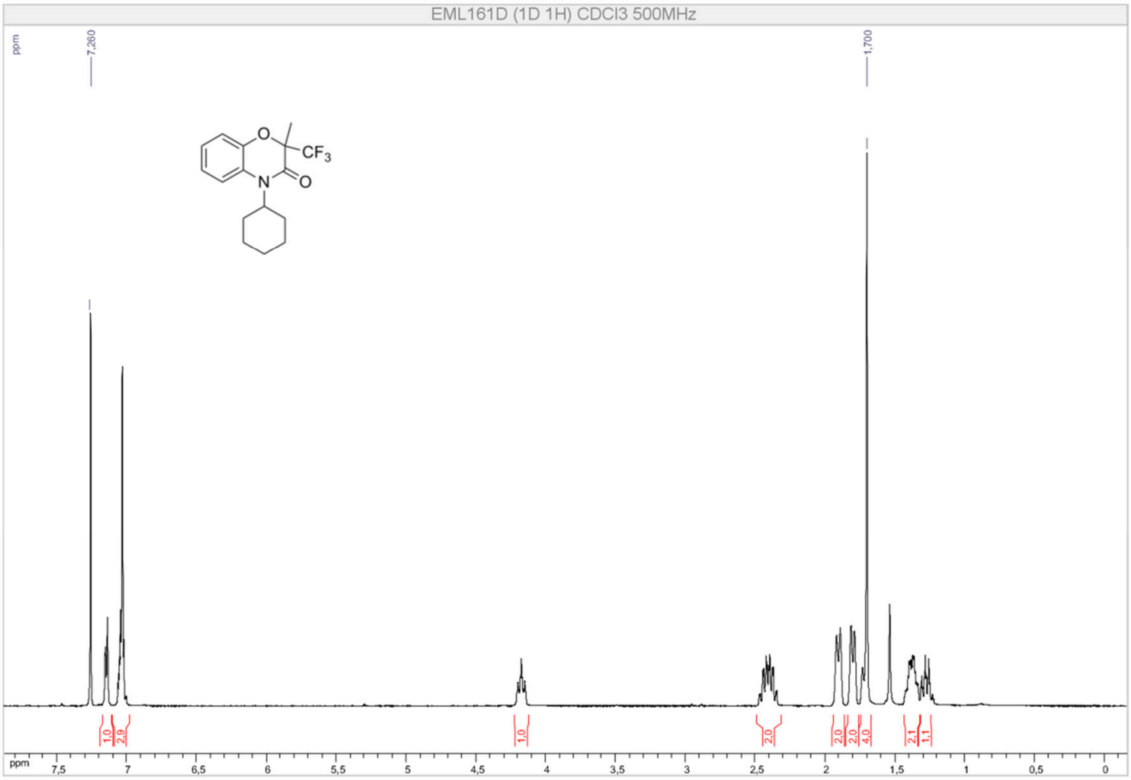

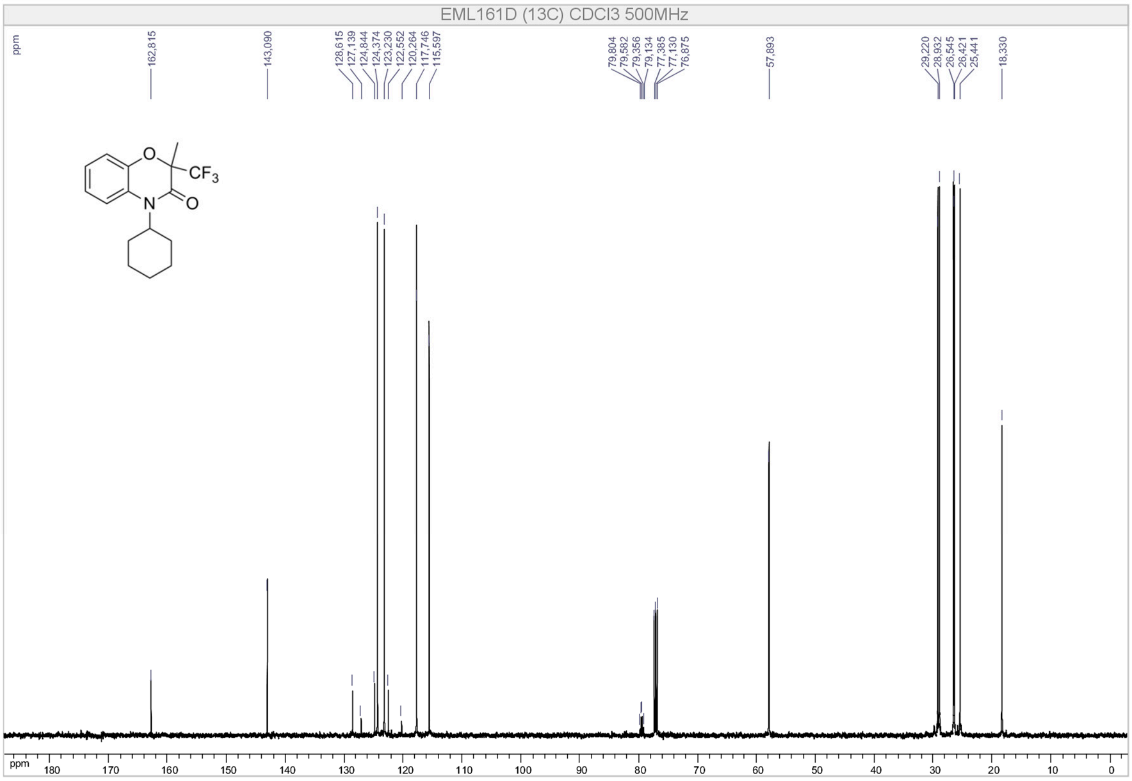

2-Ethyl-4-(4-methoxybenzyl)-3-oxo-3,4-dihydro-2H-benzo[b][1,4]oxazine-7-carbonitrile (**6e**)

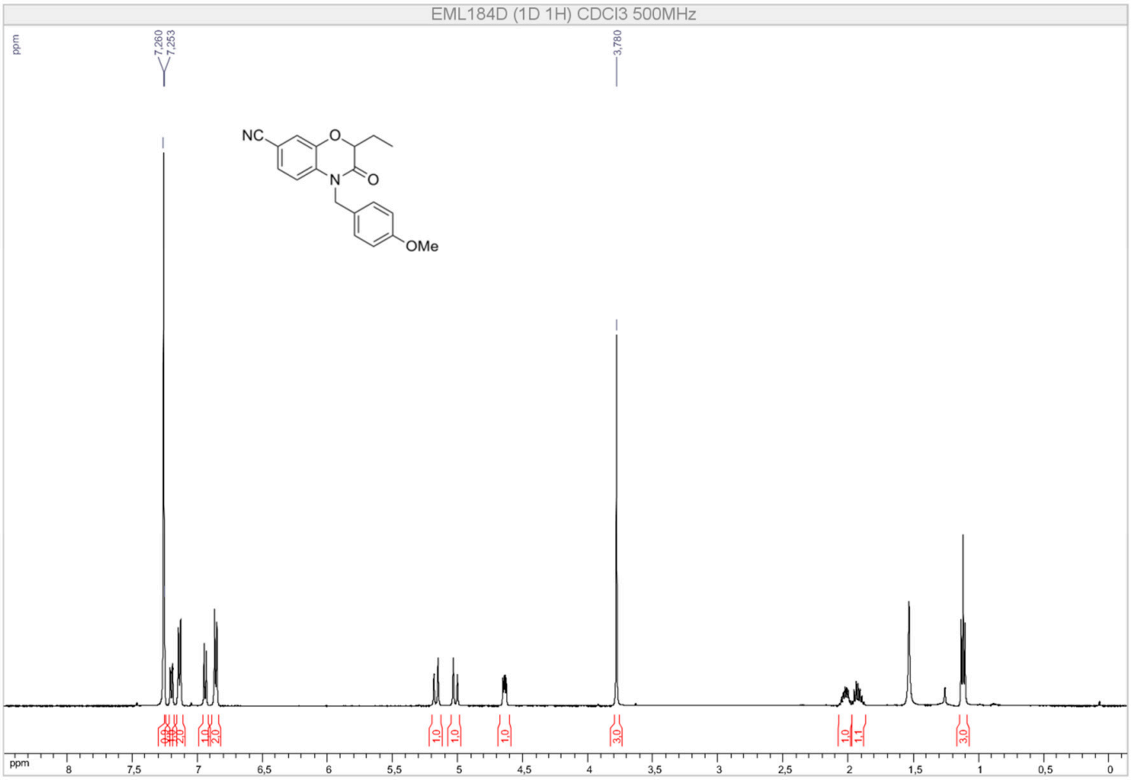

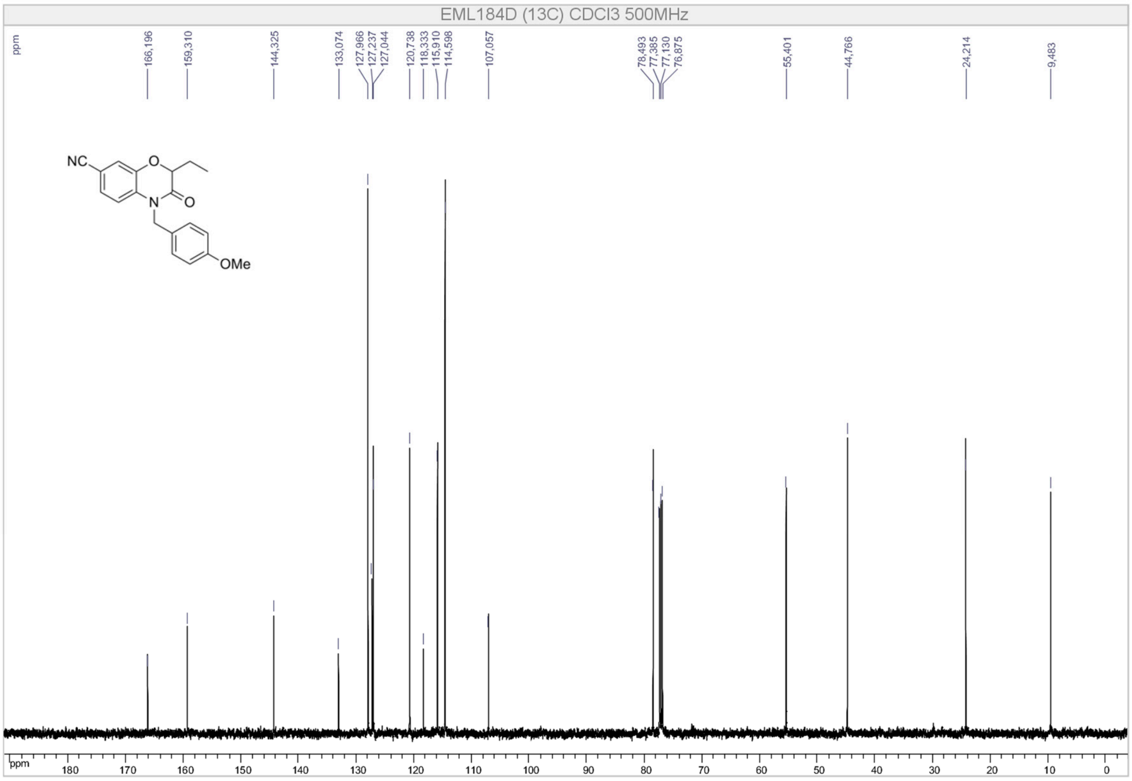

7-Bromo-4-(3,4-dimethoxyphenethyl)-2-isopropyl-2H-benzo[b][1,4]oxazin-3(4H)-one (**6f**)

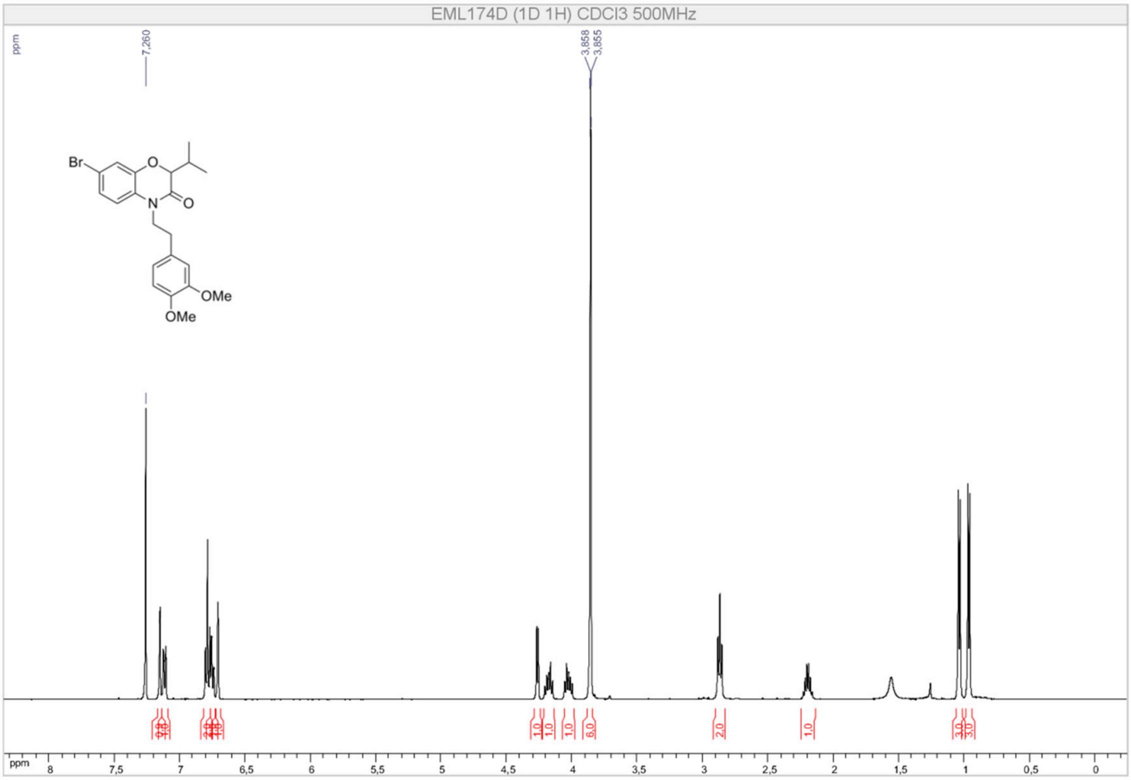

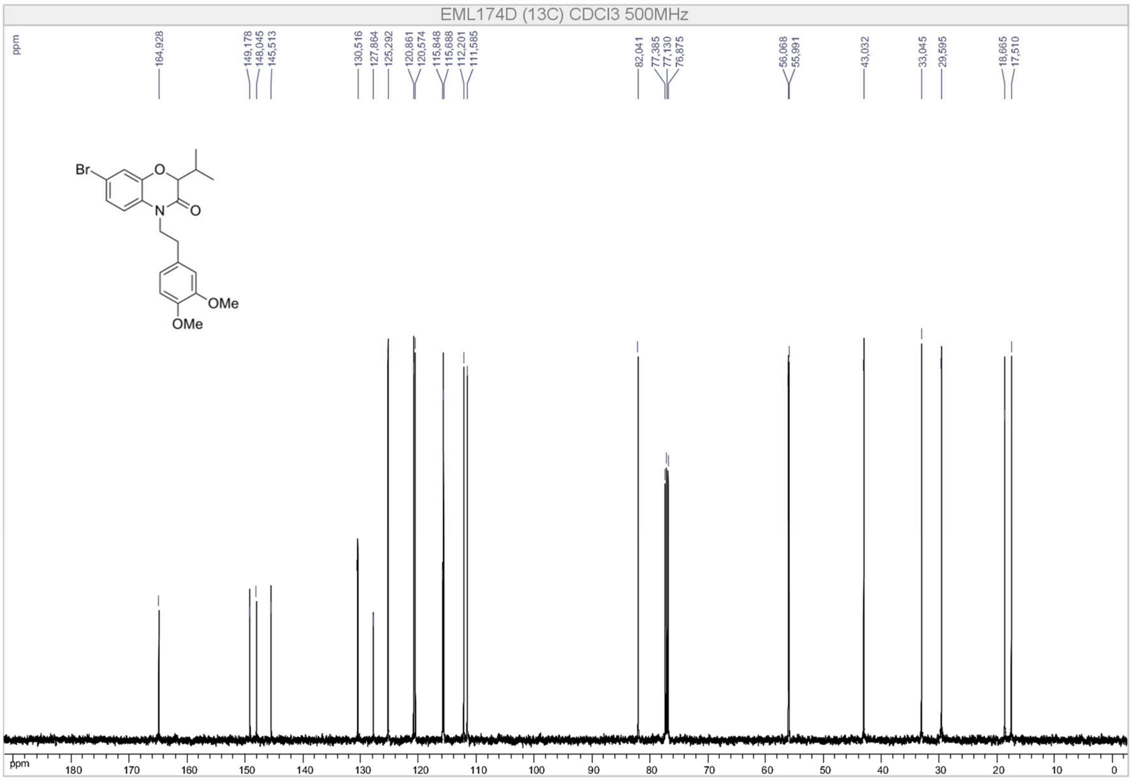

4-(4-Methoxybenzyl)spiro[benzo[b][1,4]oxazine-2,1′-cyclobutan]-3(4H)-one (**6g**)

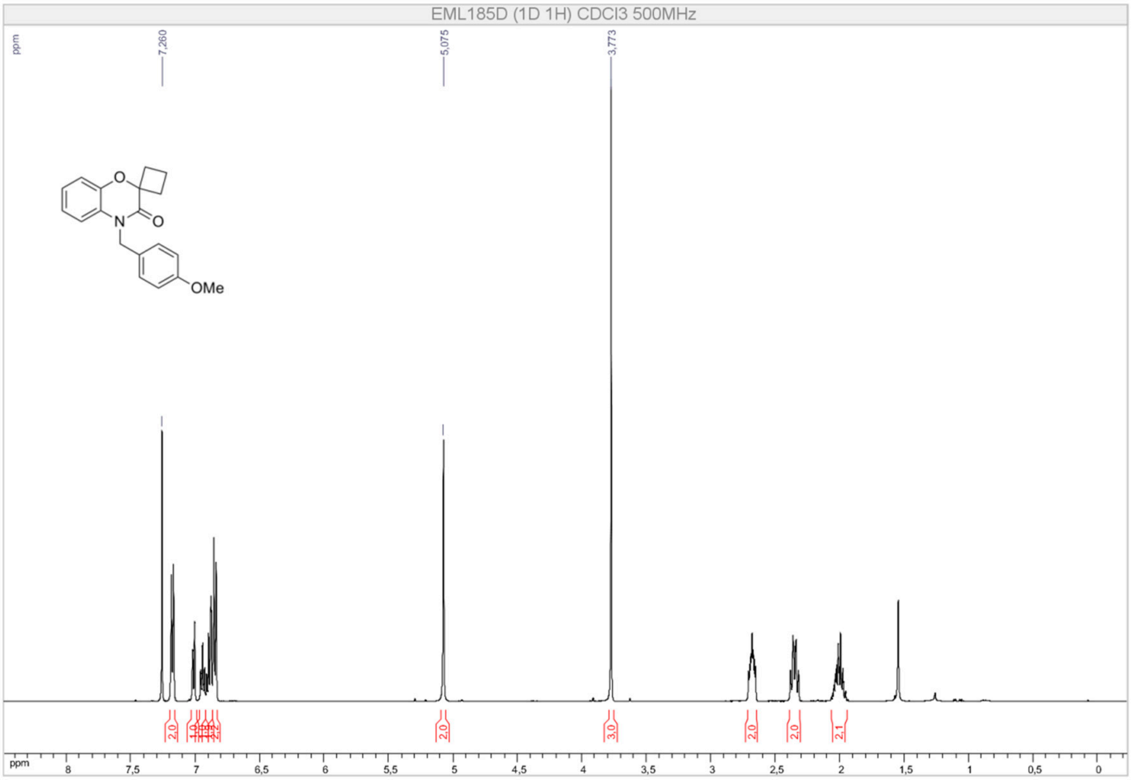

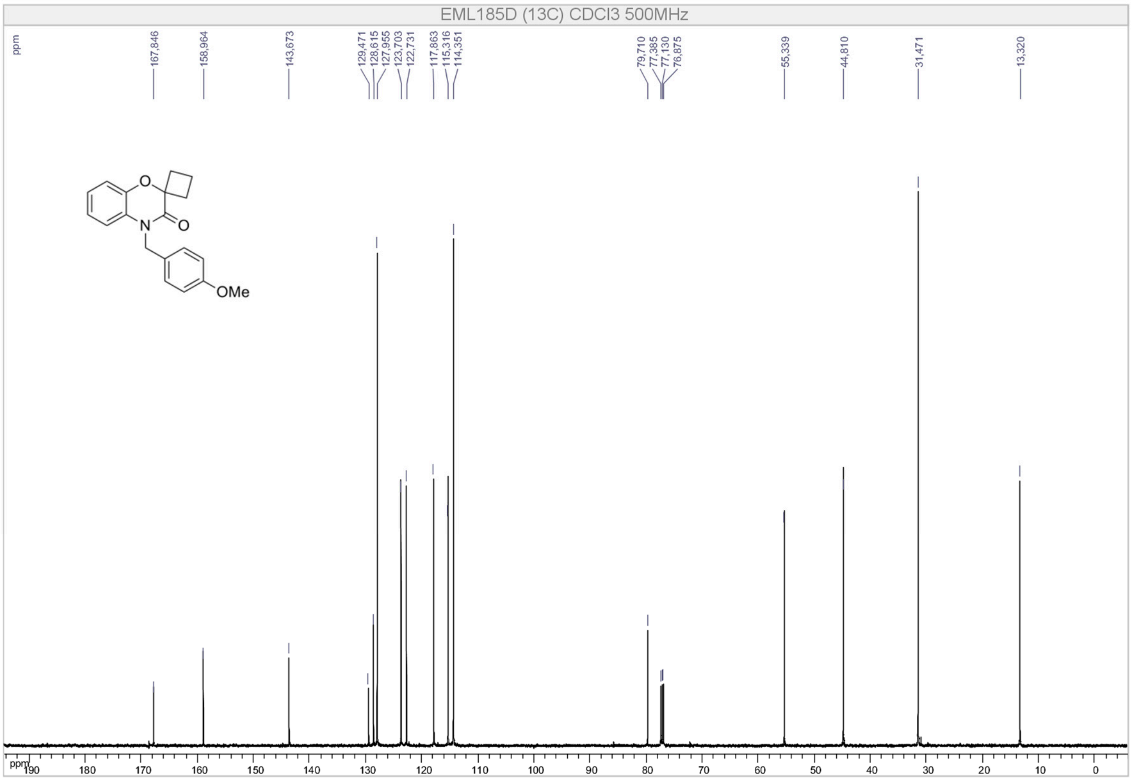

4-(Tert-butyl)-7-chloro-2-isopropyl-2H-benzo[b][1,4]oxazin-3(4H)-one (**6h**)

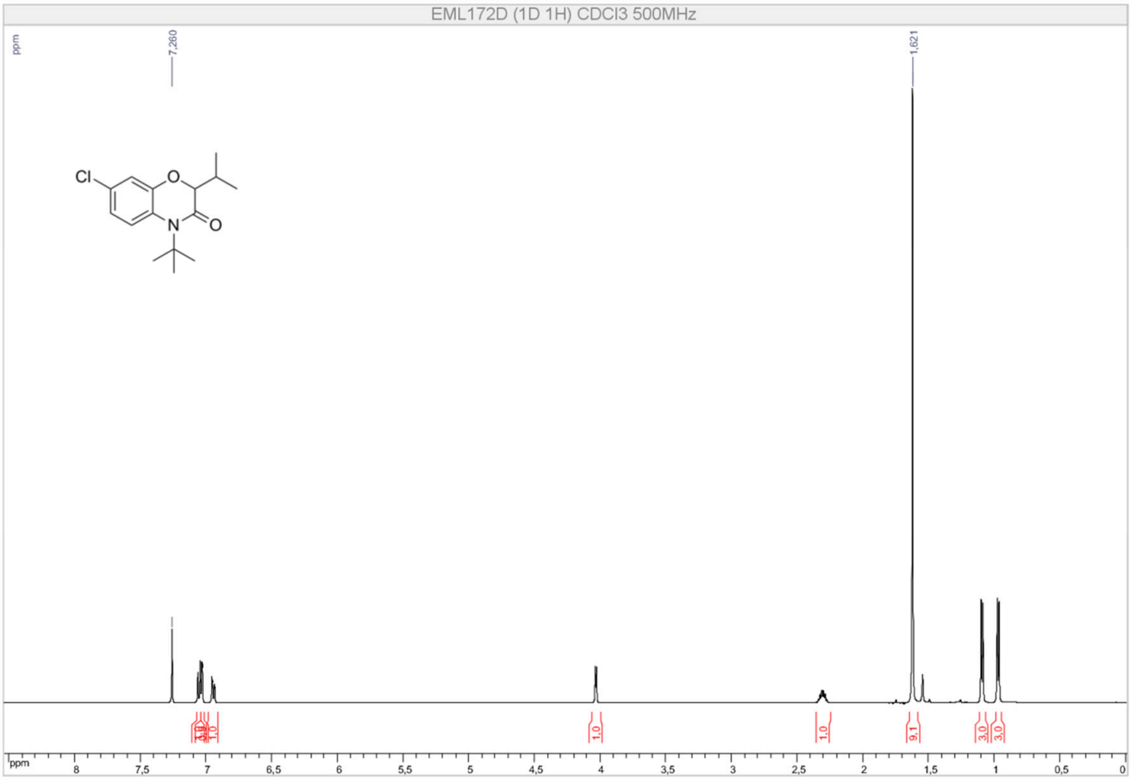

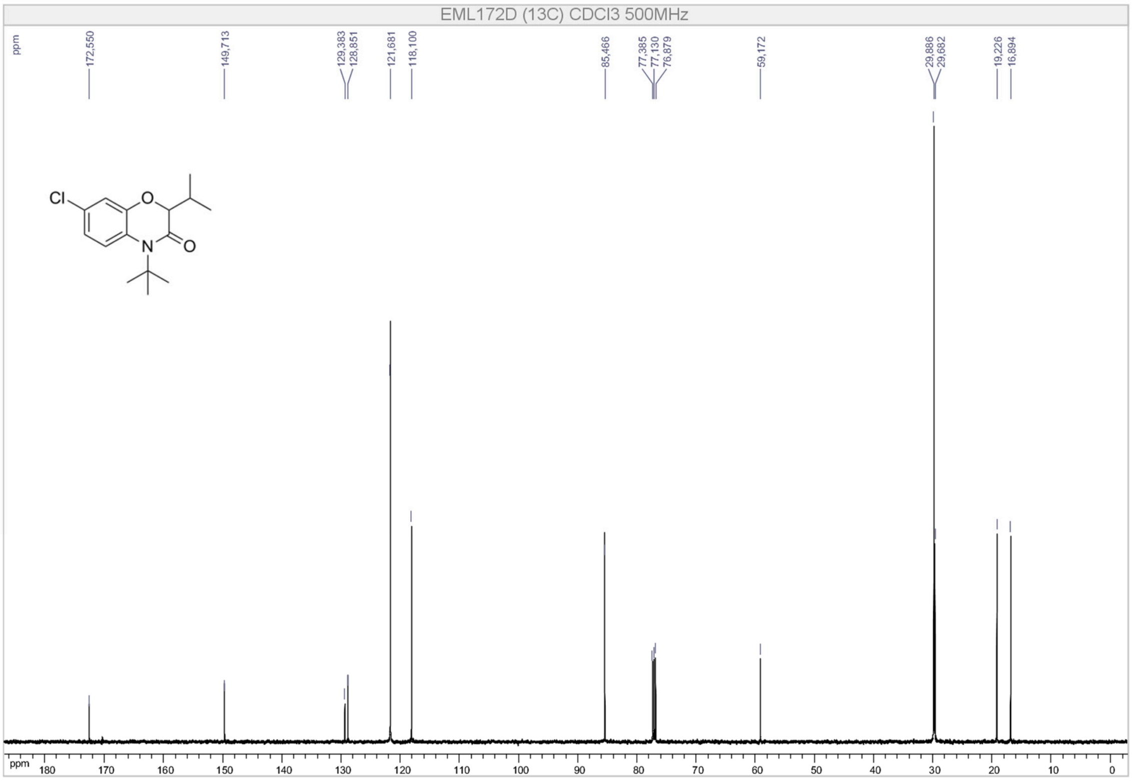

4-(3,4-Dimethoxyphenethyl)-2-(2,6-dimethylhept-5-en-1-yl)-7-(trifluoromethyl)-2H-benzo[b][1,4]oxazin-3(4H)-one (**6i**)

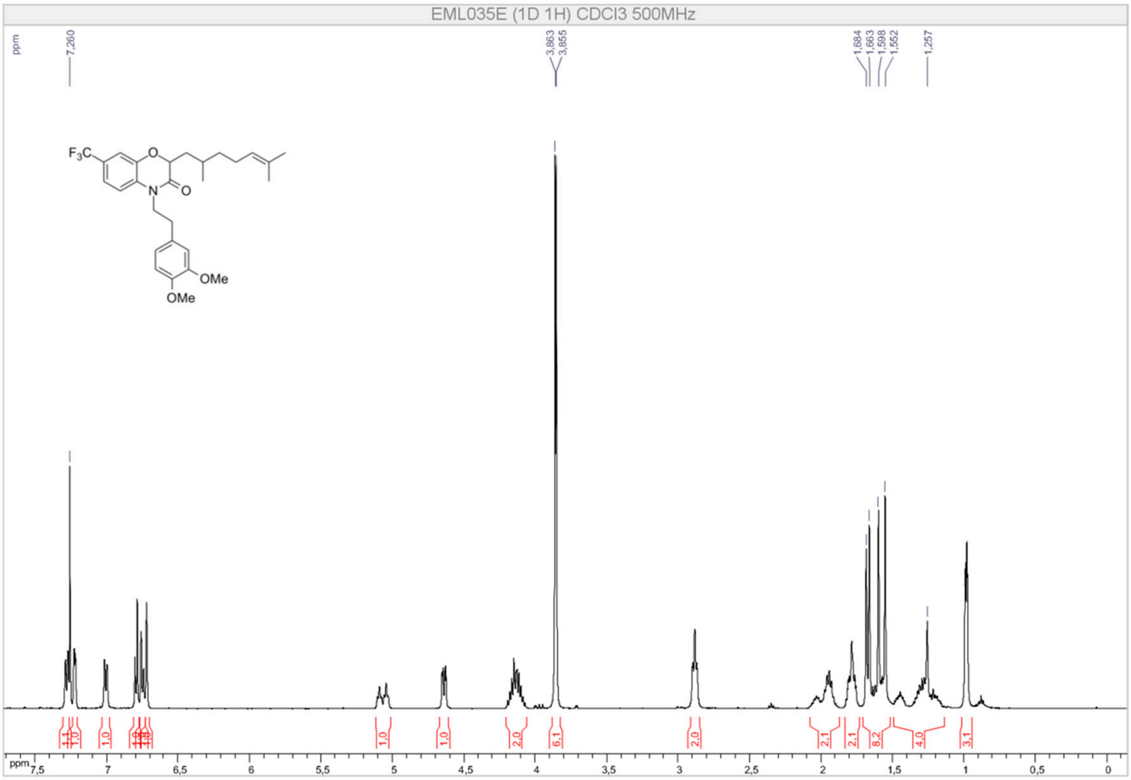

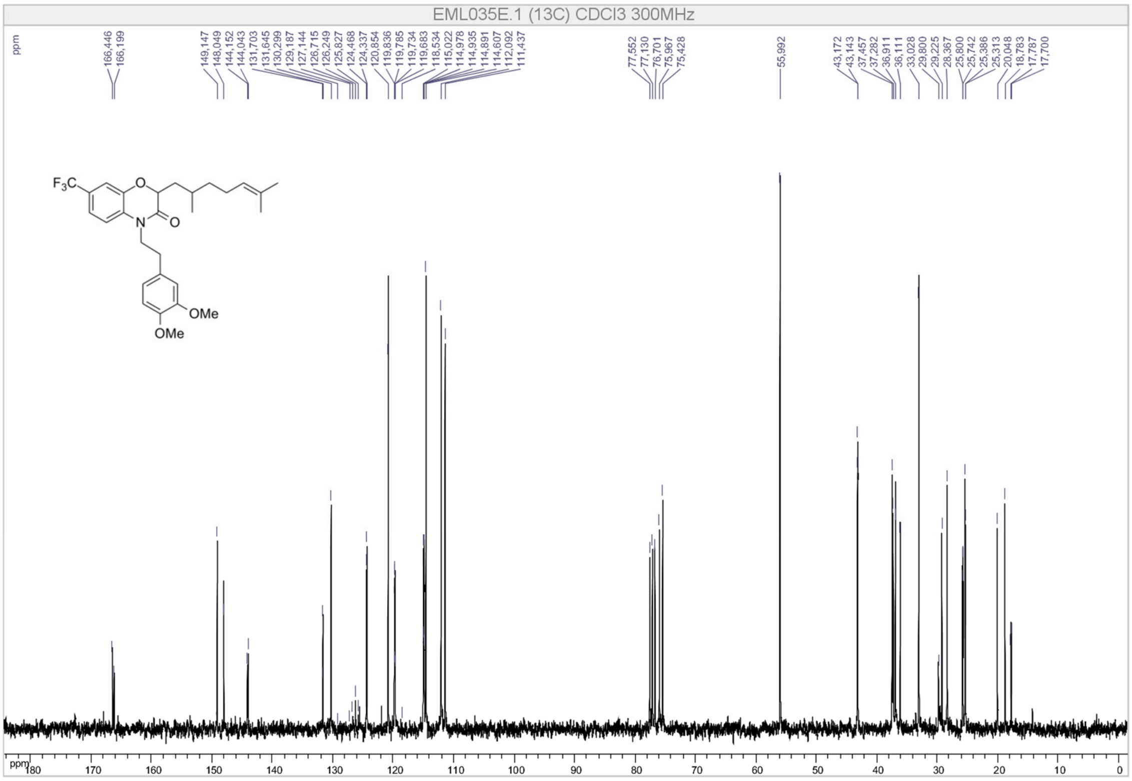

4-(4-Chlorobenzyl)-2-isobutyl-7-methyl-2H-benzo[b][1,4]oxazin-3(4H)-one (**6j**)

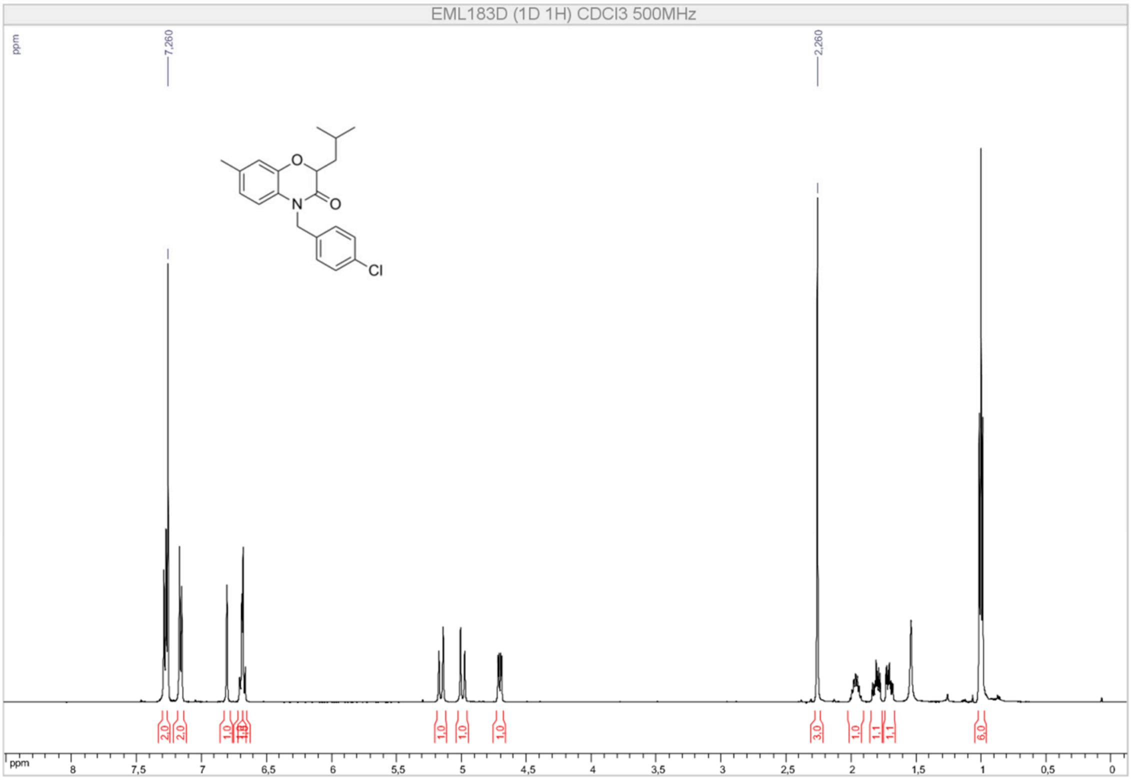

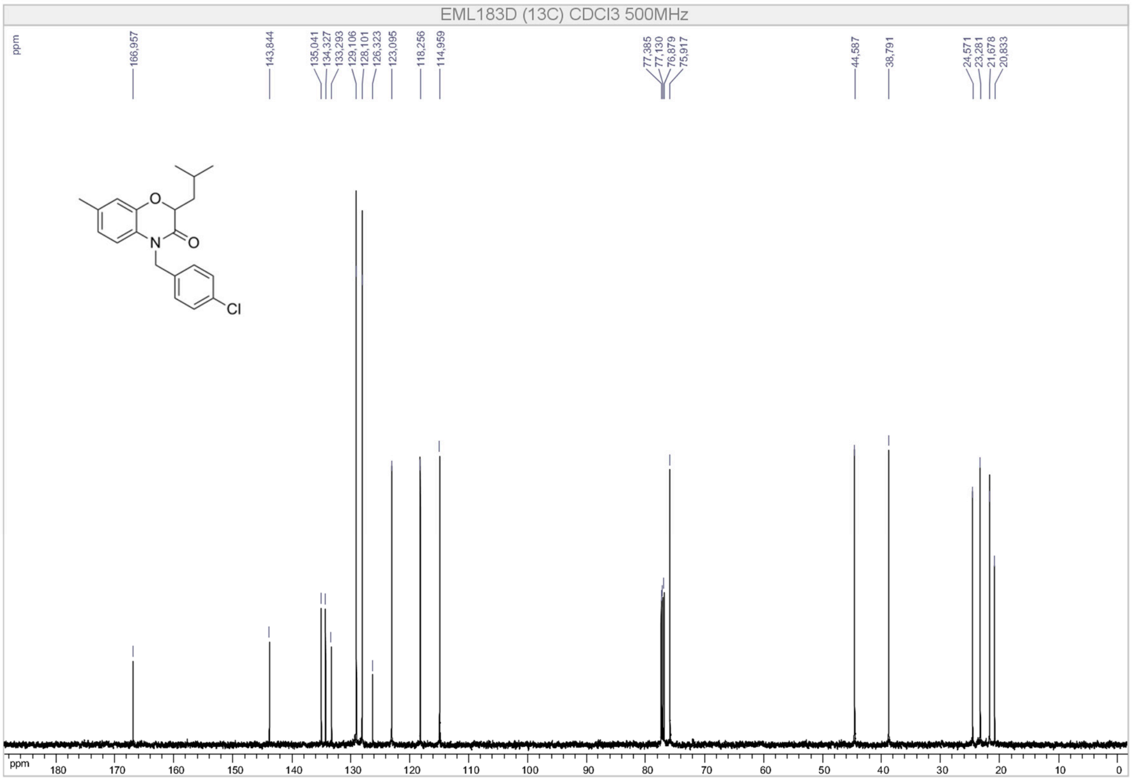

2-(Tert-butyl)-7-methoxy-4-phenethyl-2H-benzo[b][1,4]oxazin-3(4H)-one (**6k**)

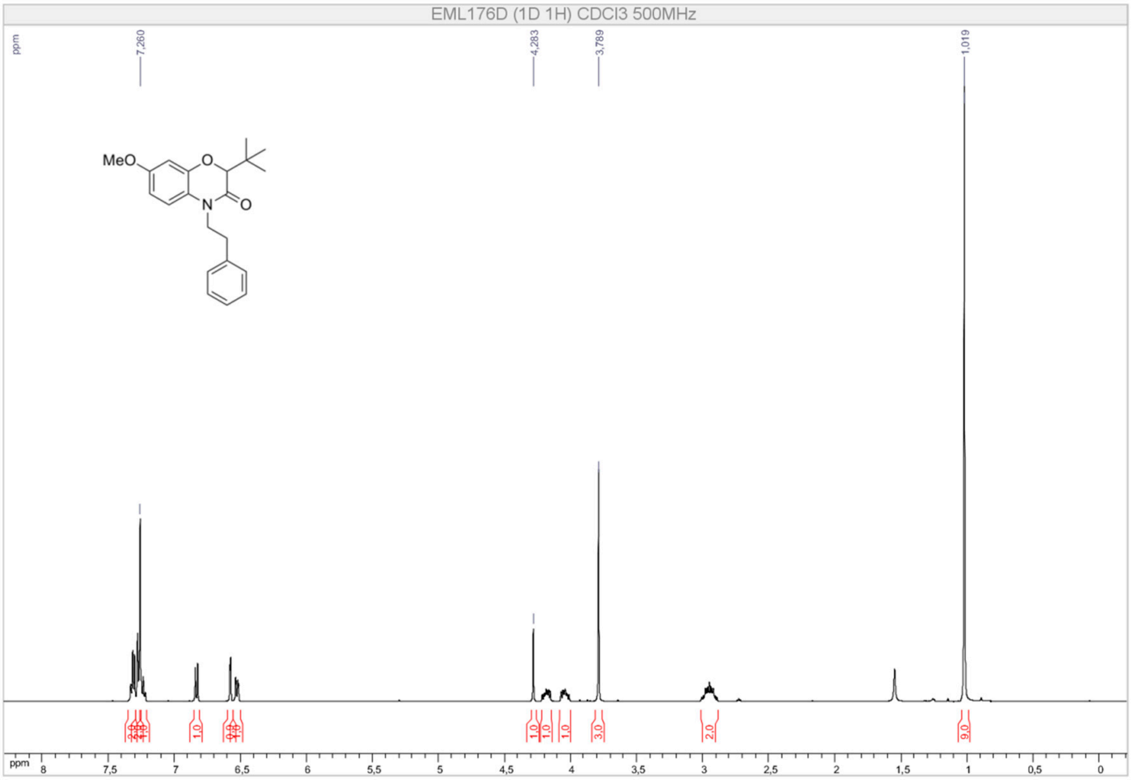

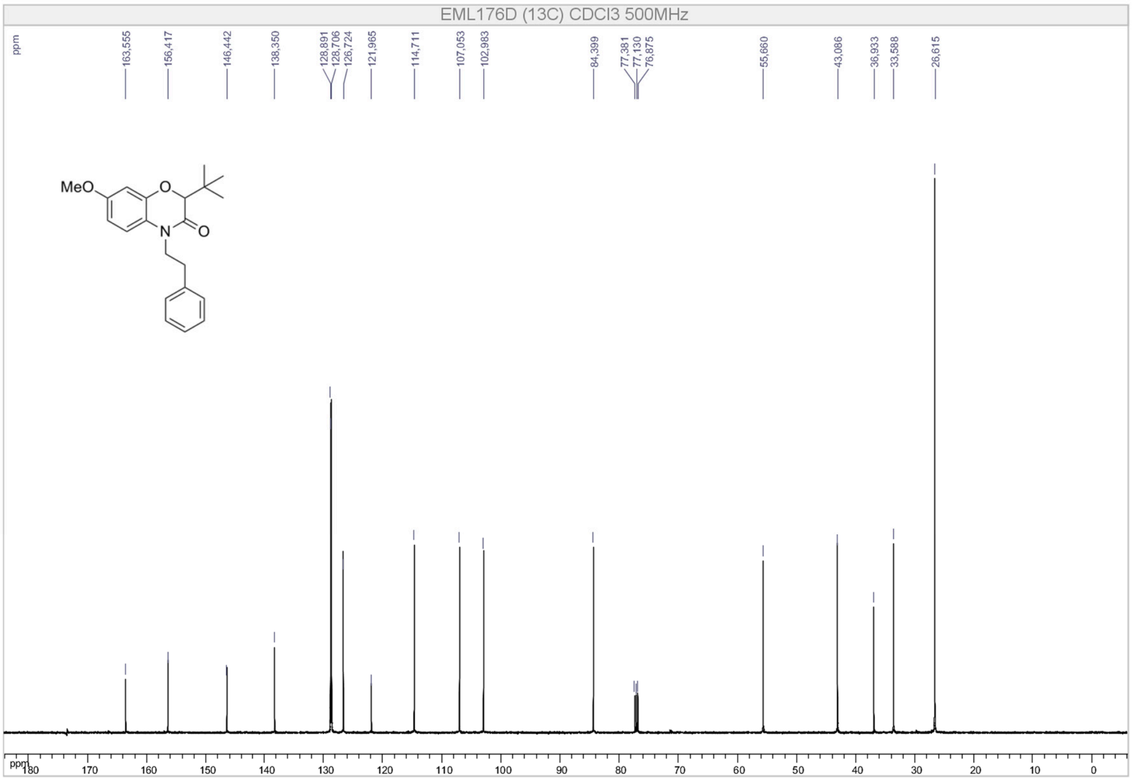

4-Cyclohexyl-2-ethyl-2H-benzo[b][1,4]oxazin-3(4H)-one (**6l**)

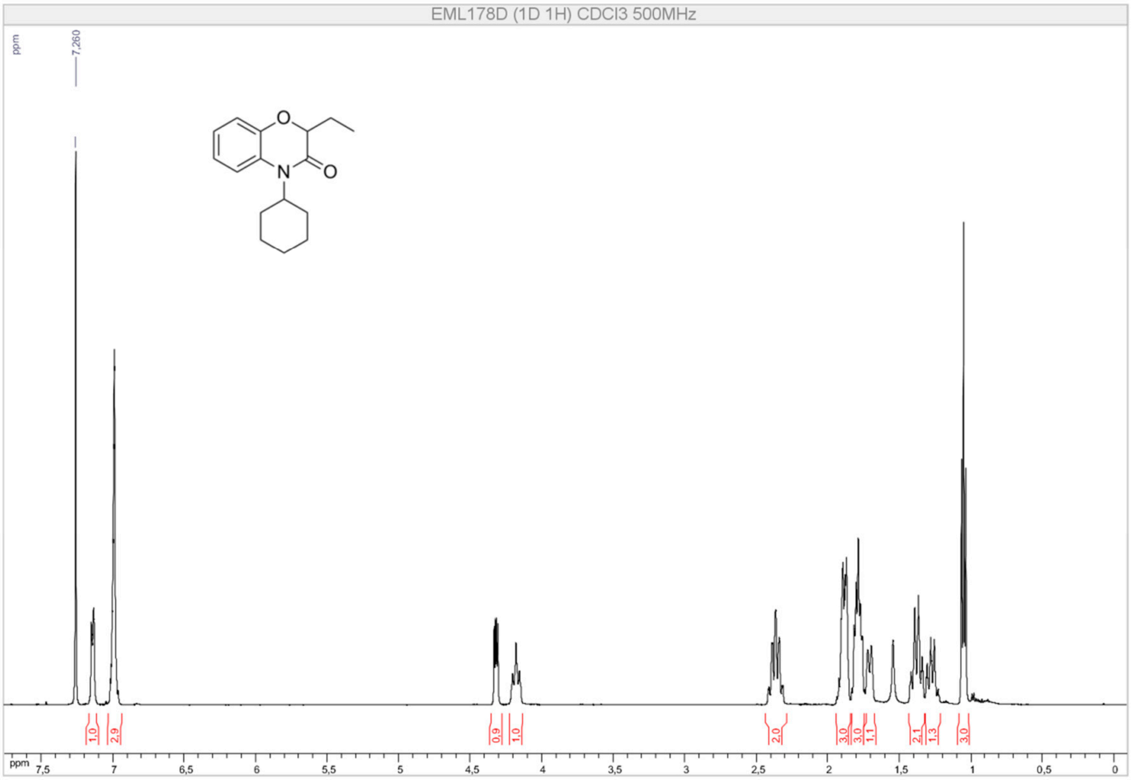

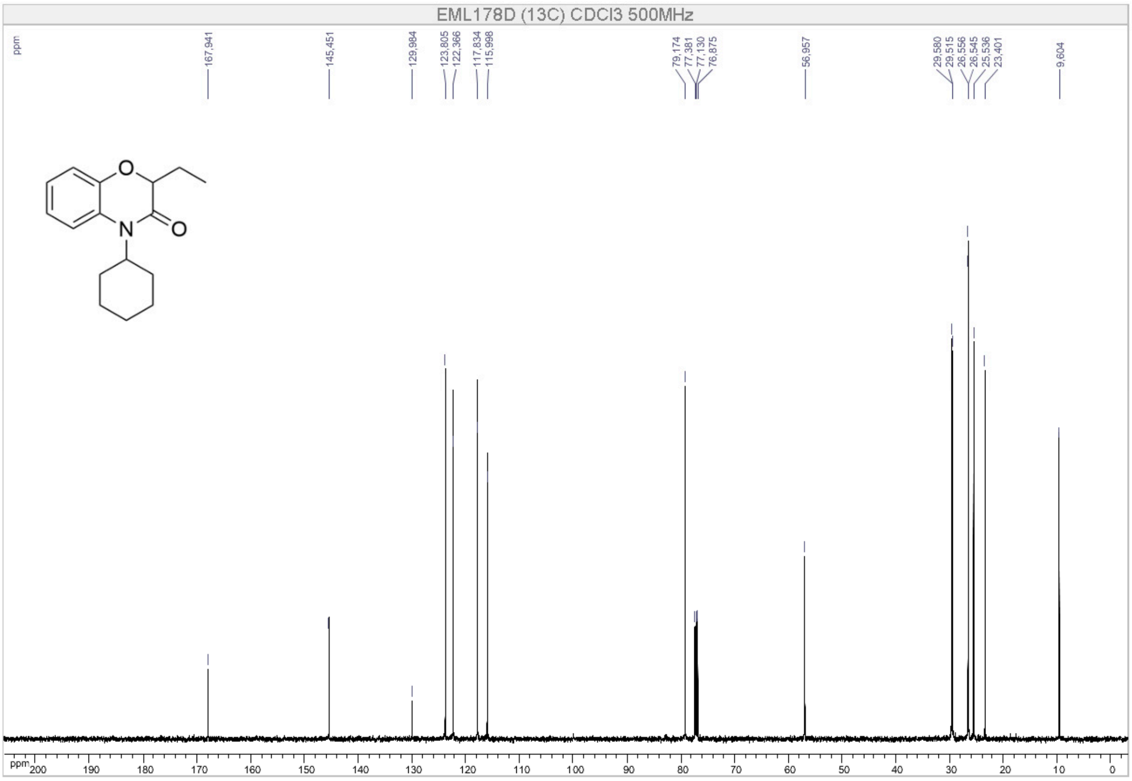

7-Bromo-2-(tert-butyl)-4-phenethyl-2H-benzo[b][1,4]oxazin-3(4H)-one (**6m**)

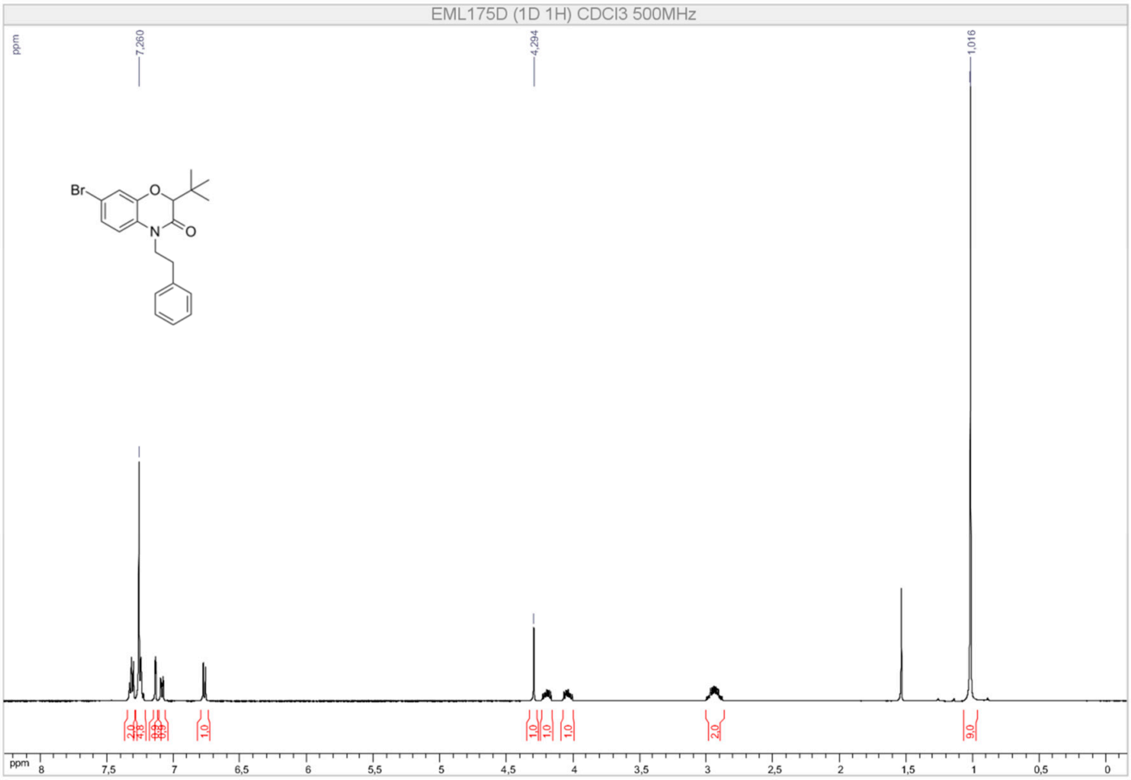

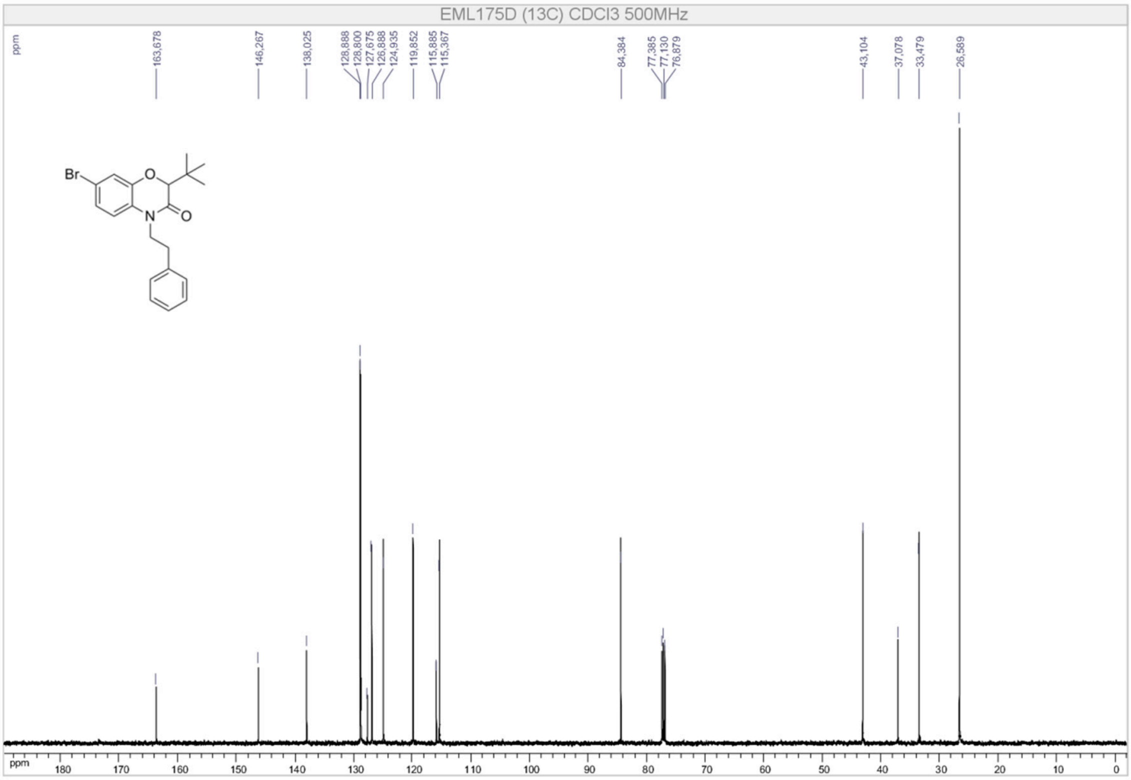

4-(4-Methoxybenzyl)-7-methyl-2-(2-methylpent-4-en-2-yl)-2H-benzo[b][1,4]oxazin-3(4H)-one (**6n**)

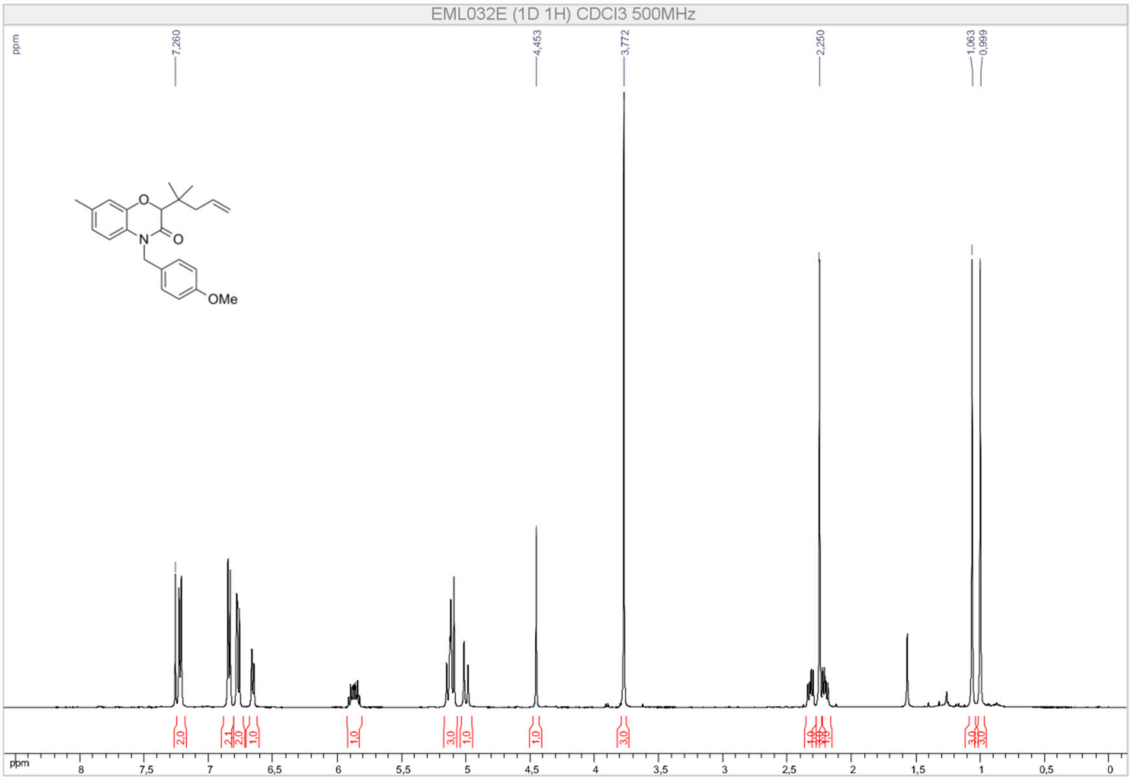

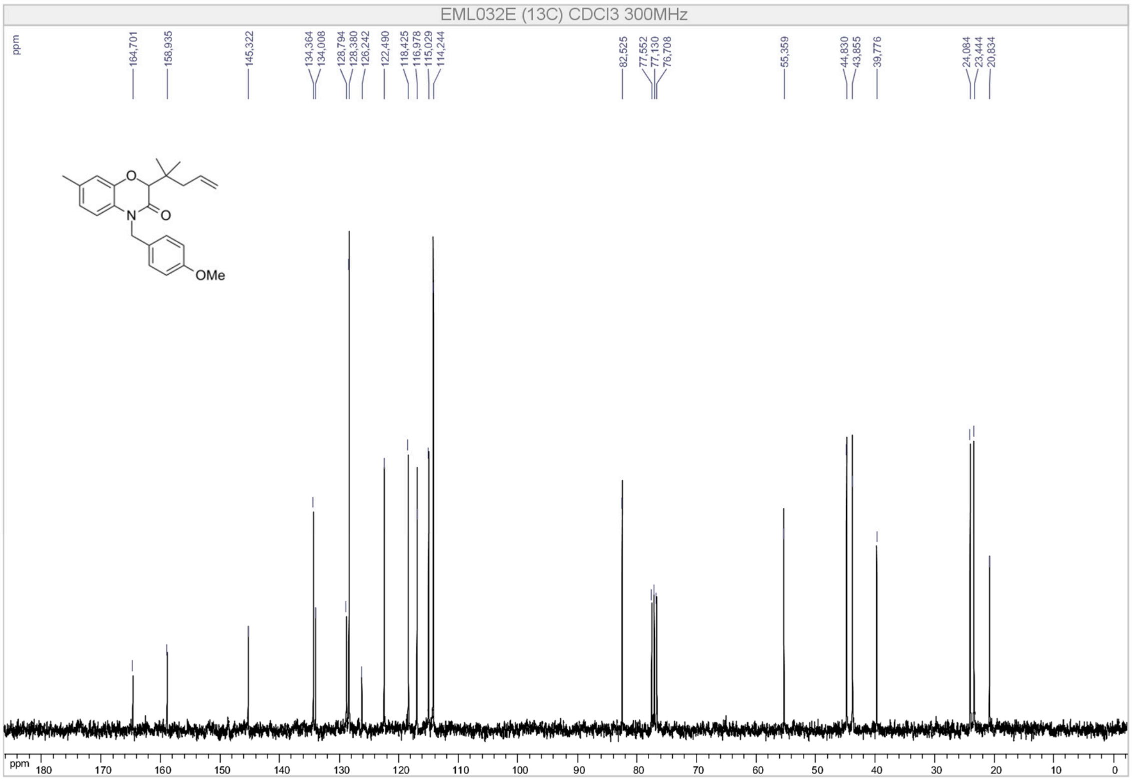

2-(Tert-butyl)-4-cyclohexyl-2H-benzo[b][1,4]oxazin-3(4H)-one (**6o**)

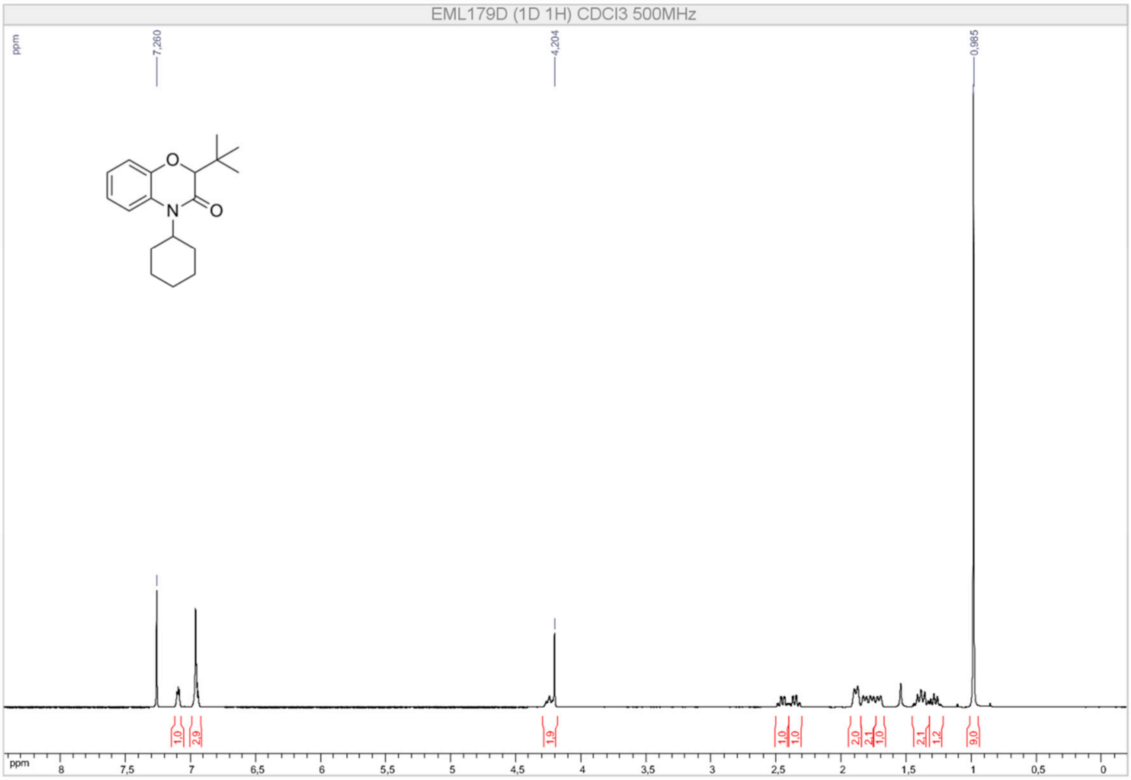

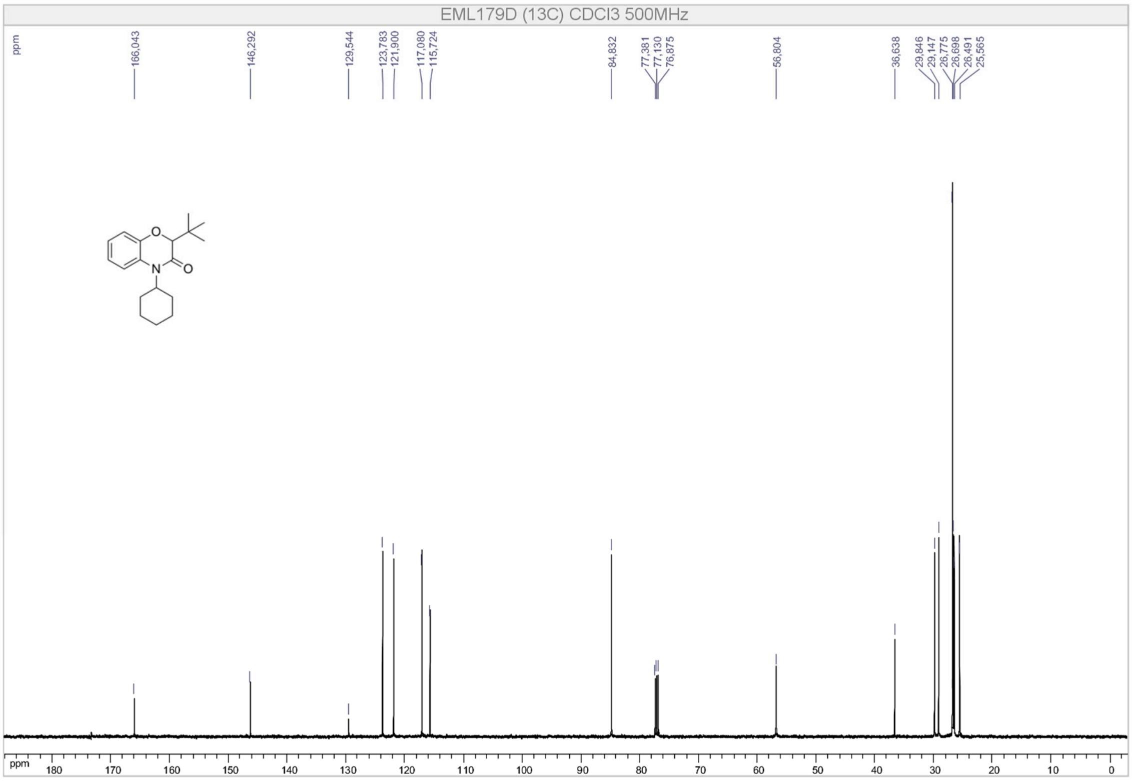

7-Bromo-4-(3,4-dimethoxyphenethyl)spiro[benzo[b][1,4]oxazine-2,1′-cyclobutan]-3(4H)-one (**6p**)

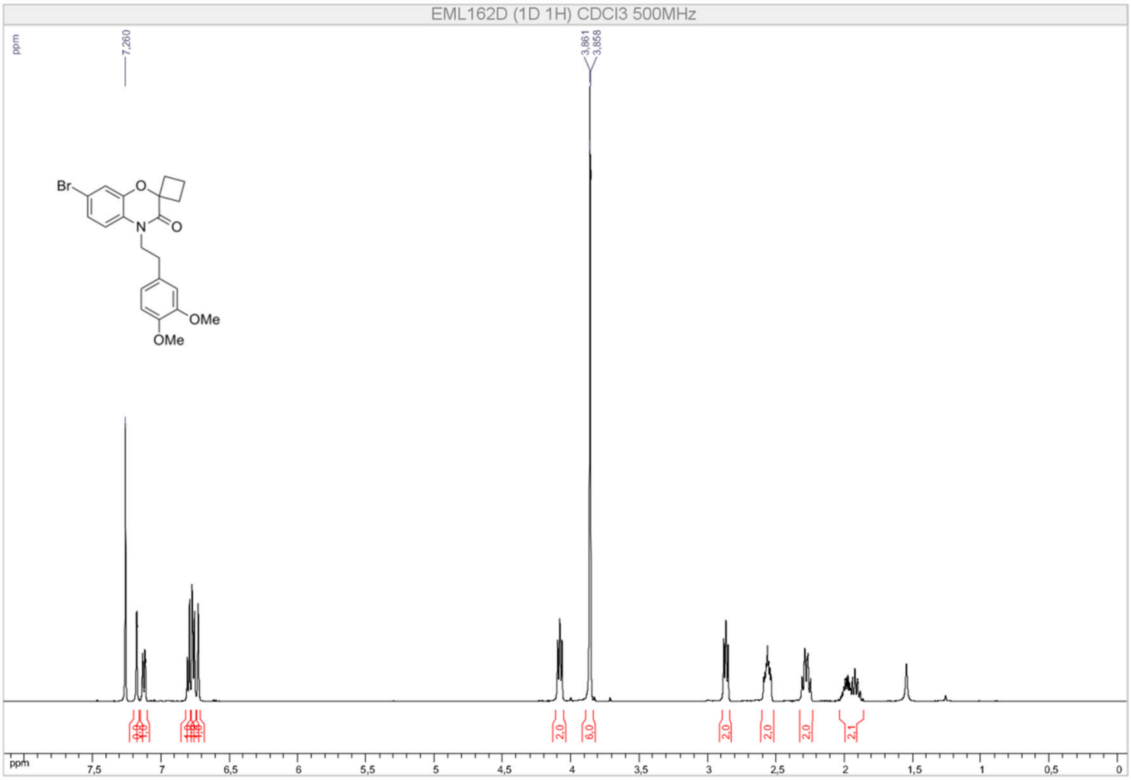

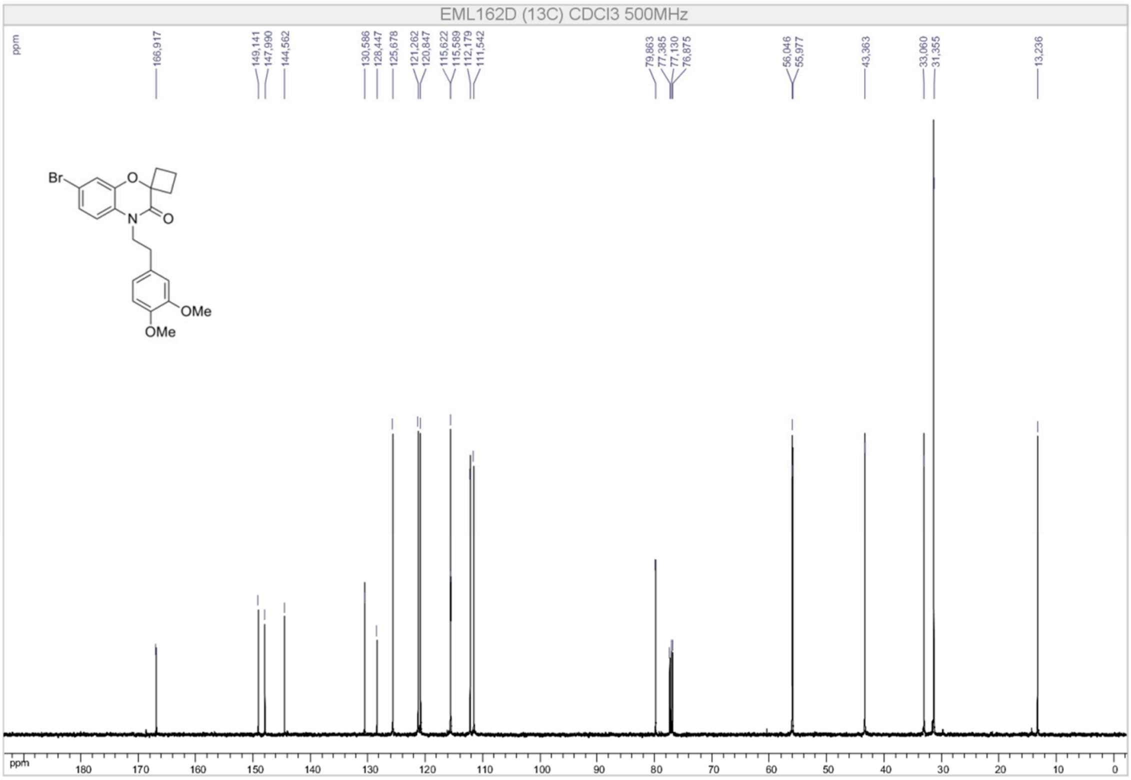

4-(3,4-Dimethoxyphenethyl)-2-isobutyl-3-oxo-3,4-dihydro-2H-benzo[b][1,4]oxazine-7-carbonitrile (**6q**)

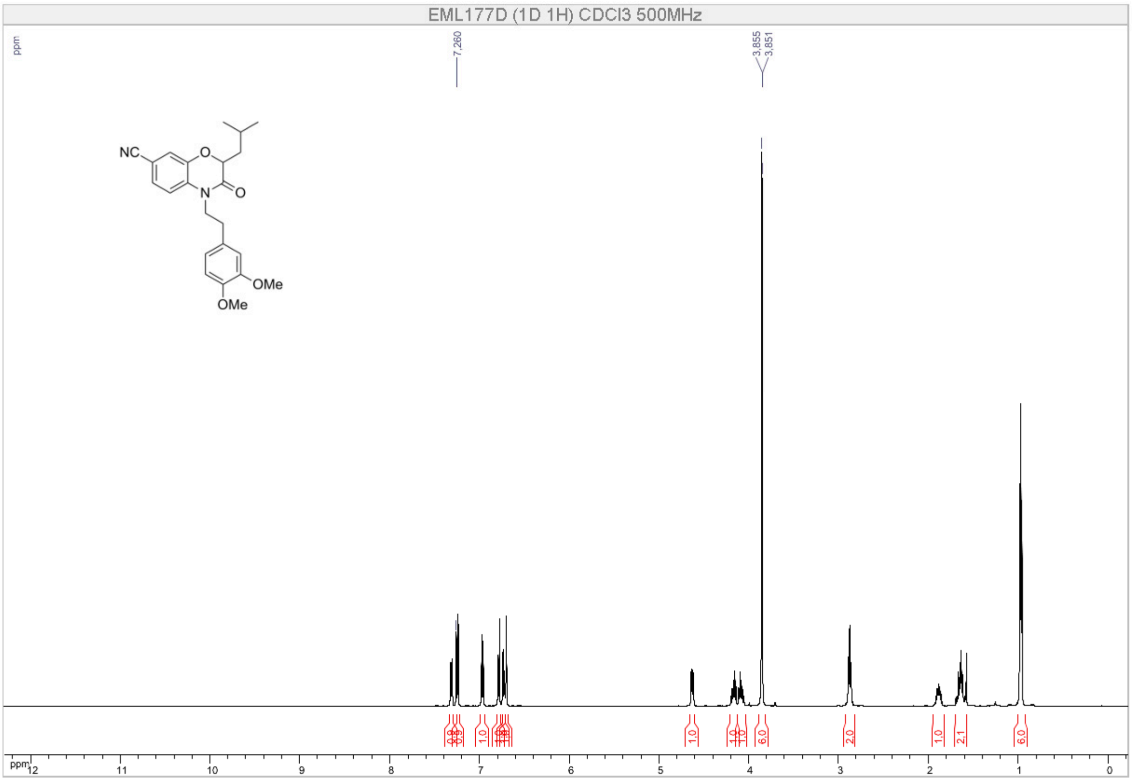

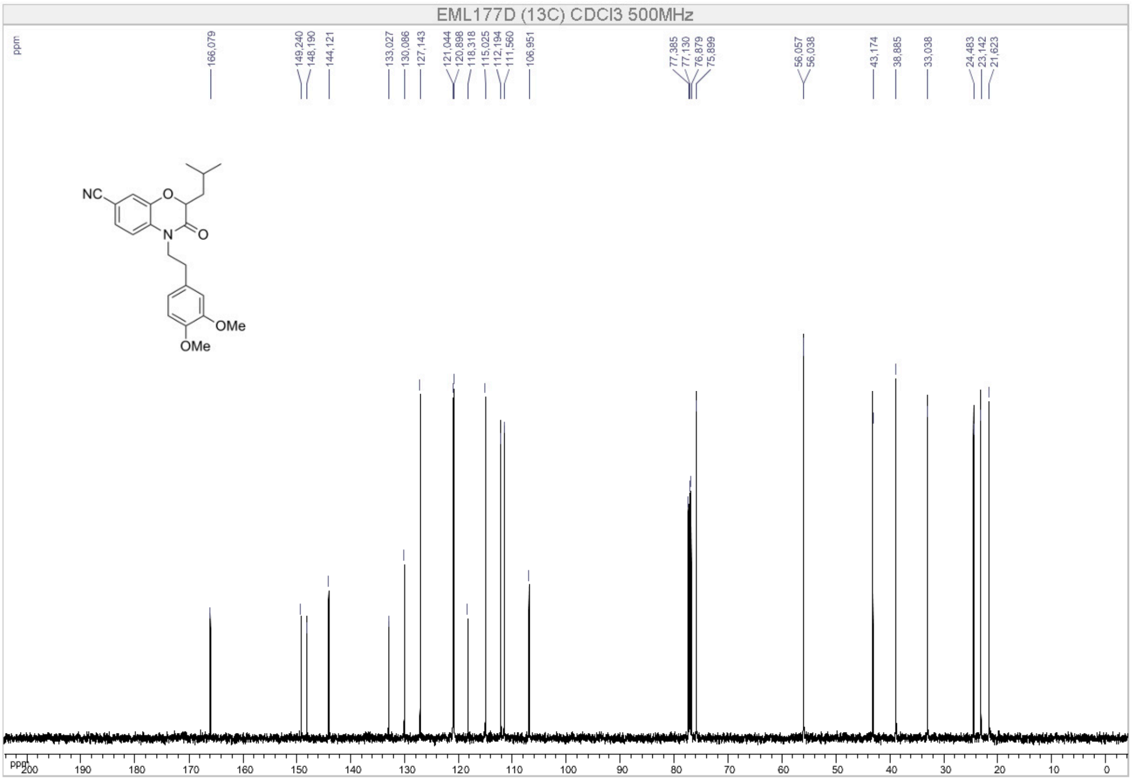

4-Cyclohexyl-2-phenethyl-2H-benzo[b][1,4]oxazin-3(4H)-one (**6r**)

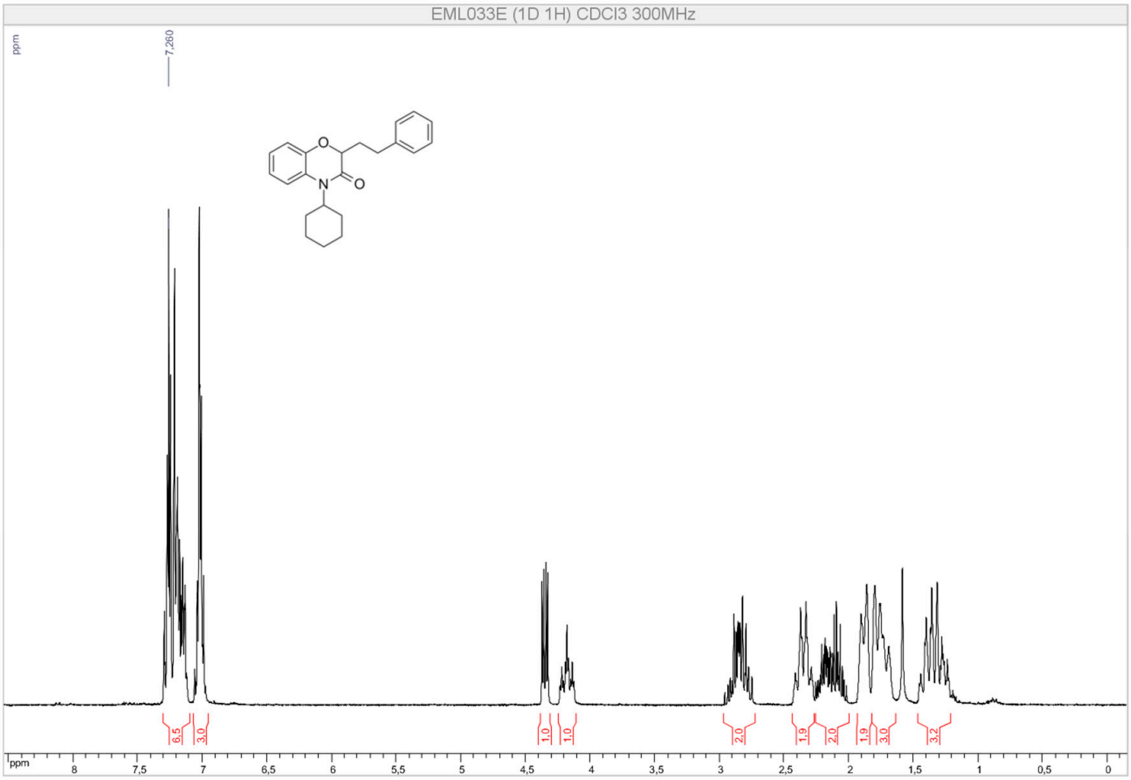

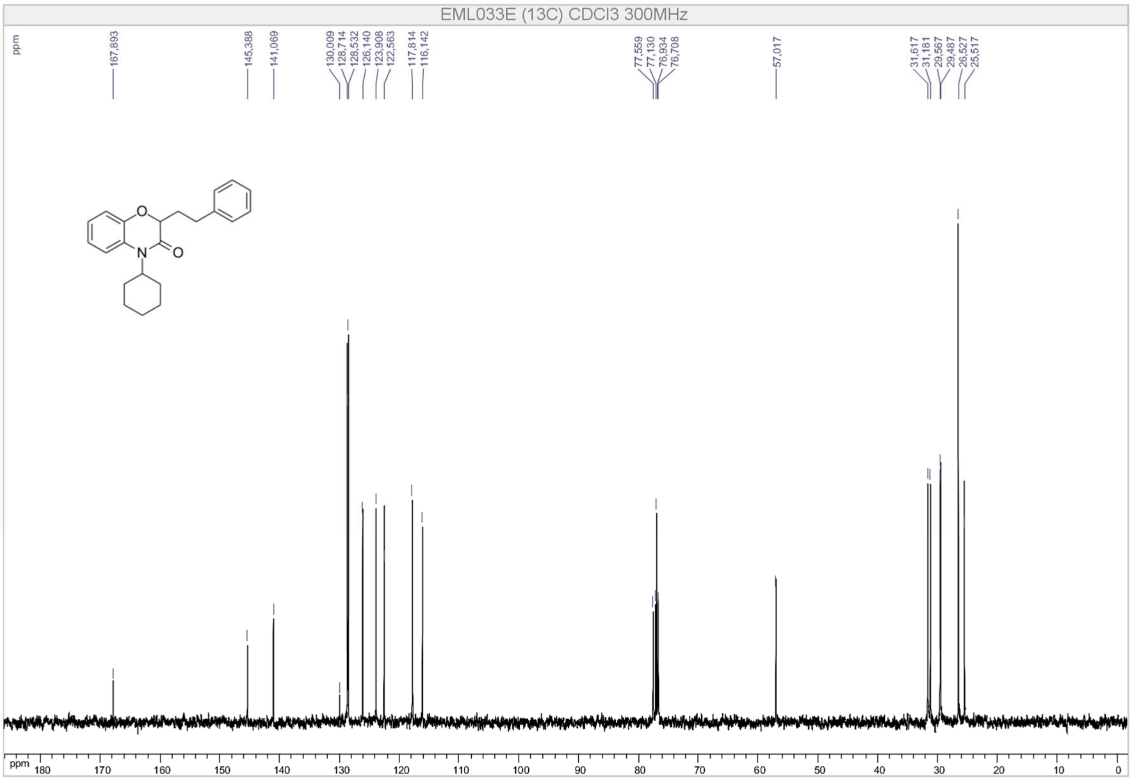

4-(Tert-butyl)-7-chloro-2-ethyl-2H-benzo[b][1,4]oxazin-3(4H)-one (**6s**)

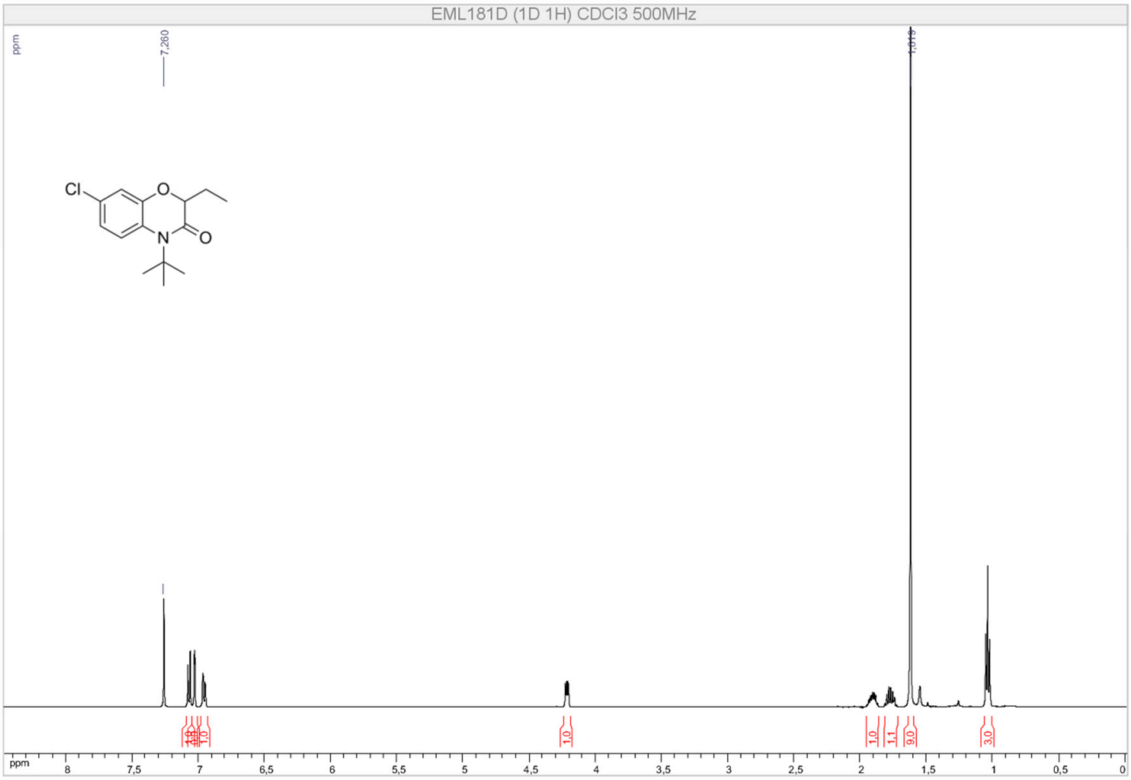

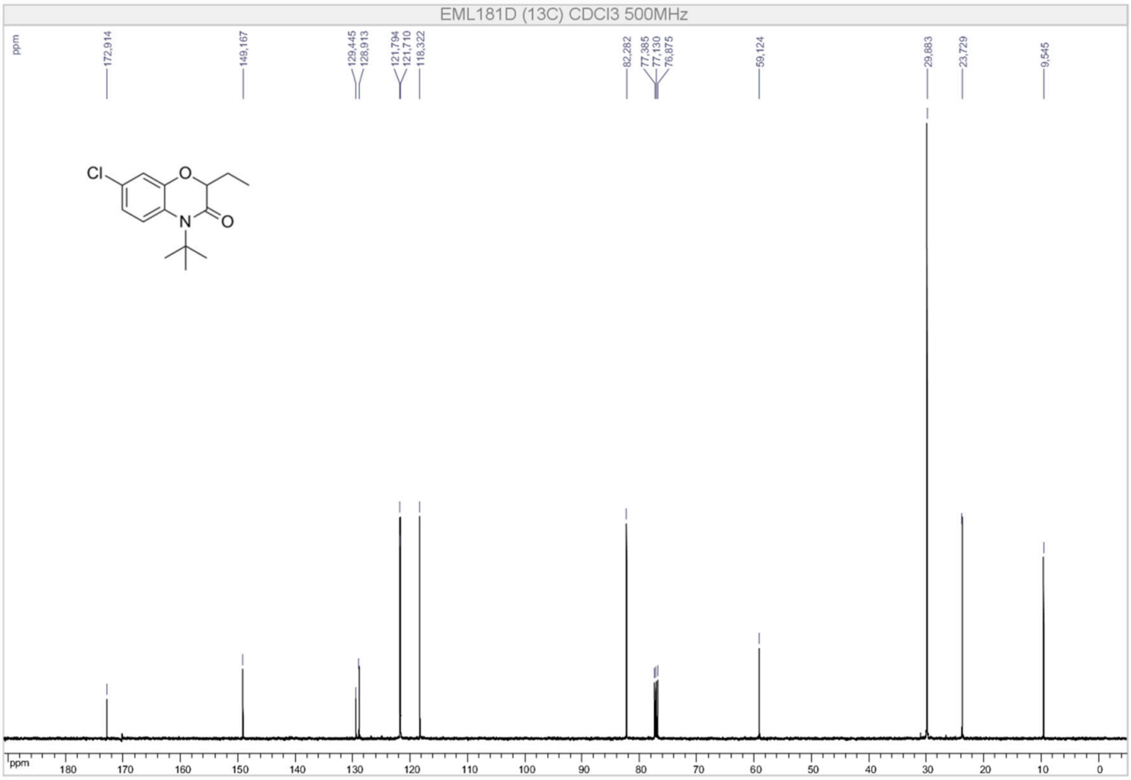

7-Chloro-2-(methoxymethyl)-2-methyl-4-phenethyl-2H-benzo[b][1,4]oxazin-3(4H)-one (**6t**)

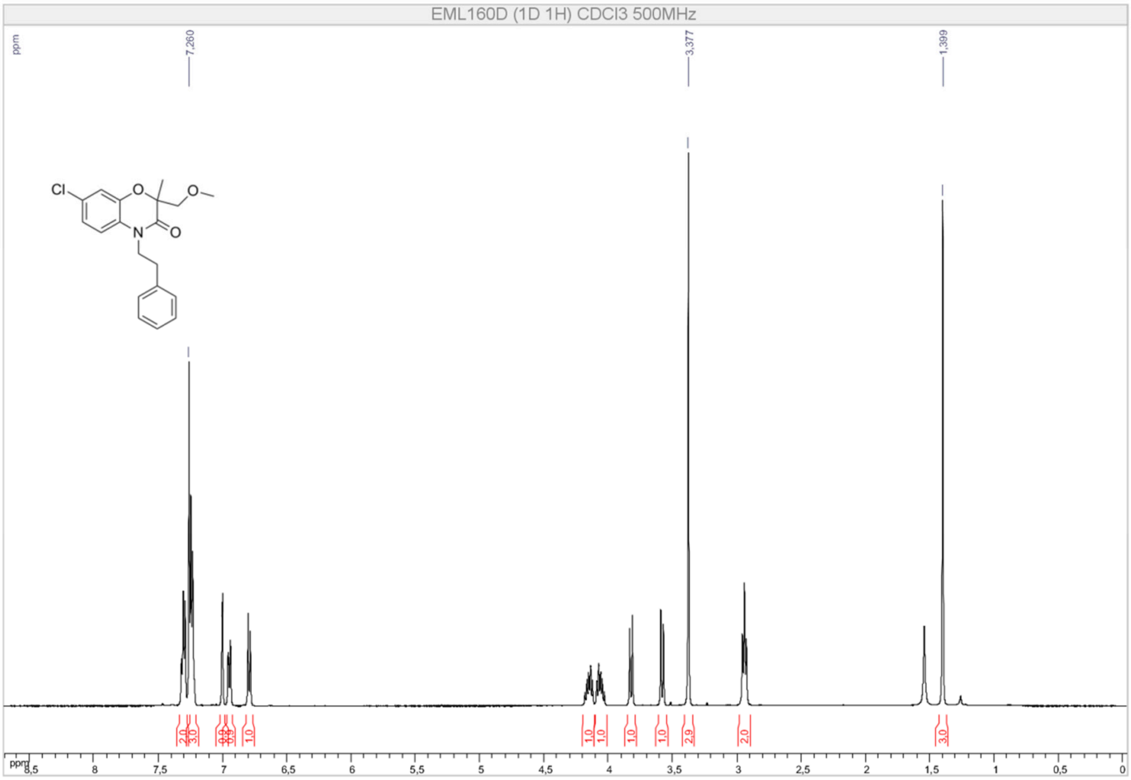

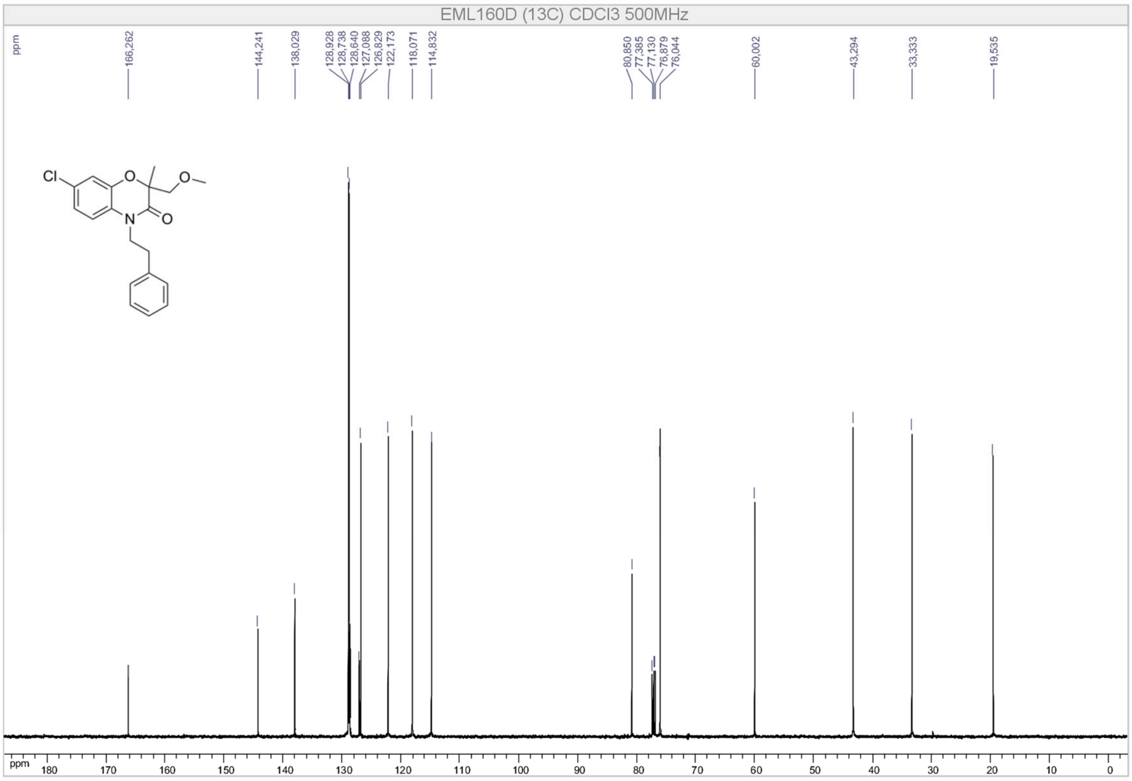

2-(Tert-butyl)-7-chloro-4-(4-chlorobenzyl)-2H-benzo[b][1,4]oxazin-3(4H)-one (**6u**)

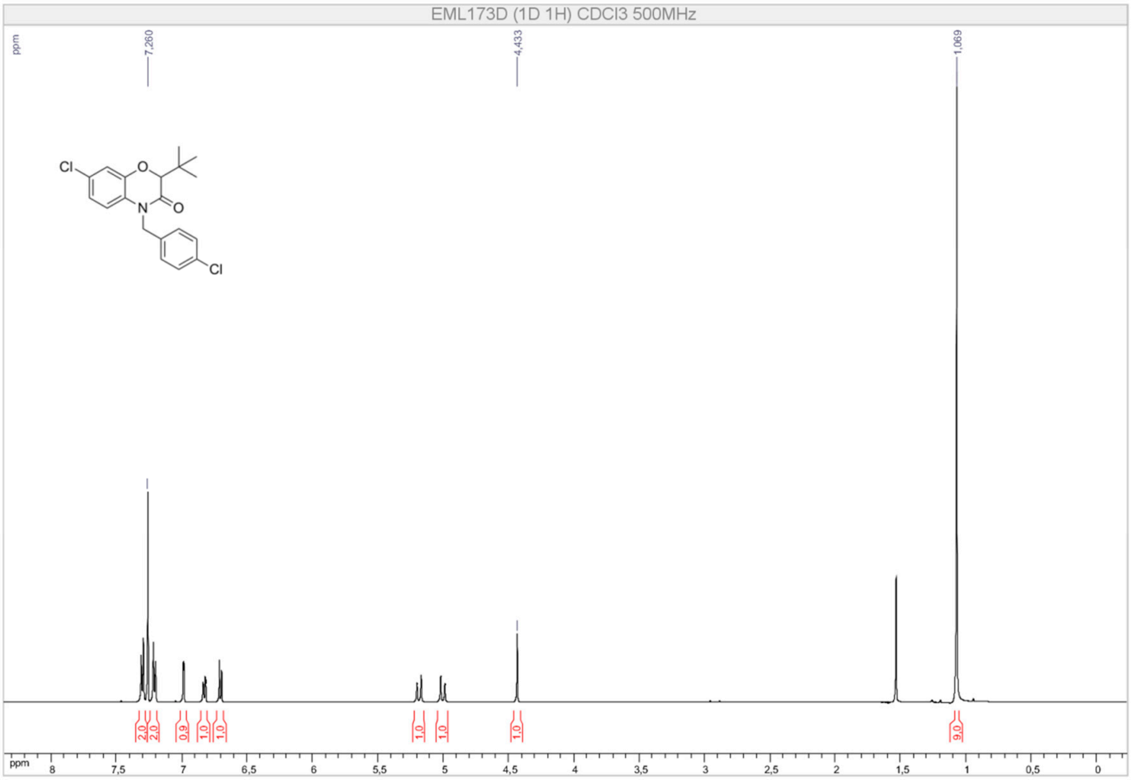

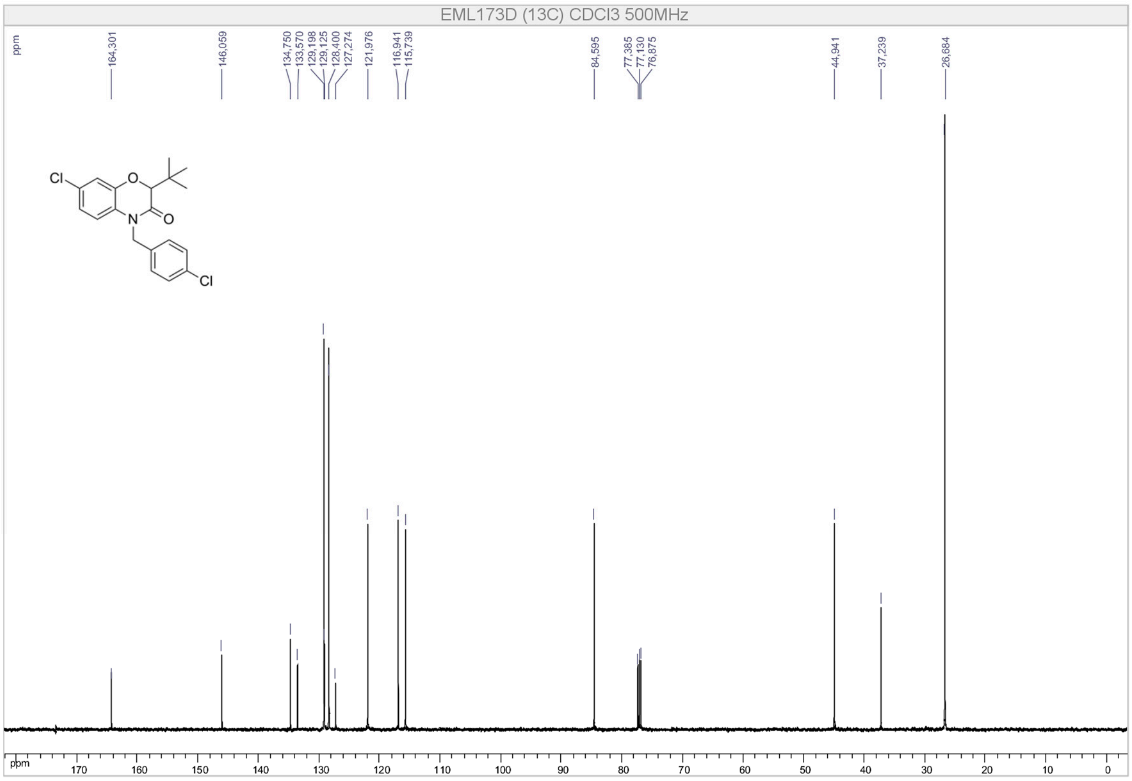

4-Cyclohexyl-2-(methoxymethyl)-2-methyl-2H-benzo[b][1,4]oxazin-3(4H)-one (**6v**)

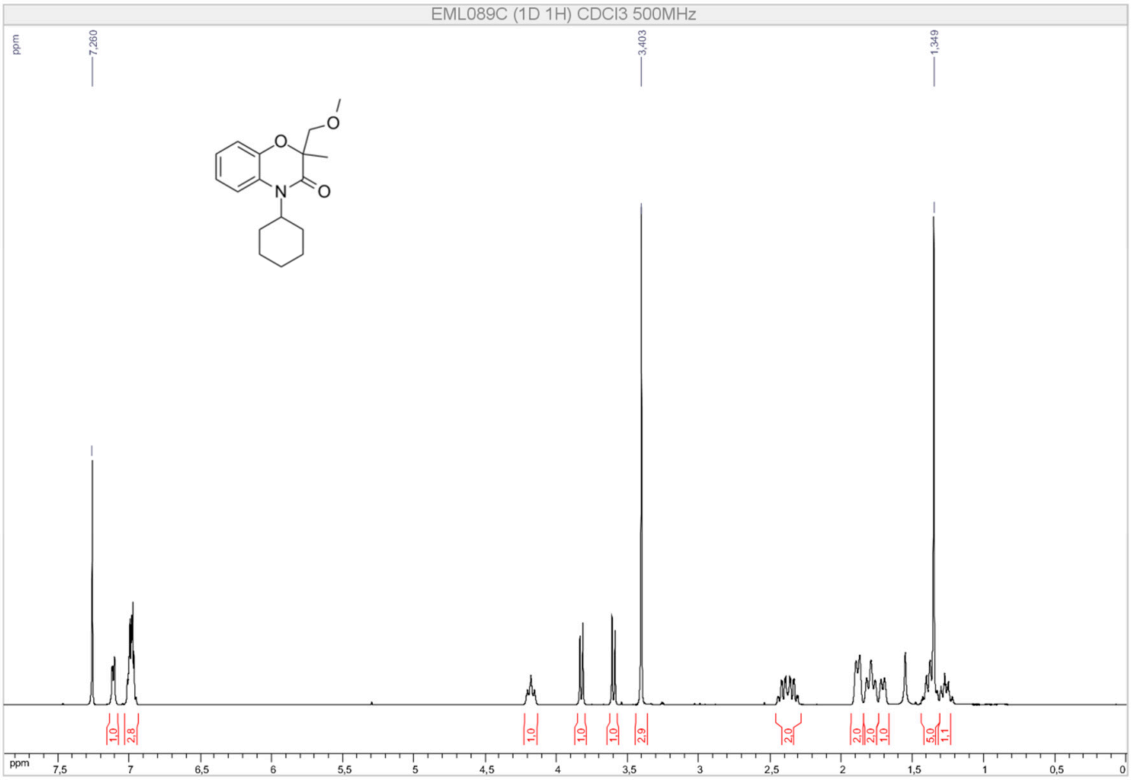

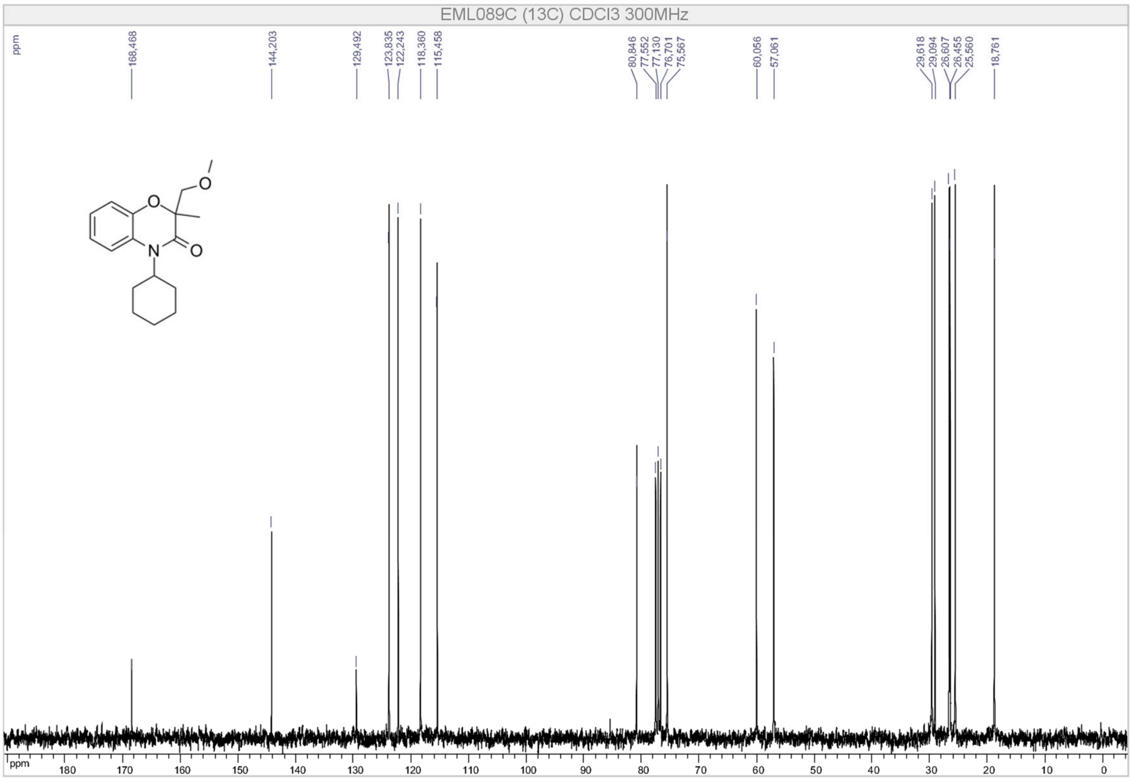

4-Cyclohexylspiro[benzo[b][1,4]oxazine-2,1′-cyclobutan]-3(4H)-one (**6w**)

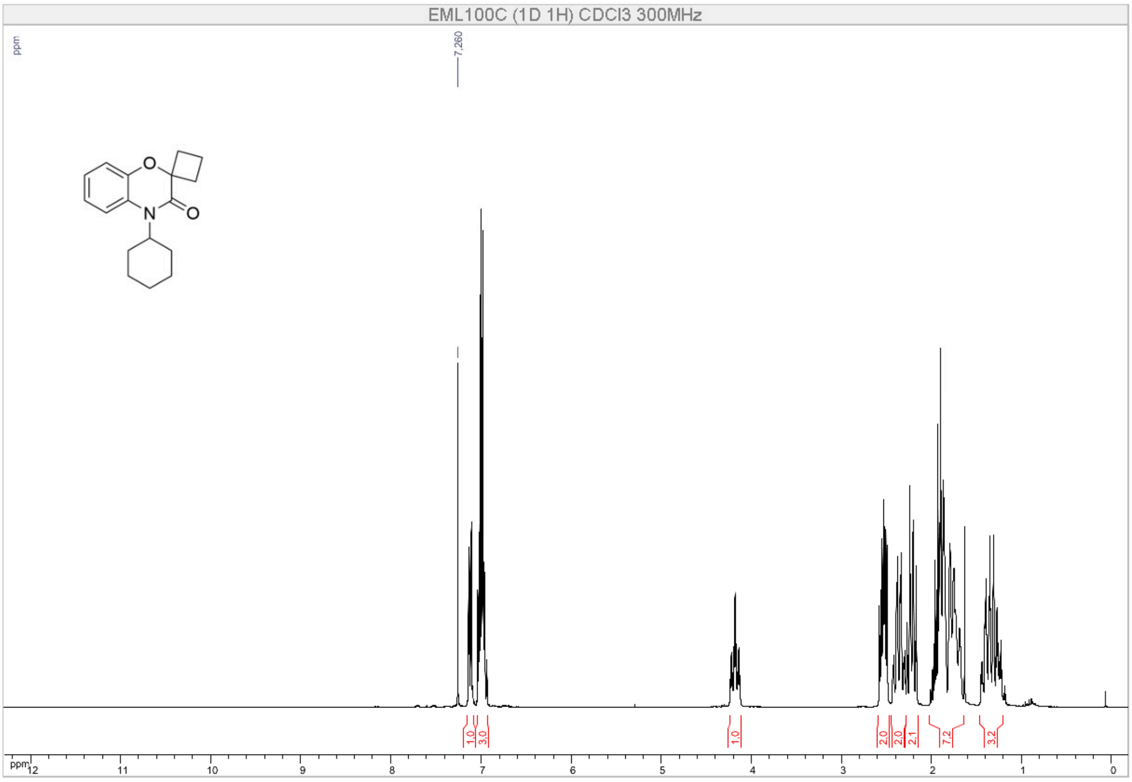

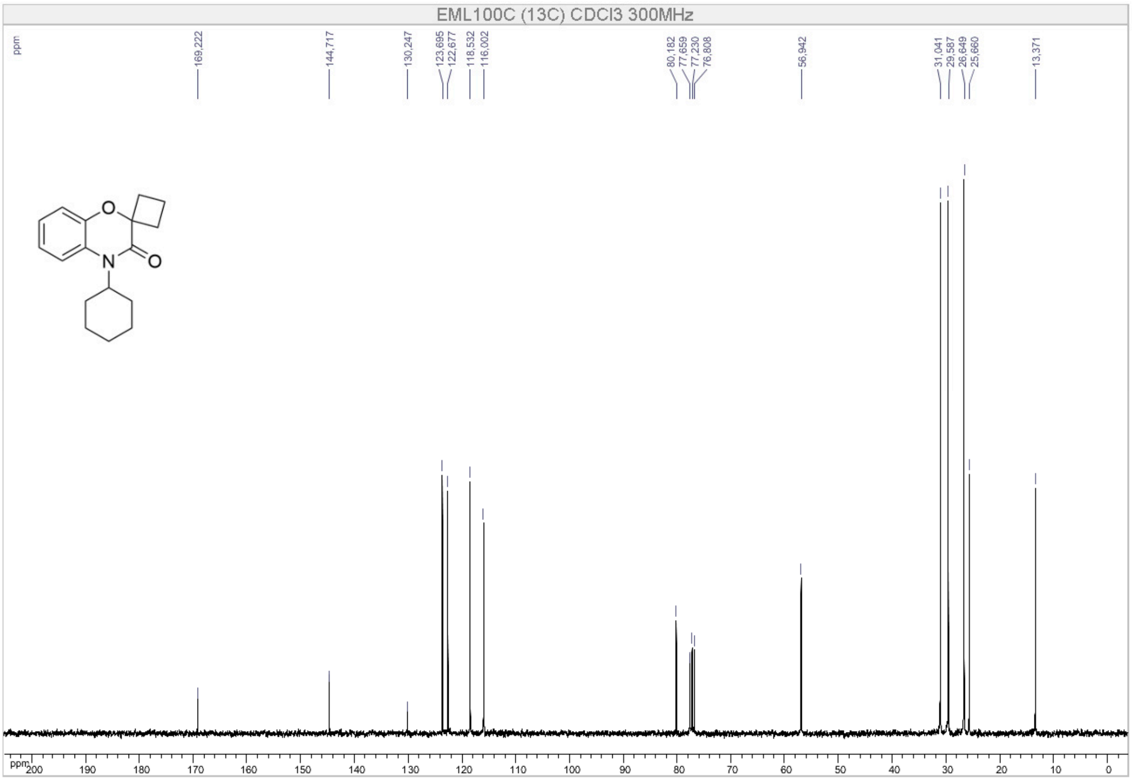

4-Cyclohexyl-2-ethyl-2H-pyrido[3,2-b][1,4]oxazin-3(4H)-one (**6x**)

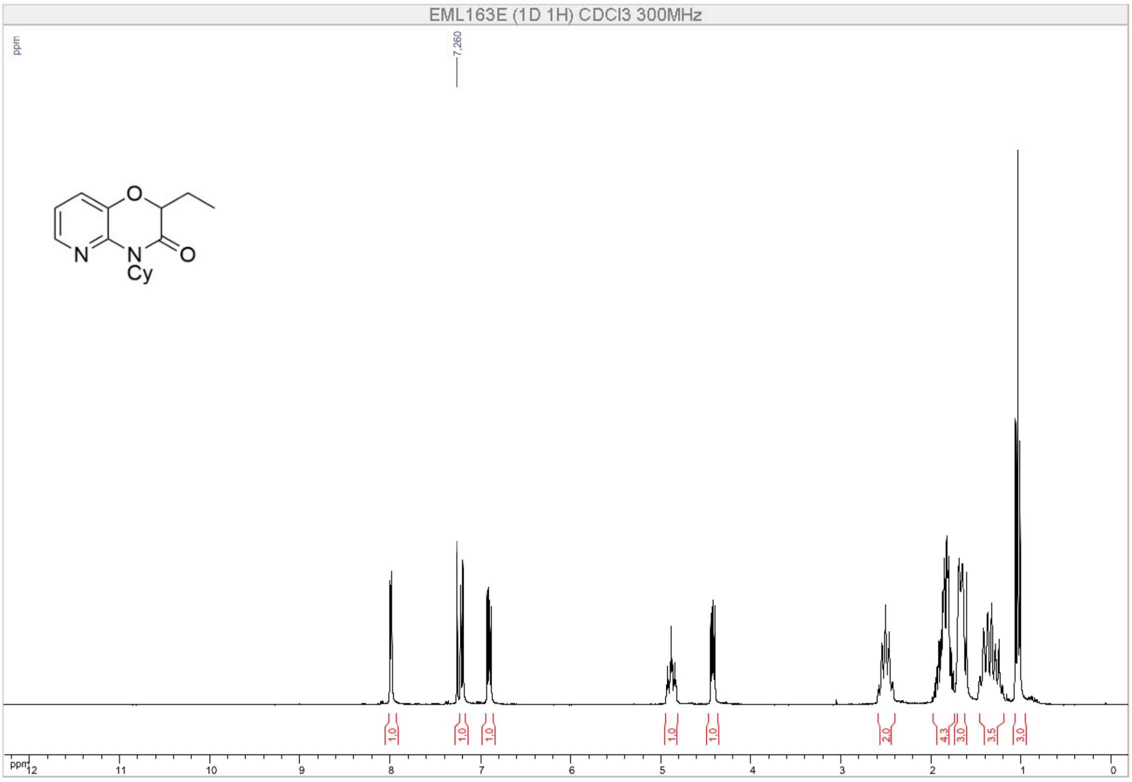

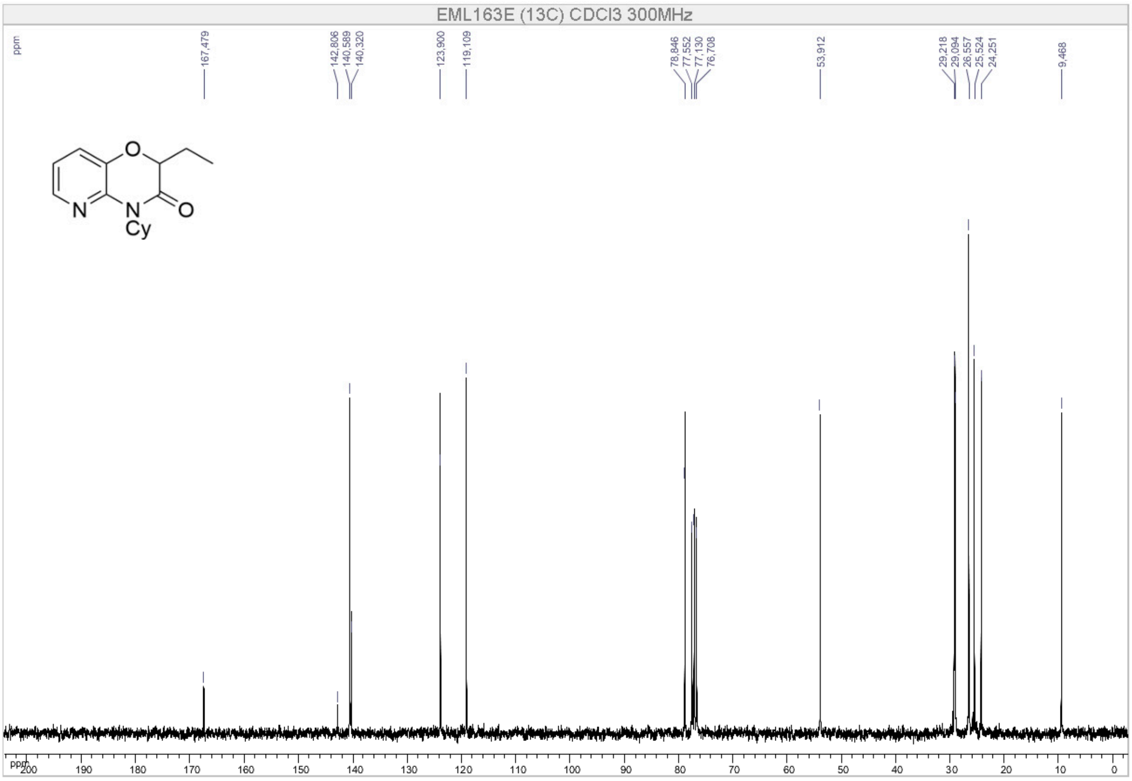

1-Cyclohexyl-3-isopropyl-1H-pyrido[2,3-b][1,4]oxazin-2(3H)-one (**6y**)

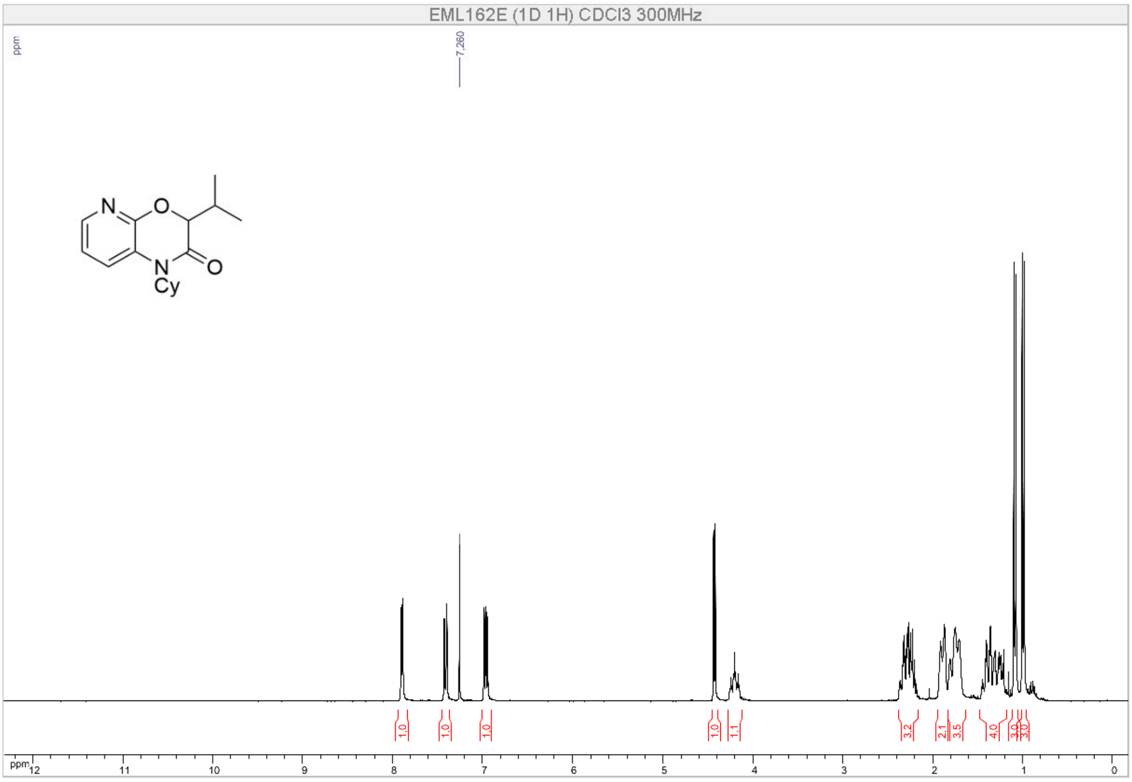

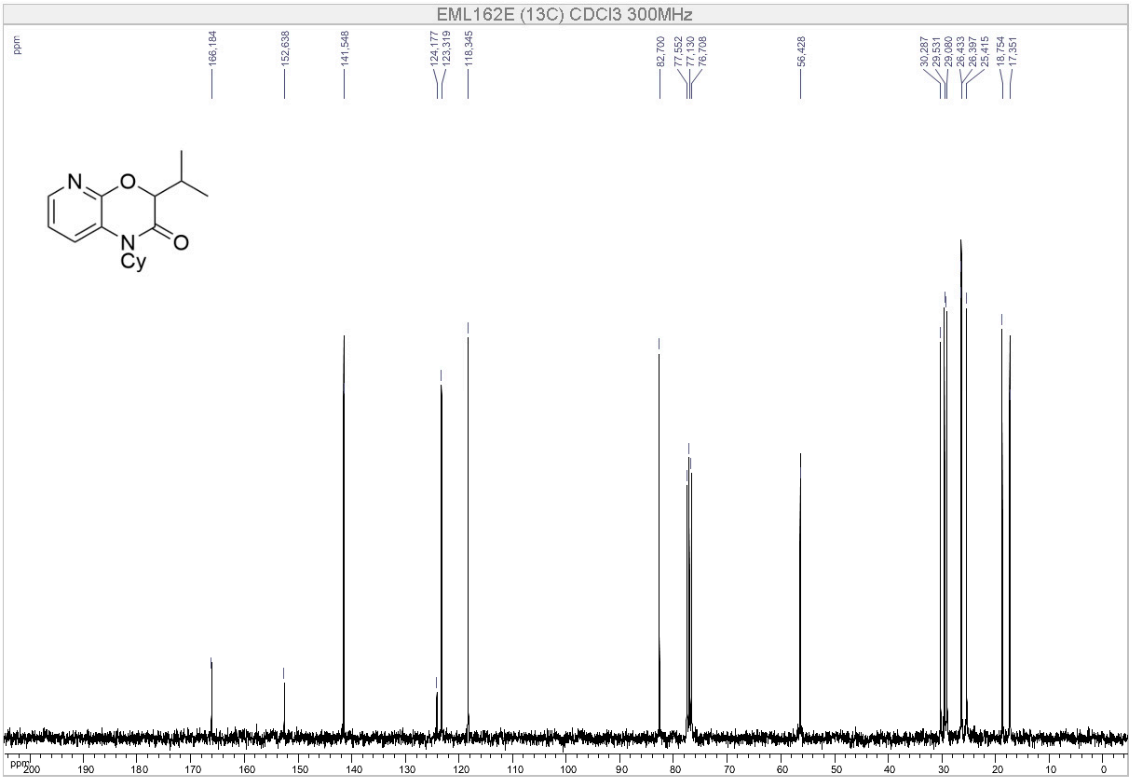

2-(4-Chlorophenyl)-N-cyclohexyl-2-oxoacetamide (**7**)

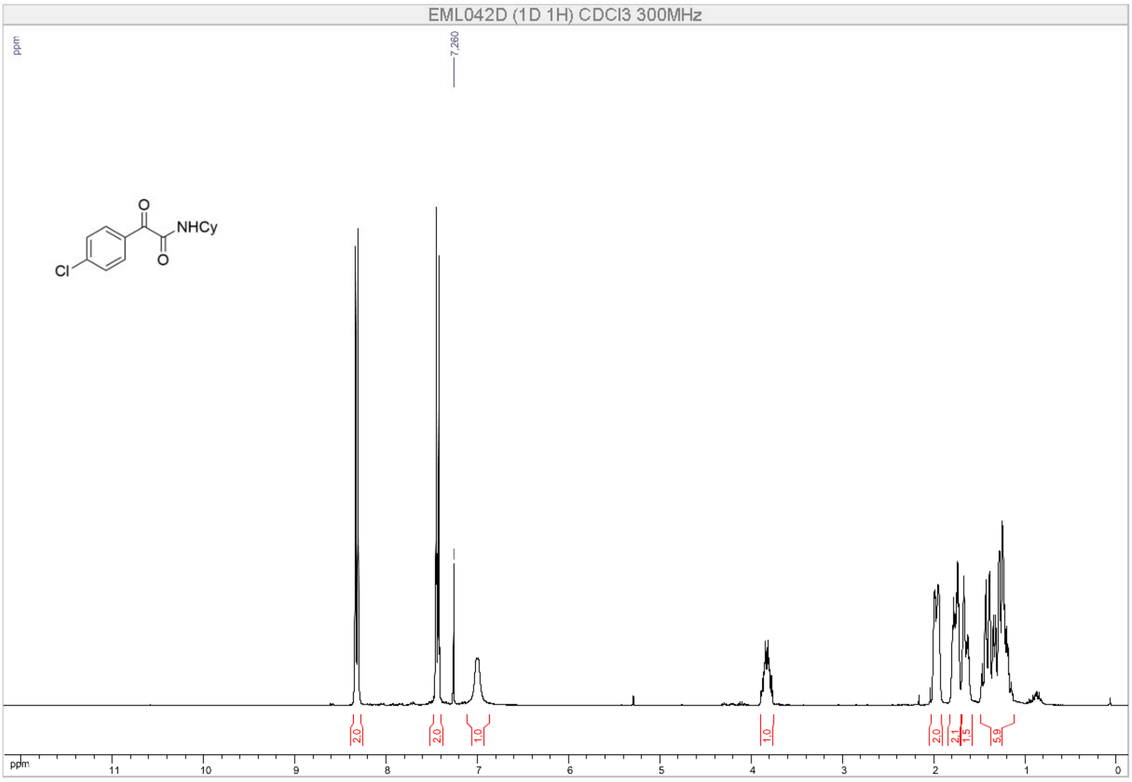

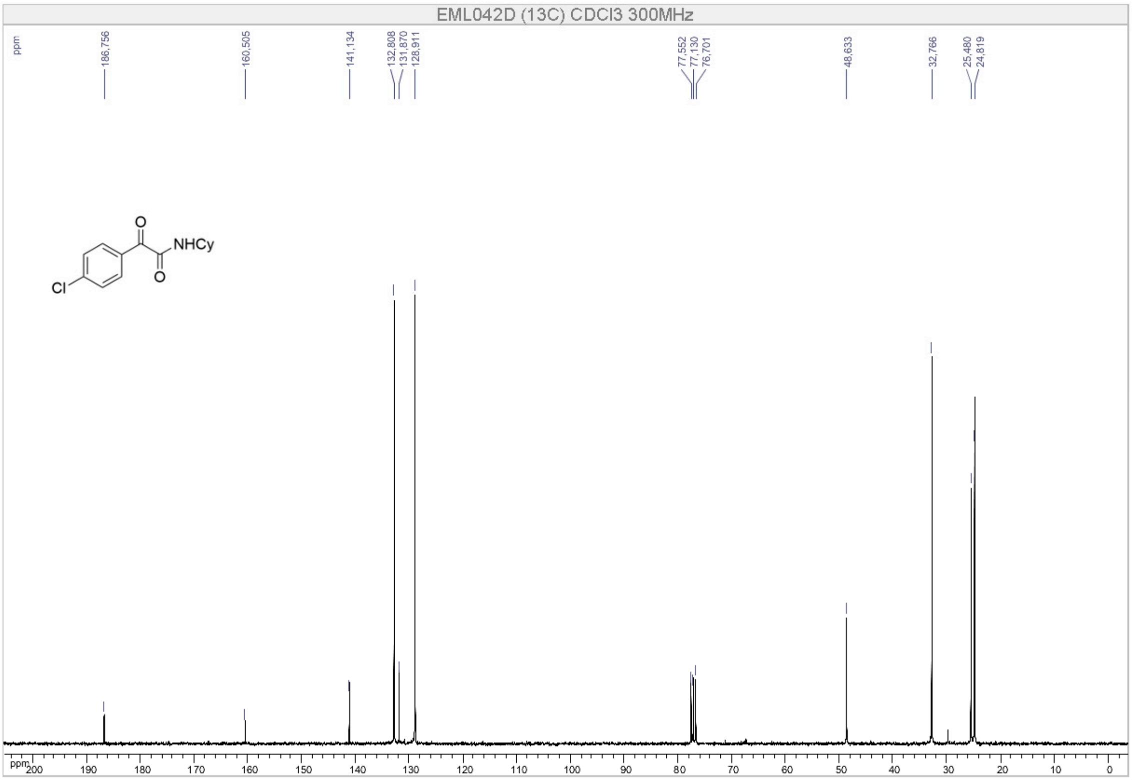

4-(4-Chlorobenzyl)-2-((S)-2,6-dimethylhept-5-en-1-yl)-7-nitro-2H-benzo[b][1,4]oxazin-3(4H)-one (**8a**)

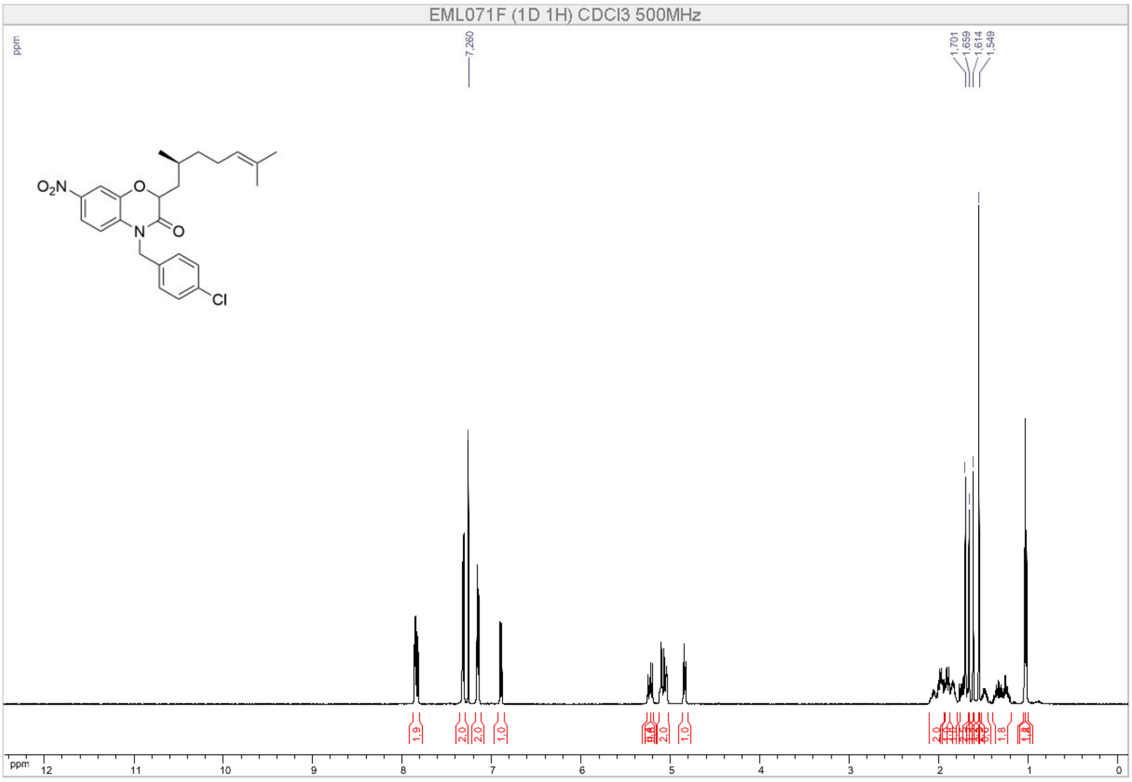

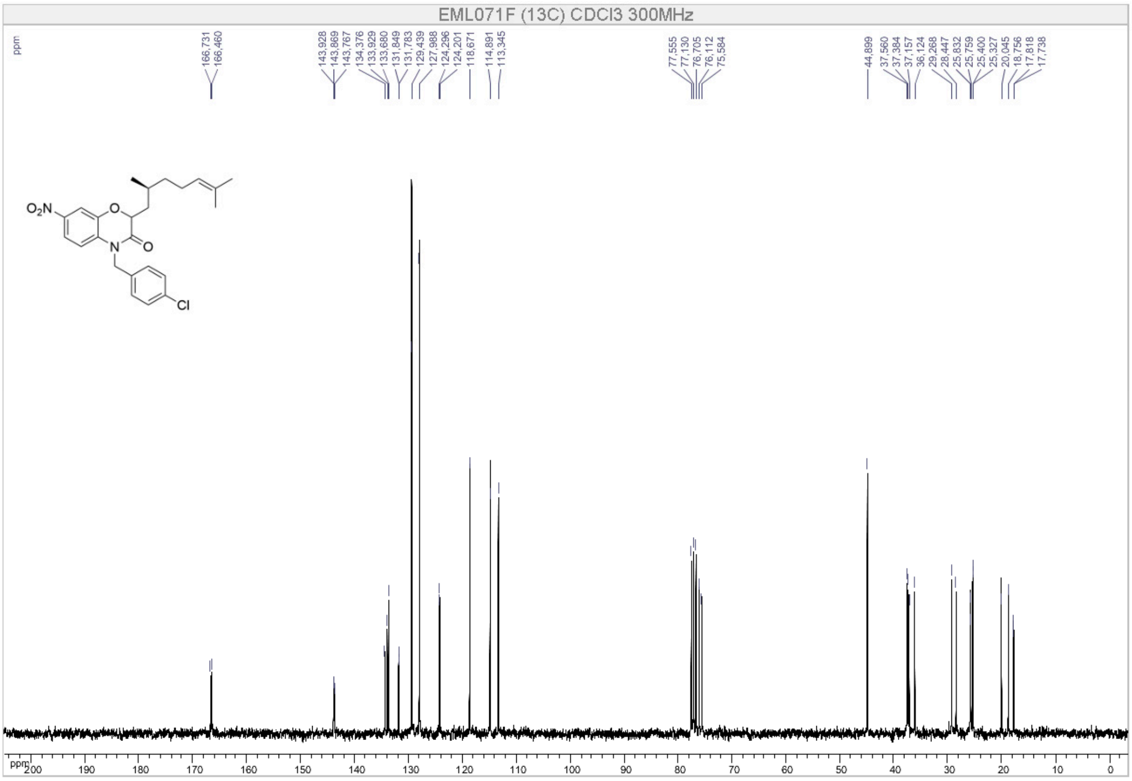

4-Cyclohexyl-2-ethyl-7-nitro-2H-benzo[b][1,4]oxazin-3(4H)-one (**8b**)

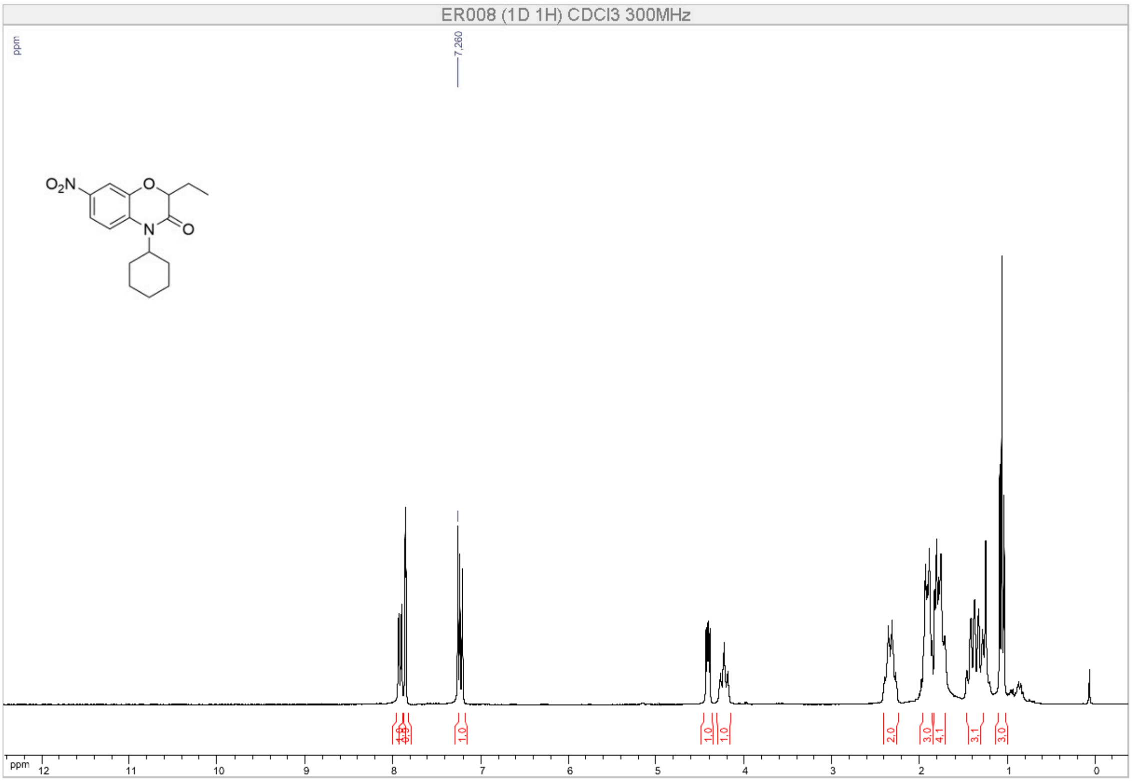

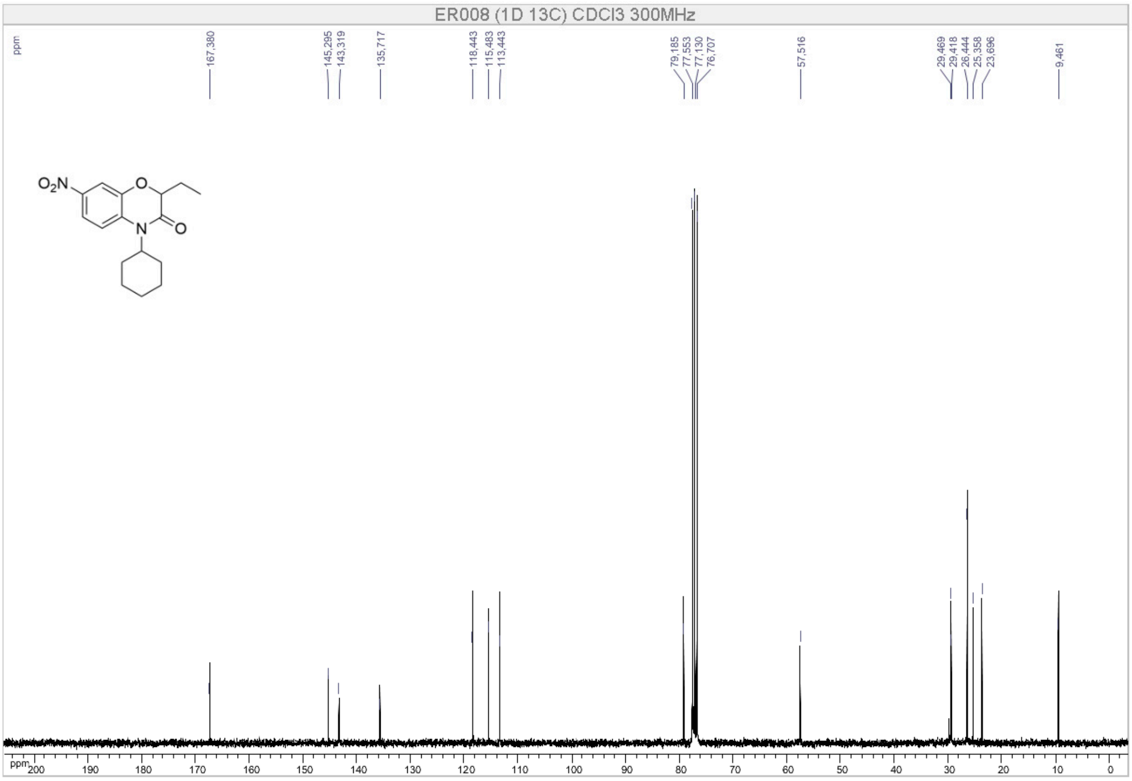

2-Isopropyl-4-(4-methoxybenzyl)-7-nitro-2H-benzo[b][1,4]oxazin-3(4H)-one (**8c**)

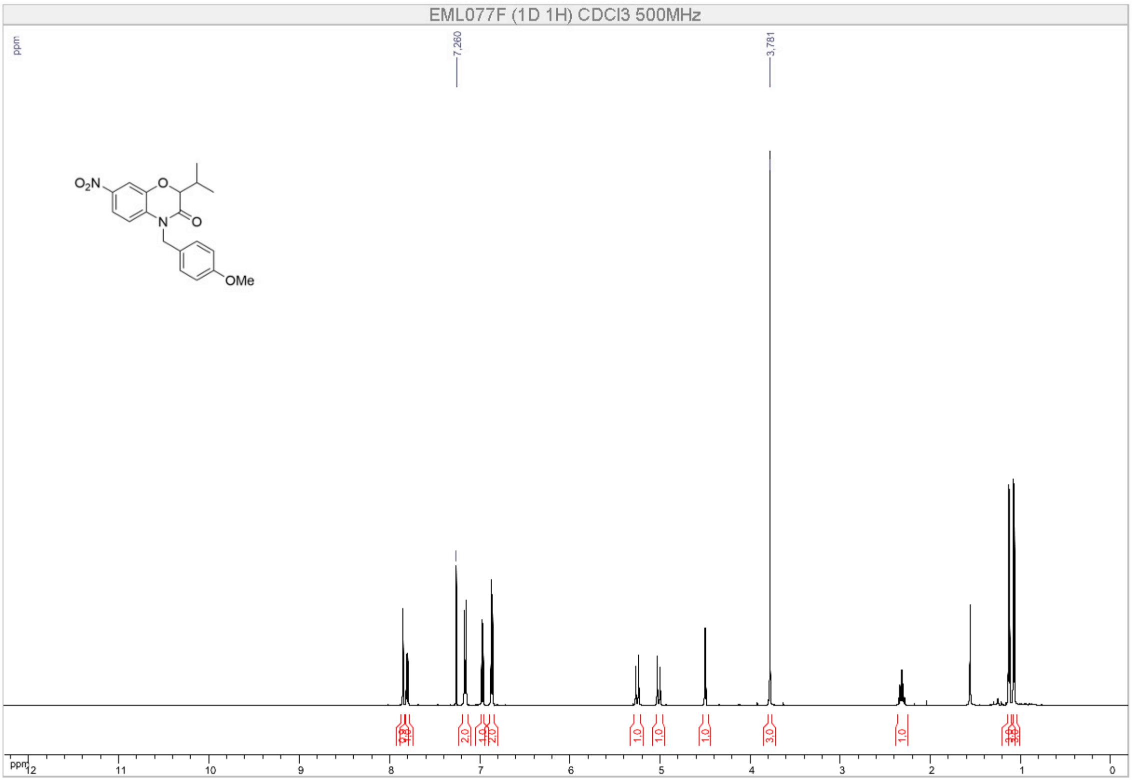

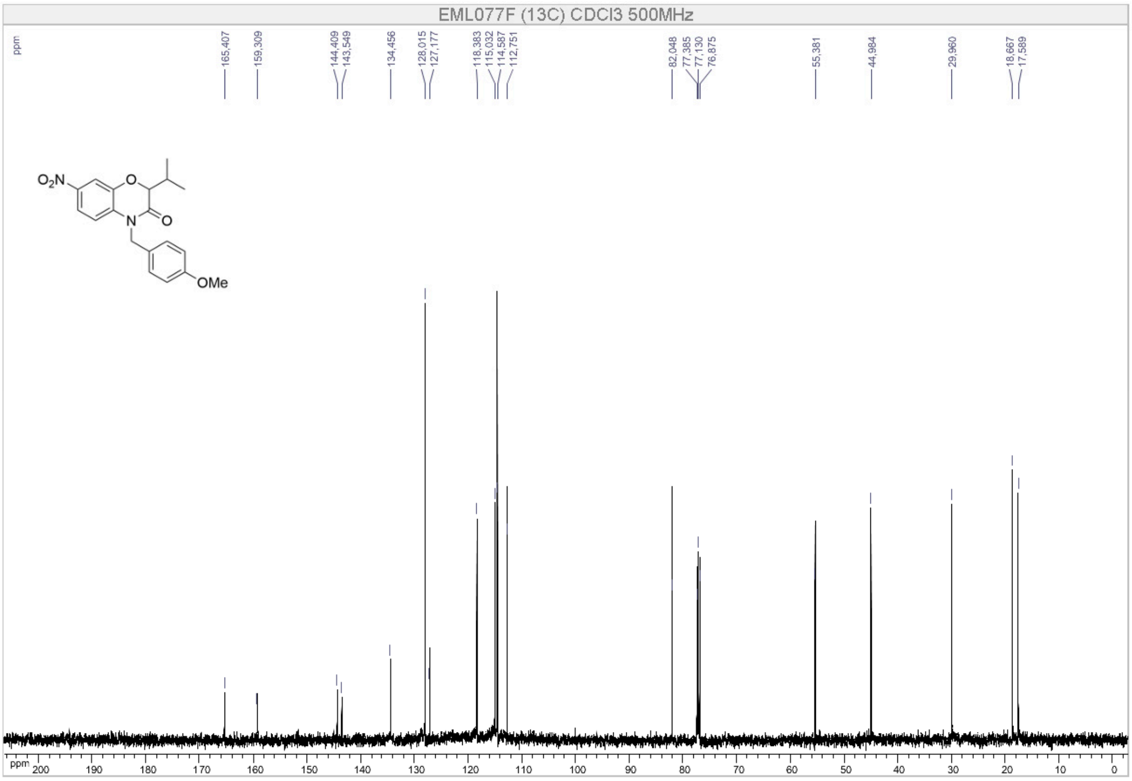

2-(Tert-butyl)-7-nitro-4-phenethyl-2H-benzo[b][1,4]oxazin-3(4H)-one (**8d**)

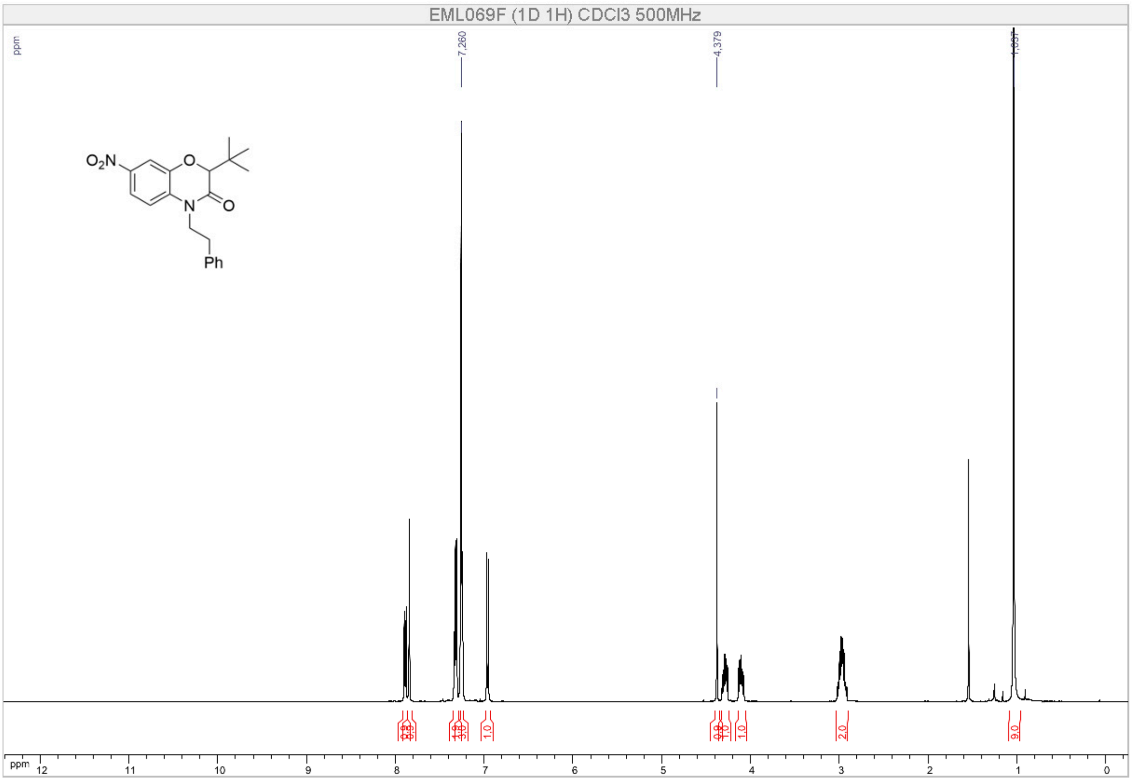

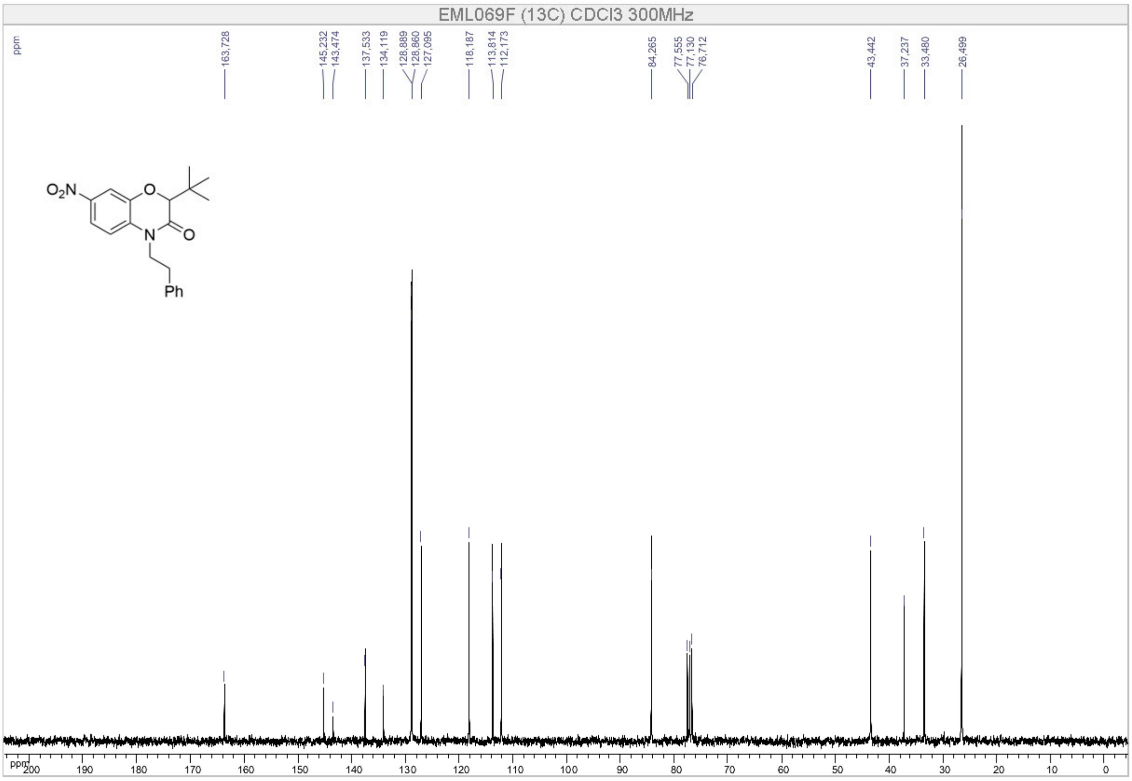

4-(4-Methoxybenzyl)-2-(2-methylpent-4-en-2-yl)-7-nitro-2H-benzo[b][1,4]oxazin-3(4H)-one (**8e**)

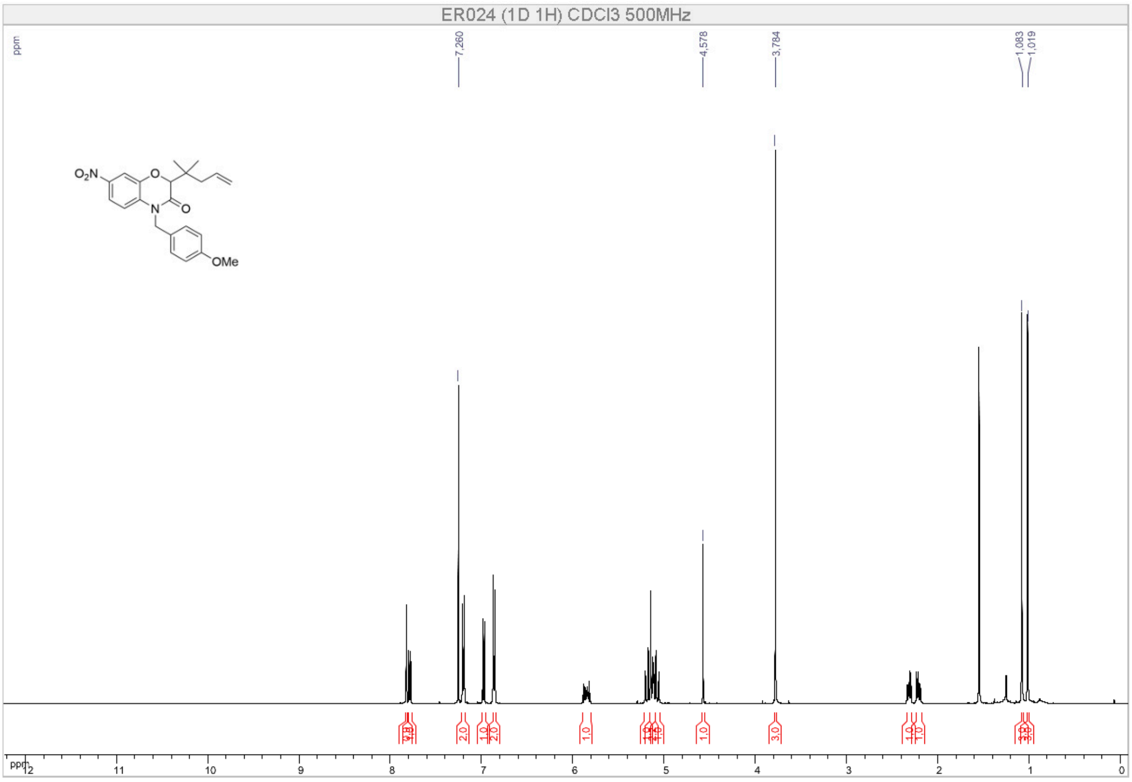

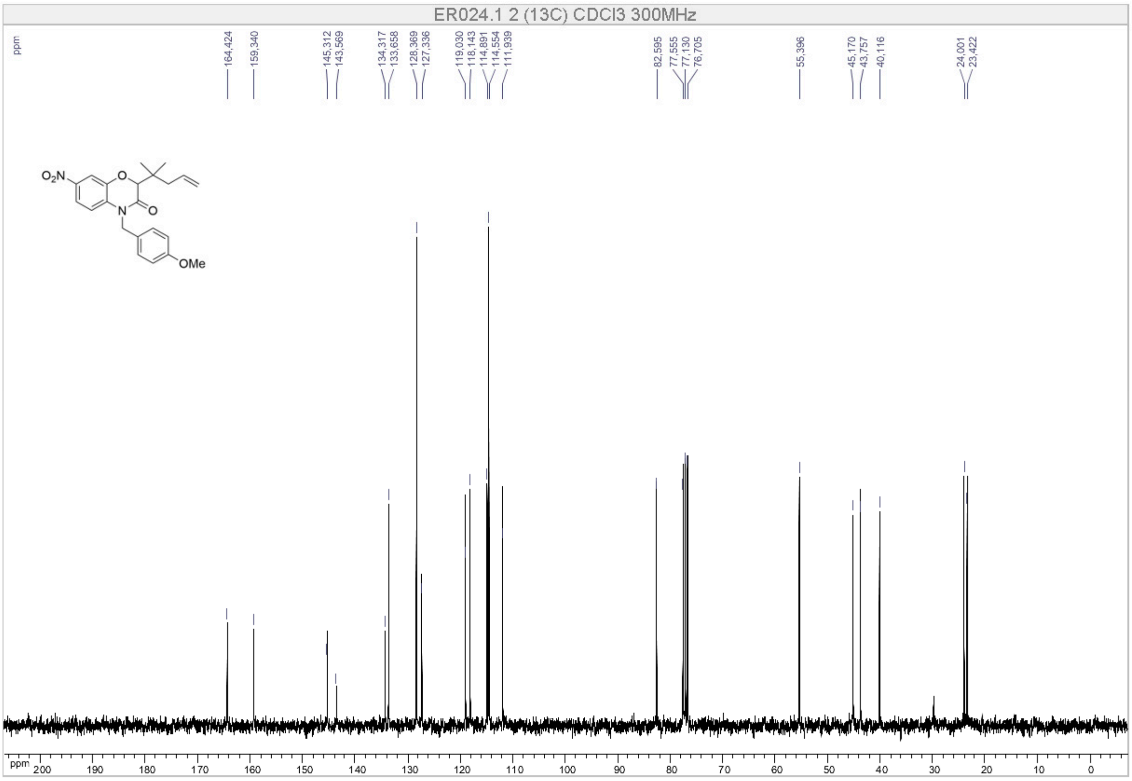

2-(Tert-butyl)-4-cyclohexyl-7-nitro-2H-benzo[b][1,4]oxazin-3(4H)-one (**8f**)

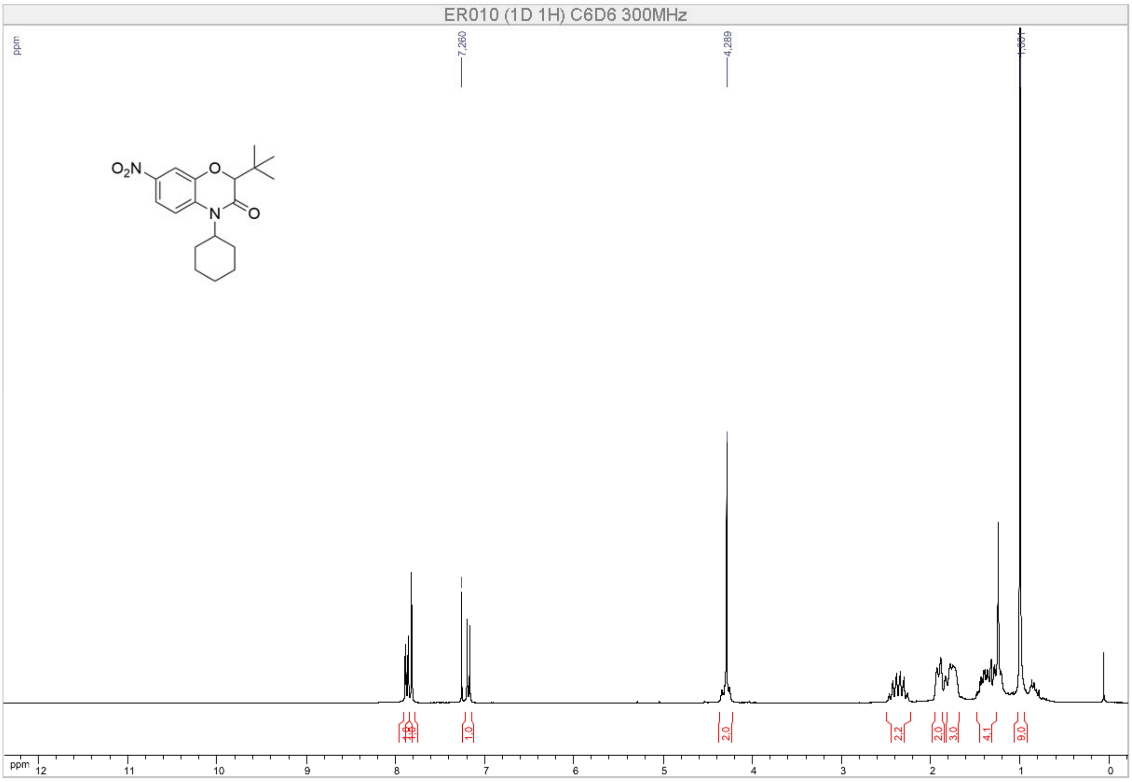

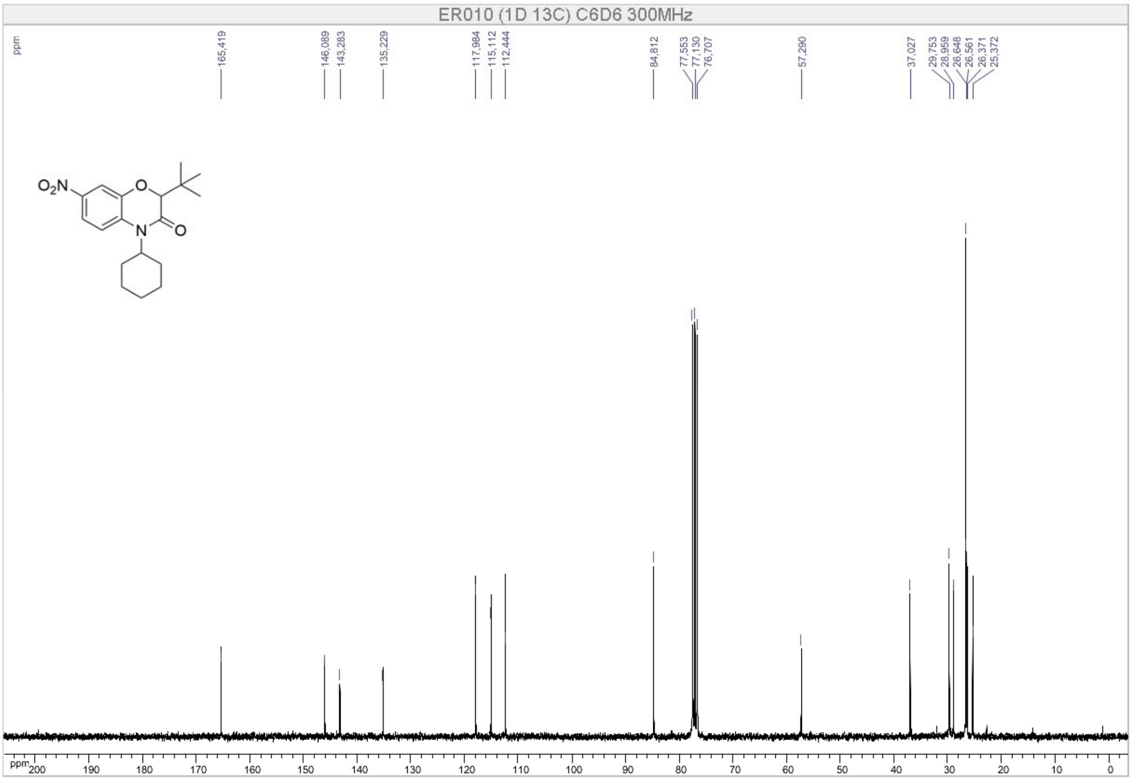

7-Nitro-2,4-diphenethyl-2H-benzo[b][1,4]oxazin-3(4H)-one (**8g**)

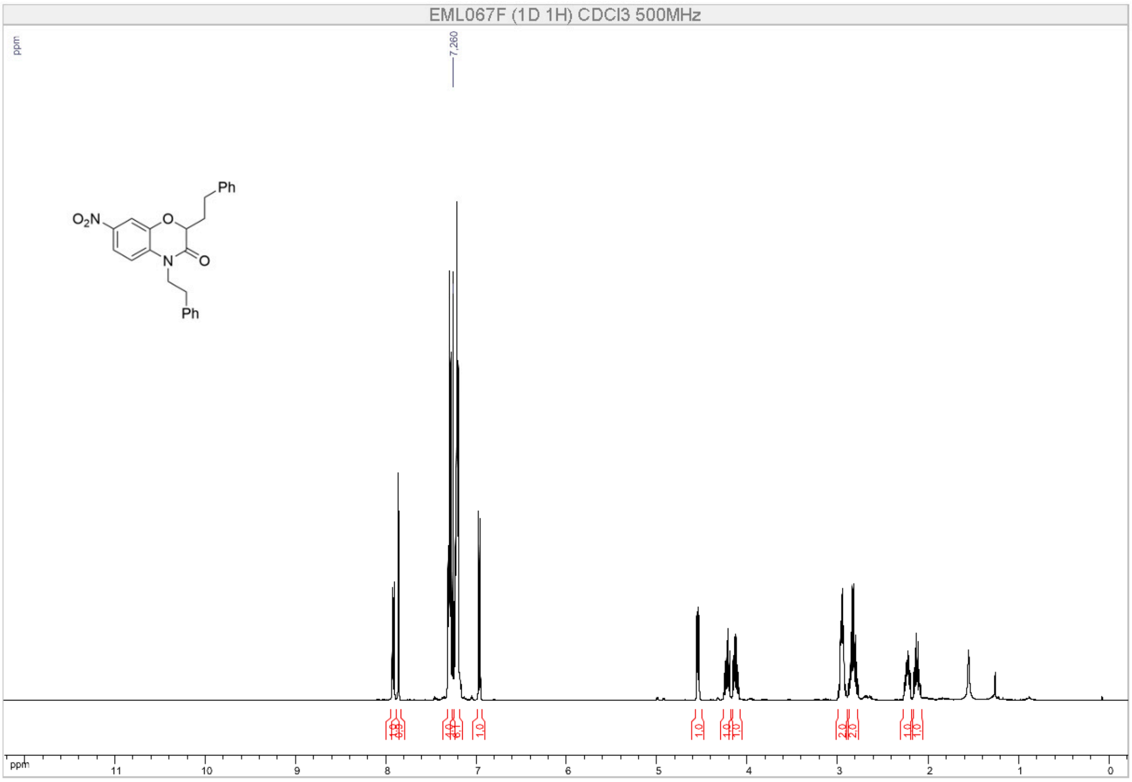

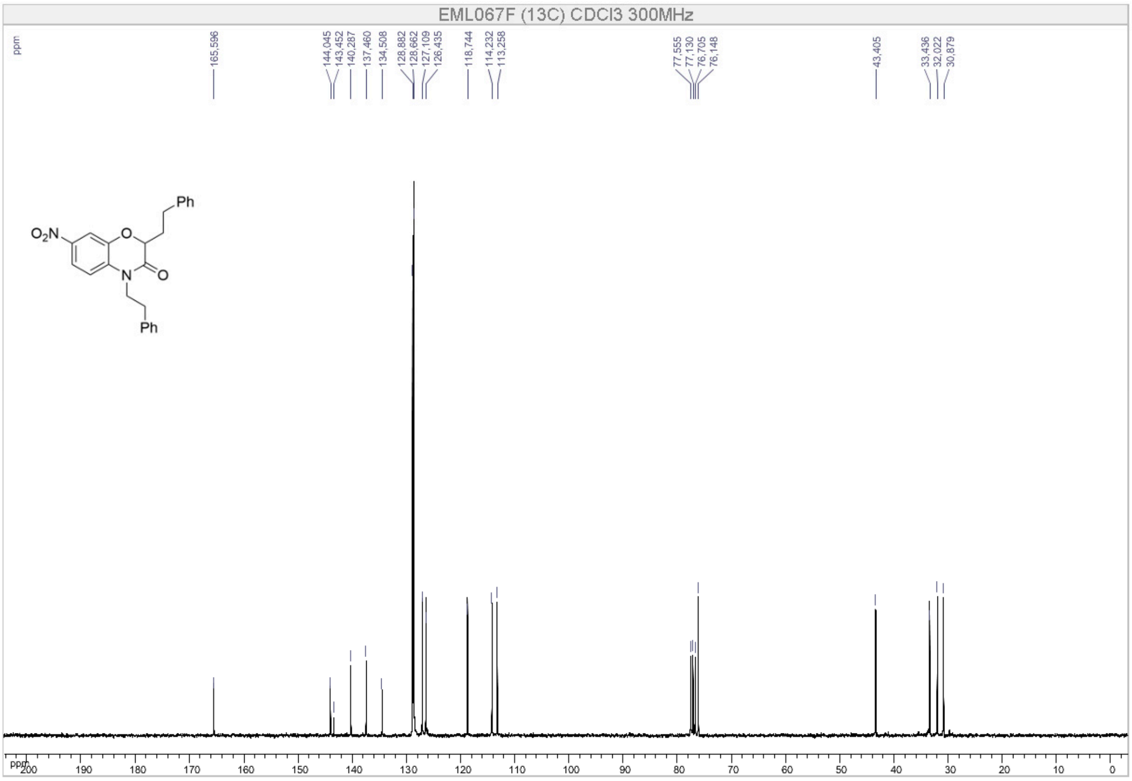

4-(3,4-Dimethoxyphenethyl)-2-((S)-2,6-dimethylhept-5-en-1-yl)-7-nitro-2H-benzo[b][1,4]oxazin-3(4H)-one (**8h**)

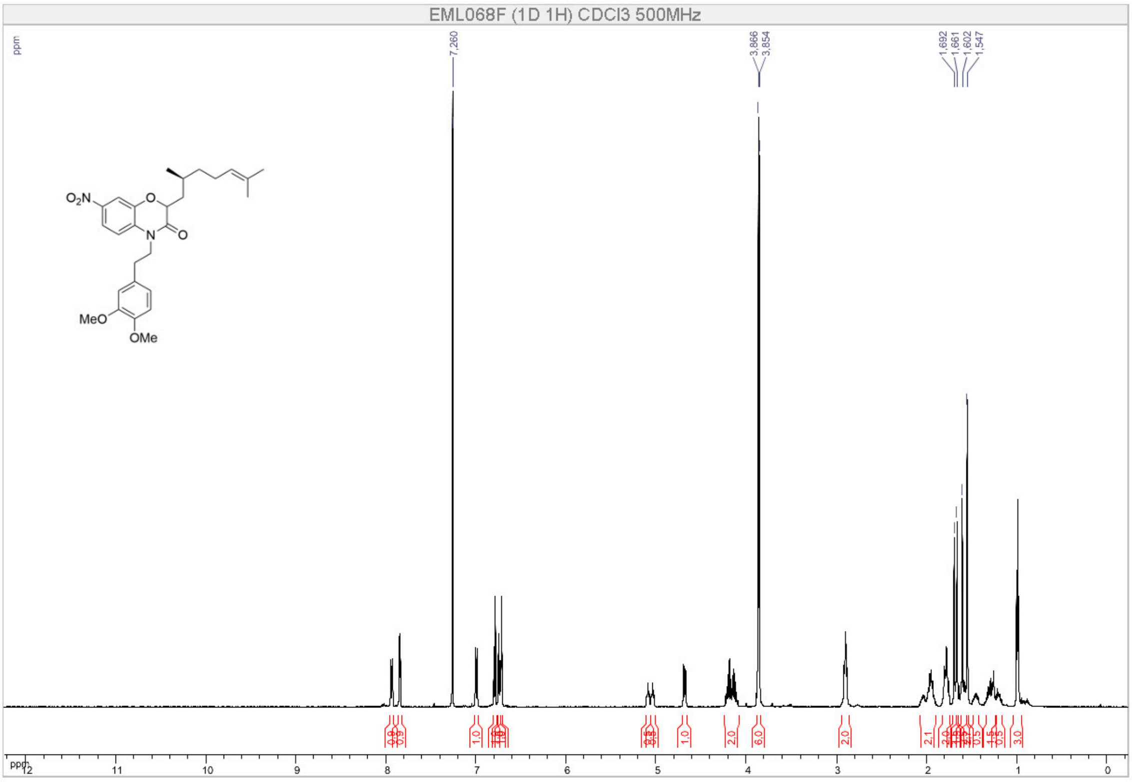

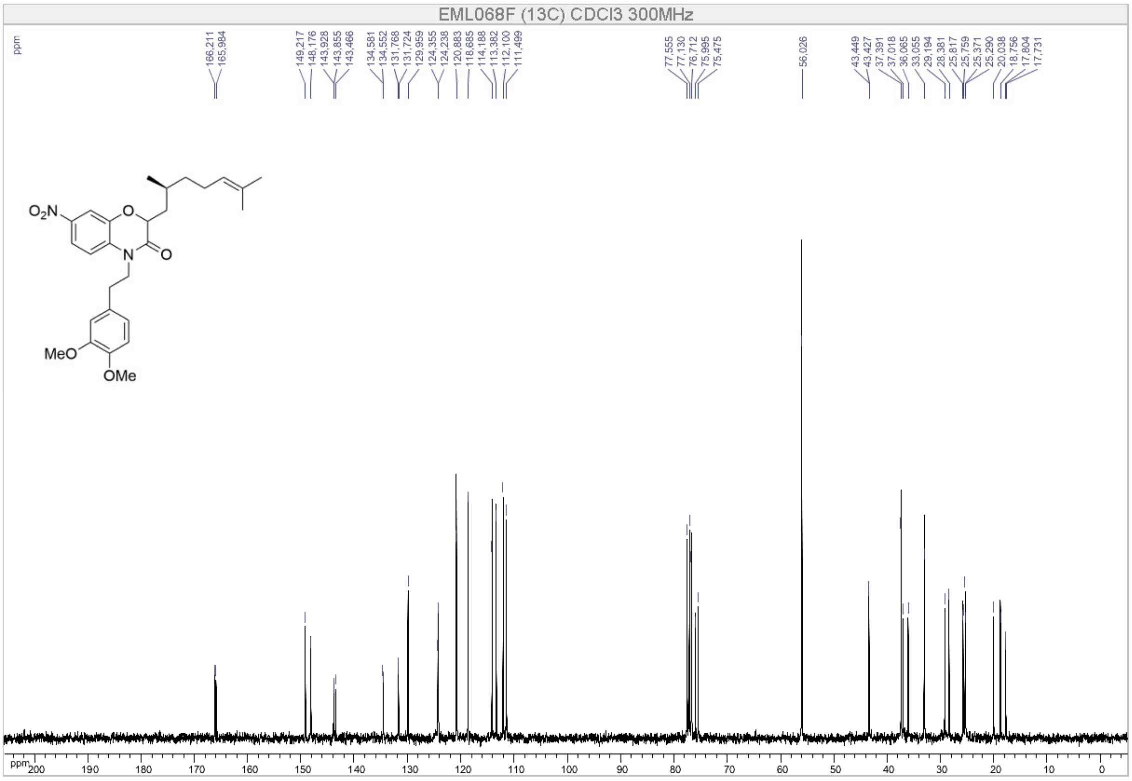

4-(4-Chlorobenzyl)-2-ethyl-7-nitro-2H-benzo[b][1,4]oxazin-3(4H)-one (**8i**)

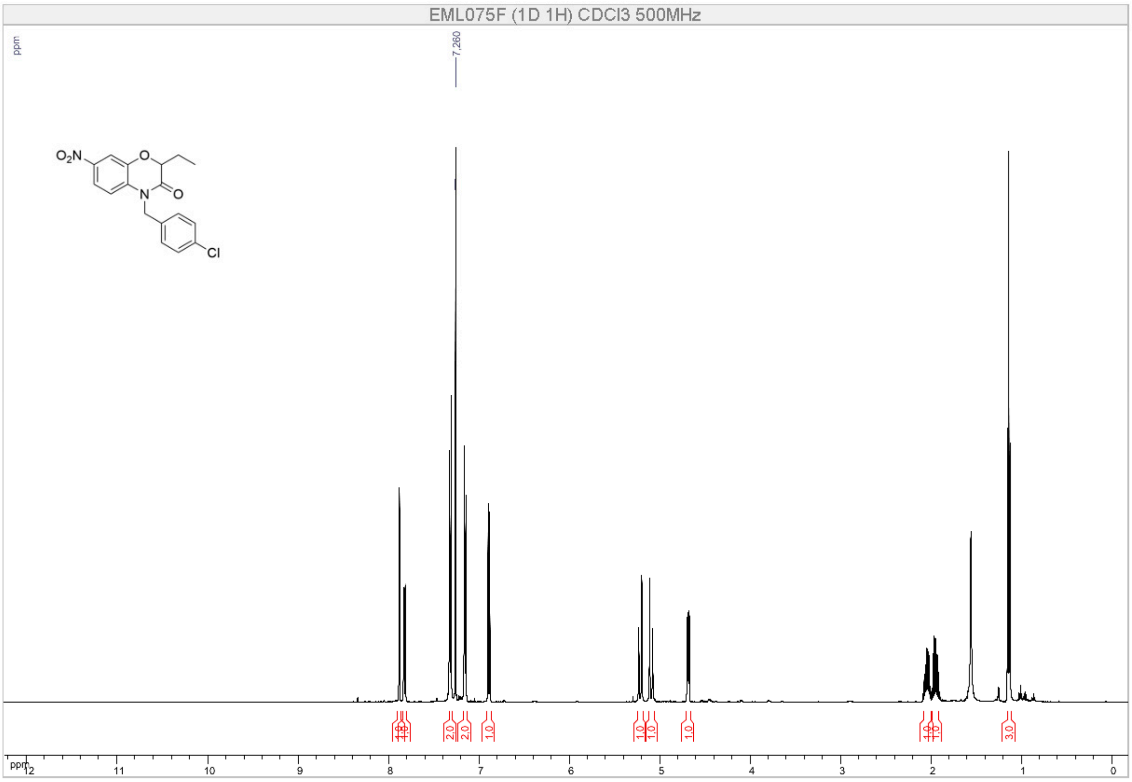

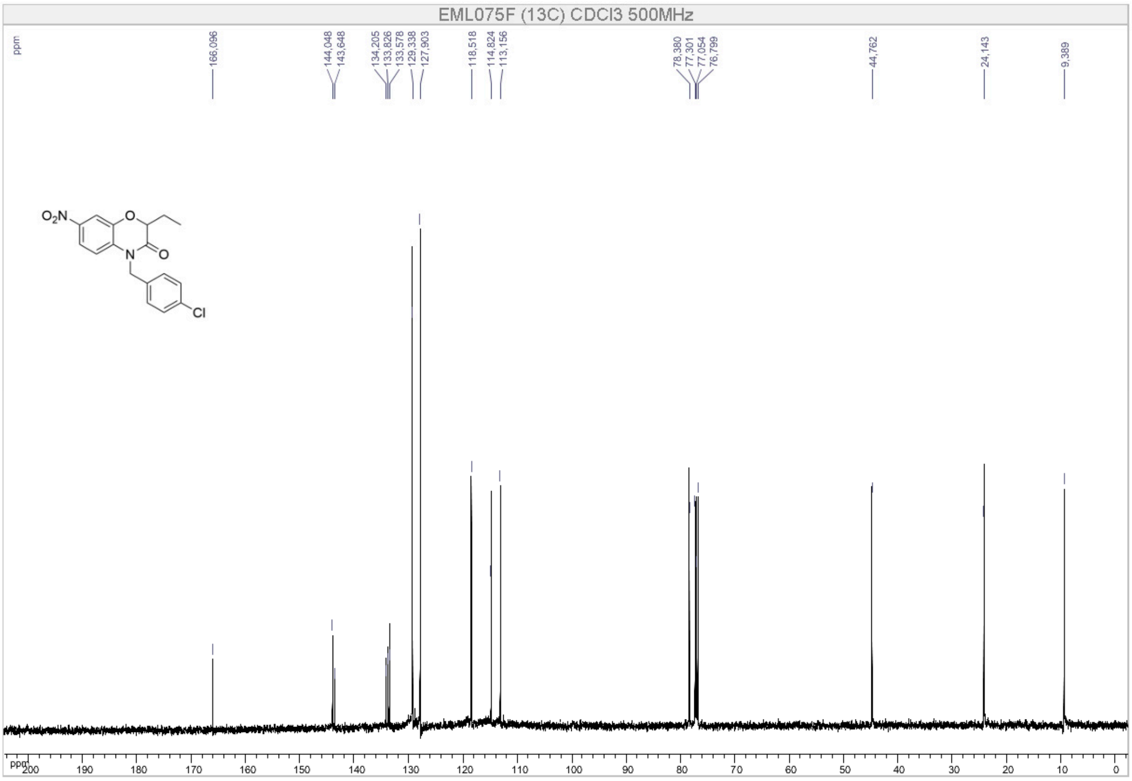

4-Cyclohexyl-2-isopropyl-7-nitro-2H-benzo[b][1,4]oxazin-3(4H)-one (**8j**)

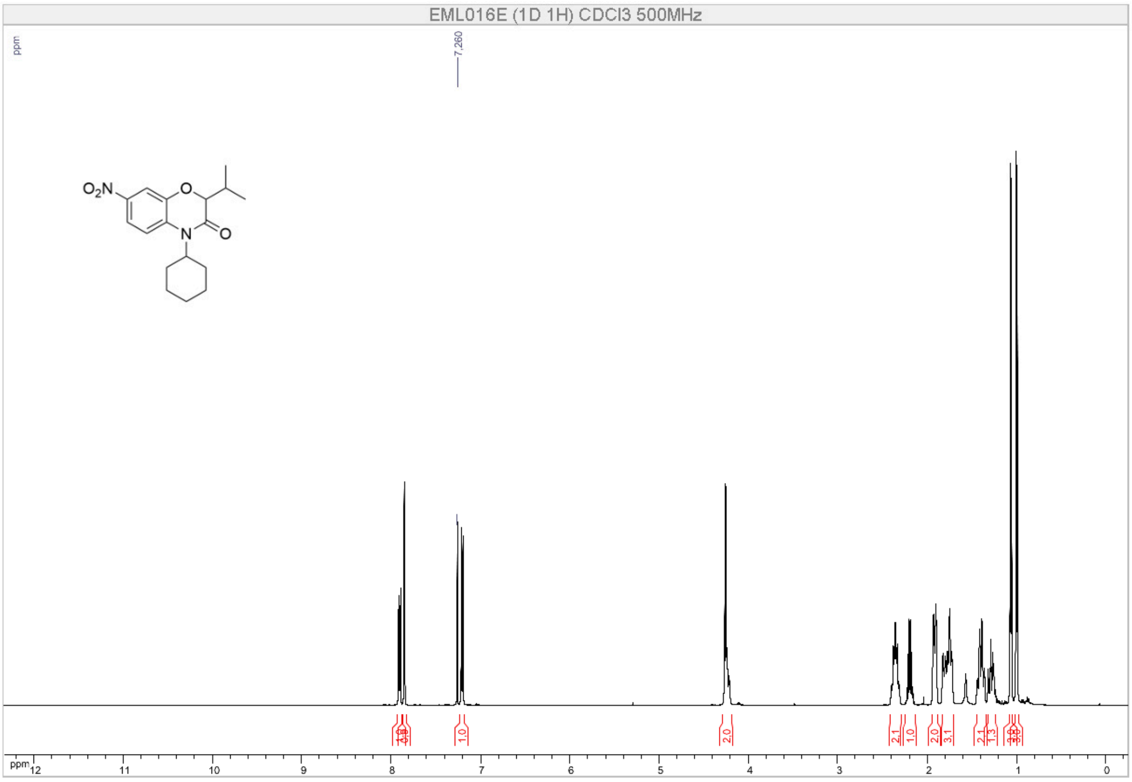

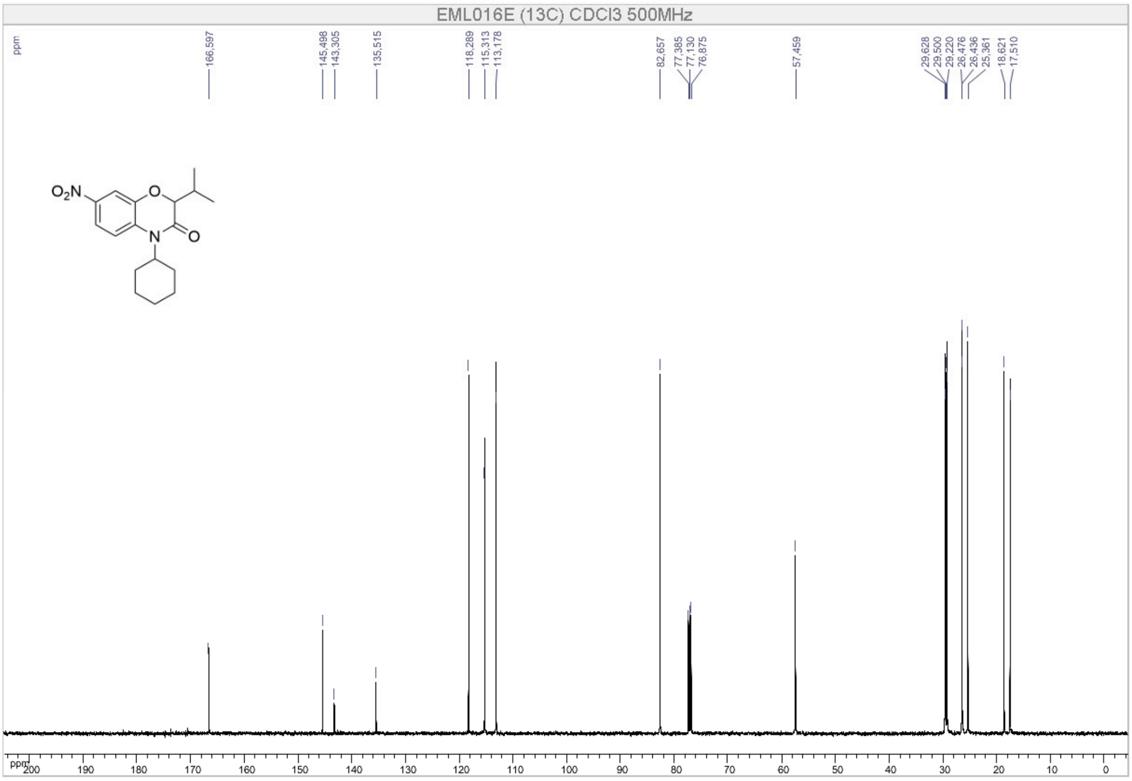

3. X-ray Crystallography

Details on X-ray analyses for compounds **1b**, **6b**, **6m**, and **6u** were provided elsewhere [1] and related crystallographic models were deposited in the Cambridge Structural Database [2], with the following CSD refcodes XISYIJ, XISYOP, XISZAC, and XISYUV, respectively.

For the seven newly obtained structures, they were all derived from X-ray analyses upon single crystals grown during slow evaporation of dichloromethane, or diisopropyl ether for EM016E. For five of them (**2h**, **2j**, **5i**, **5j** and **6w**) data collections were carried out by means of an Enraf–Nonius Kappa-CCD diffractometer using graphite-monochromated Mo-$K\alpha$ (λ= 0.71073 Å) radiation at ambient temperature. The determination of crystal class and unit cell parameters was carried out by the COLLECT program package [3] running the Denzo-HKL2000 program [4]. The raw frame data were integrated using Denzo, then scaled and reduced after semi-empirical absorption correction using Scalepack [4] to yield a unique reflection data file. For the last compound **8j**, the tested crystal was mounted on a chi-partial, three axes goniometer of a Rigaku MM007 HF copper (λ = 1.54187 Å) rotating-anode diffractometer, equipped with Osmic CMF optics and a Rapid II curved Image Plate. All structures were solved by Direct Methods with the SHELX-S97 structure solution program [5] and refined with the SHELX-L2014 refinement package [6] on F^2 anisotropically for all the non-hydrogen atoms by the full-matrix least-squares method. Most of the H atoms attached to C atoms were located from difference Fourier maps in the final stages of refinement, but all were introduced in their idealized positions and treated as riding, with **U**iso (H) =1.2**U**eq (C) or 1.5 if methyl C atoms. H atoms attached to N atoms if present were located from difference Fourier maps and refined with restrained N-H distances in **2h** and **5j** (if not for **2j**). With respect to **5j**, residual electron density (≥e.Å^{-3}) over the methyl group may suggest an exchange disorder between the methyl group and the CF3 group, both attached at the sp3 C2 atom. This exchange turns out to be extremely minor with site-occupancy factors of 0.98/0.02, leading to leave the minor-occupied orientation with isotropic atomic displacement parameters and restrained bond distances (SADI, standard su). In absence of strong anomalous signal, the absolute structure parameter is meaningless (su of 0.5) in the case of the compound **6w** that crystallized in the non-centrosymmetric space group, Pna21.

Thermal ellipsoid plots of the molecular structures were made using MERCURY [7], the ellipsoids enclosing 50 % of the electronic density.

Crystallographic data have been deposited with the Cambridge Crystallographic Data Centre as supplementary publication nos. CCDC-1054699 (**2h**), CCDC-1054700 (**2j**), CCDC-1054701 (**5i**), CCDC-1054702 (**5j**), CCDC-1054703 (**6w**) and CCDC-1054704 (**8j**). These data can be obtained free of charge from the Cambridge Crystallographic Data Centre via www.ccdc.cam.ac.uk/data_request/cif.

**Identification Code**	**1b**	**2h**	**2j**	**5i**
Chemdraw Drawing	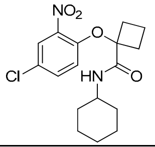	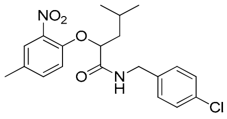	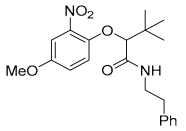	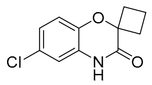
Ortep view	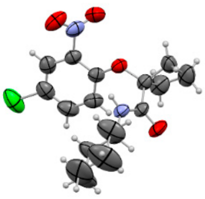	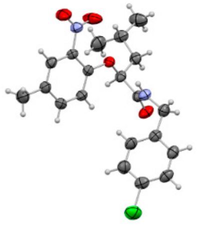	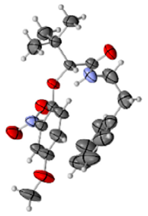	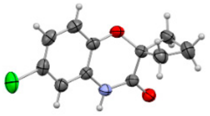
**CCDC code**	**XISYIJ**	**1054699**	**1054700**	**1054701**
Empirical formula	$C_{17}H_{21}ClN_2O_4$	$C_{20}H_{23}ClN_2O_4$	$C_{21}H_{26}N_2O_5$	$C_{11}H_{10}ClNO_2$
Formula weight	352.81	390.85	386.44	223.65
Temperature (K)	293(2)	293(2)	293(2)	293(2)
Wavelength (Å)	0.71073	0.71073	0.71073	0.71073
Recrystallization solvent	Isopropyl oxide	CH_2Cl_2	CH_2Cl_2	CH_2Cl_2
Crystal system, space group	Monoclinic, $P2_1/c$	Monoclinic, $P2_1/c$	Triclinic, P-1	Monoclinic, C2/c
a (Å)	11.688(1)	11.668(2)	9.248(2)	18.292(2)
b (Å)	17.404(3)	16.195(3)	10.596(2)	5.251(1)
c (Å)	9.965(2)	10.603(2)	11.804(4)	21.525(3)
α (°)	90	90	91.001(2)	90
β (°)	113.76(5)	103.476(4)	109.887(2)	100.06(2)
γ (°)	90	90	103.196(4)	90
Volume ($Å^3$)	1855.2(8)	1948.4(6)	1053.3(5)	2035.7(5)
Z,	4,	4,	2,	8,
Calc. density (Mg/m^3)	1.263	1.332	1.218	1.459
Abs. coefficient (mm^{-1})	0.228	0.224	0.087	0.352
F(000)	744	824	412	928
Crystal size (mm)	0.53 × 0.36 × 0.14	0.45 × 0.31 × 0.15	0.52 × 0.40 × 0.18	0.580 × 0.280 × 0.180
θ range for data coll. (°)	3.266 to 25.362	2.515 to 26.117	3.417 to 25.298	4.042 to 27.502
Limiting indices	$-14 \leq h \leq 14$, $-20 \leq k \leq 19$, $-11 \leq l \leq 12$	$-14 \leq h \leq 14$, $-18 \leq k \leq 20$, $-13 \leq l \leq 13$	$-11 \leq h \leq 11$, $-12 \leq k \leq 12$, $-14 \leq l \leq 14$.	$-23 \leq h \leq 23$, $-6 \leq k \leq 6$, $-27 \leq l \leq 27$
Reflections collected/unique	22970 / 3381 [R(int) = 0.0219]	20616 / 3854 [R(int) = 0.0205]	17230 / 3778 [R(int) = 0.0172]	8120 / 2263 [R(int) = 0.0298]
Completeness to θ_{max} (%)	99.5	99.6	98.9	97.0
Absorption correction		Semi-empirical from equivalents		
Max. and min. transm.	0.96 and 0.88	0.97 and 0.88	0.98 and 0.94	0.94 and 0.79
Refinement method		Full-matrix least-squares on F^2		
Data/restr./param.	3381/21/231	3846/1/251	3767/0/261	2261/1/140
Goodness-of-fit on F^2	1.037	1.035	1.043	1.062
Final R indices [$I > 2\sigma(I)$]	R1 = 0.0497, wR2 = 0.1335	R1 = 0.0430, wR2 = 0.1065	R1 = 0.0465, wR2 = 0.1201	R1 = 0.0433, wR2 = 0.1110
R indices (all data)	R1 = 0.0762, wR2 = 0.1527	R1 = 0.0656, wR2 = 0.1195	R1 = 0.0637, wR2 = 0.1331	R1 = 0.0562, wR2 = 0.1202
Extinction coefficient	0.036(5)	0.014(3)	0.092(13)	0.020(4)
Largest diff. peak and hole (e. $Å^{-3}$)	0.197 and −0.228	0.208 and −0.266	0.191 and −0.143	0.236 and −0.267

Identification Code	5j	6b	6m	6u
Chemdraw Drawing	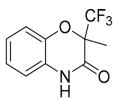	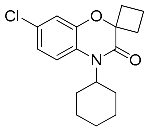	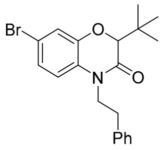	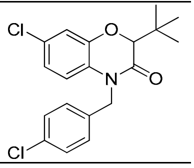
Ortep view	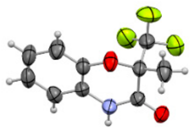	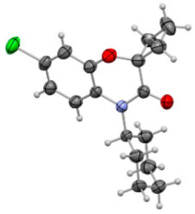	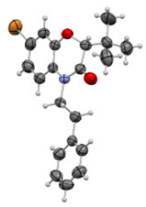	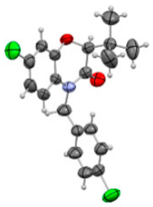
**CCDC code**	**1054702**	**XISYOP**	**XISZAC**	**XISYUV**
Empirical formula	$C_{10}H_8F_3NO_2$	$C_{17}H_{20}ClNO_2$	$C_{20}H_{22}BrNO_2$	$C_{19}H_{19}Cl_2NO_2$
Formula weight	231.17	305.79	388.30	364.25
Temperature (K)	293(2)	293(2)	293(2)	293(2)
Wavelength (Å)	0.71073	0.71073	0.71073	0.71073
Recrystallization solvent	CH_2Cl_2	CH_2Cl_2	CH_2Cl_2	CH_2Cl_2
Crystal system, space group	Monoclinic, C2/c	Triclinic, P -1	Monoclinic, P 2_1/c	Monoclinic, P 2_1/c
a (Å)	20.936(2)	5.880(1)	7.217(1)	5.954(1)
b (Å)	5.538(1)	9.120(2)	11.276(2)	8.628(2)
c (Å)	18.616(2)	14.895(2)	22.803(3)	34.703(5)
α (°)	90	77.537(2)	90	90
β (°)	112.33(2)	85.432(3)	92.725(3)	93.476(2)
γ (°)	90	77.725(3)	90	90
Volume (Å^3)	1996.5(5)	761.6(2)	1853.6(5)	1779.5(6)
Z,	8,	2,	4,	4,
Calc. density (Mg/m^3)	1.538	1.333	1.391	1.360
Abs. coefficient (mm^{-1})	0.144	0.255	2.229	0.376
F(000)	944	324	800	760
Crystal size (mm)	0.59 × 0.56 × 0.37	0.560 × 0.360 × 0.100	0.51 × 0.28 × 0.24	0.30 × 0.30 × 0.30
θ range for data coll. (°)	3.716 to 25.342	3.548 to 26.338	3.354 to 25.507	3.33 to 26.23
Limiting indices	-24 ≤ h ≤ 24, -6 ≤ k ≤ 6, -22 ≤ l ≤ 22	-7 ≤ h ≤ 6, -11 ≤ k ≤ 11, -18 ≤ l ≤ 18	-8 ≤ h ≤ 8, -12 ≤ k ≤ 13, -27 ≤ l ≤ 27	-7 ≤ h ≤ 7, -10 ≤ k ≤ 9, -42 ≤ l ≤ 42
Reflections collected/unique	22412/1797 [R(int) = 0.0368]	6531/3038 [R(int) = 0.0237]	11449/3395 [R(int) = 0.0350]	9987 / 3429 [R(int) = 0.0279]
Completeness to θ_{max} (%)	98.3	97.9	98.7	95.9
Absorption correction	Semi-empirical from equivalents			
Max. and min. transm.	0.95 and 0.90	0.98 and 0.92	0.58 and 0.44	0.89 and 0.78
Refinement method	Full-matrix least-squares on F^2			
Data / restr. / param.	1797/7/166	3035/0/190	3391/0/221	3420/0/221
Goodness-of-fit on F^2	1.047	1.017	1.027	1.032
Final R indices [I > 2σ(I)]	R1 = 0.0514, wR2 = 0.1335	R1 = 0.0449, wR2 = 0.1098	R1 = 0.0473, wR2 = 0.1178	R1 = 0.0473, wR2 = 0.1199
R indices (all data)	R1 = 0.0786, wR2 = 0.1490	R1 = 0.0615, wR2 = 0.1217	R1 = 0.0770, wR2 = 0.1362	R1 = 0.0773, wR2 = 0.1357
Extinction coefficient	0.011(3)	-	0.014(2)	0.024(4)
Largest diff. peak and hole (e. Å^{-3})	0.197 and −0.204	0.196 and −0.320	0.448 and −0.452	0.226 and −0.219

**Identification Code**	**6w**	**8j**
Chemdraw Drawing	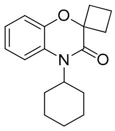	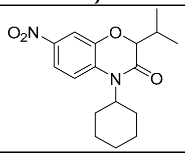
Ortep view	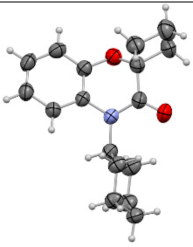	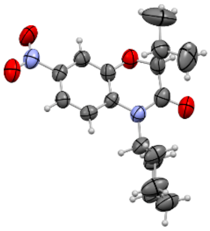
**CCDC code**	**1054703**	**1054704**
Empirical formula	$C_{17}H_{21}NO_2$	$C_{17}H_{22}N_2O_4$
Formula weight	271.35	318.36
Temperature (K)	293(2)	293(2)
Wavelength (Å)	0.71073	1.54187
Recrystallization solvent	CH_2Cl_2	diisopropyl ether
Crystal system, space group	Orthorhombic, $Pna2_1$	Monoclinic, C2/c
a (Å)	12.696(2)	29.153(2)
b (Å)	7.067(1)	9.0210(7)
c (Å)	16.318(3)	12.7261(9)
α (°)	90	90
β (°)	90	93.001(7)
γ (°)	90	90
Volume (Å^3)	1464.1(4)	3342.2(4)
Z,	4,	8,
Calc. density (Mg/m^3)	1.231	1.265
Abs. coefficient (mm^{-1})	0.080	0.743
F(000)	584	1360
Crystal size (mm)	0.58 × 0.56 × 0.50	0.31 × 0.20 × 0.08
θ range for data coll. (°)	3.528 to 28.696	3.036 to 68.142
Limiting indices	$-16 \le h \le 17$, $-9 \le k \le 9$, $-22 \le l \le 21$	$-34 \le h \le 34$, $-6 \le k \le 10$, $-14 \le l \le 14$
Reflections collected/unique	15986/3451 [R(int) = 0.031]	6382/2934 [R(int) = 0.0409]
Completeness to θ_{max} (%)	98.5	96.1
Absorption correction	Semi-empirical from equivalents	
Max. and min. transm.	0.96 and 0.91	0.94 and 0.76
Refinement method	Full-matrix least-squares on F^2	
Data/restr./param.	3451/1/181	2929/0/210
Goodness-of-fit on F^2	1.071	1.017
Final R indices [$I > 2\sigma(I)$]	R1 = 0.0412, wR2 = 0.0960	R1 = 0.0662, wR2 = 0.1573
R indices (all data)	R1 = 0.0526, wR2 = 0.1034	R1 = 0.1573, wR2 = 0.2285
Extinction coefficient	-	-
Absolute structure parameter	0.032(480)	-
Largest diff. peak and hole (e. Å^{-3})	0.114 and −0.118	0.227 and −0.249

Kinetic studies

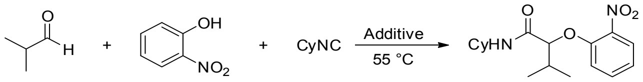

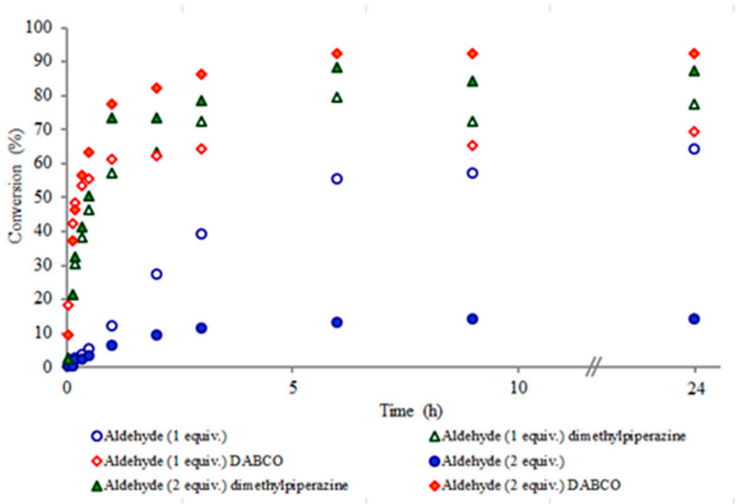

Reference

1. Martinand-Lurin, E.; Dos Santos, A.; El Kaim, L.; Grimaud, L.; Retailleau, P. Double Smiles rearrangement of Passerini adducts towards benzoxazinones. *Chem. Commun.* **2014**, *50*, 2214–2217
2. Groom, C.R.; Allen, F.H. The Cambridge Structural Database in retrospect and prospect. *Angew. Chem. Int. Ed.* **2014**, *53*, 662–671.
3. Nonius. *COLLECT*. 1998. Nonius BV, Delft, The Netherlands.
4. Otwinowski, Z.; Minor, W. *Macromolecular Crystallography. Part A. Methods in Enzymology*; Charles, W.C. Jr., Robert, M.S., Eds.; New York: Academic Press: New York, NY, USA, 1997; Volume 276, 307–326.
5. Sheldrick, G.M. A short history of SHELX. *Acta Cryst.* **2008**, *A64*, 112–122.
6. Sheldrick, G.M. Crystal structure refinement with *SHELXL*. Acta Cryst. **2015**, *C71*, 3–8.
7. Macrae, C.F.; Edgington, P.R.; McCabe, P.; Pidcock, E.; Shields, G.P.; Taylor, R.; Towler, M.; van de Streek, J. *Mercury*: Visualization and analysis of crystal structures. *J. Appl. Cryst.* **2006**, *39*, 453–457.
